# Integrated molecular landscape of Parkinson’s disease

**DOI:** 10.1038/s41531-017-0015-3

**Published:** 2017-04-10

**Authors:** C. J. H. M. Klemann, G. J. M. Martens, M. Sharma, M. B. Martens, O. Isacson, T. Gasser, J. E. Visser, G. Poelmans

**Affiliations:** 1 Department of Molecular Animal Physiology, Donders Institute for Brain, Cognition and Behaviour, Radboud Institute for Molecular Life Sciences (RIMLS), Radboud University, Nijmegen, The Netherlands; 2 Centre for Genetic Epidemiology, Institute for Clinical Epidemiology and Applied Biometry, University of Tübingen, Tübingen, Germany; 3 Department of Neuroinformatics, Donders Institute for Brain, Cognition and Behaviour, Radboud University, Nijmegen, The Netherlands; 4 Neuroregeneration Research Institute, McLean Hospital/Harvard Medical School, Belmont, MA USA; 5 Department of Neurodegenerative Diseases, Hertie-Institute for Clinical Brain Research, University of Tübingen, and German Center for Neurodegenerative Diseases (DZNE), Tübingen, Germany; 6 Department of Neurology, Donders Institute for Brain, Cognition and Behaviour, Radboud University Medical Center, Nijmegen, The Netherlands; 7 Department of Neurology, Amphia Hospital, Breda, The Netherlands; 8 Department of Cognitive Neuroscience, Donders Institute for Brain, Cognition and Behaviour, Radboud University Medical Center, Nijmegen, The Netherlands; 9 Department of Human Genetics, Radboud University Medical Center, Nijmegen, The Netherlands

## Abstract

Parkinson’s disease is caused by a complex interplay of genetic and environmental factors. Although a number of independent molecular pathways and processes have been associated with familial Parkinson’s disease, a common mechanism underlying especially sporadic Parkinson’s disease is still largely unknown. In order to gain further insight into the etiology of Parkinson’s disease, we here conducted genetic network and literature analyses to integrate the top-ranked findings from thirteen published genome-wide association studies of Parkinson’s disease (involving 13.094 cases and 47.148 controls) and other genes implicated in (familial) Parkinson’s disease, into a molecular interaction landscape. The molecular Parkinson’s disease landscape harbors four main biological processes—oxidative stress response, endosomal-lysosomal functioning, endoplasmic reticulum stress response, and immune response activation—that interact with each other and regulate dopaminergic neuron function and death, the pathological hallmark of Parkinson’s disease. Interestingly, lipids and lipoproteins are functionally involved in and influenced by all these processes, and affect dopaminergic neuron-specific signaling cascades. Furthermore, we validate the Parkinson’s disease -lipid relationship by genome-wide association studies data-based polygenic risk score analyses that indicate a shared genetic risk between lipid/lipoprotein traits and Parkinson’s disease. Taken together, our findings provide novel insights into the molecular pathways underlying the etiology of (sporadic) Parkinson’s disease and highlight a key role for lipids and lipoproteins in Parkinson’s disease pathogenesis, providing important clues for the development of disease-modifying treatments of Parkinson’s disease.

## Molecular interactions: the importance of fats

Lipids and lipoproteins play a central role in four key biological processes underlying Parkinson’s disease (PD). Using bioinformatics and other extensive analyses of previously published data, Geert Poelmans, Cornelius Klemann and colleagues in The Netherlands, Germany and the USA have mapped the interactions of proteins that are encoded by genes associated with both familial and sporadic forms of PD. They identify the oxidative stress response, lysosomal function, endoplasmic reticulum stress response and immune response activation as the main mechanisms leading to the death of dopaminergic neurons. Lipid signaling is implicated in all four of these processes and the authors find a link between the levels of particular lipids and lipoproteins and the risk of PD. These findings suggest that compounds that regulate lipid or lipoprotein levels offer a potential new treatment strategy for PD.

## Introduction

Parkinson’s disease (PD) is the second most common neurodegenerative disease, with an estimated prevalence of 0.3%, affecting 1–2% of people over 60 years of age.^1, 2^ The pathological hallmark of PD is loss of dopaminergic (DA) neurons in the substantia nigra (SN), and the presence of protein aggregates (i.e., Lewy bodies) involving synuclein alpha (SNCA) in the residual DA neurons.^3^ A number of biological processes that contribute to the pathogenesis of PD have been identified, including defects in mitochondrial function,^4^ oxidative stress,^5^ and protein aggregation.^6–8^ However, detailed insights into the molecular mechanisms underlying these processes, and how they interact with each other, are essentially lacking. In many studies exploring PD pathogenesis, familial PD genes served as starting point. Thus far, at least eighteen genetic loci for familial PD have been found, and twelve familial PD candidate genes have been identified (*ATP13A*2, *DJ-1*, *DNAJC6*, *EIF4G1*, *FBXO7*, *LRRK2*, *PARK2*, *PINK1*, *PLA2G6*, *SNCA*, *SYNJ1*, and *VPS35*) (refs 9, 10). However, as a mutation in one of these familial genes is found in only 5–10% of the cases, PD should be considered a predominantly sporadic disease,^11, 12^ with both genetic and environmental contributing risk factors. In recent years, 15 genome-wide association studies (GWASs) have investigated genetic risk factors for sporadic PD^13–26^ but the functional coupling of the proteins encoded by the GWAS-identified candidate genes to PD pathophysiology is often not clear. In the present study, we aimed to identify the core mechanisms underlying PD pathogenesis by using bioinformatics and extensive literature analyses to integrate (1) the genes corresponding to the top-ranked single-nucleotide polymorphisms (SNPs) found in published GWASs of sporadic PD, and (2) other PD candidate genes (e.g., familial PD genes) into a protein interaction landscape. This molecular landscape allowed us to identify the specific biological processes that are key in PD pathogenesis and provides clues for the development of novel PD treatment strategies.

## Results

### Selected PD GWAS genes and genetic network enrichment analysis

Thirteen of the fifteen published PD GWASs met our inclusion criteria (Supplementary Table 1) and were used to select a total of 451 PD GWAS candidate genes based on SNPs with *p* < 0.0001 (Supplementary Table 2). Of the five most significantly enriched ingenuity pathway analysis (IPA) networks (Supplementary Table 3), the network with the highest enrichment score (*p* = 1.00E-44) and the highest number of PD GWAS candidate gene-encoded proteins (28 proteins) served as the starting point for the building of the molecular landscape (Supplementary Fig. 1).

### The molecular landscape of PD

Guided by the most significantly enriched genetic network and extensive literature searches, we built a molecular landscape consisting of 260 interacting proteins (i.e., encoded by approximately 58% of the 451 PD GWAS genes, Supplementary Table 2), 128 proteins implicated in PD through other evidence (Supplementary Table 4) and 49 proteins that have not been directly linked to PD (yet) but have multiple functional interactions within the landscape (Supplementary Table 4). Approximately one in three landscape proteins are implicated in PD etiology through at least two types of evidence.

Supplementary Figures 2 and 3 show all relevant protein interactions in the PD landscape that are functionally involved in four main biological processes:oxidative stress response, endosomal-lysosomal functioning, endoplasmic reticulum (ER) stress response, and neuron death and immune response. The Supplementary Information provides a detailed and referenced description of the evidence linking all the proteins in the landscape. In Supplementary Table 5, we have indicated in which process(es) each landscape protein exerts its main effect and where it is located in Supplementary Figs 2 and/or 3.

**Fig. 1:**
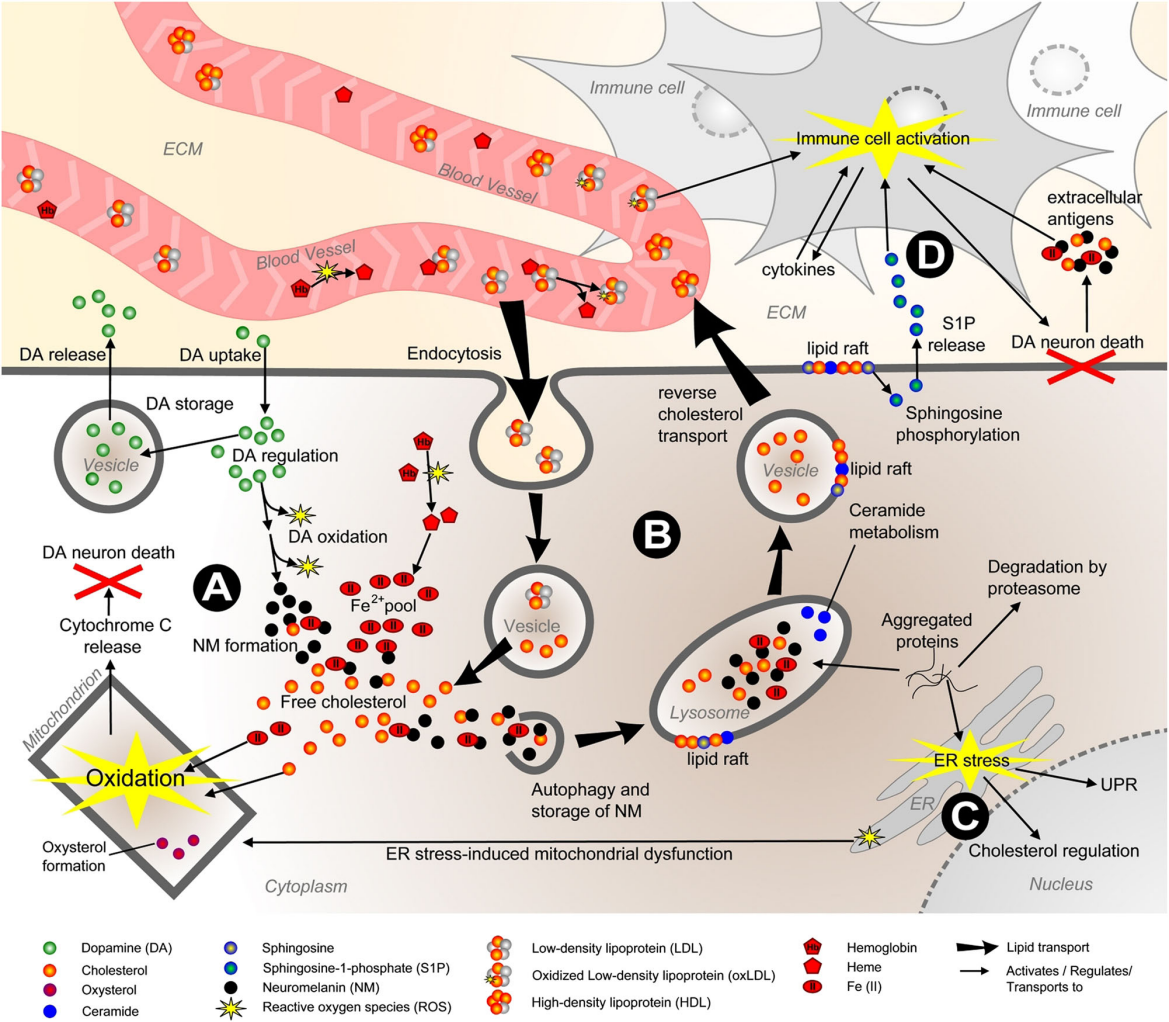
Overview of the molecular landscape of PD. The four main biological processes in the PD landscape—oxidative stress response (Fig. 1a), endosomal-lysosomal functioning (Fig. 1b), endoplasmic reticulum (*ER*) stress response (Fig. 1c), and neuron death and immune response (Fig. 1d)—are depicted. *ECM* extracellular matrix, *ER* endoplasmic reticulum, *UPR* unfolded protein response

The above being said, we here give a succinct description of the four main biological processes and signaling cascades in the PD landscape that are depicted in Fig. 1. Central in the landscape is signaling involving lipoproteins—i.e., low-density lipoprotein (LDL), high-density lipoprotein (HDL) and very low-density lipoprotein (VLDL)—and their component lipids and metabolites (e.g., cholesterol, oxysterols, sphingolipids such as ceramide and sphingosine, and triglycerides). Lipid and lipoprotein signaling represents the ‘‘common denominator’’ that functionally integrates, regulates and is regulated by the four landscape processes (Fig. 1a–d). Either by themselves or in combination, deficits or impairments in any of these four processes—each composed of multiple signaling cascades—can contribute to the degeneration and ultimately death of DA neurons.

First, deficits or impairments in dopamine synthesis and—linked to this—iron metabolism can cause an increased oxidative stress response (Fig. 1a). Dopamine can be either taken up through active transport or is newly synthesized in neurons and can subsequently be re-released (through vesicular exocytosis), degraded or (auto-)oxidized into neuromelanin (NM). Further, like erythrocytes (see below), SN DA neurons have a high-oxygen demand and express oxygen-carrying hemoglobin.

Through oxidation, cytotoxic heme is released from hemoglobin and then converted in DA neurons to ferrous iron, Fe(II). Fe(II) increases oxidative stress and together with free cholesterol—that is taken up by neurons through lipoproteins (see below)—induces mitochondrial oxysterol formation. In turn, this causes mitochondrial dysfunction and triggers the release of pro-apoptotic cytochrome c and, eventually, neuron death.

The second main landscape process centers around the (dys) regulation of endosomal-lysosomal functioning (Fig. 1b). Neuronal uptake of cholesterol occurs through the endosomal system, i.e., after neuronal uptake through vesicular endocytosis, LDL particles are processed into their composite parts: proteins, free cholesterol and other lipids. Free cholesterol and Fe(II) are bound in complexes by NM, which are then stored in lysosomes through autophagy. Hence, NM complex formation prevents the above described Fe(II)- and cholesterol-induced oxidative stress response. Moreover, their ageing-related increase in NM content and the associated increased demands on lysosomal function renders DA neurons particularly vulnerable to lysosomal defects. Other important lysosomal functions include the degradation of misfolded or aggregated proteins (such as pathological SNCA aggregates), the regulation of ceramide metabolism and reverse cholesterol transport, i.e., the vesicle-mediated transport and exocytosis of cholesterol into HDL particles in the bloodstream (and back to the liver). As such, a defect in any of these endosomal-lysosomal system components results in disturbed levels of lipids such as cholesterol and ceramide. In turn, these disturbed lipid levels affect membrane function in general and more specifically the functioning of so-called lipid rafts—microdomains of the vesicular, lysosomal, and plasma membrane containing high amounts of cholesterol and sphingolipids and crucial for membrane function—and hence processes such as autophagy, endo- and exocytosis. Deficient lysosomal function together with reduced degradation by the proteasome also leads to misfolded or aggregated protein formation.

Misfolded/aggregated proteins trigger the ER stress response (Fig. 1c), the third main landscape process, and subsequent activation of the protective unfolded protein response (UPR) as well as stimulation of cholesterol influx through upregulating the expression of key lipoprotein receptors. Prolonged ER stress that can no longer be counteracted by the UPR induces mitochondrial dysfunction, which eventually results in DA neuron death.

Lastly, apart from or in addition to dysregulated processes *within* DA neurons (as described above), DA neuron death can be the consequence of *external* factors such as an exaggerated immune response (Fig. 1d), the fourth landscape process. In this respect, immune cells are activated and attracted to damaged or already dying DA neurons by extracellular factors such as the sphingolipid-derived sphingosine-1-phosphate (S1P), triglyceride-rich extraneuronal VLDL particles (not shown), heme-oxidized LDL (oxLDL, see above), and various cytokines. Subsequently, the damaged/dying DA neurons are removed by the activated immune cells, an essentially normal and adequate response that is exaggerated in PD by DA neuron-specific antigens such as SNCA aggregates and NM complexes—released by dying DA neurons—creating a vicious cycle of DA neuron death and immune cell activation.

### Polygenic risk score (PRS) analyses

**Fig. 2:**
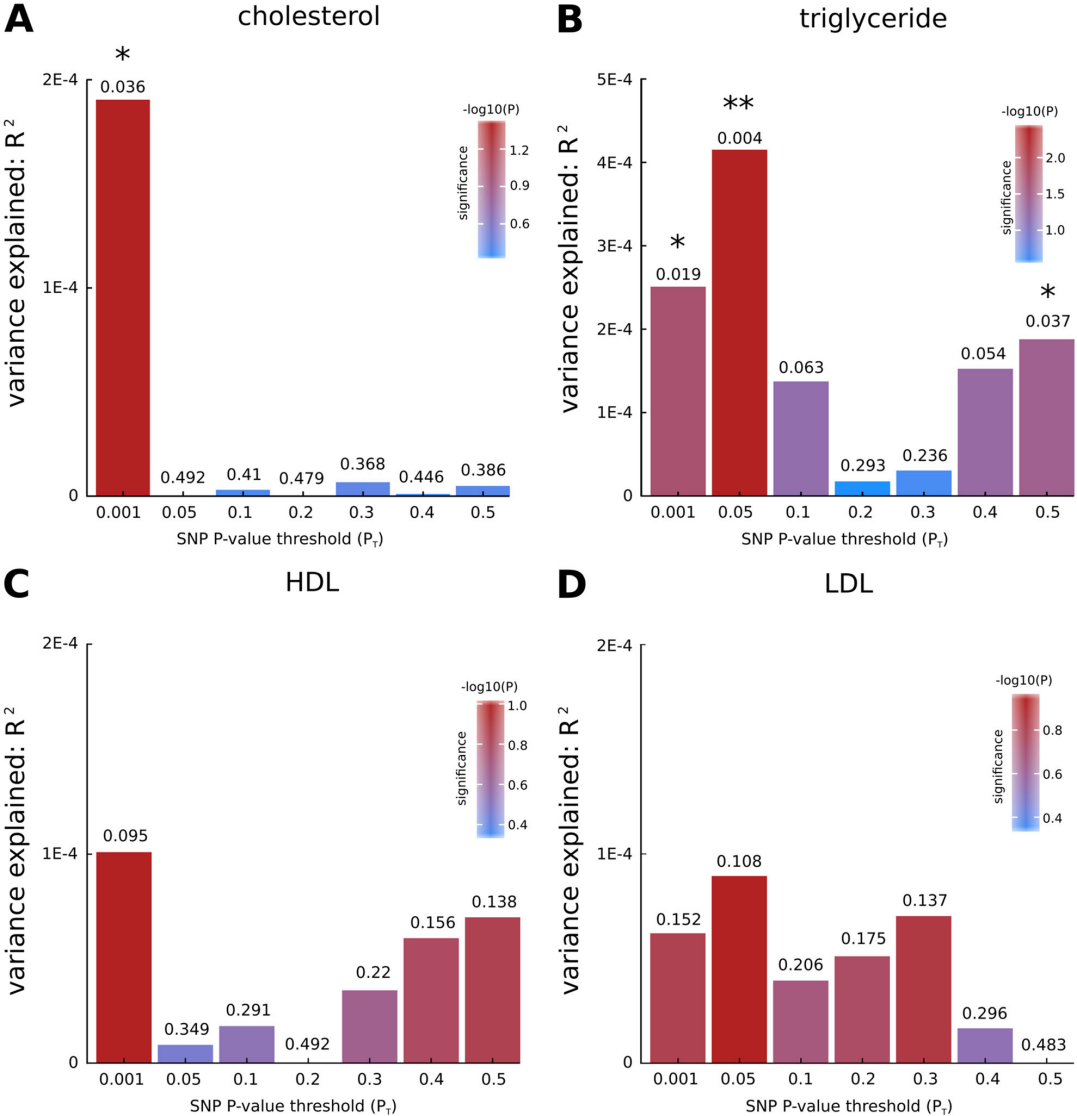
Bar plots from PRSice for shared genetic etiology between four lipid and lipoprotein traits (cholesterol, triglyceride, HDL, and LDL levels) and PD showing results at broad *p*-value thresholds (*p*_T_). The numbers above the *bars* indicate the *p*-values for shared genetic etiology, and these *p*-values were corrected using the false discovery rate (*FDR*) method; *denotes FDR-corrected *p* < 0.05, **denotes FDR-corrected *p* < 0.01

Because our molecular landscape pointed towards an important role for lipids and lipoproteins in PD etiology (see above), we conducted PRS analyses using the tool PRSice,^27^ with GWAS data for the blood levels of various lipids and lipoproteins^28^ as base samples and meta-analytic PD GWAS data from the International Parkinson Disease Genomics Consortium (IPDGC)^29^ as target sample. We found statistically significant evidence (false discovery rate (FDR)-corrected *p* < 0.05) for a shared genetic etiology between the lipid traits ‘‘total cholesterol levels’’ and ‘‘total triglyceride levels’’ and PD, with the most predictive *p*-value threshold (*p*_T_) at 0.001 and 0.05, respectively (Fig. 2). In contrast, the lipoprotein traits ‘‘total HDL levels’’ and ‘‘total LDL levels’’ yielded no evidence for a shared genetic risk with PD (Fig. 2). For the various combinations of increased or decreased HDL and LDL levels, we found significant evidence for a shared genetic etiology between PD and the combined trait ‘‘increased HDL + increased LDL’’ (most predictive *p*_T_ = 0.05) (Supplementary Fig. 4).

## Discussion

In this study, we integrated the available genetic and expression data with data about environmental risk factors into a protein interaction landscape that reveals the main biological processes and signaling cascades that are affected in PD and occur in DA neurons and immune cells. Our PD landscape represents processes and cascades that are affected in both the monogenic, familial and the more prevalent polygenic, sporadic forms of PD. In this respect, the molecular landscape includes the ‘‘classic’’ processes and cascades known to be affected in PD that are based on the familial PD candidate genes (e.g., SNCA, PARK2, LRRK2): mitochondrial function, oxidative stress and protein aggregation. In addition, the landscape harbors more novel processes that have been less well studied in relation to PD pathogenesis yet, such as impairments in lysosomal function and immune response regulation. The landscape does not imply a ‘‘sequence of events’’ that leads to DA neuron loss, i.e., a number of (impaired) biological processes and cascades that occur in a temporally and/or spatially distinct order. Instead, deficits in any of the main landscape processes/cascades, either by themselves or in combination with deficits of other processes/cascades, may cause DA neurons to die. Moreover, an aging-related decline in the functionality and/or efficiency of landscape processes/cascades may play a role in PD onset and progression. For example, a gradual buildup of NM or aggregated proteins may disturb lysosomal function in DA neurons^30^ or an age-related decrease in the expression and activity of ER folding enzymes can compromise proper protein folding.^31^

Lipid and lipoprotein signaling functionally integrates, regulates and is regulated by the key landscape processes and cascades. Any disturbance of these processes and cascades can (eventually) result in DA neuron death, which is further aggravated or initiated by an increased (auto-)immune response.^32^ The involvement of deficient lipid and lipoprotein signaling in PD pathophysiology is corroborated by a number of environmental risk factor studies. Increased plasma levels of total cholesterol are associated with a lower PD risk.^33–35^ Nevertheless, a recent meta-analysis did not show an effect of higher or lower dietary cholesterol intake on PD risk,^36^ suggesting that direct cholesterol intake through food may not play a major role in PD etiology. Further, low plasma levels of LDL are linked to a higher PD risk,^37, 38^ whereas high-plasma HDL and CSF oxysterol levels are associated with increased PD risk and duration.^39, 40^ In addition, the levels of *oxidized* LDL, oxysterols and sphingolipids are increased in the plasma of PD patients.^41–43^ Thus, PD patients have a lower LDL:HDL ratio that is associated with a lower risk of cardiovascular disease (CVD)^44^ and could at least to some extent explain why the PD population is indeed less susceptible to developing CVD.^39^ Apart from the observed dysregulated levels of cholesterol (metabolites) and cholesterol-containing lipoproteins in PD patients, lower serum levels of triglycerides—which are highly enriched in VLDL particles—associate with an increased PD risk.^45, 46^

Intriguingly, we found a significant overlap between the polygenic risk associated with total cholesterol and triglyceride levels and PD. We also identified a shared genetic etiology between the combined lipoprotein trait ‘‘increased HDL + increased LDL’’ and PD. To our knowledge, we are the first to find a shared genetic risk between quantitative traits and a neurodegenerative disease but these findings need to be replicated in larger data sets, especially for the target sample, i.e., the PD GWAS data set. Together, the epidemiological and our PRS analysis findings indicate that the link between specific lipid/lipoprotein traits and PD may be the result of both shared environmental and genetic risk factors.

Given the converging evidence for lipid and lipoprotein signaling playing a key role in PD etiology, compounds that modulate lipid/lipoprotein levels could represent effective novel PD treatments. In this respect, statins—inhibitors of peripheral cholesterol synthesis that are used to treat hypercholesterolemia and hence prevent cholesterol-associated CVD—have a neuroprotective effect in the rat brain,^47^ but their effect on PD risk remains unclear.^48–55^ Interestingly, the only published prospective study that has adjusted for baseline cholesterol levels before statin treatment has found that statin use is associated with a significantly higher PD risk,^35^ which is in keeping with the observation that higher total plasma cholesterol levels—which are lowered by statins—are protective against PD. Other signaling molecules from the landscape that affect cholesterol and lipoprotein levels are testosterone and vitamin D3. Caucasian male PD patients show significantly reduced testosterone levels,^56–58^ and free testosterone levels are positively correlated with LDL, HDL, and total cholesterol levels.^59^ Therefore, decreased testosterone levels may impact on several key PD landscape processes, as testosterone regulates the efflux of LDL and HDL to the circulation.^60^ Hence, testosterone could be used for treating PD in male patients and indeed, testosterone treatment has some modest beneficial effects in men with PD.^61, 62^ Deficiency of vitamin D3—which affects cholesterol metabolism through downregulating SREBF1 (ref. 63), the main transcriptional activator of lipid homeostasis and key landscape protein—has been consistently associated with an increased PD risk^64^ and its supplementation may stabilize PD symptoms.^65^

Lastly, a number of landscape proteins that both regulate lipid/lipoprotein signaling and landscape cascades involved in DA neuron death represent attractive (novel) drug targets for PD. Examples include HMOX1 that prevents oxidative stress by heme, PSAP and its receptor GPR37 that mediate ceramide metabolism, the immunity-related ICAM1 that is regulated by extracellular lipids and (oxidized) lipoproteins, and plasmin that regulates the degradation of extracellular SNCA and (lipo)proteins.

In conclusion, our integrated molecular landscape yields detailed insights into the mechanisms underlying PD pathogenesis, and highlights the involvement of deficient lipid and lipoprotein signaling. These findings warrant future rigorous perturbation experiments in PD cell and animal models that may eventually provide validated drug target ‘‘leads’’ for the development of novel disease-modifying PD treatments.

## Methods

### PD GWAS gene selection

The first step of our molecular landscape building approach^66–68^ is the selection of candidate genes based on GWAS SNPs and their corresponding *p*-values. All 15 PD GWASs published to date were considered. Criteria for inclusion were a publicly available GWAS discovery sample with all SNPs associated at *p* < 0.0001. From the GWASs for which these data were available, we then selected the SNPs that were associated with PD at *p* < 0.0001 to compile a list of associated genes. The selected genes either contained a SNP that was located within an exonic, intronic or untranslated region of the gene or were found within 100 kilobases (kb) downstream and upstream of the SNP. The latter was based on the fact that the vast majority of expression quantitative trait loci (eQTL) for a given gene are located within 100 kb downstream and/or upstream of a gene^69–71^ and because trait-associated SNPs are more likely to be eQTL.^72^ The chosen cutoff for association (*p* < 0.0001) is often employed to designate ‘‘suggestive’’ association and has been used in GWASs of multiple disorders.^73–75^ Subsequently, the literature was searched for additional (genetic) evidence linking the selected GWAS candidate genes to PD.

### Genetic network enrichment analysis

To identify enriched protein networks in the PD GWAS candidate genes, a network analysis using the IPA software package (http://www.ingenuity.com) was performed with default parameters, i.e., the analysis used the so-called reference set of known genes and endogenous chemicals. This reference set is accessible through the ‘‘Ingenuity Knowledge Base’’, a repository of data from publicly accessible databases (e.g., BioGRID, IntAct) and data that are manually curated by Ingenuity through systematically reviewing published literature. In this respect, of all protein–protein interactions between the PD candidate proteins in the most significantly enriched IPA network shown in Supplementary Fig. 1 (see Results), we found approximately 4/5 through publicly accessible databases and 1/5 by IPA. In addition, only functional relationships that are corroborated by experimental evidence were included in the IPA networks. For each network, the Ingenuity software also generates an enrichment score that takes into account the number of eligible molecules/proteins in the network and its size, as well as the total number of network-eligible molecules analyzed and the total number of molecules in the Ingenuity Knowledge Base that could potentially be included in networks. This score is the negative logarithm of the right-tailed Fisher’s exact test result.

### Molecular landscape building

Following the network enrichment analysis, the literature was extensively searched for the functions and interactions of all proteins encoded by the candidate genes implicated through PD GWASs as well as other PD candidates implicated via other evidence, including genetic association studies, messenger RNA/protein expression studies and/or functional studies. First, we used the UniProt Protein Knowledge Base (http://www.uniprot.org/uniprot)^76^ to gather basic information on the functions of all candidate genes and their encoded proteins. Subsequently and starting with the interactions in the most enriched genetic network, we used PubMed (http://www.ncbi.nlm.nih.gov/sites/entrez) to search for all functional, experimental evidence-based interactions between all PD candidate genes/proteins. While building the landscape in this way, we also included genes/proteins and metabolites that have no known link with PD, but have multiple—i.e., at least two different—functional interactions with PD-implicated proteins. Based on all gathered information, we generated a protein interaction landscape. The figures depicting this landscape were made using the drawing program Serif DrawPlus version 4.0 (www.serif.com).

### PRS analyses

Our molecular landscape pointed towards an important role for lipids and lipoproteins in PD etiology (see below). Therefore, we conducted PRS analyses using the tool PRSice,^27^ with summary statistics data from GWASs of blood levels of total cholesterol, total triglycerides, total HDL and total LDL as ‘‘base’’ samples (GWAS data for 188577 European-ancestry individuals from the general population)^28^ and summary statistics data from a meta-analytic PD GWAS by the IPDGC as ‘‘target’’ sample (GWAS data for 5333 PD cases and 12,019 healthy control subjects, all of European ancestry)^29^. Using the default settings in PRSice we calculated the shared genetic etiology between the four lipid/lipoprotein traits and PD at seven broad *p*-value thresholds (indicated by *p*_T_), which were used to select the SNPs from the base sample that were included in the PRS analysis, i.e., *p*_T_ < 0.001, 0.05, 0.1, 0.2, 0.3, 0.4, and 0.5. As such, the seven *p*_T_ thresholds led to the selection of all SNPs that were associated with the base lipid/lipoprotein phenotype at *p* < 0.001, 0.05, 0.1 etc.

The calculated *p*-values indicating the significance of a shared genetic etiology between each lipid/lipoprotein trait and PD were aggregated and corrected for multiple comparisons using the FDR method, incorporating potential dependencies between *p*-values.^77^

To calculate the FDR, we used the mafdr function in MATLAB (R2012a, The Mathworks, Natick, MA, USA) using the bootstrap selection method for the FDR parameter lambda. FDR was set to not lower *p*-values below uncorrected *p*-values, which would have occurred due to overall (relatively) low uncorrected *p*-values.

In addition to calculating the shared genetic etiology between the four lipid/lipoprotein traits and PD, we performed similar analyses using four ‘‘combined lipoprotein traits’’. In order to do this, we first divided the HDL and LDL level summary statistics GWAS data into four groups of SNPs, i.e., all SNPs associated with (1) increased HDL levels, (2) increased LDL levels, (3) decreased HDL levels, and (4) decreased LDL levels (where ‘‘increased’’ and ‘‘decreased’’ refer to all SNPs that had an effect size-indicating beta > 0 and beta < 0, respectively). Subsequently, we conducted PRSice analyses with four combined data sets as base sample, i.e., (1) all SNPs increasing HDL and LDL levels, (2) all SNPs decreasing HDL and LDL levels, (3) all SNPs increasing HDL and decreasing LDL levels, and (4), all SNPs decreasing HDL and increasing LDL levels. Before the data sets were fed into PRSice, an equivalent, weighted *p*-value for each SNP in each of the four combined HDL/LDL data sets was calculated as: $\frac{2}{{p}_{{\rm{eq}}}}=\frac{1}{{p}_{{\rm{HDL}}}}+\frac{1}{{p}_{{\rm{LDL}}}}$

## Electronic supplementary material

Supplementary Information

Supplementary Figure 2

Supplementary Figure 3
