# Supplementary material for: Integrated molecular landscape of Parkinson’s disease
Source: NPJ Parkinsons Dis. 2017 Apr 10;3:14. doi: 10.1038/s41531-017-0015-3 (PMC5460267; doi:10.1038/s41531-017-0015-3)
Supplement: Supplementary file 1 — Supplementary Information [file 41531_2017_15_MOESM1_ESM.docx]

**SUPPLEMENTARY INFORMATION**

**Integrated Molecular Landscape of Parkinson’s Disease**

C.J.H.M. Klemann, M.Sc.^1^, G.J.M. Martens, Ph.D.^1^, M. Sharma, Ph.D.^2^, M.B. Martens, M.Sc.^3^,
O. Isacson, M.D., Ph.D.^4^, T. Gasser, M.D.^5^, J.E. Visser, M.D., Ph.D.^1,6,7#^, G. Poelmans, M.D., Ph.D.^1,8,9#^

^1^Department of Molecular Animal Physiology, Donders Institute for Brain, Cognition and Behaviour, Radboud Institute for Molecular Life Sciences (RIMLS), Radboud University, Nijmegen, The Netherlands

^2^Centre for Genetic Epidemiology, Institute for Clinical Epidemiology and Applied Biometry, University of Tübingen, Germany

^3^Department of Neuroinformatics, Donders Institute for Brain, Cognition and Behaviour, Radboud University, Nijmegen, The Netherlands

^4^Neuroregeneration Research Institute, McLean Hospital/Harvard Medical School, Belmont, MA, USA

^5^Department of Neurodegenerative Diseases, Hertie-Institute for Clinical Brain Research, University of Tübingen, and German Center for Neurodegenerative Diseases (DZNE), Tübingen, Germany

^6^Department of Neurology, Donders Institute for Brain, Cognition and Behaviour, Radboud University Medical Center, Nijmegen, The Netherlands

^7^Department of Neurology, Amphia Hospital, Breda, The Netherlands

^8^Department of Cognitive Neuroscience, Donders Institute for Brain, Cognition and Behaviour, Radboud University Medical Center, Nijmegen, The Netherlands

^9^Department of Human Genetics, Radboud University Medical Center, Nijmegen, The Netherlands

# equal contribution

**Supplementary Figure 1:** Top enriched Ingenuity genetic network

**Supplementary Figure 2:** PD landscape; intracellular pathways constituting processes A-D

**Supplementary Figure 3:** PD landscape; immune cell regulation-related pathways of process D

**Supplementary Figure 4:** Additional Polygenic Risk Score Analyses

**List of abbreviations**

**Supplementary Table 1:** Overview of the published PD GWASs

**Supplementary Table 2:** List of PD GWAS candidate genes

**Supplementary Table 3:** Top Networks Ingenuity analysis

**Supplementary Table 4:** Other genes implicated in PD

**Supplementary Table 5:** Landscape proteins, the main process(es) they are functionally involved in and their location within the figures

**Detailed Description of the Molecular Landscape for Parkinson’s Disease**

**
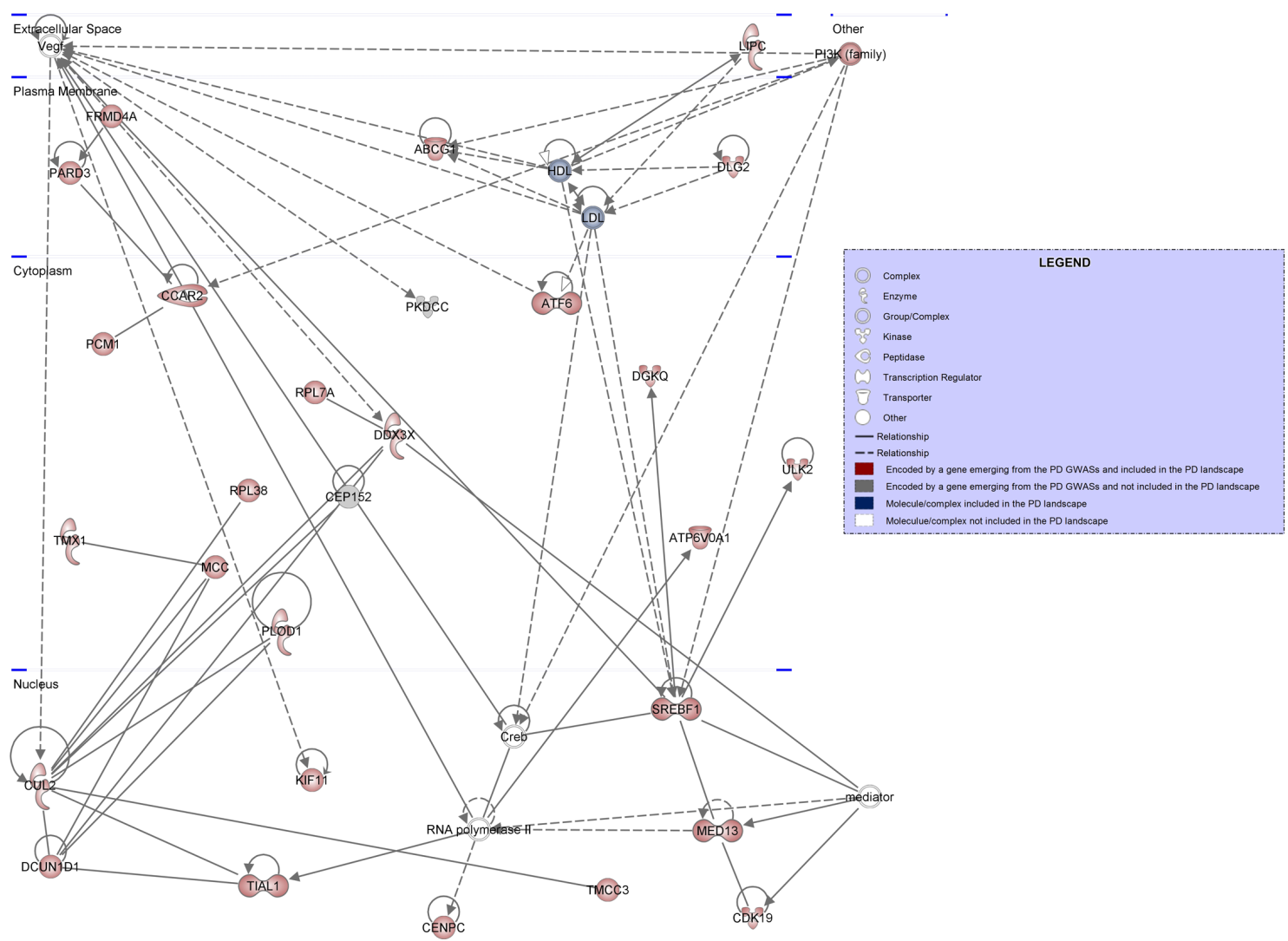
Supplementary Figure 1. Top enriched Ingenuity genetic network.** The network with the highest score (P=1.00E-44) and containing the highest number of proteins (28 proteins) obtained by a network enrichment analysis of the PD GWAS candidate genes from **Supplementary Table 2** using Ingenuity pathway software (www.ingenuity.com) (**Supplementary Table 3**). The proteins encoded by a gene that emerged from (at least) one of the PD GWASs and that were included in the PD landscape are indicated in red, whereas the PD GWAS gene-encoded proteins that are not included in the landscape are depicted in grey. Of note, two molecules – HDL and LDL – did not emerge from one of the GWASs, but are included in the landscape (in blue).

**
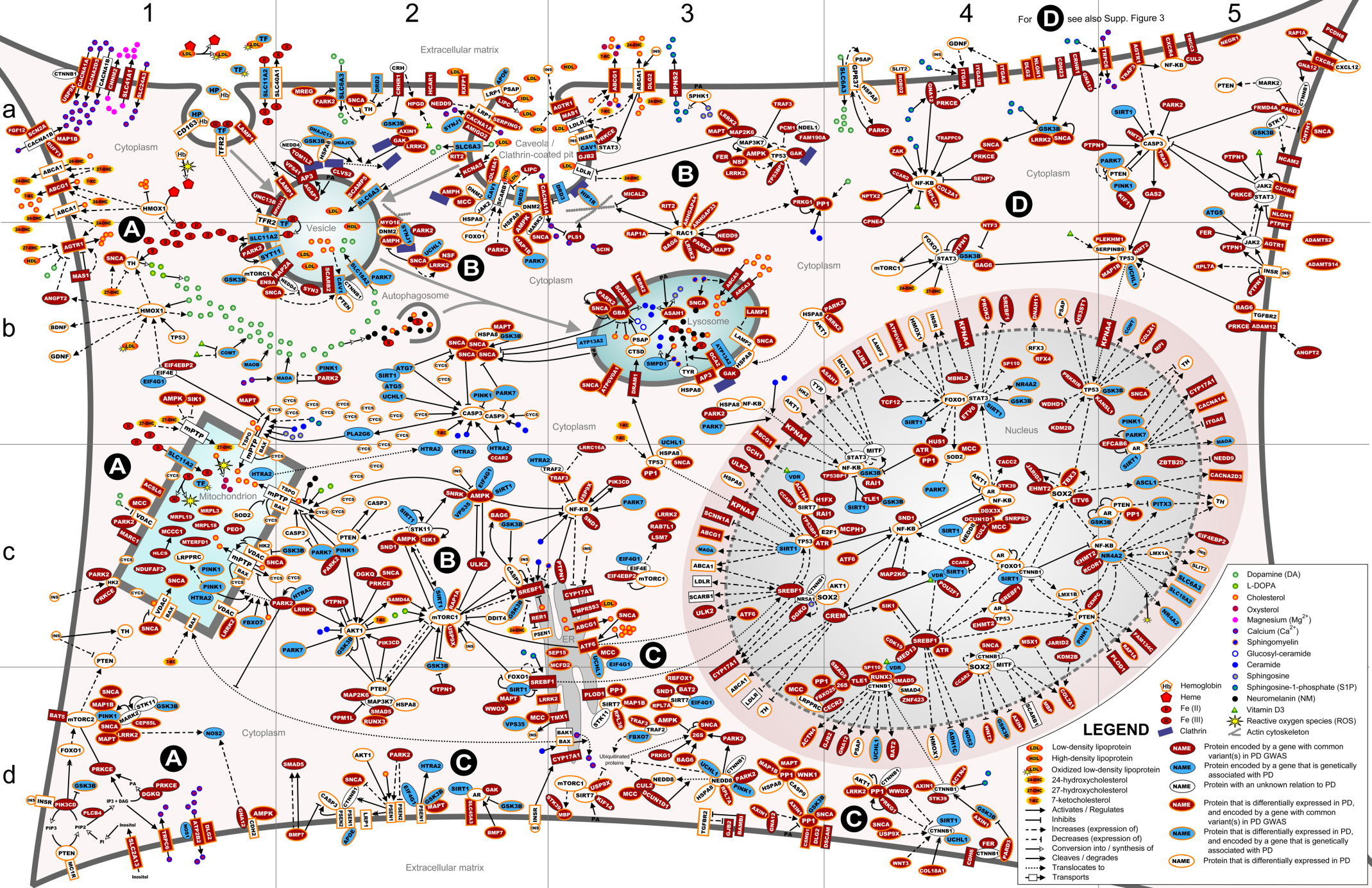
**

**Supplementary Figure 2: PD landscape; intracellular pathways constituting processes A-D.** The functional interactions between proteins functioning within the processes oxidative stress response (A), endosomal-lysosomal functioning (B), ER stress response (C) and neuron death and immune response (D) are shown. The transcription factors and transcription regulators in the nucleus regulate the expression of proteins that are involved in all four processes. These expressed proteins are shown in the vicinity of the nucleus (in the light brown band surrounding the nucleus) and will translocate to their cellular destination, i.e. either the cytoplasm, one of the organelles, or the cell membrane. The functional interactions within process D that are more directly related to immune cell regulation are shown in **Supplementary Figure 3**. For each individual protein in this figure, **Supplementary Table 5** shows the location coordinates and the main process(es) (A-D) in which they exert their main effect.

**
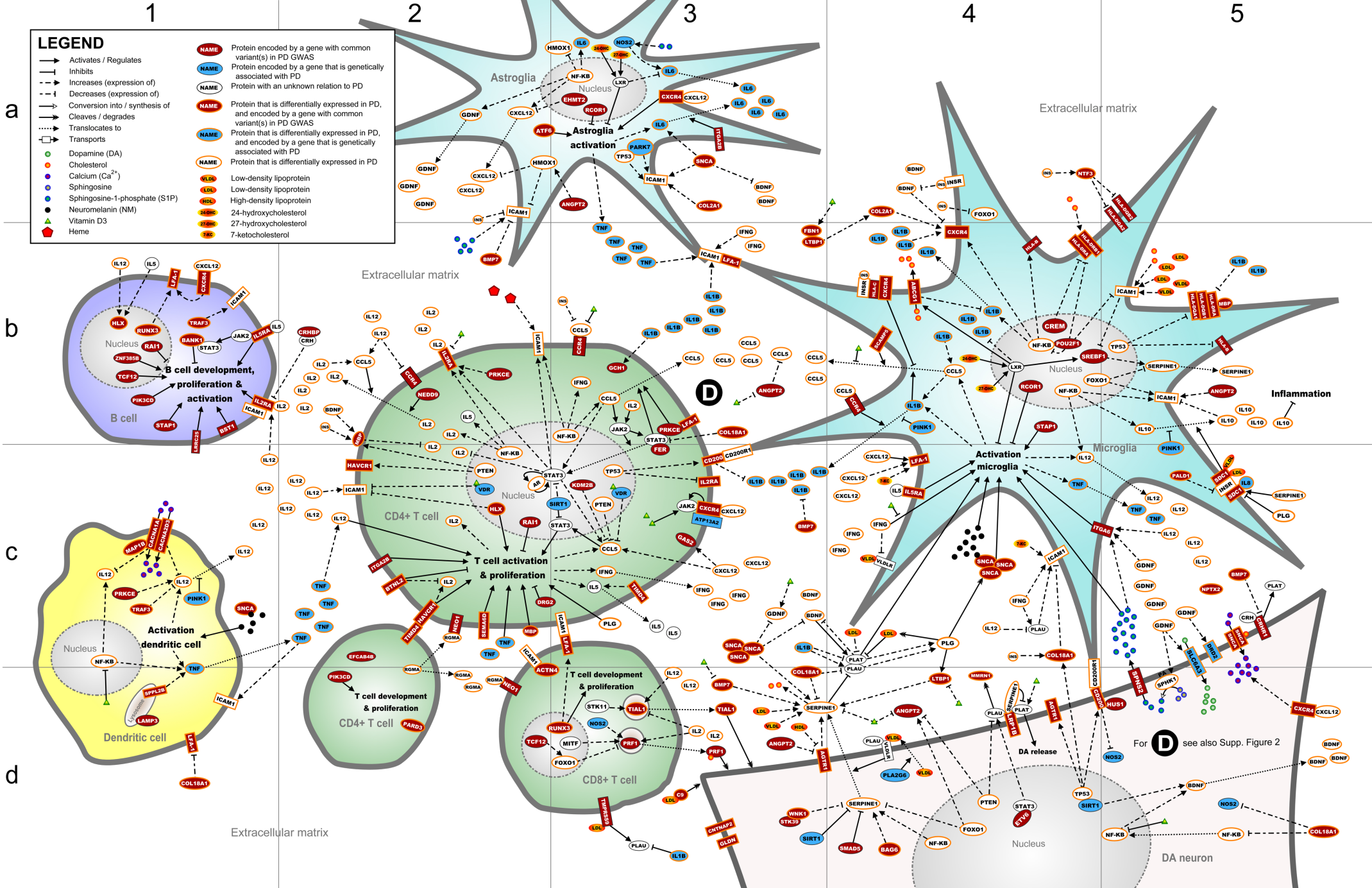
**

**Supplementary Figure 3: PD landscape; immune cell regulation-related pathways of process D.** The functional interactions within process D: ´neuron death and immune response´ that regulate the activation of astroglia, microglia, T cells, B cells and dendritic cells are shown. For each individual protein in this figure, **Supplementary Table 5** shows the location coordinates and the main process(es) (A-D) in which they exert their main effect.

**
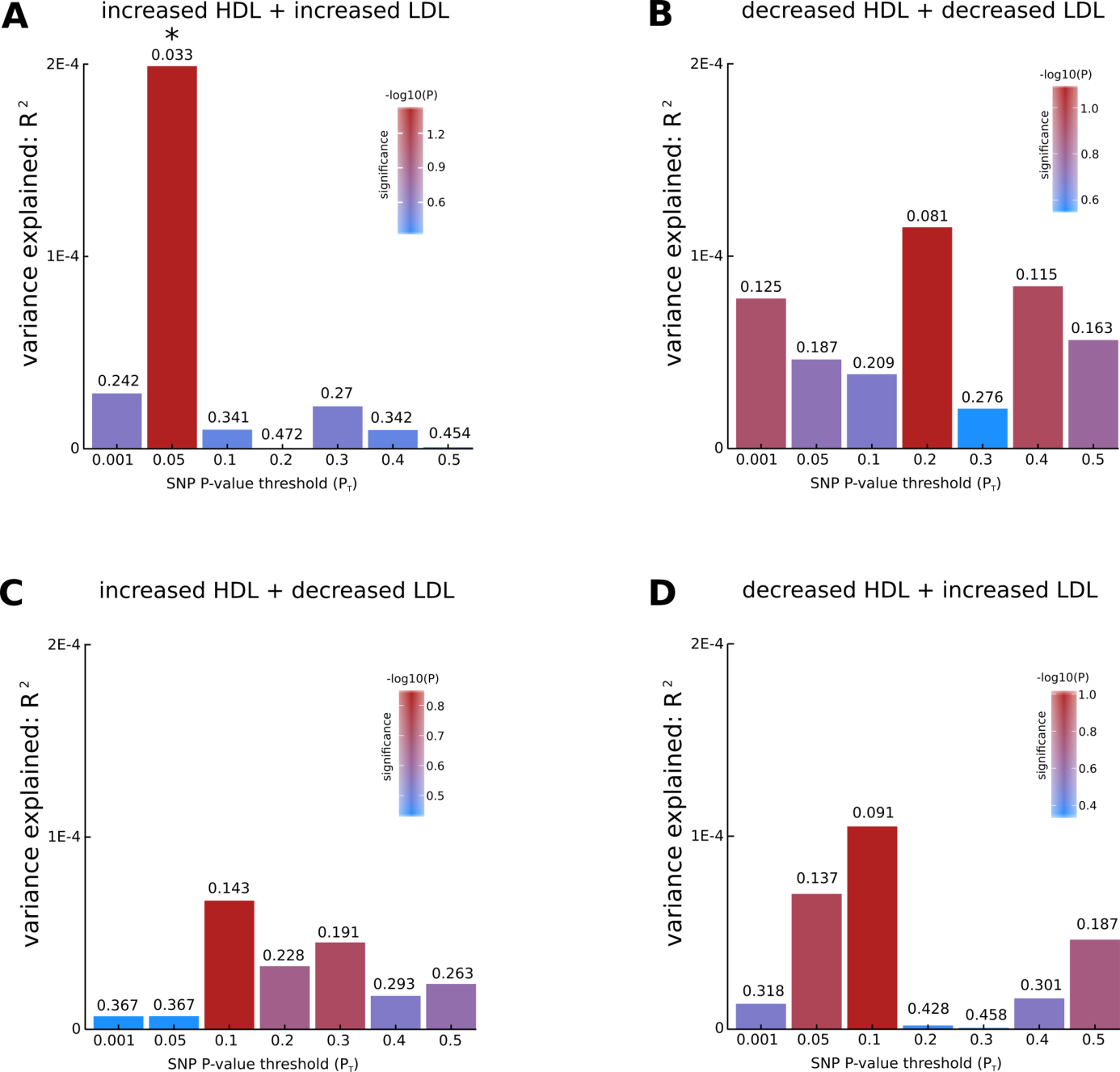
**

**Supplementary Figure 4.** **Additional Polygenic Risk Score Analyses.** Bar plots from PRSice showing results at broad P-value thresholds (P_T_) for shared genetic etiology between four 'combined lipoprotein traits' (increased HDL + increased LDL, decreased HDL + decreased LDL, increased HDL + decreased LDL, decreased HDL + increased LDL) and PD (see **Supplementary Methods**). The numbers above the bars indicate the P-values for shared genetic etiology, and these P-values were corrected using the false discovery rate (FDR) method; * denotes FDR-corrected P < 0.05

**LIST OF ABBREVIATIONS**

ΔΨm, mitochondrial transmembrane potential;

Ca^2+^, calcium ion;

CSF, cerebral spinal fluid;

DA, dopamine;

eQTL, expression quantitative trait locus;

ECM, extracellular matrix;

ER, endoplasmic reticulum;

FPD, familial Parkinson’s disease;

GWAS, genome-wide association study;

GWASs, genome-wide association studies;

INS, insulin;

IPD, idiopathic Parkinson’s disease;

K^+^, potassium ion;

LB, Lewy bodies;

LD, linkage disequilibrium;

Mg^2+^, magnesium ion;

mPTP, mitochondrial permeability transition pore;

Na^+^, sodium ion;

NM, neuromelanin;

nonsyn, nonsynonymous;

NS, not shown;

OR, odds ratio;

PD, Parkinson’s disease;

RAS, renin-angiotensin system;

SN, substantia nigra;

SNP(s), single nucleotide polymorphism(s);

syn, synonymous;

UTR, untranslated region.

| **Supplementary Table 1.** Overview of the fifteen published GWASs for PD. The thirteen GWASs used in our analysis are shown in bold (in total 13044 cases and 47148 controls). | | | | | | | |
| --- | --- | --- | --- | --- | --- | --- | --- |
| **GWAS** | **Discovery sample** | | **Population** | **Genotyping platform** | **Phenotype** | **Diagnosis** | **Notes** |
|  | **Patients** | **controls** |  |  |  |  |  |
| **Maraganore et al., 2005**^1^ | 443 | 443 | Primarily European origin | Perlegen genotyping platform | IPD | Standardized clinical assessment performed by a neurologist specialized in movement disorders | Sibling pairs |
| **Fung et al., 2006**^2^ | 267 | 270 | Caucasian | Illumina Infinium I  and Infinium HumanHap300 SNP chip | IPD (>55yrs) | N/A | Samples derived from NINDS Neurogenetics repository |
| **Pankratz et al., 2009**^3^ | 857 | 867 | Caucasian, non-hispanic | Illumina HumanCNV370 version1_C BeadChips | FPD | Neurological evaluation based on a modified version of the UK PD society Brain Bank Criteria | Known disease producing mutations were excluded [1]. |
| **Latourelle et al., 2009**^4^ [2] | 857 | N/A | Caucasian, non-hispanic | Illumina HumanCNV370 version1_C BeadChips | FPD | Neurological evaluation based on a modified version of the UK PD society Brain Bank Criteria | Known disease producing mutations were excluded [1]. |
| **Latourelle et al., 2009**^4^ [3] | 440 | N/A | Primarily European origin | Perlegen genotyping platform and Illumina HumanCNV370Duo array | IPD | Standardized clinical assessment performed by a neurologist specialized in movement disorders | - |
| Satake et al., 2009^5^ | 1078 | 2628 | Japanese | Illumina Infinium HumanHap550 array | IPD [4] | At least two cardinal PD symptoms without secondary cause,  levodopa responsive, and without other neurological problems. | Known disease producing mutations were excluded [5]. Not, included; not all data online available. |
| **Simon-Sanchez et al., 2009**^6^ | 1713 | 3978 | Caucasian; European ancestry | Illumina Infinium HumanHap550 array | IPD | Diagnosed according to the UK Brain Bank criteria | - |
| **Edwards et al., 2010**^7^ | 604 | 619 | Caucasian | Illumina Infinium 610-quad BeadChip / Illumina HumanHap 550  BeadChip | IPD | At least two cardinal PD symptoms without secondary cause and without other neurological problems. | - |
| **Hamza et al., 2010**^8^ | 2000 | 1986 | Ashkenazi Jewish and European origin | Illumina HumanOmni1-Quad_v1-0_B BeadChips | IPD  (435 FPD) | Diagnosed according to the UK Brain Bank criteria | - |
| **Spencer et al., 2011**^9^ | 1705 | 5175 | UK | Illumina Human660-Quad array / Illumina 1.2 M Duo array | IPD | Diagnosed according to the UK Brain Bank criteria | - |
| **Saad et al., 2011**^10^ | 1039 | 1984 | French | Illumina Human610-Quad BeadChip | FPD | At least two cardinal PD symptoms,  levodopa responsive, and without other neurological problems. | Known disease producing mutations were excluded [5]. |
| **Simon-Sanchez et al., 2011**^11^ | 772 | 2024 | Dutch | Illumina Human660W-Quad beadchips / Illumina Human610K beadchips | PD | N/A | - |
| **Do et al., 2011**^12^ | 3426 | 29624 | Primarily European ancestry | Illumina  HumanHap550+ BeadChip + approximately 25.000 custom SNPs | PD | Patients who stated they had been diagnosed with PD and who gave complete, non-suspicious answers to the other questions were included | Cases and controls were selected based on online questionnaires. |
| **Liu et al., 2011**^13^ | 268 | 178 | Ashkenazi Jewish | Illumina Human 610-quad bead arrays / Illumina Human  660-quad bead arrays | PD | Patients and controls were evaluated using the Unified Parkinson’s Disease Rating Scale (UPDRS) and the Mini Mental State Exam (MMSE). | Enriched for cases with an age of onset of 50 or younger. |
| Hernandez et al., 2012^14^ | 387 | 496 | Finish | Illumina Human660W v1 BeadChip / Illumina HumanCNV370 BeadChip | PD (<55yrs) | The diagnosis fulfilled international criteria for PD | Not, included; not all data online available. |

[1] All cases were negative for LRRK2 (G2019S) and most were screened for SNCA, PARK2 and PARK7 mutations.

[2] GWAS for age of PD onset. PD cases from Pankratz et al., 2009 were used.

[3] GWAS for age of PD onset. PD cases from Maragnore et al., 2005 were used.

[4] 39 cases had a relative with PD.

[5] Subjects diagnosed genetically with known PARK mutations (SNCA, LRRK2, PARK2 and PINK1) were excluded.

| **Supplementary Table 2.** PD candidate genes. Top single SNPs located in gene regions (including 100 kb of flanking downstream and/or upstream sequences) and with *P* < 1.00E-04 for association with Parkinson’s disease (PD) from the genome-wide association studies (GWASs) reported by Maraganore et al.^1^ (GWAS 1), Fung et al.^2^ (GWAS 2), Pankratz et al.^3^ (GWAS 3), Latourelle et al.^4^ (GWAS 4), Latourelle et al.^4^ (GWAS 5), Simon-Sanchez et al.^6^ (GWAS 6), Edwards et al.^7^ (GWAS 7), Hamza et al.^8^ (GWAS 8), Spencer et al.^9^ (GWAS 9), Saad et al.^10^ (GWAS 10), Simon-Sanchez et al.^11^ (GWAS 11), Do et al.^12^ (GWAS 12), and Liu et al.^13^ (GWAS 13). The genes encoding proteins that could be directly placed in the PD landscape (Figures 1, S2 and S3) are indicated in **bold**. If these genes or their protein products are linked to PD, either by genetic or expression and immunohistological studies (in PD patients), this is shown in the column ‘Corroborating evidence’. Single underlined genes are genetically associated with PD, dotted underlined genes encode proteins that are differentially expressed in PD patients and double underlined genes are both genetically associated with PD and encode a protein that is differentially expressed in PD. | | | | | | |
| --- | --- | --- | --- | --- | --- | --- |
| **GWAS 1** (Maraganore et al.) | | | | | | |
| **SNP** | ***P* value** | **Locus** | **Gene** | **Position ~ gene** [1] | **Corroborating evidence** | |
|  |  |  |  |  | **Genetic studies** | **Expression and immunohistological studies** [2] |
| rs3746736 | 1.30E-05 | 20p11.21 | *CSTL1* | non-syn coding | - | - |
| rs17463995 | 3.50E-05 | 15q21.1 | *CEP152* | 1.4 kb downstream | - | - |
| rs17463995 | 3.50E-05 | 15q21.1 | ***FBN1*** | 66 kb upstream | - | FBN1 mRNA is increased in blood^15^ and FBN1 protein is increased in CSF^16^ of PD patients compared to controls. |
| rs16887478 | 3.64E-05 | 8p11.22 | *C8orf86* | 56 kb upstream | - | - |
| rs11887431 | 3.66E-05 | 2p21 | *PKDCC* | 7.7 kb upstream | - | - |
| rs1984279 | 3.89E-05 | 20p11.21 | ***NAPB*** | 42 kb downstream | - | NAPB mRNA is decreased in the SN^17, 18^ and NAPB mRNA^19^ and protein^20^ is increased in the striatum of PD patients compared to controls. |
| rs1984279 | 3.89E-05 | 20p11.21 | *NXT1* | 18 kb upstream | - | - |
| rs10815285 | 4.64E-05 | 9p24.1 | *ERMP1* | intronic | - | - |
| rs960190 | 4.97E-05 | Xp22.13 | *GPR64* | 69 kb upstream | - | - |
| rs7694392 | 5.56E-05 | 4q24 | ***BANK1*** | intronic | - | BANK1 mRNA is increased in the striatum^20^ and BANK1 protein is decreased in the CSF^16^ of PD patients compared to controls. |
| rs7180500 | 6.35E-05 | 15q12 | *GABRG3* | intronic | - | GABRG3 mRNA is decreased in the SN of PD patients compared to controls^20^. |
| rs723268 | 7.15E-05 | 8q24.11 | *EXT1* | 43 kb upstream | - | - |
| rs723268 | 7.15E-05 | 8q24.11 | *SAMD12* | 35 kb downstream | - | - |
| rs6039424 | 8.04E-05 | 20p12.2 | ***PLCB4*** | intronic | - | - |
| rs7686646 | 8.38E-05 | 4q13.3 | ***RUFY3*** | intronic | - | RUFY3 mRNA is increased in the blood^15^ and in the SN (female patients)^21^ of PD patients or decreased in the SN^17, 22, 23^ of PD patients compared to controls. |
| rs4752662 | 8.94E-05 | 10q26.13 | ***TACC2*** | intronic | - | - |
| rs6802211 | 8.99E-05 | 3q22.3 | ***ARMC8*** | 34 kb downstream | - | ARMC8 protein is decreased in the CSF of PD patients compared to controls^16^. |
| rs6802211 | 8.99E-05 | 3q22.3 | *NME9* | 2.7 kb upstream | - | - |
| rs17719492 | 9.12E-05 | 4p16.3 | *ZNF141* | intronic | - | ZNF141 mRNA is increased in the blood of PD patients compared to controls^15^. |
| **GWAS 2** (Fung et al.) | | | | | | |
| **SNP** | ***P* value** | **Locus** | **Gene** | **Position ~ gene** [1] | **Corroborating evidence** | |
|  |  |  |  |  | **Genetic studies** | **Expression and immunohistological studies** [2] |
| rs2242330 | 1.70E-06 | 4q13.2 | ***STAP1*** | intronic | - | - |
| rs10501570 | 7.30E-06 | 11q14 | ***DLG2*** | intronic | - | DLG2 mRNA is decreased in the blood^24^, DLG2 protein is decreased in the SN^25^ and increased in the striatum^20^ of PD patients compared to controls. |
| rs281357 | 9.80E-06 | 17p11.2 | ***ULK2*** | intronic | - | **-** |
| rs988421 | 4.90E-05 | 1p13 | ***NEGR1*** | intronic | - | NEGR1 protein is decreased in the SN^26^ and CSF^16^ and increased in the striatum^20^ of PD patients compared to controls. |
| rs1912373 | 5.60E-05 | 11q11 | *OR9G1* | 15 kb downstream | - | - |
| rs1912373 | 5.60E-05 | 11q11 | *OR5AP2* | 74 kb upstream | - | - |
| rs1887279 | 5.70E-05 | 1q25 | *COLGALT2* | intronic | - | COLGALT2 mRNA is increased in the SN of PD patients compared to control^18^. |
| rs6125829 | 6.60E-05 | 20q13.13 | *RNF114* | 3' UTR | - | RNF114 mRNA is decreased in the SN of PD patients compared to controls^23^. |
| rs355477 | 7.90E-05 | 4q13.2 | ***CENPC*** | intronic | - |  |
| **GWAS 3** (Pankratz et al.) | | | | | | |
| **SNP** | ***P* value** | **Locus** | **Gene** | **Position ~ gene** [1] | **Corroborating evidence** | |
|  |  |  |  |  | **Genetic studies** | **Expression and immunohistological studies** [2] |
| rs11248060 | 3.40E-06 | 4p16.3 | ***DGKQ*** | intronic | Genome-wide significance was reached for the intronic SNP rs11248060 in DGKQ (OR=1.21; P=3.04E-12)^27^ (OR=1.35; P=2.0E-9)^28^, a SNP that also increases the risk for PD in a Chinese population (P<0.05)^29^. | **-** |
| rs11248051 | 5.20E-06 | 4p16.3 | ***GAK*** | intronic | SNPs in GAK rs1564282 (Caucasian; OR=1.61; P=0.0151)^30^, rs1564282 (Chinese)^29^, rs11248051 (Taiwanese; CT/TT vs CC genotypes OR=1.37; P=0.03)^31^, rs11248051 (meta-analysis; OR=1.35; P=8.2E-09)^28^ are associated with an increased PD risk. Based on motor Unified Parkinson's Disease Rating Scale subscores GAK (rs1564282) is associated with tremor in PD (P=0.03)^32^. | **-** |
| rs1997791 | 1.90E-05 | 20q13.13 | ***PTPN1*** | 30 kb upstream | **-** | **-** |
| rs1724425 | 2.00E-05 | 17q21.31 | ***CRHR1*** | 79 kb upstream | **-** | **-** |
| rs4240910 | 3.90E-05 | 1p36.22 | ***PIK3CD*** | intronic | **-** | **-** |
| rs10094981 | 4.80E-05 | 8p11.21 | *ZMAT4* | 76 kb upstream | **-** | ZMAT4 mRNA is increased in the SN of PD patients compared to controls^20^. |
| rs898528 | 4.90E-05 | 17q25.3 | *RBFOX3* | intronic | **-** | **-** |
| rs12871648 | 5.00E-05 | 13q34 | ***LAMP1*** | intronic | - | LAMP1 mRNA is decreased in the SN of female PD patients compared to controls^23^. LAMP1 expression is decreased in SN neurons of PD patients^33, 34^ and even further decreased when these neurons contained SNCA inclusions^33^. |
| rs4670322 | 5.10E-05 | 2p22.3 | ***LTBP1*** | intronic | **-** | LTBP1 mRNA is increased in the blood of PD patients compared to controls^15^. |
| rs10937194 | 5.90E-05 | 3q27.2 | *VPS8* | intronic | **-** | **-** |
| rs3775478 | 6.10E-05 | 4q22.1 | ***MMRN1*** | intronic | **-** | MMRN1 mRNA is increased in the striatum of PD patients compared to controls^20^. |
| rs1519686 | 7.10E-05 | 6q21 | *HS3ST5* | intronic | **-** | **-** |
| rs4890430 | 7.10E-05 | 18q12.3 | ***RIT2*** | 1.9 kb upstream | In a meta-analysis of GWASs RIT2 was identified as a novel susceptibility locus (rs12456492, OR=1.19, P=2E-10 (combined sample))^28^. | RIT2 mRNA is decreased in the SN of PD patients c-ompared to controls^17^. |
| rs4901519 | 7.60E-05 | 14q22.2 | *CGRRF1* | 13 kb downstream | **-** | **-** |
| rs2083482 | 7.60E-05 | 2q24.3 | *FIGN* | 12 kb downstream | **-** | **-** |
| rs4901519 | 7.60E-05 | 14q22.2 | ***SAMD4A*** | 15 kb upstream | **-** | SAMD4A mRNA is increased in the blood of PD patients compared to controls^15, 24^. |
| rs1159220 | 8.20E-05 | 22q12.3 | ***SYN3*** | intronic | **-** | **-** |
| rs12638253 | 8.30E-05 | 3q25.31 | *LEKR1* | intronic | **-** | **-** |
| rs1504489 | 8.40E-05 | 4q22.1 | *GPRIN3* | 29 kb upstream | **-** | **-** |
| rs356188 | 8.40E-05 | 4q22.1 | ***SNCA*** | intronic | The mutation G209A (Ala53Thr) in SNCA was found in one Italian and three Greek families with PD, but not in controls^35^ and the G88C (Ala30Pro) mutation was found in a German family^36^. SNCA gene duplication^37, 38^ and triplication^39^ causes familial PD. Based on motor Unified PD Rating Scale subscores SNCA (rs356220) is associated with rigidity in PD (P=0.04)^32^. | SNCA mRNA is decreased in the SN of PD patients compared to controls^17, 22, 23^. LB immunoreactive for SNCA were found in the SN of PD patients^40, 41^. |
| rs10859725 | 8.50E-05 | 12q22 | *CEP83* | 90 kb upstream | **-** | **-** |
| rs10859725 | 8.50E-05 | 12q22 | ***TMCC3*** | 17 kb downstream | **-** | **-** |
| rs11012 | 8.80E-05 | 17q21.31 | ***PLEKHM1*** | 3' UTR | **-** | PLEKHM1 mRNA is decreased in the SN of female PD patients compared to controls^23^ and is increased in the blood of PD patients compared to controls^15^. |
| rs9655034 | 8.80E-05 | 7p14.1 | *POU6F2* | intronic | **-** | **-** |
| rs1197313 | 8.90E-05 | 3q22.1 | *TMEM108* | intronic | **-** | **-** |
| rs6912010 | 9.20E-05 | 6q21 | ***CDK19*** | 35 kb downstream | **-** | CDK19 mRNA is increased in the SN^21^ and blood^15^ of (female) PD patients compared to controls. |
| rs6912010 | 9.20E-05 | 6q21 | *SLC22A16* | 99 kb upstream | **-** | **-** |
| rs7312607 | 9.30E-05 | 12q23.1 | *CDK17* | 32 kb upstream | **-** | - |
| rs1355095 | 9.40E-05 | 5q31.1 | ***ACSL6*** | intronic | **-** | ACSL6 mRNA is decreased in the SN of PD patients compared to controls^18^. |
| rs9859577 | 9.90E-05 | 3q28 | ***FGF12*** | intronic | **-** | FGF12 mRNA is decreased in the SN of PD patients compared to controls^17, 18^. |
| **GWAS 4** (Latourelle et al.) | | | | | | |
| **SNP** | ***P* value** | **Locus** | **Gene** | **Position ~ gene** [1] | **Corroborating evidence** | |
|  |  |  |  |  | **Genetic studies** | **Expression and immunohistological studies** [2] |
| rs10952735 | 6.90E-08 | 7q36.1 | ***CNTNAP2*** | intronic | - | CNTNAP2 mRNA is decreased in the SN^23^ and increased^15^ or decreased^24^ in the blood of PD patients compared to controls. |
| rs12261736 | 1.80E-07 | 10q21.1 | ***PRKG1*** | intronic | - | - |
| rs7954006 | 7.10E-07 | 12q24.31 | ***HCAR1*** | intronic | - | - |
| rs11974194 | 1.20E-06 | 7q36.3 | *LMBR1* | intronic | - | LMBR1 mRNA is decreased in the SN of PD patients compared to controls^18^. |
| rs17565841 | 2.70E-06 | 15q12 | ***OCA2*** | 2.8 kb downstream | - | OCA2 mRNA is increased in the striatum of PD patients compared to controls^20^. |
| rs9904572 | 4.30E-06 | 17p12 | ***ARHGAP44*** | intronic | - | ARHGAP44 mRNA is decreased in the SN of PD patients compared to controls^18^. |
| rs12504099 | 7.70E-06 | 4p14 | *TBC1D1* | intronic | - | - |
| rs7076519 | 9.30E-06 | 10q26.2 | ***ADAM12*** | intronic | - | - |
| rs10767971 | 9.30E-06 | 11p13 | *QSER1* | 19 kb upstream | - | - |
| rs7828992 | 1.00E-05 | 8q24.3 | ***TRAPPC9*** | intronic | - | - |
| rs1843604 | 1.20E-05 | 3q22.1 | ***CPNE4*** | 15 kb downstream | - | - |
| rs1843604 | 1.20E-05 | 3q22.1 | ***MRPL3*** | 15 kb upstream | - | MRPL3 mRNA is decreased in the SN of PD patients compared to controls^22, 23^. |
| rs4791571 | 1.30E-05 | 17p12 | *HS3ST3B1* | intronic | - | - |
| rs12146113 | 1.30E-05 | 1q32.1 | ***KIF14*** | 78 kb downstream | - | - |
| rs6069640 | 1.30E-05 | 20q13.2 | *MC3R* | 50 kb upstream | - | - |
| rs12146113 | 1.30E-05 | 1q32.1 | *ZNF281* | 64 kb upstream | - | - |
| rs11062784 | 1.70E-05 | 12p13.32 | ***EFCAB4B*** | intronic | - | - |
| rs2826833 | 1.80E-05 | 21q21.1 | ***NCAM2*** | intronic | - | - |
| rs7556447 | 2.10E-05 | 1p36.32 | *RER1* | 11 kb downstream | - | RER1 mRNA is decreased in the blood of PD patients compared to controls^15^. |
| rs7556447 | 2.10E-05 | 1p36.32 | *PEX10* | 863 bp upstream | - |  |
| rs10918270 | 2.90E-05 | 1q23.3 | ***ATF6*** | intronic | - | ATF6 mRNA is increased in the blood of PD patients compared to controls^15^. |
| rs12328510 | 3.10E-05 | 2p21 | ***PRKCE*** | intronic | - | - |
| rs956322 | 3.10E-05 | 11p13 | *PRRG4* | intronic | - | PRRG4 mRNA is increased in the SN of PD patients compared to controls^18^. |
| rs4827256 | 3.50E-05 | Xp11.4 | ***DDX3X*** | 99 kb upstream | - | DDX3X is increased in the striatum of PD patients compared to controls^42^. |
| rs4827256 | 3.50E-05 | Xp11.4 | ***USP9X*** | 1.2 kb downstream | - | USP9X protein localizes to LB and is lower expressed in the SN of PD patients^43^. |
| rs11655490 | 3.80E-05 | 17q25.1 | *FAM104A* | 90 kb downstream | - | - |
| rs11655490 | 3.80E-05 | 17q25.1 | *SLC39A11* | 25 kb upstream | - | - |
| rs17817190 | 3.90E-05 | 2q21.2 | *NCKAP5* | 26 kb upstream | - | - |
| rs1572662 | 4.10E-05 | 6q25.2 | *CNKSR3* | intronic | - | CNKSR3 mRNA is increased in the SN of PD patients compared to controls^18^. |
| rs11127593 | 5.10E-05 | 3p12.3 | ***ROBO2*** | intronic | - | ROBO2 mRNA is decreased in the SN^17, 18, 44^ and ROBO2 protein is decreased in the CSF^16^ of PD patients compared to controls. |
| rs10420134 | 5.80E-05 | 19q13.12 | *C19orf55* | 1.9 kb downstream | - | - |
| rs10420134 | 5.80E-05 | 19q13.12 | ***ARHGAP33*** | 4.5 kb upstream | - | ARHGAP33 mRNA is decreased (in female)^23^ or increased^18^ in the SN of PD patients compared to controls. |
| rs2667648 | 7.00E-05 | 16q23.1 | ***WWOX*** | intronic | - | - |
| rs9916114 | 7.30E-05 | 17q11.2 | ***PSMD11*** | 4.6 kb upstream |  | Proteasomal function is impaired in the PD SN^45^. |
| rs9916114 | 7.30E-05 | 17q11.2 | *ZNF207* | 52 kb downstream | - | - |
| rs11899121 | 8.00E-05 | 2p24.1 | ***SDC1*** | 33 kb downstream | - | SDC1 mRNA is decreased in the SN of (female) PD patients compared to controls^18, 23^. |
| rs4764854 | 8.20E-05 | 12q23.2 | ***DRAM1*** | intronic | - | - |
| rs1234326 | 8.70E-05 | 10q22.1 | ***CDH23*** | intronic | - | - |
| **GWAS 5** (Latourelle et al.) | | | | | | |
| **SNP** | ***P* value** | **Locus** | **Gene** | **Position ~ gene** [1] | **Corroborating evidence** | |
|  |  |  |  |  | **Genetic studies** | **Expression and immunohistological studies** [2] |
| rs887458 | 1.90E-06 | 7p14.1 | ***AMPH*** | 61 kb downstream | - | AMPH mRNA is decreased in the SN of PD patients compared to controls^17, 46, 47^. AMPH protein is increased in the striatum of PD patients compared to controls^20^. |
| rs1467751 | 1.30E-05 | 21q22.2 | ***DSCAM*** | intronic | - | - |
| rs1467751 | 1.30E-05 | 21q22.2 | ***TMPRSS3*** | intronic | - | - |
| rs6440565 | 1.50E-05 | 3q24 | ***AGTR1*** | 57 kb upstream | - | AGTR1 mRNA is decreased in the SN of (male) PD patients compared to controls^17, 18, 20, 21^. |
| rs2550401 | 2.00E-05 | 16p13.3 | *ZNF200* | 6 kb downstream | - | - |
| rs2550401 | 2.00E-05 | 16p13.3 | *ZNF263* | 48 kb upstream | - | - |
| rs1355637 | 4.10E-05 | 1q21.3 | ***ENSA*** | intronic | - | - |
| **GWAS 6** (Simon-Sanchez et al.) | | | | | | |
| **SNP** | ***P* value** | **Locus** | **Gene** | **Position ~ gene** [1] | **Corroborating evidence** | |
|  |  |  |  |  | **Genetic studies** | **Expression and immunohistological studies** [2] |
| rs2736990 | **5.69E-09** | 4q22.1 | ***SNCA*** | intronic | The mutation G209A (Ala53Thr) in SNCA was found in one Italian and three Greek families with PD, but not in controls^35^ and the G88C (Ala30Pro) mutation was found in a German family^36^. SNCA gene duplication^37, 38^ and triplication^39^ causes familial PD. Based on motor Unified PD Rating Scale subscores SNCA (rs356220) is associated with rigidity in PD (P=0.04)^32^. | SNCA mRNA is decreased in the SN of PD patients compared to controls^17, 22, 23^. LB immunoreactive for SNCA were found in the SN of PD patients^40, 41^. |
| rs415430 | **4.50E-08** | 17q21.31 | ***WNT3*** | intronic | - | WNT3 mRNA is decreased in the SN of PD patients compared to controls^18^. |
| rs199533 | 5.05E-08 | 17q21.31 | ***NSF*** | intronic | - | NSF mRNA is decreased in the SN^17, 18, 22, 23, 46-48^ and striatum^46^ of PD patients compared to controls. |
| rs393152 | 1.42E-07 | 17q21.31 | ***CRHR1*** | intronic | - | - |
| rs12185268 | 1.90E-07 | 17q21.31 | *SPPL2C* | non-syn coding | - | - |
| rs1981997 | 2.02E-07 | 17q21.31 | ***MAPT*** | intronic | Genome-wide significant association (P<5E-08) of MAPT with PD was found in multiple meta-analyses (H1/H2 haplotype; OR=0.78, P=7.97E-52^27^, rs242559; OR=0.78, P=1.5E-10)^28^. | MAPT mRNA is decreased in the SN^21-23^ and MAPT protein is decreased in the CSF^49^ of PD patients compared to controls. |
| rs2532274 | 2.22E-07 | 17q21.31 | ***KANSL1*** | intronic | - | - |
| rs11648673 | 4.77E-07 | 16p13.3 | ***AXIN1*** | intronic | - | AXIN1 mRNA is increased in the SN of PD patients compared to controls^18^. |
| rs239748 | 1.17E-06 | Xp22.13 | *PHKA2* | 27 kb downstream | - | - |
| rs7013027 | 1.85E-06 | 8p23.2 | ***CSMD1*** | intronic | - | CSMD1 mRNA is decreased in the SN of PD patients compared to controls^18^. |
| rs11012 | 2.85E-06 | 17q21.31 | ***PLEKHM1*** | 3' UTR | - | PLEKHM1 mRNA is decreased in the SN of female PD patients compared to controls^23^ and is increased in the blood of PD patients compared to controls^15^. |
| rs10857899 | 3.06E-06 | 1p13.2 | ***RAP1A*** | intronic | - | RAP1A protein is increased in the SN of PD patients compared to controls^26^. |
| rs6542651 | 3.34E-06 | 2p25.3 | *DCDC2C* | intronic | - | - |
| rs2285459 | 3.38E-06 | 16p11.2 | ***ITGAL*** | intronic | - | (Part of LFA-1 complex). The number of LFA-1 (ITGAL) positive microglia in the SN and striatum increased during degeneration of the SN^50^. |
| rs2492448 | 3.84E-06 | 10p11.21 | ***PARD3*** | 91 kb upstream |  | PARD3 mRNA is increased in BA9 in PD patients compared to controls^51^. |
| rs4957473 | 4.24E-06 | 5p13.1 | ***C9*** | intronic | - | C9 protein is decreased in the CSF of PD patients compared to controls^16^. Intra- and extraneuronal LB and dendritic spheroid bodies were immunoreactive for C9 in PD SN, but not in controls^52^. |
| rs2896905 | 5.03E-06 | 12q12 | ***SLC2A13*** | intronic | An interaction between the SNP rs2896905 in SLC2A13 and caffeine intake or smoking and caffeine intake combined is associated with PD risk i.e., high caffeine intake reduces PD risk (OR=0.81, P=0.049), never smokers with low caffeine intake have a higher PD risk (OR=1.35, P=0.04) and smokers with high caffeine intake have a lower PD risk (OR=0.68, P=0.007)^53^. | SLC2A13 mRNA is decreased in the SN of PD patients compared to controls^18^. |
| rs817097 | 6.22E-06 | 17q24.3 | ***MAP2K6*** | 33 kb upstream | - | - |
| rs2856336 | 7.69E-06 | 12p13.2 | ***ETV6*** | intronic | - | - |
| rs764660 | 7.83E-06 | 2q24.3 | ***SCN2A*** | intronic | - | SCN2A mRNA is decreased in the SN^17, 18^ and increased in the striatum^19^ of PD patients compared to controls. |
| rs11244079 | 8.66E-06 | 9q34.2 | ***RPL7A*** | 31 kb upstream | - | RPL7A mRNA is decreased in male PD patients compared to controls^23^. |
| rs11244079 | 8.66E-06 | 9q34.2 | *SURF6* | 13 kb downstream | - | - |
| rs2733333 | 9.31E-06 | 15q21.3 | ***TCF12*** | intronic | - | - |
| rs11878803 | 1.07E-05 | 19q13.41 | *ZNF615* | intronic | - | - |
| rs17654531 | 1.16E-05 | 1p13.2 | *CHI3L2* | 14 kb downstream | - | CHI3L2 is increased in the striatum of PD patients compared to controls^20^. |
| rs7923172 | 1.43E-05 | 10p11.21 | ***CUL2*** | intronic | - | - |
| rs163321 | 1.49E-05 | 5q35.3 | ***ADAMTS2*** | intronic | - | ADAMTS2 mRNA is increased in the SN^23^ and blood^24^ of (female) PD patients compared to controls. |
| rs869714 | 1.54E-05 | 1q24.1 | *DUPD1* | 28 kb downstream | - | - |
| rs869714 | 1.54E-05 | 1q24.1 | ***POU2F1*** | 64 kb upstream | - | - |
| rs10827492 | 1.69E-05 | 10p11.21 | ***CREM*** | intronic | - | - |
| rs13139027 | 1.75E-05 | 4p16.2 | ***MSX1*** | 61 kb downstream | - | MSX1 mRNA is increased in the SN of PD patients compared to controls^18^. |
| rs2491015 | 1.76E-05 | 10q22.1 | *KIAA1279* | intronic | - | - |
| rs3792738 | 2.19E-05 | 5q13.3 | ***CRHBP*** | 754 b upstream | - | - |
| rs3792738 | 2.19E-05 | 5q13.3 | *S100Z* | 30 kb downstream | - | S100Z mRNA is increased in the striatum of PD patients compared to controls^20^. |
| rs7651825 | 2.20E-05 | 3p25.1 | *C3orf20* | intronic | - | - |
| rs17115100 | 2.46E-05 | 10q24.32 | ***CYP17A1*** | intronic | - | - |
| rs558076 | 2.53E-05 | 17q24.3 | ***ABCA5*** | 12 kb upstream | - | - |
| rs4247113 | 2.62E-05 | 17p13.3 | *FAM101B* | 61 kb downstream | - | FAM101B mRNA is increased in the SN of PD patients compared to controls^18^. |
| rs4247113 | 2.62E-05 | 17p13.3 | ***RPH3AL*** | 26 kb upstream | - | - |
| rs1793949 | 3.14E-05 | 12q13.11 | ***COL2A1*** | intronic | - | COL2A1 mRNA is decreased in the SN^20^ and COL2A1 protein is increased in the CSF^16^ of PD patients compared to controls. |
| rs859522 | 3.41E-05 | 7p14.1 | ***VPS41*** | intronic | - | - |
| rs2708909 | 3.44E-05 | 7p12.3 | *SUN3* | intronic | - | - |
| rs1395993 | 3.49E-05 | 3p24.3 | ***ZNF385B*** | 90 kb downstream | - | - |
| rs10849446 | 3.66E-05 | 12p13.31 | ***SCNN1A*** | intronic | - | - |
| rs3824754 | 3.92E-05 | 10q24.32 | *C10orf32* | intronic | - | - |
| rs12261843 | 4.05E-05 | 10p11.21 | *CCNY* | intronic | - | CCNY mRNA is increased in the SN of male PD patients compared to controls^21^. |
| rs4563067 | 4.11E-05 | 17q25.1 | ***RPL38*** | 11 kb upstream | - | RPL38 mRNA is decreased in the SN of PD patients compared to controls^23^. |
| rs6794137 | 4.27E-05 | 3q22.1 | *TMEM108* | intronic | - | - |
| rs9458499 | 4.27E-05 | 6q26 | ***PARK2*** | intronic | Exon deletions, duplications and triplications in the PARK2 gene cause autosomal recessive juvenile parkinsonism^54-59^. Multiple mutations in the PARK2 gene are associated with early-onset autosomal recessive PD^60-63^. The GG genotype of the -258 T/G PARK2 polymorphism is associated with a lower age of onset of PD compared with the common TT genotype^64^. | A PARK2 splice variant (resulting in exon 4 deletion and a truncated protein) is increased in PD SN and lymphocytes and the PARK2 splice variant/wild type ratio increases with age in PD patients^65^. |
| rs6044224 | 4.37E-05 | 20p12.1 | ***SNRPB2*** | 52 kb upstream | - | - |
| rs11778693 | 4.45E-05 | 8p21.3 | ***CCAR2*** | 5' UTR | - | - |
| rs7707022 | 4.58E-05 | 5p13.2 | *WDR70* | 13 kb downstream | - | - |
| rs11183395 | 4.59E-05 | 12q13.11 | *SLC38A1* | intronic | - | SLC38A1 mRNA is increased in the striatum of PD patients compared to controls^19, 20^. |
| rs935378 | 4.63E-05 | 2p21 | ***MCFD2*** | 20 kb downstream | - | MCFD2 mRNA is decreased in the SN of PD patients compared to controls^23^. |
| rs1005511 | 4.68E-05 | 11q12.1 | ***SERPING1*** | intronic | - | SERPING1 protein is decreased in the blood of PD patients compared to controls^66^. |
| rs12413409 | 4.69E-05 | 10q24.32 | ***CNNM2*** | intronic | - | - |
| rs17071181 | 4.86E-05 | 18q21.33 | ***SERPINB5*** | intronic | - | - |
| rs2227928 | 5.11E-05 | 7p13.2 | ***ATR*** | non-syn coding | - | ATR mRNA is decreased in the SN of PD patients compared to controls^23^. |
| rs10246477 | 5.25E-05 | 7q21.11 | ***SEMA3E*** | intronic | - | SEMA3E mRNA is decreased in the SN of PD patients compared to controls^18^. |
| rs10894203 | 5.46E-05 | 11q24.3 | *ADAMTS15* | intronic | - | - |
| rs9525776 | 5.53E-05 | 13q14.11 | *ENOX1* | intronic | - | - |
| rs7454430 | 5.54E-05 | 6q22.31 | ***CLVS2*** | 27 kb upstream | - | CLVS2 mRNA is decreased in the SN of PD patients compared to controls^18^. |
| rs9924026 | 5.94E-05 | 16q12.1 | ***ZNF423*** | intronic | - | - |
| rs1934828 | 6.08E-05 | 13q22.2 | *LMO7DN* | 75 kb downstream | - | - |
| rs6599389 | 6.28E-05 | 4p16.3 | *TMEM175* | intronic | - | - |
| rs265120 | 6.38E-05 | 1q41 | *GPATCH2* | 7 kb downstream | - | - |
| rs3740484 | 6.57E-05 | 10p24.31 | ***PEO1*** | intronic | The mutations c.G1121A (Arg374Gln) and c.G1750A (Ala359Thr) in PEO1 are associated with familial parkinsonism and ophthalmoplegia and segregate with the disease phenotype^67, 68^. | - |
| rs636508 | 6.65E-05 | 9q21.32 | ***TLE1*** | 88 kb upstream | - | - |
| rs6812193 | 6.67E-05 | 4q21.1 | FAM47E | intronic | - | - |
| rs6812193 | 6.67E-05 | 4q21.1 | *STBD1* | intronic | - | - |
| rs6959225 | 6.84E-05 | 7p21.3 | *NXPH1* | intronic | - | - |
| rs8111509 | 6.84E-05 | 19q13.41 | ***FPR3*** | intronic | - | FPR3 mRNA is increased in the SN^18^ and striatum^20^ of PD patients compared to controls. |
| rs7077361 | 6.88E-05 | 10p13 | ***ITGA8*** | intronic | A meta-analysis on GWAS data showed genowe-wide association of a SNP (rs7077361) in ITGA8 with PD (OR=0.88, P=1.3E-08)^27^. | ITGA8 mRNA is decreased in the SN and increased in the striatum of PD patients compared to controls^20^. |
| rs9839984 | 6.92E-05 | 3q26.1 | ***PPM1L*** | intronic | - | - |
| rs2240914 | 6.98E-05 | 9q34.11 | *GPR107* | 3' UTR | - | - |
| rs4661747 | 7.02E-05 | 1p36.13 | *SPATA21* | intronic | - | - |
| rs4584384 | 7.09E-05 | 1q21.3 | *TDRD10* | intronic | - | - |
| rs595046 | 7.27E-05 | 21q22.3 | *C21orf125* | 64 kb upstream | - | - |
| rs595046 | 7.27E-05 | 21q22.3 | ***SIK1*** | 32 kb downstream | - | SIK1 mRNA is decreased in the SN of PD patients compared to controls^18^. |
| rs2686831 | 7.31E-05 | 7p12.3 | ***HUS1*** | intronic | - | - |
| rs4242434 | 7.31E-05 | 8p21.3 | *BIN3* | intronic | - | BIN3 mRNA is increased in the SN of PD patients compared to controls^18^. |
| rs6582668 | 7.41E-05 | 12q12 | *ALG10B* | 43 kb downstream | - | - |
| rs6780193 | 7.93E-05 | 3p13 | ***PROK2*** | 72 kb upstream | - | PROK2 mRNA is increased in the striatum of PD patients compared to controls^20^. |
| rs16944593 | 7.95E-05 | 12q24.21 | ***TBX3*** | 35 kb upstream | - | TBX3 mRNA is increased in the SN of PD patients compared to controls^18^. |
| rs7436941 | 8.13E-05 | 4q32.1 | ***RXFP1*** | intronic | - | RXFP1 mRNA is increased in the striatum of PD patients compared to controls^20^. |
| rs7911697 | 8.19E-05 | 10p13 | ***FRMD4A*** | intronic | - | - |
| rs7903802 | 8.38E-05 | 10p13 | *CCDC3* | intronic | - | - |
| rs699038 | 8.53E-05 | 12p12.1 | *C12orf77* | 9 kb upstream | - | - |
| rs2515501 | 8.85E-05 | 8p23.1 | ***ANGPT2*** | intronic | - | - |
| rs2515501 | 8.85E-05 | 8p23.1 | ***MCPH1*** | intronic | - | - |
| rs9480154 | 9.25E-05 | 6q25.1 | *IYD* | 79 kb upstream | - | - |
| rs9480154 | 9.25E-05 | 6q25.1 | ***PPP1R14C*** | 39 kb downstream | - (Part of the PP1-complex). | - |
| rs2470179 | 9.27E-05 | 15q21.2 | ***GLDN*** | intronic | - | GLDN mRNA is decreased in the striatum^20^ and increased in the blood^24^ and GLDN protein is increased in the CSF^16^ of PD patients compared to controls. |
| rs12777747 | 9.30E-05 | 10q26.13 | ***TACC2*** | intronic | - | - |
| rs1224671 | 9.32E-05 | 15q21.1 | ***SEMA6D*** | intronic | - | SEMA6D mRNA is decreased in the SN^18^ and SEMA6D protein is decreased in CSF^16^ of PD patients compared to controls. |
| rs2708851 | 9.33E-05 | 7p12.3 | *C7orf57* | intronic | - | C7orf57 mRNA is increased in the striatum of PD patients compared to controls^20^. |
| rs8014371 | 9.34E-05 | 14q13.1 | *NPAS3* | intronic | - | - |
| rs1605527 | 9.41E-05 | 3p24.3 | *ZNF385D* | 83 kb downstream | - | ZNF385D mRNA is decreased in the SN of PD patients compared to controls^18^. |
| rs6440096 | 9.52E-05 | 3q23 | ***PLS1*** | intronic | - | - |
| rs748088 | 9.66E-05 | 21q22.13 | *DSCR4* | intronic | - | - |
| rs6596287 | 9.72E-05 | 5q31.1 | ***SMAD5*** | intronic | - | - |
| **GWAS 7** (Edwards et al.) | | | | | | |
| **SNP** | ***P* value** | **Locus** | **Gene** | **Position ~ gene** [1] | **Corroborating evidence** | |
|  |  |  |  |  | **Genetic studies** | **Expression and immunohistological studies** [2] |
| rs356220 | 2.67E−06 | 4q22.1 | ***SNCA*** | 5 kb downstream | The mutation G209A (Ala53Thr) in SNCA was found in one Italian and three Greek families with PD, but not in controls^35^ and the G88C (Ala30Pro) mutation was found in a German family^36^. SNCA gene duplication^37, 38^ and triplication^39^ causes familial PD. Based on motor Unified PD Rating Scale subscores SNCA (rs356220) is associated with rigidity in PD (P=0.04)^32^. | SNCA mRNA is decreased in the SN of PD patients compared to controls^17, 22, 23^. LB immunoreactive for SNCA were found in the SN of PD patients^40, 41^. |
| rs1543467 | 2.97E−06 | 1p22.3 | *CLCA4* | intronic | - | - |
| rs12063142 | 5.02E-06 | 1p36.13 | *TAS1R2* | 27 kb downstream | - | - |
| rs9513249 | 5.92E-06 | 13q32.1 | ***RAP2A*** | 39 kb downstream | - | - |
| rs976683 | 1.04E-05 | 3q26.31 | ***NLGN1*** | intronic | - | NLGN1 mRNA is decreased in the SN of PD patients compared to controls^18^. |
| rs1816879 | 1.16E-05 | 15q21.3 | ***LIPC*** | 92 kb upstream | - | - |
| rs1406968 | 1.16E-05 | 20p11.23 | ***SLC24A3*** | intronic | - | SLC24A3 mRNA is decreased in the SN of (female) PD patients compared to controls^18, 23^. |
| rs11625012 | 1.61E-05 | 14q22.1 | ***TMX1*** | 80 kb downstream | - | - |
| rs135066 | 1.91E-05 | 22q13.2 | *MPPED1* | intronic | - | MPPED1 mRNA is increased in the SN of PD patients compared to controls^69^. |
| rs9457743 | 2.03E-05 | 6q25.3 | ***MAS1*** | 54 kb upstream | - | - |
| rs9457743 | 2.03E-05 | 6q25.3 | *PNLDC1* | 32 kb downstream | - | - |
| rs1159278 | 2.21E-05 | 13q32.1 | ***MBNL2*** | 39 kb downstream | - | - |
| rs12142266 | 2.31E-05 | 1p22.3 | ***SEP15*** | intronic | - | SEP15 mRNA is downregulated in the SN of PD patients^70^. |
| rs358079 | 2.99E-05 | 3p14.3 | ***CACNA2D3*** | 1.7 kb downstream | - | CACNA2D3 mRNA is decreased in the SN^18^ and striatum^42^ of PD patients compared to controls. CACNA2D3 protein is increased in the CSF of PD patients compared to controls^16^. |
| rs13411180 | 3.09E-05 | 2q31.2 | ***ZNF385B*** | intronic | - | - |
| rs6930229 | 3.14E-05 | 6q25.3 | ***MRPL18*** | 42 kb downstream | - | - |
| rs13157 | 3.52E-05 | 1p36.11 | ***RUNX3*** | 3' UTR | - | RUNX3 mRNA is increased in the SN of PD patients compared to controls^18^. |
| rs929708 | 3.70E-05 | 3p25.3 | ***ATP2B2*** | intronic | **-** | ATP2B2 mRNA is decreased in the SN of PD patients compared to controls^18, 22, 23^. |
| rs10882088 | 3.89E-05 | 10q23.33 | ***KIF11*** | intronic | - | - |
| rs11833635 | 4.09E-05 | 12q15 | *PTPRR* | intronic | - | - |
| rs4777585 | 4.57E-05 | 15q24.1 | ***NEO1*** | intronic | - | - |
| rs12938031 | 4.61E-05 | 17q21.31 | ***CRHR1*** | 7 kb upstream | - | - |
| rs6598020 | 4.63E-05 | 11p15.5 | *ANO9* | 2.6 kb upstream | - | - |
| rs6598020 | 4.63E-05 | 11p15.5 | *PKP3* | 40 kb downstream | - | - |
| rs4927602 | 4.79E-05 | 2p25.3 | ***TPO*** | intronic | - | - |
| **GWAS 8** (Hamza et al.) | | | | | | |
| **SNP** | ***P* value** | **Locus** | **Gene** | **Position ~ gene** [1] | **Corroborating evidence** | |
|  |  |  |  |  | **Genetic studies** | **Expression and immunohistological studies** [2] |
| rs356220 | **3.40E-11** | 4q22.1 | ***SNCA*** | 5 kb downstream | The mutation G209A (Ala53Thr) in SNCA was found in one Italian and three Greek families with PD, but not in controls^35^ and the G88C (Ala30Pro) mutation was found in a German family^36^. SNCA gene duplication^37, 38^ and triplication^39^ causes familial PD. Based on motor Unified PD Rating Scale subscores SNCA (rs356220) is associated with rigidity in PD (P=0.04)^32^. | SNCA mRNA is decreased in the SN of PD patients compared to controls^17, 22, 23^. LB immunoreactive for SNCA were found in the SN of PD patients^40, 41^. |
| rs1350855 | **1.30E-09** | 4q22.1 | ***FAM190A*** | intronic | - | - |
| rs3129882 | **2.90E-08** | 6p21.32 | ***HLA-DRA*** | intronic | The PD associated SNP rs3129882 is an eQTL for HLA-DRA and affects its expression^71, 72^. HLA-DRA intronic variant rs3129882 is associated with late-onset sporadic PD in Chinese Han patients^73^. | HLA-DRA mRNA is increased in the striatum of PD patients compared to controls^20^. |
| rs199533 | 1.30E-06 | 17q21.31 | ***NSF*** | intronic | - | NSF mRNA is decreased in the SN^17, 18, 22, 23, 46-48^ and striatum^46^ of PD patients compared to controls. |
| rs199528 | 1.40E-06 | 17q21.31 | ***WNT3*** | intronic | - | WNT3 mRNA is decreased in the SN of PD patients compared to controls^18^. |
| rs7915262 | 2.80E-06 | 10p13 | ***NMT2*** | intronic | - | - |
| rs3117098 | 2.90E-06 | 6p21.32 | *C6orf10* | 19 kb upstream | - | - |
| rs10741569 | 3.20E-06 | 11p15.3 | ***MICAL2*** | intronic | - | - |
| rs3129955 | 3.80E-06 | 6p21.32 | ***BTNL2*** | intronic | - | BTNL2 mRNA is decreased in the SN of PD patients compared to controls^20^. |
| rs17651549 | 4.80E-06 | 17q21.31 | ***MAPT*** | non-syn coding | Genome-wide significant association (P<5E-08) of MAPT with PD was found in multiple meta-analyses (H1/H2 haplotype; OR=0.78, P=7.97E-52^27^, rs242559; OR=0.78, P=1.5E-10)^28^. | MAPT mRNA is decreased in the SN^21-23^ and MAPT protein is decreased in the CSF^49^ of PD patients compared to controls. |
| rs4790246 | 5.30E-06 | 17p13.2 | *ZFP3* | 10 kb upstream | - | - |
| rs4790246 | 5.30E-06 | 17p13.2 | *ZNF232* | 37 kb downstream | - | - |
| rs12373142 | 5.50E-06 | 17q21.31 | *SPPL2C* | non-syn coding | - | - |
| rs241041 | 5.80E-06 | 17q21.31 | ***CRHR1*** | intronic | - | - |
| rs4678550 | 6.40E-06 | 3p22.2 | *DCLK3* | 23 kb upstream | - | - |
| rs4678550 | 6.40E-06 | 3p22.2 | *TRANK1* | 64 kb downstream | - | - |
| rs36076725 | 6.50E-06 | 17q21.31 | ***KANSL1*** | intronic | - | - |
| rs2957316 | 9.70E-06 | 17q21.31 | *LRRC37A* | 39 kb upstream | - | - |
| **GWAS 9** (Spencer et al.) | | | | | | |
| **SNP** | ***P* value** | **Locus** | **Gene** | **Position ~ gene** [1] | **Corroborating evidence** | |
|  |  |  |  |  | **Genetic studies** | **Expression and immunohistological studies** [2] |
| rs10447854 | **3.11E-09** | 7q32.1 | ***SND1*** | 63 kb downstream | - | - |
| rs356220 | **5.18E-09** | 4q22.1 | ***SNCA*** | 5 kb downstream | The mutation G209A (Ala53Thr) in SNCA was found in one Italian and three Greek families with PD, but not in controls^35^ and the G88C (Ala30Pro) mutation was found in a German family^36^. SNCA gene duplication^37, 38^ and triplication^39^ causes familial PD. Based on motor Unified PD Rating Scale subscores SNCA (rs356220) is associated with rigidity in PD (P=0.04)^32^. | SNCA mRNA is decreased in the SN of PD patients compared to controls^17, 22, 23^. LB immunoreactive for SNCA were found in the SN of PD patients^40, 41^. |
| rs7215239 | **1.49E-08** | 17q21.31 | ***CRHR1*** | intronic | - | - |
| rs8070723 | 5.21E-08 | 17q21.31 | ***MAPT*** | intronic | Genome-wide significant association (P<5E-08) of MAPT with PD was found in multiple meta-analyses (H1/H2 haplotype; OR=0.78, P=7.97E-52^27^, rs242559; OR=0.78, P=1.5E-10)^28^. | MAPT mRNA is decreased in the SN^21-23^ and MAPT protein is decreased in the CSF^49^ of PD patients compared to controls. |
| rs4522464 | 9.84E-07 | 17q23.2 | ***MED13*** | 56 kb upstream | - | - |
| rs4457092 | 4.05E-06 | 5p13.3 | ***CDH6*** | intronic | - | - |
| rs2033884 | 7.33E-06 | 7p15.3 | ***DNAH11*** | intronic | - | DNAH11 mRNA is increased in the striatum of PD patients compared to controls^20^. |
| rs252761 | 7.85E-06 | 5q14.1 | ***AP3B1*** | intronic | - | AP3B1 mRNA is increased in the blood of PD patients compared to controls^15^. |
| rs11256442 | 8.30E-06 | 10p15.1 | ***IL2RA*** | intronic | - | IL2RA mRNA is increased in the striatum of PD patients compared to controls^20^. |
| rs2642444 | 8.94E-06 | 1q41 | ***MARC1*** | intronic | - | - |
| rs10744675 | 2.51E-05 | 12p13.32 | ***KCNA5*** | 8 kb upstream | - | - |
| rs12674264 | 2.95E-05 | 7q21.13 | *ZNF804B* | intronic | - | - |
| rs17183533 | 3.83E-05 | 16p13.3 | ***ABCA3*** | intronic | - | - |
| rs11759658 | 4.38E-05 | 6p22.2 | ***LRRC16A*** | intronic | - | - |
| rs1320163 | 4.73E-05 | 3p22.1 | *ANO10* | 93 kb downstream | - | - |
| rs1320163 | 4.73E-05 | 3p22.1 | ***SNRK*** | 25 kb upstream | - | - |
| rs10477933 | 4.78E-05 | 5q21.3 | ***FER*** | intronic | - | - |
| rs11118618 | 4.86E-05 | 1q41 | ***HLX*** | 41 kb upstream | - | HLX mRNA is increased in the blood of PD patients compared to controls^15^. |
| rs7748486 | 5.15E-05 | 6p24.2 | ***NEDD9*** | intronic | The intronic SNP rs760678 in NEDD9 is associated with susceptibility to PD (OR=1.26, P=0.0017)^74^. | - |
| rs764606 | 5.18E-05 | 12q13.12 | *METTL7A* | 5 kb upstream | - | METTL7A is alternatively spliced in the blood of PD patients compared to controls^75^. |
| rs2242565 | 5.19E-05 | 11p15.5 | *ATHL1* | 3' UTR | - | ATHL1 mRNA is increased in the blood of PD patients compared to controls^15^. |
| rs12831858 | 8.35E-05 | 12q13.12 | *TMPRSS12* | intronic | - | - |
| **GWAS 10** (Saad et al.) | | | | | | |
| **SNP** | ***P* value** | **Locus** | **Gene** | **Position ~ gene** [1] | **Corroborating evidence** | |
|  |  |  |  |  | **Genetic studies** | **Expression and immunohistological studies** [2] |
| rs2736990 | **2.88E-08** | 4q22.1 | ***SNCA*** | intronic | The mutation G209A (Ala53Thr) in SNCA was found in one Italian and three Greek families with PD, but not in controls^35^ and the G88C (Ala30Pro) mutation was found in a German family^36^. SNCA gene duplication^37, 38^ and triplication^39^ causes familial PD. Based on motor Unified PD Rating Scale subscores SNCA (rs356220) is associated with rigidity in PD (P=0.04)^32^. | SNCA mRNA is decreased in the SN of PD patients compared to controls^17, 22, 23^. LB immunoreactive for SNCA were found in the SN of PD patients^40, 41^. |
| rs12294719 | 5.42E-07 | 11p12 | *C11orf74* | 47 kb downstream | - | - |
| rs17690703 | 3.94E-06 | 17q21.31 | *SPPL2C* | 859 bp downstream | - | - |
| rs17690703 | 3.94E-06 | 17q21.31 | ***MAPT*** | 46 kb upstream | Genome-wide significant association (P<5E-08) of MAPT with PD was found in multiple meta-analyses (H1/H2 haplotype; OR=0.78, P=7.97E-52^27^, rs242559; OR=0.78, P=1.5E-10)^28^. | MAPT mRNA is decreased in the SN^21-23^ and MAPT protein is decreased in the CSF^49^ of PD patients compared to controls. |
| rs9899558 | 5.04E-06 | 17p13.2 | ***SPNS2*** | intronic | - | - |
| rs621341 | 5.11E-06 | 2q21.3 | *TMEM163* | intronic | - | - |
| rs26990 | 6.67E-06 | 5q22.2 | ***MCC*** | intronic | - | MCC mRNA is decreased in the SN of PD patients compared to controls^18^. |
| rs4698412 | 6.88E-06 | 4p15 | ***BST1*** | intronic | The SNP rs11724635, intronic in BST1, is associated with an increased risk of PD^27, 76^, whereas this association is stronger in Asian than in Caucasian populations^77^. Further, interaction between this SNP and ever drinking well water was associated with increased PD risk in Taiwanese (heterozygous; OR=1.45, P=0.024 and homozygous OR=1.623, P=0.008), possibly due to heavy metal (arsenic) contamination of well water in Taiwan^78^. | - |
| rs6741233 | 9.34E-06 | 2q35 | ***MREG*** | 52 kb downstream | - | MREG mRNA is decreased in the SN of PD patients compared to controls^17^. |
| rs4954564 | 1.21E-05 | 2q22.1 | ***CXCR4*** | 20 kb upstream | - | CXCR4 mRNA is increased in the SN^44^ and striatum^20, 42^ and CXCR4 is alternatively spliced in the blood^75^ of PD patients compared to controls. CXCR4 expression is increased in the SN of PD patients^79^. |
| rs1035767 | 1.50E-05 | 12q23.3 | ***RFX4*** | intronic | - | RFX4 mRNA is decreased in the SN^18^ and increased in the striatum^20^ of PD patients compared to controls. |
| rs368039 | 1.52E-05 | 4p15.33 | ***HS3ST1*** | 14 kb upstream | - | - |
| rs6729702 | 1.75E-05 | 2q21.3 | ***ACMSD*** | intronic | A meta-analysis on GWAS data identified ACMSD (rs6710823) as a new risk locus for PD (OR=1.38, P=1.35E-09)^76^. | - |
| rs2532269 | 1.90E-05 | 17q21.31 | ***KANSL1*** | intronic | - | - |
| rs1423326 | 2.10E-05 | 5q12.1 | *ZSWIM6* | 18 kb downstream | - | - |
| rs393152 | 2.68E-05 | 17q21.31 | ***CRHR1*** | intronic | - | - |
| rs4964469 | 2.73E-05 | 12q23.3 | *POLR3B* | 46 kb downstream | - | POLR3B protein is increased in the striatum of PD patients compared to controls^20^. |
| rs11064524 | 2.80E-05 | 12p13.33 | ***WNK1*** | intronic | - | WNK1 mRNA is increased in the striatum^20^ and decreased in the blood^15^ of PD patients compared to controls |
| rs9608247 | 2.99E-05 | 22q11.23 | *SUSD2* | 2.2 kb downstream | - | - |
| rs2259599 | 3.74E-05 | 13q34 | *TMCO3* | 1.4 kb downstream | - | - |
| rs12724129 | 4.35E-05 | 1p36.22 | *KIAA2013* | 14 kb downstream | - | - |
| rs12724129 | 4.35E-05 | 1p36.22 | ***PLOD1*** | 37 kb upstream | - | - |
| rs12295401 | 4.40E-05 | 11q13.5 | ***PRKRIR*** | 29 kb downstream | - | - |
| rs10902724 | 5.13E-05 | 1p36.11 | *CEP85* | intronic | - | CEP85 mRNA is increased in the SN of PD patients compared to controls^18^. |
| rs9360414 | 5.34E-05 | 6q13 | *COL19A1* | intronic | - | COL19A1 mRNA is decreased in the blood of PD patients compared to controls^24^. |
| **GWAS 11** (Simon-Sanchez et al.) | | | | | | |
| **SNP** | ***P* value** | **Locus** | **Gene** | **Position ~ gene** [1] | **Corroborating evidence** | |
|  |  |  |  |  | **Genetic studies** | **Expression and immunohistological studies** [2] |
| rs7918386 | 6.39E-06 | 10q22.1 | ***EIF4EBP2*** | intronic | - | - |
| rs2412777 | 1.51E-05 | 15q15.2 | *TGM7* | intronic | - | - |
| rs2736990 | 1.63E-05 | 4q22.1 | ***SNCA*** | intronic | The mutation G209A (Ala53Thr) in SNCA was found in one Italian and three Greek families with PD, but not in controls^35^ and the G88C (Ala30Pro) mutation was found in a German family^36^. SNCA gene duplication^37, 38^ and triplication^39^ causes familial PD. Based on motor Unified PD Rating Scale subscores SNCA (rs356220) is associated with rigidity in PD (P=0.04)^32^. | SNCA mRNA is decreased in the SN of PD patients compared to controls^17, 22, 23^. LB immunoreactive for SNCA were found in the SN of PD patients^40, 41^. |
| rs10497310 | 2.22E-05 | 2q24.3 | *XIRP2* | intronic | - | - |
| rs10504139 | 2.65E-05 | 8q11.23 | *FAM150A* | 1.7 kb downstream | - | - |
| rs6057657 | 2.77E-05 | 20q11.21 | *DEFB119* | 1.1 kb downstream | - | - |
| rs6057657 | 2.77E-05 | 20q11.21 | *DEFB123* | 64 kb upstream | - | - |
| rs12704998 | 3.31E-05 | 7q22.1 | ***NPTX2*** | 40 kb downstream | - | NPTX2 mRNA is increased in the SN^18, 20^ and decreased in the striatum^42^ of PD patients compared to controls. NPTX2 is highly upregulated in PD SN and a component of LB^80^. |
| rs2383025 | 3.61E-05 | 9p22.2 | *CNTLN* | intronic | - | CNTLN mRNA is increased in the blood of PD patients compared to controls^15^. |
| rs4248166 | 4.39E-05 | 6p21.32 | ***BTNL2*** | intronic | - | BTNL2 mRNA is decreased in the SN of PD patients compared to controls^20^. |
| rs1217770 | 4.44E-05 | 5q13.2 | ***MAP1B*** | 96 kb upstream | - | MAP1B mRNA is decreased in the SN of PD patients compared to controls^22, 23^. |
| rs17782975 | 4.46E-05 | 15q15.3 | ***TP53BP1*** | intronic | - | - |
| rs8446 | 5.17E-05 | 2q31.1 | ***ZAK*** | intronic | - | ZAK mRNA is decreased in the SN of female PD patients compared to controls^21^. |
| rs524908 | 5.52E-05 | 15q15.3 | *FRMD5* | intronic | - | FRMD5 mRNA is increased in the SN of PD patients compared to controls^18^. |
| rs17202259 | 6.22E-05 | 6p21.32 | *C6orf10* | 18 kb upstream | - | - |
| rs8132225 | 6.49E-05 | 21q22.13 | ***HLCS*** | intronic | - | - |
| rs2250175 | 6.50E-05 | 4q34.1 | *GLRA3* | 55 kb downstream | - | GLRA3 mRNA is decreased in the SN^20^ and increased in the blood^15^ of PD patients compared to controls. |
| rs2250175 | 6.50E-05 | 4q34.1 | ***HPGD*** | 59 kb upstream | - | HPGD mRNA is decreased in the SN of PD patients compared to controls^17^. |
| rs12184950 | 6.91E-05 | 14q32.32 | ***RCOR1*** | 3.2 kb downstream | - | - |
| rs12184950 | 6.91E-05 | 14q32.32 | ***TRAF3*** | 44 kb upstream | - | TRAF3 mRNA is decreased in the blood of PD patients compared to controls^15^. |
| rs6693597 | 7.01E-05 | 1p31.1 | *NFIA* | intronic | - | - |
| rs2255663 | 7.15E-05 | 15q15.3 | *PPIP5K1* | intronic | - | - |
| rs11177355 | 8.95E-05 | 12q15 | *SLC35E3* | intronic | - | - |
| rs6832140 | 9.42E-05 | 4q22.1 | ***FAM190A*** | intronic | - | - |
| rs4819594 | 9.52E-05 | 22q11.21 | ***CECR2*** | intronic | - | - |
| **GWAS 12** (Do et al.) | | | | | | |
| **SNP** | ***P* value** | **Locus** | **Gene** | **Position ~ gene** [1] | **Corroborating evidence** | |
|  |  |  |  |  | **Genetic studies** | **Expression and immunohistological studies** [2] |
| rs34637584 | **1.82E-28** | 12q12 | ***LRRK2*** | non-syn coding | The mutations c.4322G>A (Arg1441His), c.4321C>G (Arg1441Gly), c.4321C>T (Arg1441Cys), c.5096A>G (Tyr1699Cys), c.6055G>A (Gly2019Ser) and c.6059T>C (Ile2020Thr) in LRRK2 cause late-onset PD and segragate with disease in PD families^81-85^. | LRRK2 protein is increased in the CSF of PD patients compared to controls^16^. |
| i4000416 / rs76763715 | **5.17E-21** | 1q22 | ***GBA*** | non-syn coding / splice site | GBA mutations are associated with (early-onset) parkinsonism^86^ and (early-onset) PD in multiple ethnic populations^87^ (Ashkenazi Jews, P<0.001^88^; Brazillians, P=0.0379^89^; Italians, P=0.0018^90^, caucasian P=0.0001^91^; Chinese, P=0.001^92^; Brazillians, P=0.0047^93^; Greek, P=0.006^94^; Koreans, P<0.01^95^; Serbians, P=0.0041^96^; Mexicans, P=0.014^97^. | GBA enzyme activity is lower in the SN of both PD patients with a GBA mutation (P<0.01) and sporadic PD patients (P<0.05)^98^. |
| rs356219 | **7.91E-21** | 4q22.1 | ***SNCA*** | 9 kb downstream | The mutation G209A (Ala53Thr) in SNCA was found in one Italian and three Greek families with PD, but not in controls^35^ and the G88C (Ala30Pro) mutation was found in a German family^36^. SNCA gene duplication^37, 38^ and triplication^39^ causes familial PD. Based on motor Unified PD Rating Scale subscores SNCA (rs356220) is associated with rigidity in PD (P=0.04)^32^. | SNCA mRNA is decreased in the SN of PD patients compared to controls^17, 22, 23^. LB immunoreactive for SNCA were found in the SN of PD patients^40, 41^. |
| rs1876828 | **1.14E-14** | 17q21.31 | ***CRHR1*** | intronic | - | - |
| rs12185268 | **2.72E-14** | 17q21.31 | *SPPL2C* | non-syn coding | - | - |
| rs1918798 | **3.54E-14** | 17q21.31 | ***KANSL1*** | intronic | - | - |
| rs17563986 | **4.85E-14** | 17q21.31 | ***MAPT*** | intronic | Genome-wide significant association (P<5E-08) of MAPT with PD was found in multiple meta-analyses (H1/H2 haplotype; OR=0.78, P=7.97E-52^27^, rs242559; OR=0.78, P=1.5E-10)^28^. | MAPT mRNA is decreased in the SN^21-23^ and MAPT protein is decreased in the CSF^49^ of PD patients compared to controls. |
| rs415430 | **1.71E-13** | 17q21.31 | ***WNT3*** | intronic | - | WNT3 mRNA is decreased in the SN of PD patients compared to controls^18^. |
| rs199533 | **1.90E-13** | 17q21.31 | ***NSF*** | syn coding | - | NSF mRNA is decreased in the SN^17, 18, 22, 23, 46-48^ and striatum^46^ of PD patients compared to controls. |
| rs11012 | **7.74E-11** | 17q21.31 | ***PLEKHM1*** | non-syn coding, 3' UTR | - | PLEKHM1 mRNA is decreased in the SN of female PD patients compared to controls^23^ and is increased in the blood of PD patients compared to controls^15^. |
| rs10513789 | **2.67E-10** | 3q27.1 | ***MCCC1*** | intronic | The intronic SNP rs11711441 in MCCC1 is in a GWAS meta-analysis associated with PD (OR=0.82, P=1.17E-08^76^; OR=0.86, P=9.20E-10^27^) and also with a lower risk for PD in Han Chinese (OR=0.82, P=0.043^99^; OR=0.43, P<0.001^100^). | - |
| rs6812193 | **7.55E-10** | 4q21.1 | *FAM47E* | intronic | - | - |
| rs6812193 | **7.55E-10** | 4q21.1 | *STBD1* | intronic | - | - |
| rs6599389 | **3.87E-08** | 4p16.3 | *TMEM175* | intronic | - | - |
| rs11868035 | 5.61E-08 | 17p11.2 | ***SREBF1*** | splice site, intronic | Based on motor Unified PD Rating Scale subscores SREBF1 (rs11868035) is associated with gait impairment in PD (P=0.005)^32^. | - |
| rs823156 | 1.27E-07 | 1q32.1 | ***SLC41A1*** | intronic | Three mutations c.436A>G (Lys146Glu), c.1049C>T (Ala350Val) (gain of function^101^) and c.1440A>G (Pro480Pro) were identified in the SLC41A1 gene in PD patients, but not in controls^102, 103^. | - |
| rs11724804 | 2.43E-07 | 4p16.3 | ***DGKQ*** | intronic | Genome-wide significance was reached for the intronic SNP rs11248060 in DGKQ (OR=1.21; P=3.04E-12) ^27^ (OR=1.35; P=2.0E-9)^28^, a SNP that also increases the risk for PD in a Chinese population (P<0.05)^29^. | - |
| rs4130047 | 2.44E-07 | 18q12.3 | ***RIT2*** | intronic | In a meta-analysis of GWASs RIT2 was identified as a novel susceptibility locus (rs12456492, OR=1.19, P=2E-10 (combined sample)^28^. | RIT2 mRNA is decreased in the SN of PD patients compared to controls^17^. |
| rs482912 | 5.17E-07 | 3q27.1 | ***LAMP3*** | non-syn coding | - | - |
| rs4925114 | 6.75E-07 | 17p11.2 | ***RAI1*** | intronic | - | - |
| rs9878775 | 1.00E-06 | 3q26.33 | ***DCUN1D1*** | 3' UTR | - | - |
| rs278901 | 1.17E-06 | 12q12 | ***CNTN1*** | intronic | - | CNTN1 mRNA and protein^20^ is increased in the striatum and decreased in the CSF^16^ of PD patients compared to controls. |
| rs4925119 | 1.55E-06 | 17p11.2 | ***TOM1L2*** | 7 kb downstream | - | - |
| rs10999435 | 1.81E-06 | 10q22.1 | ***PALD1*** | 48 kb downstream | - | PALD1 mRNA is increased in the SN of PD patients compared to controls^18^. |
| rs10999435 | 1.81E-06 | 10q22.1 | ***PRF1*** | 13 kb upstream | - | - |
| rs823114 | 2.12E-06 | 1q32.1 | *NUCKS1* | regulatory region | - | - |
| rs660895 | 2.13E-06 | 6p21.32 | ***HLA-DRB1*** | intronic | The PD associated SNP rs2395163 is an eQTL for HLA-DRB1 and affects its expression^72^. | HLA-DRB1 mRNA is increased in the striatum of PD patients compared to controls^20^. |
| rs7451962 | 2.49E-06 | 6p21.32 | ***HLA-DQB1*** | 45 kb downstream | The alleles HLA-DQB1*03:02 (OR=0.75, P=3E-04) and HLA-DQB1*06:02 (OR=1.26, P=7E-04) are associated with PD^72^. The PD associated SNP rs3129882 is an eQTL for HLA-DQB1 and affects its expression^71, 72^. | HLA-DQB1 mRNA is increased in the striatum of PD patients compared to controls^20^. |
| rs1564282 | 2.56E-06 | 4p16.3 | ***GAK*** | intronic | SNPs in GAK rs1564282 (Caucasian; OR=1.61; P=0.0151)^30^, rs1564282 (Chinese)^29^, rs11248051 (Taiwanese; CT/TT vs CC genotypes OR=1.37; P=0.03)^31^, rs11248051 (meta-analysis; OR=1.35; P=8.2E-09)^28^ are associated with an increased PD risk. Based on motor Unified Parkinson's Disease Rating Scale subscores GAK (rs1564282) is associated with tremor in PD (P=0.03) )^32^. | - |
| rs9379968 | 2.59E-06 | 6p22.1 | *POM121L2* | 9 kb downstream | - | - |
| rs9379968 | 2.59E-06 | 6p22.1 | *ZNF391* | 98 kb upstream | - | ZNF391 mRNA is increased in the striatum of PD patients compared to controls^20^. |
| rs2894181 | 3.32E-06 | 6p21.33 | *HCG27* | 2.8 kb downstream | - | - |
| rs2894181 | 3.32E-06 | 6p21.33 | *PSORS1C3* | 29 kb upstream | - | - |
| rs2837740 | 3.62E-06 | 21q22.2 | ***DSCAM*** | intronic | - | - |
| rs1467751 | 3.62E-06 | 21q22.2 | ***TMPRSS3*** | intronic | - | - |
| rs7080373 | 3.66E-06 | 10q22.1 | ***ADAMTS14*** | 76 kb upstream | - | ADAMTS14 mRNA is decreased in the SN of PD patients compared to controls^20^. |
| rs9917256 | 3.72E-06 | 2q24.3 | ***STK39*** | 39 kb upstream | A meta-analysis on GWAS data identified STK39 (rs2102808) as a new risk locus for PD (OR=1.28, P=3.31E-11)^76^, which was replicated in a Caucasian (OR=1.21, P<0.001, Sharma, 2012) and a Scandinavian PD group (OR=1.34, P=0.0005)^104^. A meta-analysis on GWAS data also showed association of rs2390669 intronic in STK39 with PD (OR=1.19, P=1.37E-09)^27^. | - |
| rs3763309 | 4.70E-06 | 6p21.32 | ***BTNL2*** | intronic | - | BTNL2 mRNA is decreased in the SN of PD patients compared to controls^20^. |
| rs11878694 | 5.59E-06 | 19p13.2 | *MBD3L4* | 41 kb downstream | - | - |
| rs11878694 | 5.59E-06 | 19p13.2 | *MBD3L5* | 34 kb upstream | - | - |
| rs10886515 | 5.86E-06 | 10q26.11 | ***TIAL1*** | intronic | - | TIAL1 mRNA is decreased in the SN of PD patients compared to controls^23^. |
| rs1882642 | 6.27E-06 | 2q22.1 | ***LRP1B*** | intronic | - | LRP1B mRNA is decreased in the SN of PD patients compared to controls^20^. |
| rs35883 | 6.67E-06 | 3p24.1 | *RBMS3* | intronic | - | - |
| rs659445 | 7.20E-06 | 6p21.33 | ***EHMT2*** | intronic | - | - |
| rs1471738 | 8.60E-06 | 3q12.3 | *IMPG2* | 29 kb downstream | - | - |
| rs3957148 | 8.83E-06 | 6p21.32 | ***HLA-DQA2*** | 27 kb upstream | The PD associated SNPs rs3129882 and rs2395163 are eQTLs for HLA-DQA2 and affect its expression^71, 72^. | - |
| rs3957148 | 8.83E-06 | 6p21.32 | ***HLA-DQB2*** | 42 kb downstream | - | - |
| rs11097338 | 9.03E-06 | 4q21.1 | *CCDC158* | intronic | - | - |
| rs4925138 | 9.22E-06 | 17p11.2 | ***DRG2*** | intronic | - | - |
| rs9275184 | 1.09E-05 | 6p21.32 | ***HLA-DQA1*** | 40 kb downstream | The alleles HLA-DQA1*01:02 (OR=1.15, P=0.02) and HLA-DQA1*03:01 (OR=0.77, P=1E-04) are associated with PD^72^. The PD associated SNP rs2395163 is an eQTL for HLA-DQA1 and affects its expression^72^. | HLA-DQA1 mRNA is increased in the striatum of PD patients compared to controls^20^. |
| rs1568069 | 1.24E-05 | 2q37.2 | ***AGAP1*** | intronic | - | - |
| rs2139950 | 1.30E-05 | 3q13.31 | ***ZBTB20*** | 5 kb upstream | - | - |
| rs7251424 | 1.36E-05 | 19p13.3 | ***LSM7*** | 92 bp downstream | - | - |
| rs7251424 | 1.36E-05 | 19p13.3 | ***SPPL2B*** | 7 kb upstream | - | SPPL2B protein is decreased in the CSF of PD patients compared to controls^16^. |
| rs7803999 | 1.37E-05 | 7p12.3 | *ABCA13* | intronic | - | ABCA13 protein is increased in the CSF of PD patients compared to controls^16^. |
| rs2072369 | 1.51E-05 | 7p15.3 | *KLHL7* | 5' UTR | - | KLHL7 mRNA is decreased in the SN of PD patients compared to controls^18^. |
| rs10519001 | 1.55E-05 | 15q22.2 | ***MYO1E*** | intronic | - | MYO1E mRNA is increased in the SN of PD patients compared to controls^18^. |
| rs12694823 | 1.55E-05 | 2q36.3 | ***FBXO36*** | 74 kb downstream | - | - |
| rs12694823 | 1.55E-05 | 2q36.3 | *SLC16A14* | 18 kb upstream | - | SLC16A14 mRNA is decreased in the SN of PD patients compared to controls^47^. |
| rs3905495 | 2.01E-05 | 6p21.33 | ***HLA-B*** | 56 kb downstream | The alleles HLA-B*07:02 (OR=1.20, P=8E-03) and HLA-B*40:01 (OR=0.79, P=0.02) are associated with PD^72^. | - |
| rs3905495 | 2.01E-05 | 6p21.33 | ***HLA-C*** | 26 kb upstream | The alleles HLA-C*03:04 (OR=0.76, P=2E-03) and HLA-C*07:02 (OR=1.22, P=3E-03) are associated with PD^72^. | - |
| rs10992619 | 2.05E-05 | 9q22.31 | *SUSD3* | intronic | - | SUSD3 mRNA is increased in the SN^18^ and striatum^20^ of PD patients compared to controls. |
| rs3792424 | 2.12E-05 | 3p26.2 | ***IL5RA*** | intronic | - | IL5RA mRNA is increased in the striatum of PD patients compared to controls^20^. |
| rs11946079 | 2.16E-05 | 4q21.1 | ***SCARB2*** | intronic | The SNP rs6825004, intronic in SCARB2 (OR=0.71, P=0.03), as well as a haplotype of 5 intronic SNPs in SCARB2 is associated with PD in Greek patients (OR=1.75, P=0.004)^105^. | - |
| rs9461362 | 2.26E-05 | 6p22.1 | *PRSS16* | 80 kb downstream | - | - |
| rs8012152 | 2.33E-05 | 14q22.2 | ***WDHD1*** | intronic | - | - |
| rs34255679 | 2.35E-05 | 16p13.3 | ***RBFOX1*** | intronic | - | RBFOX1 mRNA is increased in the striatum of PD patients compared to controls^19^. |
| rs6735555 | 2.37E-05 | 2q37.1 | ***SP110*** | 11 kb downstream | - | SP110 is alternatively spliced^75^ and SP110 mRNA is increased^15^ in the blood of PD patients compared to controls. |
| rs6735555 | 2.37E-05 | 2q37.1 | *SP140* | 69 kb upstream | - | - |
| rs4698412 | 2.41E-05 | 4p15.32 | ***BST1*** | intronic | The SNP rs11724635, intronic in BST1, is associated with an increased risk of PD^27, 76^, whereas this association is stronger in Asian than in Caucasian populations^77^. Further, interaction between this SNP and ever drinking well water was associated with increased PD risk in Taiwanese (heterozygous; OR=1.45, P=0.024 and homozygous OR=1.623, P=0.008), possibly due to heavy metal (arsenic) contamination of well water in Taiwan^78^. | - |
| rs1688609 | 2.45E-05 | 6q22.31 | ***CEP85L*** | 3.9 kb upstream | - | CEP85L mRNA is decreased in the SN of PD patients compared to controls^18^. |
| rs1688609 | 2.45E-05 | 6q22.31 | *MCM9* | 99 kb downstream | - | - |
| rs947211 | 2.55E-05 | 1q32.1 | ***RAB7L1*** | 8 kb upstream | A RAB7L1 mutation (c.379-12insT) is associated with PD (OR=3.3, P=0.0399) and a novel mutation (Lys157Arg) was found in one PD patient^102^. The SNPs rs1572931 and rs823144 are localized in the putative RAB7L1 promotor and are associated with a reduced PD risk in Ashkenazim Jews (OR=0.64, P=0.0002; OR=0.72, P=0.002)^106^. | - |
| rs2736994 | 2.56E-05 | 4q22.1 | ***MMRN1*** | 16 kb upstream | - | MMRN1 mRNA is increased in the striatum of PD patients compared to controls^20^. |
| rs854791 | 2.57E-05 | 17p11.2 | *MYO15A* | intronic | - | - |
| rs11065598 | 2.66E-05 | 12q24.31 | ***KDM2B*** | intronic | - | - |
| rs708382 | 2.81E-05 | 17q21.31 | *FAM171A2* | 1.1 kb upstream | - | - |
| rs708382 | 2.81E-05 | 17q21.31 | ***ITGA2B*** | 7 kb downstream | - | - |
| rs11564252 | 2.83E-05 | 12q12 | *MUC19* | intronic | - | - |
| rs7018431 | 2.89E-05 | 8p23.3 | *FAM87A* | 45 kb downstream | - | - |
| rs7018431 | 2.89E-05 | 8p23.3 | ***FBXO25*** | 76 kb upstream | - | - |
| rs138054 | 2.95E-05 | 22q13.31 | ***EFCAB6*** | 11 kb upstream | - | - |
| rs138054 | 2.95E-05 | 22q13.31 | *SULT4A1* | 817 bp downstream | - | SULT4A1 mRNA is decreased in the SN of PD patients compared to controls^18^. |
| rs12132270 | 3.03E-05 | 1q32.1 | ***SLC45A3*** | 11 kb upstream | - | SLC45A3 mRNA is increased in the SN of PD patients compared to controls^18^. |
| rs2801943 | 3.17E-05 | 10q11.21 | *CCNYL2* | intronic | - | - |
| rs6140909 | 3.43E-05 | 20p12.2 | ***PLCB4*** | intronic | - | - |
| rs16860458 | 3.44E-05 | 2q31.1 | ***ITGA6*** | intronic | - | - |
| rs17763599 | 3.50E-05 | 19p13.3 | ***TMPRSS9*** | 20 kb upstream | - | - |
| rs11727049 | 3.66E-05 | 4q28.2 | *LARP1B* | intronic | - | - |
| rs12464032 | 3.79E-05 | 2q14.3 | *CNTNAP5* | intronic | - | CNTNAP5 mRNA is increased in (female) SN^21^ or decreased in SN PD patients compared to controls^18^. |
| rs8116325 | 3.90E-05 | 20q12 | ***PTPRT*** | intronic | A copy number variant study showed association of a heterozygous deletion in the intron of PTPRT with PD^107^. | - |
| rs1867153 | 3.91E-05 | 15q24.2 | ***MPI*** | 79 kb downstream | - | - |
| rs1867153 | 3.91E-05 | 15q24.2 | ***SCAMP5*** | 17 kb upstream | - | SCAMP5 mRNA is decreased in the SN of PD patients compared to controls^18^. |
| rs10819174 | 3.95E-05 | 9q33.3 | *MVB12B* | 3' UTR | - | MVB12B mRNA is increased^18^ or decreased^108^ in the SN of PD patients compared to controls. |
| rs9948128 | 4.03E-05 | 18p11.31 | *L3MBTL4* | 62 kb downstream | - | - |
| rs9948128 | 4.03E-05 | 18p11.31 | *TMEM200C* | 414 bp upstream | - | TMEM200C mRNA is decreased in the SN of PD patients compared to controls^17^. |
| rs8054636 | 4.21E-05 | 16p12.3 | *SYT17* | intronic | - | SYT17 mRNA is decreased in the SN of PD patients compared to controls^18^. |
| rs1265093 | 4.22E-05 | 6p21.33 | *PSORS1C1* | intronic | - | - |
| rs4076437 | 4.22E-05 | 3q21.3 | ***H1FX*** | 829 bp upstream | - | - |
| rs2553427 | 4.35E-05 | 3q12.3 | ***SENP7*** | intronic | - | - |
| i4000434 / rs80338939 | 4.47E-05 | 13q12.11 | ***GJB2*** | Deletion / frameshift | - | - |
| rs2968538 | 4.58E-05 | 7q11.22 | *CALN1* | intronic | - | CALN1 mRNA is decreased in the SN of PD patients compared to controls^17^. |
| rs17615676 | 4.73E-05 | 7p21.3 | ***ARL4A*** | 8 kb upstream | - | ARL4A mRNA is increased in the SN of PD patients compared to controls^18^. |
| rs17615676 | 4.73E-05 | 7p21.3 | ***SCIN*** | 25 kb downstream | - | SCIN mRNA is increased in the striatum of PD patients compared to controls^19, 20^. |
| rs10038927 | 4.80E-05 | 5q12.3 | *ADAMTS6* | intronic | - | - |
| rs17126237 | 4.92E-05 | 8p22 | ***ASAH1*** | 39 kb upstream | - | ASAH1 mRNA is decreased in the SN^23^ and increased in the blood^15^ and ASAH1 protein is increased in the striatum^20^ and CSF^16^ of PD patients compared to controls. ASAH1 is alternatively spliced in the blood of PD patients compared to controls^75^. |
| rs17126237 | 4.92E-05 | 8p22 | ***PCM1*** | 94 kb downstream | - |  |
| rs9897702 | 5.00E-05 | 17q21.2 | ***ATP6V0A1*** | 1.5 kb upstream | - | ATP6V0A1 mRNA is decreased in the SN of PD compared to controls^22, 108^. |
| rs10074991 | 5.11E-05 | 5p13.1 | ***PRKAA1*** | Intronic | - (Part of the AMPK-complex). | - |
| rs909626 | 5.19E-05 | 6p22.3 | ***JARID2*** | intronic | - | - |
| rs11675641 | 5.27E-05 | 2p12 | *GCFC2* | 39 kb upstream | - | - |
| rs11675641 | 5.27E-05 | 2p12 | ***MRPL19*** | 60 kb downstream | - | - |
| rs1920650 | 5.27E-05 | 3q25.33 | ***KPNA4*** | 52 kb upstream | - | - |
| rs902910 | 5.45E-05 | 4q35.2 | *TRIML2* | intronic | - | - |
| rs17529642 | 5.55E-05 | 2q33.1 | *TYW5* | 97 kb downstream | - | - |
| rs17529642 | 5.55E-05 | 2q33.1 | *C2orf69* | 78 kb upstream | - | - |
| rs755690 | 5.82E-05 | 19q13.2 | ***ACTN4*** | intronic | - | ACTN4 mRNA^18^ and protein^26^ is increased in the SN of PD patients compared to controls. |
| rs624032 | 6.01E-05 | 10p12.1 | ***BAMBI*** | 8 kb upstream | - | - |
| rs624032 | 6.01E-05 | 10p12.1 | *WAC* | 47 kb downstream | - | - |
| rs716409 | 6.23E-05 | 13q14.3 | ***PCDH8*** | 20 kb upstream | - | PCDH8 mRNA is decreased in the SN of PD patients compared to controls^17, 20^. |
| rs4793039 | 6.46E-05 | 17q21.2 | ***FAM134C*** | intronic | - | - |
| rs11780980 | 6.53E-05 | 8p23.1 | *C8orf12* | intronic | - | - |
| rs11780980 | 6.53E-05 | 8p23.1 | *FAM167A* | intronic | - | - |
| rs11145739 | 6.70E-05 | 9q21.2 | *CEP78* | 43 kb upstream | - | - |
| rs2270822 | 6.98E-05 | 5q12.1 | *SMIM15* | 739 bp upstream | - | - |
| rs2270822 | 6.98E-05 | 5q12.1 | ***NDUFAF2*** | 10 kb downstream | - | - |
| rs7402147 | 7.04E-05 | 15q12 | *GABRG3* | intronic | - | GABRG3 mRNA is decreased in the SN of PD patients compared to controls^20^. |
| rs10934878 | 7.08E-05 | 3q21.3 | *HMCES* | intronic | - | HMCES mRNA is decreased in the SN of female PD patients compared to controls^23^. |
| rs2242656 | 7.12E-05 | 6p21.33 | ***BAG6*** | intronic | - | BAG6 mRNA is decreased in the blood of PD patients compared to controls^15^. |
| rs2395157 | 7.12E-05 | 6p21.32 | *C6orf10* | 8 kb upstream | - | - |
| rs1266071 | 7.18E-05 | 6p21.33 | ***BAT5*** | intronic | - | - |
| rs1266071 | 7.18E-05 | 6p21.33 | *LY6G6E* | intronic | - | - |
| rs1901632 | 7.20E-05 | 10p15.1 | *AKR1E2* | 16 kb upstream | - | - |
| rs7320516 | 7.27E-05 | 13q13.3 | *PROSER1* | 31 kb upstream | - | - |
| rs7320516 | 7.27E-05 | 13q13.3 | *NHLRC3* | 18 kb downstream | - | - |
| rs6576808 | 7.33E-05 | 1p22.3 | *COL24A1* | 16 kb upstream | - | - |
| rs7147286 | 7.55E-05 | 14q22.2 | ***GCH1*** | intronic | - | - |
| rs6905949 | 7.58E-05 | 6p22.1 | *TRIM10* | 12 kb upstream | - | - |
| rs6905949 | 7.58E-05 | 6p22.1 | *TRIM15* | 52 bp downstream | - | - |
| rs1406773 | 8.24E-05 | 3q22.1 | ***CPNE4*** | intronic | - | - |
| rs4255064 | 8.44E-05 | 7q22.1 | *KPNA7* | 75 kb upstream | - | - |
| rs2721819 | 8.47E-05 | 7p15.3 | *DFNA5* | 3' UTR | - | - |
| rs3132453 | 8.76E-05 | 6p21.33 | ***BAT2*** | non-syn coding | - | - |
| rs17632029 | 8.92E-05 | 10p13 | ***FRMD4A*** | intronic | - | - |
| rs6873137 | 9.01E-05 | 5q33.3 | ***HAVCR1*** | 22 kb downstream | - | HAVCR1 mRNA is decreased in the SN and increased in the striatum of PD patients compared to controls^20^. |
| rs6873137 | 9.01E-05 | 5q33.3 | ***TIMD4*** | 44 kb upstream | - | TIMD4 mRNA is increased in the striatum of PD patients compared to controls^20^. |
| rs979316 | 9.14E-05 | 20q13.31 | ***BMP7*** | 87 kb downstream | - | BMP7 mRNA is decreased in the blood of PD patients compared to controls^15^. |
| rs6464536 | 9.27E-05 | 7q34 | *KEL* | 30 kb upstream | - | KEL mRNA is increased in the SN of PD patients compared to controls^18^. |
| rs6464536 | 9.27E-05 | 7q34 | *OR9A2* | 34 kb downstream | - | - |
| rs10928374 | 9.31E-05 | 2q23.1 | *MBD5* | intronic | - | - |
| rs10211158 | 9.40E-05 | 2q14.3 | *GYPC* | 72 kb upstream | - | - |
| rs11174631 | 9.64E-05 | 12q12 | ***SLC2A13*** | intronic | An interaction between the SNP rs2896905 in SLC2A13 and caffeine intake or smoking and caffeine intake combined is associated with PD risk i.e., high caffeine intake reduces PD risk (OR=0.81, P=0.049), never smokers with low caffeine intake have a higher PD risk (OR=1.35, P=0.04) and smokers with high caffeine intake have a lower PD risk (OR=0.68, P=0.007)^53^. | SLC2A13 mRNA is decreased in the SN of PD patients compared to controls^18^. |
| rs7297212 | 9.89E-05 | 12p13.31 | ***NTF3*** | intronic | - | In the PD SN increased numbers of NTF3 immunoresponsive ramified glia cells surrounded fragmented neurons^109^. |
| rs4072739 | 9.96E-05 | 17p11.2 | *LRRC48* | intronic | - | - |
| **GWAS 13** (Liu et al.) | | | | | | |
| **SNP** | ***P* value** | **Locus** | **Gene** | **Position ~ gene** [1] | **Corroborating evidence** | |
|  |  |  |  |  | **Genetic studies** | **Expression and immunohistological studies** [2] |
| rs151358 | 2.24E-06 | 20q13.32 | *SLMO2* | 3' UTR | - | - |
| rs1879512 | 3.04E-06 | 3q13.2 | *SLC9A10* | 81 kb upstream | - | - |
| rs1916642 | 3.41E-06 | 5q13.2 | *TMEM171* | 25 kb downstream | - | TMEM171 mRNA is decreased in the striatum of PD patients compared to controls^20^. |
| rs1916642 | 3.41E-06 | 5q13.2 | *TMEM174* | 16 kb upstream | - | - |
| rs12613026 | 6.49E-06 | 2p21 | *HAAO* | intronic | - | - |
| rs1684524 | 7.74E-06 | 3p24.3 | *ZNF385D* | intronic | - | ZNF385D mRNA is decreased in the SN of PD patients compared to controls^18^. |
| rs10999501 | 9.75E-06 | 10q22.1 | ***ADAMTS14*** | intronic | - | ADAMTS14 mRNA is decreased in the SN of PD patients compared to controls^20^. |
| rs11661054 | 1.01E-05 | 18q23 | ***MBP*** | intronic | - | MBP mRNA is increased in the SN^70, 108^ and MBP mRNA^42^ and protein^20^ are decreased in the striatum of PD patients compared to controls. Serum IgM autoantibodies against MBP are increased in PD patients (P<0.0001) and increase during disease progression (P<0.05)^110^. |
| rs7464066 | 1.35E-05 | 8q24.3 | *ZFP41* | intronic | - | - |
| rs8030609 | 1.78E-05 | 15q13.3 | *TMCO5B* | 4 kb upstream | - | - |
| rs9867544 | 3.08E-05 | 3q13.2 | ***CD200*** | intronic | - | CD200 mRNA is decreased in the striatum^46^ and BA9^46^ of PD patients compared to controls. CD200 protein is decreased in the SN^26^ and CSF^16^ of PD patients compared to controls. |
| rs10415765 | 3.16E-05 | 19p13.11 | ***LRRC25*** | 54 kb downstream | - | - |
| rs10415765 | 3.16E-05 | 19p13.11 | *PGPEP1* | 3 kb upstream | - | - |
| rs7171137 | 3.79E-05 | 15q26.1 | ***SLCO3A1*** | intronic | - | SLCO3A1 mRNA is increased in the SN^18, 21^ and blood^15^ of PD patients compared to controls. |
| rs12469652 | 3.95E-05 | 2p22.2 | *VIT* | intronic | - | - |
| rs12734001 | 4.03E-05 | 1q32.1 | ***PPP1R12B*** | Intronic | - (Part of the PP1-complex). | PPP1R12B mRNA is decreased in the SN of female PD patients compared to controls^21^ and is increased in the blood of PD patients compared to controls^15^. |
| rs225376 | 4.30E-05 | 21q22.3 | ***ABCG1*** | intronic | - | ABCG1 protein is increased in the CSF of PD patients compared to controls^16^. |
| rs7129006 | 4.61E-05 | 11p14.3 | ***GAS2*** | intronic | - | - |
| rs2186580 | 4.69E-05 | 11q22.1 | ***TRPC6*** | 70 kb upstream | - | TRPC6 mRNA is decreased in the blood of PD patients compared to controls^24^. |
| rs10121009 | 5.32E-05 | 9p13.3 | ***UNC13B*** | intronic | - | - |
| rs3808386 | 5.37E-05 | 8q22.1 | ***MTERFD1*** | intronic | - | - |
| rs2183593 | 5.43E-05 | 21q22.3 | ***COL18A1*** | 33 kb upstream | - | COL18A1 mRNA is increased in the SN^18^ and COL18A1 protein is decreased in the CSF^16^ of PD compared to controls. |
| rs4745122 | 5.48E-05 | 9q21.13 | *ABHD17B* | 85 kb downstream | - | ABHD17B mRNA is increased in the SN of PD patients compared to controls^18^. |
| rs4745122 | 5.48E-05 | 9q21.13 | ***TMEM2*** | 9 kb upstream | - | TMEM2 mRNA is increased in the SN of PD patients compared to controls^18^. |
| rs2843518 | 5.63E-05 | Xq25 | *TENM1* | intronic | - | - |
| rs1194491 | 5.64E-05 | 10q21.1 | ***PRKG1*** | intronic | - | - |
| rs2266920 | 5.76E-05 | 7p22.3 | *AMZ1* | intronic | - | - |
| rs2266920 | 5.76E-05 | 7p22.3 | ***GNA12*** | intronic | - | - |
| rs2158133 | 6.19E-05 | 12q13.11 | ***AMIGO2*** | 19 kb downstream | - | - |
| rs2158133 | 6.19E-05 | 12q13.11 | *PCED1B* | 23 kb upstream | - | - |
| rs4976493 | 6.66E-05 | 5q31.1 | *SLC25A48* | intronic | - | SLC25A48 mRNA is increased in the striatum^20^ and BA9^51^ of PD patients compared to controls. |
| rs183211 | 7.16E-05 | 17q21.31 | ***NSF*** | intronic | - | NSF mRNA is decreased in the SN^17, 18, 22, 23, 46-48^ and striatum^46^ of PD patients compared to controls. |
| rs11714053 | 7.98E-05 | 3q22.1 | ***CPNE4*** | intronic | - | - |
| rs415430 | 8.45E-05 | 17q21.31 | ***WNT3*** | intronic | - | WNT3 mRNA is decreased in the SN of PD patients compared to controls^18^. |
| rs4678649 | 8.54E-05 | 3p22.3 | ***CCR4*** | 19 kb downstream | - | - |
| rs12985786 | 8.86E-05 | 19p13.2 | ***CACNA1A*** | intronic | - | CACNA1A protein is increased in the CSF of PD patients compared to controls^16^. |
| rs13003114 | 8.92E-05 | 2q31.2 | ***ZNF385B*** | intronic | - | - |

[1] Genetic position according to the Ensembl Human Genome Browser (<http://www.ensembl.org/Homo_sapiens/>).

[2] Expression data from genome wide expression studies in PD-associated brain areas (SN, striatum, BA9), CSF or blood were included if they met the following criteria; *with* an adjusted p-value for multiple comparisons <0.05, mRNAs should have a fold change of <-1.5 or >1.5 and proteins <-1.2 or >1.2, and *without* a published adjusted p-value, mRNAs and proteins were only included with a p-value <0.05 and a fold change of <-2.0 or >2.0 and of <-1.5 or >1.5 respectively.

| **Supplementary Table 3. Network enrichment analysis** of the PD GWAS candidate genes from **Supplementary** **Table 2** using Ingenuity pathway software (www.ingenuity.com). Separate networks of interacting proteins were generated and ranked by their enrichment score (see [1]). Network 1, the network with the highest score and also containing the highest number of molecules/proteins, is shown in **Supplementary** **Figure 1**. The five most significantly enriched networks are shown. In addition, the PD GWAS genes encoding proteins that could be directly placed in the molecular PD landscape are indicated in bold. | | | |
| --- | --- | --- | --- |
| **Rank** | **PD GWAS genes in network** | **Score** [1] | ***P*** [1] |
| 1 | **ABCG1**, **ATF6**, **ATP6V0A1**, **CCAR2**, **CDK19**, **CENPC**, CEP152, **CUL2**, **DCUN1D1**, **DDX3X**, **DGKQ**, **DLG2**, **FRMD4A**, **KIF11**, **LIPC**, **MCC**, **MED13**, **PARD3**, **PCM1**, PKDCC, **PLOD1**, **RPL38**, **RPL7A**, **SREBF1**, **TIAL1**, **TMCC3**, **TMX1**, **ULK2** | 44 | 1.00E-44 |
| 2 | **ARL4A**, **ASAH1**, **AXIN1**, **BTNL2**, **EIF4EBP2**, **GJB2**, **HLCS**, **HPGD**, **JARID2**, **KCNA5**, **MSX1**, **NTF3**, **PROK2**, **ROBO2**, **RUNX3**, **SEMA6D**, SLC22A16, **SP110**, SP140, **TBX3**, **TCF12**, **TLE1**, **TRPC6**, **WNT3** | 37 | 1.00E-37 |
| 3 | **ADAMTS2**, **ADAMTS14**, **BMP7**, **CD200**, **CNTN1**, **COL18A1**, **COL2A1**, **ENSA**, EXT1, **FGF12**, **GAK**, **ITGA6**, **ITGA8**, **LAMP1**, **LRP1B**, **NLGN1**, **RAP2A**, **RER1**, **SCN2A**, **SDC1**, **SERPINB5**, **SERPING1**, **SMAD5** | 35 | 1.00E-35 |
| 4 | **CYP17A1**, **DSCAM**, **FBN1**, **GNA12**, **SIK1**, **LRRK2**, **LTBP1**, **MAP2K6**, **MBP**, MYO15A, **PIK3CD**, **PPM1L**, **PRKAA1**, **PSMD11**, **SEMA3E**, **SND1**, **SNRK**, **STK39**, **WNK1**, XIRP2, **ZAK** | 29 | 1.00E-29 |
| 5 | **AGAP1**, **AMIGO2**, **AP3B1**, **CPNE4**, **GAS2**, **ITGA2B**, **ITGAL**, **KPNA4**, **MAP1B**, **MYO1E**, **OCA2**, **PPP1R12B**, **PRKG1**, **RAP1A**, **SCIN**, SYT17, **TPO**, **TRAPPC9**, **VPS41**, ZNF207 | 29 | 1.00E-29 |

[1] This score takes into account the number of eligible molecules/proteins in the network and its size, as well as the total number of network-eligible molecules analyzed and the total number of molecules in the Ingenuity Knowledge Base that could potentially be included in networks. The score is calculated with the right-tailed Fisher's exact test and displayed as the negative logarithm of the Fisher’s exact test result.

| **Supplementary Table 4. Additional genes** that encode proteins located in the PD landscape. If these genes or their protein products are linked to PD, either by genetic or by expression and immunohistological studies (in PD patients), this is shown in the column ‘Corroborating evidence’. Single underlined genes are genetically associated with PD, dotted underlined genes encode proteins that are differentially expressed in PD patients and double underlined genes are both genetically associated with PD and encode a protein that is differentially expressed in PD. | | | |
| --- | --- | --- | --- |
| **Gene** | **Locus** | **Corroborating evidence** | |
|  |  | **Genetic studies** | **Expression and immunohistological studies** [1] |
| ABCA1 | 9q31.1 | - | ABCA1 protein is increased in CSF of PD patients compared to controls^111^. |
| ADH1C | 4q23 | A mutation in ADH1C c.232G>T (rs283413), introducing a stop codon, increases the risk for PD (OR=3.25, P=0.007)^112^. | - |
| AICDA | 12p13.31 | - | - |
| AKT1 | 14q32.33 | - | Levels of total and Ser473-phosphorylated AKT1 is lower in the PD brain, but are increased in glia cells in the SN of PD patients^113^. |
| AKT1S1 | 19q13.33 | - (Part of the mTORC1-complex). | - |
| APOE | 19q13.32 | In Thai PD patients the ApoE-ε2 allele shows an increased risk (OR=2.309)^114^, in non-Hispanic Caucasians PD patients the ApoE-ε4 allele shows a lower risk (OR=0.75)^115^, whereas in mexican PD patients the ApoE-ε3 allele shows a protective risk effect (OR=0.36) and the ApoE-ε4 allele shows an increased risk for PD (OR=2.57)^116^. | APOE protein is higher expressed in melanized neurons of the SN of early PD patients^117^, higher expressed in the striatum of PD patients^20^ and dysregulated in the CSF of PD patients compared to controls^16, 49, 118^. |
| AR | Xq12 | - | AR mRNA is increased in the striatum^20^ and decreased in the SN of PD patients compared to controls^18^. |
| ASCL1 | 4q35.1 | Polyglutamine length variants in the MASH1 gene are associated with PD risk^119^. | ASCL1 mRNA is increased in the straitum of PD patients compared to controls^42^. |
| ATG5 | 6q21 | A variant in the ATG5 promotor (106774459T>A) enhancing its transcriptional activity (P<0.01) was identified in one patient, but not in controls^120^. | ATG5 mRNA expression is increased in nucleated blood cells^15^ and in leukocytes^120^ of PD patients compared to controls. |
| ATG7 | 3p25.3 | Four variants (11313449G>A, 11313811T>C, 11313913G>A and 11314041G>A) that decrease the transcriptional activity of the ATG7 gene promoter (P<0.01) were identified in PD patients, but not in the controls^121^. | ATG7 mRNA expression is decreased in the SN of PD patients compared to controls^18, 122^. |
| ATP13A2 | 1p36.13 | ATP13A2 missense mutations c.35C>T (Thr12Met) and c.1597G>A (Gly533Arg) were found in Italian young onset PD patients^123^ and the missense mutation c.2236G>A (Ala746Thr) was found to be more frequent in Chinese PD patients (Relative risk=4.3, P=0.01)^124^. | -  - |
| BAK1 | 6p21.31 | - |  |
| BAX | 19q13.33 | - | PD patients show increased BAX immunoreactivity in neuromelanin containing neurons^125^ and differences in aggregation of BAX-rich inclusions^126^. |
| BDNF | 11p14.1 | The BDNF polymorphisms C270T (in familial PD)^127^ and G196A (Val66Met; cognitive impairment in PD)^128, 129^ are associated with PD. | BDNF protein expression is decreased in neurons of the SN^130^, decreased^131^ or increased^132^ in the serum and decreased^49^ or increased^133^ in the CSF of PD patients. BDNF serum levels have been shown to positively correlate with PD motor impairment^131^. BDNF mRNA is decreased in the striatum of PD patients compared to controls^48^. |
| CACNA1B | 9q34.3 | - | - |
| CACNA1C | 12p13.33 | - | The number of CACNA1C-positive stained cells in the SN is lower in PD patients than in controls^134^. |
| CACNA1D | 3p21.1 | - | CACNA1D is higher expressed in the PD SN than in controls^135^. The number of CACNA1D-positive stained cells in the SN is lower in PD patients, but their intensity is higher than in controls^134^. |
| CACNA1E | 1q25.3 | - | CACNA1E mRNA is increased in the striatum^19^ and decreased in the SN^18^ of PD patients compared to controls. |
| CACNA1F | Xp11.23 | - | - |
| CACNA1S | 1q32.1 | - | CACNA1S mRNA is increased in the striatum of PD patients compared to controls^20^. |
| CASP3 | 4q35.1 | - | CASP3 expression is increased in the SN of PD patients^125, 136^. |
| CASP9 | 1p36.21 | - | CASP9 protein is activated in the SN of PD brains^137^ and immunologically detectable within TH+ SN neurons of late-onset sporadic PD patients^138^. |
| CAV1 | 7q31.2 | Motif lengths in CAV1 haplotypes that increase CAV1 expression were found in PD patients, but not in controls (P<0.002)^139^. | CAV1 mRNA is increased in the SN^18^ and striatum^42^ of PD patients compared to controls. |
| CCL5 | 17q12 | - | CCL5 protein expression is increased in the circulation of PD patients (P<0.001)^140-142^. CCL5 is alternatively spliced in the blood of PD patients compared to controls^75^. |
| CD163 | 12p13.31 | - | The number of CD163-positive microglia is increased in the SN of PD patients^143^. CD163 mRNA is increased in the striatum^20^ and the blood^24^ of PD patients compared to controls. |
| CD200R1 | 3q13.2 | - | Induction of CD200R expression on monocyte-derived macrophages is reduced in PD patients^144^. |
| CDH2 | 18q12.1 | - | CDH2 protein is increased in the striatum of PD patients compared to controls^20^. |
| COMT | 22q11.21 | The COMT missense variant rs4680 (Val158Met) lowers enzyme activity and is associated with an increased risk of PD (AA compared to GG genotype; OR = 1.86, P=0.044)^145^ and associated with age of onset in males (P=0.007)^146^. | COMT mRNA is decreased in the blood of PD patients compared to controls^15^. |
| CRH | 8q13.1 | - | - |
| CTNNB1 | 3p22.1 | - | - |
| CTSD | 11p15.5 | - | CTSD protein is lower expressed in PD SN neurons^33^, but is increased in the CSF of PD patients^16^ compared to controls. |
| CXCL12 | 10q11.21 | - | CXCL12 protein expression is increased in the SN of PD patients^79^ and CXCL12 mRNA expression is increased in the striatum^42^ and blood of PD patients^24^ compared to controls. |
| CYCS | 7p15.2 | - | CYCS mRNA is decreased in the SN of PD patients compared to controls^23^. |
| DDIT4 | 10q22.1 | - | DDIT4 protein expression is increased in the SN of PD patients^147^ and DDIT4 mRNA is increased^46^ or decreased^23^ in the SN and increased^20, 46^ in the striatum and BA9^46^ of PD patients compared to controls. |
| DEPTOR | 8q24.12 | - (Part of the mTORC1- and mTORC2-complex). | - |
| DNAJC13 | 3q22.1 | Missense variant rs387907571 (Asn855Ser) in the DNAJC13 gene increases late-onset PD^148^. | DNAJC13 mRNA is decreased in the blood of PD patients compared to controls^15^. |
| DNAJC6 | 1p31.3 | The splice acceptor variant rs398122404 (c.801-2A>G) in DNAJC6, decreases its expression and is associated with juvenile parkinsonism^149^. | DNAJC6 mRNA is decreased in the SN of PD patients compared to controls^23^. |
| DNM2 | 19p13.2 | - | DNM2 mRNA is increased in the SN of PD patients compared to controls^18^. |
| DRD2 | 11q23.2 | The DRD2 Taq1A SNP (rs1800497) is associated with PD (P=0.025; OR=2.2)^150^. Among non-Hispanic whites, homozygous carriers of this SNP have an increased risk of PD compared to homozygous wildtype carriers (OR=1.5), whereas African-Americans, showed an inverse association with PD risk (OR=0.10)^151^. | DRD2 mRNA is decreased in the SN of PD patients compared to controls^18, 20^. |
| DRD3 | 3q13.31 | In white Hispanics homozygeous carriers of the DRD3 missense variant rs6280 (Ser9Gly) were associated with a decreased risk of PD (OR=0.4)^151^. | - |
| E2F1 | 20q11.22 | - | DA SN neurons show high cytoplasmic E2F1 protein expression compared to controls^152^. |
| EIF2A | 3q25.1 | - | - |
| EIF4E | 4q23 | - | - |
| EIF4G1 | 3q27.1 | The c.3614G>A (Arg1205His) mutation in the EIF4G1 gene segregates with disease in families with PD and the mutations c.1505C>T (p.Ala502Val) and c.2056G>T (p.Gly686Cys) were found in PD patients but not in controls. The c.3614G>A and c.1505C>T mutations impair EIF4G1 protein function^153^. | - |
| FBXO7 | 22q12.3 | The homozygous mutation c.1132C>G (Arg378Gly) in FBXO7 is associated with parkinsonian-pyramidal syndrome^154^. In an Italian family a FBXO7 homozygous truncating mutation (Arg498Stop) was found in an Italian family, while in a Dutch family a splice-site mutation (IVS7 + 1G/T) and a missense mutation (Thr22Met) were associated with early-onset parkinsonian-pyramidal syndrome^155^. | FBXO7 mRNA is decreased in the SN of female PD patients compared to female controls^23^. |
| FOXO1 | 13q14.11 | - | FOXO1 expression is increased in the BA9 of PD patients compared to controls^51^. |
| GDNF | 5p13.2 | - | GDNF expression is decreased in neurons of the SN of PD patients (P<0.0001)^130^. |
| GPR37 | 7q31.33 | - | GPR37 accumulates in PD lewy bodies^156^ and in the brains of juvenile Parkinsonism patients, and may cause DA neuron death^157^. GPR37 mRNA is increased in the SN of female PD patients compared to female controls^21^. |
| GSK3B | 3q13.33 | The GSK3B polymorphisms rs334558 and rs6438552 are associated to PD and can alter GSK3B transcription and splicing respectively^158^. | GSK3B protein is localized in lewy bodies^159^ and the active form of GSK3B (phosphorylated on Tyr216) is elevated in the striatum of PD patients compared to controls^160^. GSK3B mRNA is decreased in the SN of PD patients compared to controls^18^. |
| HBA1 | 16p13.3 | - (Part of the hemoglobin complex). | After adjustment for age and concomitant risk factors, PD incidence was increased from 10.3 to 34.9/10,000 person-years as Hb increased from <14 to ≥16 g/dL (p=0.022)^161^. The SN of PD brains shows that the few remaining nigral neurons have increased quantity of HBA containing mitochondria compared to controls, however, reduced mitochondrial/cytoplasmic ratios are suggested for HBA (P=0.06)^162^. HBA1 mRNA is decreased in the SN^23^ and HBA1 protein is decreased in the striatum^20^, but increased in the CSF^16^ of PD patients compared to controls. |
| HBA2 | 16p13.3 | - (Part of the hemoglobin complex). | HBA2 mRNA is decreased in the blood^15^ and HBA2 protein is increased in the CSF^16^ of PD patients compared to controls. |
| HBB | 11p15.4 | - (Part of the hemoglobin complex). | After adjustment for age and concomitant risk factors, PD incidence was increased from 10.3 to 34.9/10,000 person-years as Hb increased from <14 to ≥16 g/dL (p=0.022)^161^. The SN of PD brains shows that the few remaining nigral neurons have increased quantity of HBA containing mitochondria compared to controls, however, mitochondrial/cytoplasmic ratios are reduced for HBB in PD SN neurons (P=0.038)^162^. HBB protein is increased^108^ or decreased in the SN^20^ and decreased in the striatum^20^ of PD patients compared to controls. HBB protein is increased in the SN^135^ and blood^163^ and increased^16^ or decreased in the CSF^164^ of PD patients compared to controls. |
| HBD | 11p15.4 | - (Part of the hemoglobin complex). | HBD mRNA is decreased in the striatum^20^ and blood^165^ of PD patients compared to controls. HBD protein is decreased in the striatum of PD patients compared to controls^20^. |
| HBE1 | 11p15.4 | - (Part of the hemoglobin complex). | - |
| HBG1 | 11p15.4 | - (Part of the hemoglobin complex). | HBG1 mRNA is decreased in the SN^20^ and the blood^24^ of PD patients compared to controls. |
| HBG2 | 11p15.4 | - (Part of the hemoglobin complex). | - |
| HBM | 16p13.3 | - (Part of the hemoglobin complex). | HBM mRNA is decreased in the striatum^20^ and blood^24, 165^ of PD patients compared to controls. |
| HBQ1 | 16p13.3 | - (Part of the hemoglobin complex). | HBQ1 mRNA is decreased in the SN of PD patients compared to controls^18^. |
| HBZ | 16p13.3 | - (Part of the hemoglobin complex). | - |
| HIP1R | 12q24.31 | The SNP rs10847864 in HIP1R is associated with an increased risk for PD (OR=1.15; P=9.06E-07)^77^. | HIP1R mRNA is increased in the SN of PD patients compared to controls^17^. |
| HK2 | 2p12 | - | HK2 mRNA is increased in the striatum of PD patients compared to controls^20^. |
| HMOX1 | 22q12.3 | - | Lewy bodies in PD SN neurons exhibit intense HMOX1 staining in their peripheries^166^ and the number of HMOX1-positive astroglia is increased in the PD SN^166^. Further, HMOX1 is increased in the serum of PD patients^167^. |
| HMOX2 | 16p13.3 | The homozygous G/G genotype of SNP rs2270363 in HMOX2 is associated with PD (OR=1.38, P=0.015)^168^. | - |
| HP | 16q22.2 | The Hp 2-1 genotype is associated with increased PD risk in female, moreover in never-smokers (adjusted for gender) the Hp 2-1 and Hp 1-1 genotypes were associated with an increased PD risk (OR=1.79 and 1.62 respectively) (P=0.034)^169^. | HP protein is increased in the striatum^20^, decreased in the blood^66, 170^ and decreased^118^ or increased^16, 111^ in the CSF of PD patients compared to controls. |
| HSPA5 | 9q33.3 | - | HSPA5 mRNA and protein are increased in the SN^108^ and striatum^20^ respectively of PD patients compared to controls. |
| HSPA8 | 11q24.1 | - | HSPA8 mRNA is decreased in the (male) SN^22, 23, 69^ and blood^75^ of PD patients compared to controls. HSPA8 protein is increased in the striatum^20^ and decreased in the SN^171^ and CSF^16^ of PD patients compared to controls. |
| HTRA2 | 2p13.1 | The loss of function mutation c.1195G>A (Gly399Ser) in HTRA2 was identified in PD patients and another mutation c.421G>T (Ala141Ser) was associated with PD risk in Germans (OR=2.15, P=0.039)^172^. The loss of function mutation c.1210C>T (Arg404Trp) in HTRA2 and six patient-specific variants that might affect HTRA2 expression were identified in Belgians^173^. The c.427C>G (Pro143Ala) mutation in HTRA2 induces its hyperphosphorylation in mitochondria and is associated to PD susceptibility in Taiwanese (Relative risk=2.3; P = 0.04)^174^. In Han Chinese the HTRA2 IVS5+29T>A variant may be a risk factor for PD (OR=7.53, P<0.05)^175^. | HTRA2 is a component of LB in the PD brain^172^. |
| ICAM1 | 19p13.2 | - | ICAM1 expression is increased in astroglia cells in the SN of PD patients^176^ and activated, ICAM1 positive, microglia are increased in the SN and striatum of PD patients^50^. Plasma ICAM1 protein levels are increased in stage 1 and 2, but not stage 3 and 4 PD patients compared to controls^177^. |
| IFNG | 12q15 | - | IFNG protein serum levels^178, 179^ and IFNG mRNA^180^ are increased in the blood of PD patients compared to controls. |
| IL10 | 1q32.1 | - | IL10 is increased in the serum PD patients^179, 181^, whereas in the SN of PD patients IL10 mRNA is decreased (P<0.01)^182^. |
| IL12A | 3q24.33 | - (Part of the IL12-complex). | IL12A mRNA is increased in the blood of PD patients compared to controls^15^. |
| IL12B | 5q33.3 | - (Part of the IL12-complex). | - |
| IL1B | 2q14.1 | The IL1B (-511) allele (rs16944) is associated with PD in Finish patients^183^. | IL1B protein is increased in the CSF^184, 185^ and peripheral blood mononuclear cells^141^, whereas IL1B mRNA is decreased in the SN^20, 182^ and increased in the striatum^20^ of PD patients compared to controls. |
| IL2 | 4q27 | - | IL2 is increased in the serum and CSF of PD patients^179, 185, 186^. |
| IL4 | 5q31.1 | - | IL4 is increased in the serum and CSF of PD patients^179, 185^. |
| IL5 | 5q31.1 | - | - |
| IL6 | 7p15.3 | The SNP rs1800795 in the promotor of IL6 is associated with PD in Ashkenazi Jews and caucasians^187^. | IL6 is increased in the serum and CSF in PD patients^179, 184^, whereas in the SN of PD patients IL6 mRNA is decreased (P<0.01)^182^. |
| IL8 | 4q13.3 | Different genotypes of the SNP c.251A>T in the gene promotor of IL8 are associated with PD in Irish patients^188^. | IL8 is increased in the blood of PD patients^141^. |
| INS | 11p15.5 | - | The autoimmune reaction towards serum insulin (INS) is increased by 70% in PD patients^189^. |
| INSR | 12p13.2 | - | INSR protein and mRNA is decreased in the PD SN^190, 191^ and INSR protein is increased in the CSF of PD patients^16^ compared to controls. |
| ITGB2 | 21q22.3 | - (Part of the LFA-1 complex). | ITGB2 mRNA expression is increased in the angular cortex in PD from Braak stage 3 onwards^182^ and is increased in the striatum of PD patients compared to controls^20^. |
| JAK2 | 9p24.1 | - | - |
| LAMP2 | Xq24 | - | PD patients show a decreased LAMP2 protein expression in the SN^171^, in peripheral leukocytes^192^ and in CSF^16^ compared to controls, whereas LAMP2 mRNA is increased in the SN^18^ and blood^15^ and decreased in peripheral leukocytes^192^ of PD patients compared to controls. |
| LDLR | 19p13.2 | - | - |
| LMX1A | 1q23.3 | - | LMX1A mRNA is decreased in the SN of PD patients compared to controls^20^. |
| LMX1B | 9q33.3 | - | LMX1B protein is decreased in midbrain DA neurons of PD patients compared to controls (P<0.0005)^193^. |
| LRP1 | 12q13.3 | - | LRP1 expression in melanized neurons of the SN is increased early in PD^117^. |
| LRPPRC | 2p21 | - | LRPPRC mRNA is decreased in the SN of PD patients compared to controls^17, 22, 23^. |
| LXRA | 11p11.2 | - (LXR isoform). | - |
| LXRB | 19q13.33 | - (LXR isoform). | - |
| MAOA | Xp11.3 | The variation c.941T>G (rs1799835) in MAOA is associated with early onset PD^194^ and MAOA variations seem to be more associated with the male gender in PD patients^194-196^. | MAOA mRNA is increased in the SN of PD patients compared to controls^122^. |
| MAOB | Xp11.3 | A GT repeat variation (> or =188 bp) in MAOB is associated with PD (OR=4.60, P<0.00005)^197^. The synonymous SNP rs1799836 (G>A) in MAOB is associated with PD in females^198^, but also with an overal increased PD risk (AA vs AG/GG OR=1.70, P=0.016)^145^. | MAOB mRNA is increased in the striatum of PD patients compared to controls^19, 20^. |
| MAP3K7 | 6q15 | - | - |
| MAPKAP1 | 9q33.3 | - (Part of the mTORC2-complex). | - |
| MARK2 | 11q13.1 | - | - |
| MC1R | 16q24.3 | - | MC1R mRNA is decreased in the SN of male PD patients compared to controls^23^. MC1R protein is upregulated in the striatum of PD patients compared to controls^20^. |
| MITF | 3p13 | - | - |
| MLST8 | 16p13.3 | - (Part of the mTORC1- and mTORC2-complex). | - |
| MTOR | 1p36.22 | - (Part of the mTORC1- and mTORC2-complex). | MTOR protein is increased in the CSF of PD patients compared to controls^16^. |
| NDEL1 | 17p13.1 | - | - |
| NEDD4 | 15q21.3 | - | - |
| NEDD8 | 14q12 | - | NEDD8 increases PARK2 activity and stabilizes PINK1 and its accumulation is observed in LB in DA neurons in the SN of PD patients^199, 200^. |
| NEUROG2 | 4q25 | - | - |
| NFKB1 | 4q24 | - (Part of the NF-KB complex). | NFKB1 mRNA is increased in the SN of PD patients compared to controls^69^. NFKB levels are elevated in the striatum (P<0.05) and SN of PD patients (P<0.01)^201^. |
| NFKB2 | 10q24.32 | - (Part of the NF-KB complex). | NFKB levels are elevated in the striatum (P<0.05) and SN of PD patients (P<0.01)^201^. |
| NOS1 | 12q24.22 | The SNP in exon 29 of NOS1 is associated with PD (OR for T allele carriers=1.53, P=0.02)^202^. Further, the SNPs rs3782218, rs11068447, rs7295972, rs2293052, rs12829185, rs1047735, rs3741475, and rs2682826 in NOS1 were associated with early-onset PD families (range of P=0.00083-0.046)^203^. | - |
| NOS2 | 17q11.2 | The SNP in exon 22 of NOS2 is associated with PD (OR for AA carriers=0.50, P=0.01)^202^. Further, the SNPs rs2072324, rs944725, rs12944039, rs2248814, rs2297516, rs1060826, and rs2255929 in NOS2 were associated with early-onset PD families (range of P=0.0000040-0.047)^203^. | - |
| NR4A2 | 2q24.1 | Homozygous insertion of a single nucleotide (7048G7049) in intron 6 of the NR4A2 gene is associated with PD (OR=8.4, P<0.005)^204^. The 3G/2G genotype of the IVS6+ 18insG polymorphism is associated with early-onset PD (OR=1.91, P=0.011)^205^. Three variations in the 5UTR of NR4A2 (c.-309C>T, c.-291Tdel, c.-245T>G) are associated with PD and decrease NR4A2 expression^206, 207^. | NR4A2 mRNA is decreased in the SN^17, 18, 20^ and peripheral blood lymphocytes^208, 209^ of PD patients compared to controls. NR4A2 protein is decreased in SNCA-positive nigral neurons of PD patients compared to controls^210^. |
| NR5A1 | 9q33.3 | - | - |
| PARK7 | 1p36.23 | Exon deletions and duplications and a c.497T>C (Leu166Pro) mutation of the PARK7 gene are associated with autosomal recessive early-onset PD ^211, 212^. | PARK7 mRNA is decreased in (male) PD patients compared to controls^22, 23^. PARK7 protein is decreased in CSF of PD patients compared to controls^16^. |
| PINK1 | 1p36.12 | Exon deletions and multiple mutations in PINK1 are associated with early-onset PD^213-217^. | PINK1 mRNA is decreased in the SN of PD patients compared to controls^22, 23^. |
| PITX3 | 10q24.32 | The PITX3 promoter SNP rs3758549 (C>T) is associated with PD susceptibility (OR=1.42, P=0.004)^218^. In a meta-analysis the A allele of the SNP rs4919621 was significantly associated with increased risk of PD in a Caucasian population (OR=1.15, P=0.04) and both the C allele of rs2281983 (OR=1.62, P=0.0001) and the A allele of rs4919621 (OR=1.70, P<0.0001) are associated with early-onset PD^219^. Another meta-analysis showed association of the PITX3 SNP rs3758549 with PD risk (OR=1.21, P=0.019) and early-onset PD in an Asian population (OR=1.44, P=0.004)^220^. The T allele of the SNP rs3758549 is associated with PD in the Asian population (P=0.019) and early-onset PD (P=0.004)^220^. | PITX3 mRNA is decreased in the SN^18^ and peripheral blood lymphocytes^209^ of PD patients compared to controls. |
| PLA2G6 | 22q13.1 | The mutations c.2222G>A (Arg741Gln) and c.2239C>T (Arg747Trp) in the PLA2G6 gene are associated with dystonia-parkinsonism^221^. The nonsyn SNPs c.2339A>G (Asn780Ser) and c.2341G>A (Ala781Thr) were found in patients with sporadic early-onset PD but not in controls^222^. The SNP c.1959T>A, two nonsyn c.1966C>G (Leu656Val) and c.2077C>G (Leu693Val) and a frameshift (His597fx69) were identified in Chines PD patients but not in controls. The two nonsyn SNPs and the frameshift reduce PLA2G6 enzymatic activity^223^. | - |
| PLAT | 8p11.21 | - | - |
| PLAU | 10q22.2 | - | - |
| PLAUR | 19q13.31 | - | - |
| PLG | 6q26 | - (Proenzym of plasmin). | PLG protein is increased in the blood of PD patients compared to controls^66^. |
| PPP1CA | 11q13.2 | - (Part of the PP1-complex). | - |
| PPP1CB | 2p23.2 | - (Part of the PP1-complex). | - |
| PPP1CC | 12q24.11 | - (Part of the PP1-complex). | - |
| PPP1R12A | 12q21.31 | - (Part of the PP1-complex). | PPP1R12A is alternatively spliced in the blood of PD patients compared to controls^75^. PPP1R12A protein is decreased in the CSF of PD patients^224^. |
| PRKAA2 | 1p32.2 | - (Part of the AMPK-complex). | - |
| PRKAB1 | 12q24.23 | - (Part of the AMPK-complex). | - |
| PRKAB2 | 1q21.1 | - (Part of the AMPK-complex). | - |
| PRKAG1 | 12q13.12 | - (Part of the AMPK-complex). | - |
| PRKAG2 | 7q36.1 | - (Part of the AMPK-complex). | PRKAG2 mRNA is increased in the SN of female PD patients compared to controls^21^. |
| PRKAG3 | 2q35 | - (Part of the AMPK-complex). | - |
| PRR5 | 22q13.31 | - (Part of the mTORC2-complex). | - |
| PSAP | 10q22.1 | - | PSAP mRNA is increased^48^ or decreased^22, 23, 69^ in the SN of PD patients compared to controls. PSAP is alternatively spliced in the blood of PD patients compared to controls^75^. |
| PSEN1 | 14q24.2 | - | PSEN1 mRNA is increased in the SN of PD patients compared to controls^18, 22, 23^. |
| PSEN2 | 1q42.13 | - | PSEN2 mRNA is decreased in the SN of PD patients compared to controls^18^. |
| PTEN | 10q23.31 | - | PTEN is alternatively spliced in the blood of PD patients compared to controls^75^. |
| RAC1 | 7p22.1 | - | RAC1 mRNA is decreased in the SN of (male) PD patients compared to controls^22, 23^. |
| REL | 2p16.1 | - (Part of the NF-KB-complex). | NFKB levels are elevated in the striatum (P<0.05) and SN of PD patients (P<0.01)^201^. |
| RELA | 11q13.1 | - (Part of the NF-KB-complex). | RELA mRNA is increased in the SN of PD patients compared to controls^18^. NFKB levels are elevated in the striatum (P<0.05) and SN of PD patients (P<0.01)^201^ and the number of NFKB (RELA) immunoreactive nuclei of DA neurons in PD patients was increased over 70-fold compared to control subjects^225^. |
| RELB | 19q13.32 | - (Part of the NF-KB-complex). | NFKB levels are elevated in the striatum (P<0.05) and SN of PD patients (P<0.01)^201^. |
| RFX3 | 9p24.2 | - | RFX3 mRNA is increased in the striatum of PD patients compared to controls^20^. |
| RGMA | 15q26.1 | - | RGMA mRNA is increased in the SN^17, 69^ and striatum^20^ of PD patients compared to controls. |
| RHOA | 3p21.31 | - | RHOA is alternatively spliced in the blood of PD patients compared to controls^75^. |
| RICTOR | 5p13.1 | - (Part of the mTORC2-complex). | - |
| RPTOR | 17q25.3 | - (Part of the mTORC1-complex). | - |
| SCARB1 | 12q24.31 | - | - |
| SERPINB9 | 6p25.2 | - | SERPINB9 mRNA is decreased in the blood of PD patients compared to controls^15^. |
| SERPINE1 | 7q22.1 | - | SERPINE1 mRNA is increased in the SN of PD patients compared to controls^20^. |
| SIRT1 | 10q21.3 | Three novel heterozygous sequence variants in the SIRT1 promotor, g.69644133C>G, g.69644213G>A and g.69644351G>A were identified in PD patients, but not in controls and may reduce SIRT1 expression^226^. | - |
| SIRT7 | 17q25.3 | - | SIRT7 mRNA is increased in the SN of PD patients compared to controls^18^. |
| SLC11A2 | 12q13.12 | The C allele of rs150909 in DMT1 (SLC11A2) is associated with 3.09 (95% CI 0.13-6.06) years older age at PD diagnosis (P=0.03)^227^. | The DMT1+IRE isoforms are upregulated (P<0.01) whereas the DMT1-IRE isoforms are downregulated in PD SNpc compared to controls (P<0.01)^228^. |
| SLC18A2 | 10q25.3 | Gain of function haplotypes in the SLC18A2 promotor are associated with lower PD risk in females (homozygeous vs wt OR=0.38, P=0.01)^229^. The SNP rs363371 in the SLC18A2 promotor is associated with lower PD risk in Italians (dominant model OR=0.72, P=0.03)^230^. | SLC18A2 mRNA is decreased in the SN of PD patients compared to controls^17, 18, 20^. |
| SLC40A1 | 2q32.2 | - | SLC40A1 (Ferroportin) is alternatively spliced in the blood of PD patients compared to controls^75^ and SLC40A1 protein is 1.4 fold increased in PD SN compared to controls (P<0.05)^231^. |
| SLC6A3 | 5p15.33 | The 11-copy allele of a variable number tandem repeat polymorphism in SLC6A3 was more frequent present in PD (OR=4.08, P=0.008^232^; OR=2.5, P<0.02^233^). The 10-copy allele of this polymorphism is neuroprotective in East Asians (OR=0.78, P=0.009)^234^, whereas the SLC6A3 promoter SNP rs2652510 (A>G) is associated with increased PD risk in Caucasians (OR=1.26, P=0.018)^234^. | SLC6A3 mRNA is decreased in the SN of PD patients compared to controls^18, 20, 48^. |
| SLIT2 | 4p15.31 | - | SLIT2 is decreased in the SN^18, 44^ and increased in the blood^24^ of PD patients compared to controls. |
| SMAD4 | 18q21.2 | - | SMAD4 mRNA is increased in the blood of PD patients compared to controls^15^. |
| SMPD1 | 11p15.4 | The SMPD1 mutations, Arg591Cys (OR=not applicable, P=0.009)^235^, Pro533Leu (OR=1.76, P=0.047)^235^ and Leu302Pro (OR=9.4, P<0.0001)^236^ are associated with an increased PD risk. | - |
| SOD2 | 6q25.3 | - | SOD2 protein levels are lower in the SN (P<0.05) and striatum (P<0.05) of PD patients, but its activity is nevertheless elevated in the straitum (P<0.05)^237^. SOD2 immunoreactivity is affected in the lateral, but not in the medial and central part of the SN of PD patients^238^. SOD2 protein is increased in the SN^25^ and decreased in the CSF of PD patients compared to controls^16^. Reactive dopamine quinones reduce enzymatic activity of SOD2 up to 50% by promoting its aggregation^239^. |
| SOX2 | 3q26.33 | - | SOX2 mRNA is increased in the SN of PD patients compared to controls^17, 46^. |
| SPHK1 | 17q25.1 | - | SPHK1 mRNA is increased in the striatum of PD patients compared to controls^20^. |
| STAR | 8p11.23 | - (Part of the mPTP-complex). | - |
| STAT3 | 17q21.2 | - | - |
| STK11 | 19p13.3 | - | - |
| SYNJ1 | 21q22.11 | Mutation c.773G>A (Arg258Gln) in the SYNJ1 gene is associated with autosomal recessive, early-onset Parkinsonism in an Italian^240^ and Iranian family^241^. | SYNJ1 mRNA is decreased in the SN of PD patients compared to controls^18^. |
| SYT11 | 1q22 | A meta-analysis on GWAS data identified SYT11 (chr1:154105678) as a new risk locus for PD (OR=1.67, P=5,70E-09)^242^ and confirmed by another meta-analysis (OR=1,73, P=2.35E-12)^27^ and replicated in a Caucasian (OR=1,43, P=0,001)^77^ and a Scandinavian PD group (OR=1,46, P=0.011)^104^. | SYT11 mRNA is decreased in the SN of PD patients compared to controls^108^. |
| TF | 3q22.1 | The AT haplotype of the SNPs rs1880669/rs1049296 in transferrin (TF) is protective for PD (OR=0.83, 95% CI: 0.71-0.96) (P=0.01)^243^. | TF mRNA is increased in the SN^46, 47^ and CSF^16, 164^, but decreased in the blood^66^ of PD patients compared to controls. Also, there is increased oxidized TF in the PD SN^244^. |
| TFR2 | 7q22.1 | - | The number of [125I]-Tf(Fe)2 binding sites, as a measure for the number of TFRs, was lower on the perikarya of melanized neurons in the PD SNpc compared to controls (P=0.01)^245^. TFR2 protein expression is highly specific for DA neurons in the SNpc^244^. |
| TGFBR2 | 3p24.1 | - | TGFBR2 mRNA is decreased in the SN of PD patients compared to controls^21^. |
| TH | 11p15.5 | - | TH mRNA is decreased in the SN of PD patients compared to controls^18, 20^. TH protein and mRNA is reduced in the SN of PD patients, but protein:mRNA ratio is unaffected^246^. |
| TNF | 6p21.33 | The homozygous genotype of the c.1031T>C mutation in the TNF promotor is associated with PD (OR=3.5, P=0.032^247^; OR=2.96, P=0.0085^248^) and even stronger associated with early-onset PD (OR=5.0, P=0.0039)^247^. Also the homozygous mutation of c.308G>A in the TNF promotor is associated with early-onset PD (OR=1.86, P=0.0037)^249^. | TNF protein is higher expressed in the striatum and CSF of PD patients^250^, whereas its mRNA is lower expressed in the PD SN (P<0.001) and striatum (P<0.05) of PD patients^182^. Furthermore, TNF protein is increased in the serum^179^ and peripheral blood mononuclear cells^141^ of PD patients. |
| TP53 | 17p13.1 | - | TP53 protein expression is higher in the caudate nucleus, but not in the SN of PD patients (P<0.05)^201^, however, an increase in (Ser15-)phosphorylated TP53 in the PD SN was shown (P<0.001)^251^. |
| TRAF2 | 9q34.3 | - | - |
| TSPO | 22q13.2 | - | Increased TSPO expression (measured with positron emission tomography imaging) in the midbrain and striatum of PD patients correlates with PD motor severity^252^. |
| TYR | 11q14.3 | - | - |
| UCHL1 | 4p13 | A dominant mutation (Ile93Met) in UCHL1 is identified in a German PD family^253^ and a heterozygeous c.54C>A (Ser18Tyr) mutation is associated with a decreased PD risk (OR=0.55, P<0.05, 10563640; OR=0.54, P=0.033^253^; OR=0.66, P=0.02^254^). | UCHL1 mRNA is decreased in the SN^22, 23, 255^ and striatum^48^ of PD patients compared to controls. UCHL1 protein is increased in the striatum of PD patients compared to controls^20^. |
| VDAC1 | 5q31.1 | - | VDAC1 mRNA is decreased in the blood of PD patients^15^, and VDAC1 protein immunoreactivity is decreased in SN PD NM-positive neurons (and significantly greater in neurons with SNCA inclusions) compared to controls^256^. |
| VDAC2 | 10q22.2 | - | VDAC2 mRNA is decreased in the SN^69^, and VDAC2 protein is increased in the striatum^20^ of PD patients compared to controls. |
| VDAC3 | 8p11.21 | - | VDAC3 protein is increased in the striatum of PD patients compared to controls^20^. |
| VDR | 12q13.11 | The frequency of the C allele of the Fokl C/T polymorphism (rs10735810) was significantly higher in PD patients than in controls (OR=1.34, P=0.024)^257^; OR=1.615, P=0.017^258^) and also a genotype with the C allele was more frequent in patients (OR=2.164, P=0.004^257^; OR=2.677, P=0.015^258^). The intronic SNPs rs4334089 and rs7299460 in VDR were associated with PD age of onset (P= 0.0008 and 0.0016 respectively)^259^. | - |
| VLDLR | 9p24.2 | - | - |
| VPS35 | 16q11.2 | The c.1858G>A (Asp620Asn) mutation in VPS35 was found to co-segragate with PD in multiple families^260, 261^. | VPS35 mRNA is decreased in the SN of (male) PD patients compared to controls^23, 108^. |

[1] Expression data from genome wide expression studies in PD-associated brain areas (SN, striatum, BA9), CSF or blood were included if they met the following criteria; *with* an adjusted p-value for multiple comparisons <0.05, mRNAs should have a fold change of <-1.5 or >1.5 and proteins <-1.2 or >1.2, and *without* a published adjusted p-value, mRNAs and proteins were only included with a p-value <0.05 and a fold change of <-2.0 or >2.0 and of <-1.5 or >1.5 respectively.

| **Supplementary Table 5.** **Landscape proteins, the main process(es) they are functionally involved in and their location within the figures.** For each protein (complex) in the landscape, the process(es) oxidative stress response (A), endosomal-lysosomal functioning (B), endoplasmic reticulum (ER) stress response (C), and neuron death and immune response (D) in which they exert their main effect is indicated in columns A to D. The location of each protein (complex) in the landscape is indicated in the column ‘Location coordinates in Figures S2 and S3’ with these coordinates corresponding to the location of the protein (complex) in Figure S2 and/or S3. A total of 113, 152, 131 and 208 proteins were assigned to processes A, B, C and D, respectively. Between parentheses, protein complex subunits (csu) or protein isoforms (iso) that function within the landscape are indicated. NS; not shown. | | | | | | | | | | | | |
| --- | --- | --- | --- | --- | --- | --- | --- | --- | --- | --- | --- | --- |
| **Protein (csu/iso)** | **A** | **B** | **C** | **D** | **Location coordinates in Figures S2 and S3** |  | **Protein (csu/iso)** | **A** | **B** | **C** | **D** | **Location coordinates in Figures S2 and S3** |
| 26S (PSMD11) |  |  | X | X | **S2**-3d |  | BAT2 |  |  | X |  | **S2**-3d, 4d |
| ABCA1 | X | X | X |  | **S2**-1a, 3a, 3c, 3d |  | BAT5 | X |  |  |  | **S2**-1d |
| ABCA3 |  | X |  |  | **S2**-3b |  | BAX | X |  | X |  | **S2**-1c, 2c, 3d |
| ABCA5 |  | X |  |  | **S2**-3b |  | BDNF | X |  |  | X | **S2**-1b; **S3**-2b, 3a, 3c, 4a, 5d |
| ABCG1 | X | X | X | X | **S2**-1a, 3a, 3b, 3c; **S3**-4b |  | BMP7 | X | X | X | X | **S2**-2d; **S3**-2b, 3c, 3d, 5c |
| ACMSD | X |  |  |  | NS |  | BST1 |  |  |  | X | **S3**-1b |
| ACSL6 |  |  | X |  | **S2**-1c |  | BTNL2 |  |  |  | X | **S3**-2c |
| ACTN4 |  | X | X | X | **S2**-3c, 3d, 4d; **S3**-2d |  | C9 |  |  |  | X | **S3**-3d |
| ADAM12 |  |  |  | X | **S2**-5b |  | CACNA1A | X |  |  |  | **S2**-1a, 2a, 5b; **S3**-1c |
| ADAMTS14 |  |  |  | X | **S2**-5b |  | CACNA1B | X |  |  |  | **S2**-1a |
| ADAMTS2 |  |  |  | X | **S2**-5b |  | CACNA1C | X |  |  |  | NS |
| ADH1C |  |  | X |  | **S2**-4d |  | CACNA1D | X |  |  |  | NS |
| AGAP1 | X | X |  |  | **S2**-2a |  | CACNA1E | X |  |  |  | NS |
| AGTR1 | X | X |  | X | **S2**-1b, 3a, 5a, 5b; **S3**-3d, 4d |  | CACNA1F | X |  |  |  | NS |
| AICDA |  |  |  | X | NS |  | CACNA1S | X |  |  |  | NS |
| AKT1 | X | X | X | X | **S2**-2c, 2d, 3b, 4c, 4d |  | CACNA2D3 | X |  |  |  | **S2**-1a, 5c; **S3**-1c |
| AMIGO2 | X |  |  | X | **S2**-2a |  | CASP3 | X |  | X | X | **S2**-2b, 2c, 2d, 5a |
| AMPH |  | X |  |  | **S2**-2a, 2b |  | CASP9 | X |  | X | X | **S2**-2b, 3d |
| AMPK (PRKAA1, PRKAA2, PRKAB1, PRKAB2, PRKAG1, PRKAG2, PRKAG3) | X | X |  |  | **S2**-1b, 1d, 2a, 2c, 3a, 3d |  | CAV1 |  | X | X |  | **S2**-2a, 2b, 3a |
| ANGPT2 | X | X |  | X | **S2**-1b, 5b; **S3**-3a, 3b, 3d, 4d, 5b |  | CCAR2 |  |  | X | X | **S2**-2c, 3c, 4a, 4c, 4d |
| AP3 (AP3B1) |  | X |  |  | **S2**-2a, 3b |  | CCL5 |  |  |  | X | **S3**-2b, 3b, 3c, 4b |
| APOE |  | X | X |  | **S2**-2a, 2d |  | CCR4 |  |  |  | X | **S3**-2b, 3b, 4b |
| APP |  |  | X |  | NS |  | CD163 | X |  |  |  | **S2**-1a |
| AR |  |  | X |  | **S2**-2d, 4c, 5c; **S3**-2c |  | CD200 |  |  |  | X | **S3**-3c, 4d |
| ARHGAP33 |  | X |  |  | **S2**-3a |  | CD200R1 |  |  |  | X | **S3**-3c, 4d |
| ARHGAP44 |  | X |  |  | **S2**-3a |  | CDH2 |  |  |  | X | **S2**-1d |
| ARL4A |  |  | X |  | NS |  | CDH23 |  |  |  | X | **S2**-4a |
| ARMC8 |  |  | X |  | NS |  | CDH6 |  |  |  | X | **S2**-4d |
| ASAH1 |  | X |  |  | **S2**-3b, 4b |  | CDK19 |  |  | X |  | **S2**-4c |
| ASCL1 |  |  |  | X | **S2**-5c |  | CECR2 | X |  |  |  | **S2**-4d |
| ATF6 |  |  | X | X | **S2**-3c, 4c; **S3**-2a |  | CENPC |  |  |  | X | **S2**-4c |
| ATG5 | X | X |  |  | **S2**-2b, 5a |  | CEP85L |  | X |  |  | **S2**-1d |
| ATG7 | X | X |  | X | **S2**-2b |  | CLVS2 |  | X |  |  | **S2**-2a |
| ATP13A2 | X | X |  | X | **S2**-3b; **S3**-3c |  | CNNM2 | X |  |  |  | **S2**-1a |
| ATP2B2 | X |  | X |  | **S2**-1d |  | CNTN1 |  |  |  | X | **S2**-5a |
| ATP6V0A1 |  | X |  |  | **S2**-3b, 4b |  | CNTNAP2 |  |  |  | X | **S3**-3d |
| ATR |  |  | X | X | **S2**-3c, 4c |  | COL18A1 | X | X | X | X | **S2**-2a, 4d; **S3**-1d, 3b, 3d, 4c, 5d |
| AXIN1 |  |  | X |  | **S2**-2a, 3d, 4d |  | COL2A1 |  | X | X | X | **S2**-4a, 4d, 5b; **S3**-3a, 4a |
| BAG6 |  | X | X | X | **S2**-2c, 3b, 3d, 4b, 5b; **S3**-4d |  | COMT | X |  |  |  | **S2**-1b, 5b |
| BAK1 | X |  | X |  | **S2**-3d |  | CPNE4 |  |  |  | X | **S2**-4a |
| BAMBI |  | X | X | X | **S2**-3d |  | CREM | X | X | X | X | **S2**-4c; **S3**-4b |
| BANK1 |  |  |  | X | **S3**-1b |  | CRH | X |  | X | X | **S2**-2a; **S3**-2b, 5c |

| **Supplementary Table 5. (continued)** | | | | | | | | | | | | |
| --- | --- | --- | --- | --- | --- | --- | --- | --- | --- | --- | --- | --- |
| **Protein (csu/iso)** | **A** | **B** | **C** | **D** | **Location coordinates in Figures S2 and S3** |  | **Protein (csu/iso)** | **A** | **B** | **C** | **D** | **Location coordinates in Figures S2 and S3** |
| CRHBP |  |  |  | X | **S3**-2b |  | FER |  | X |  | X | **S2**-3a, 4d, 5b; **S3**-3c |
| CRHR1 | X |  | X | X | **S2**-2a, 4a; **S3**-5c |  | FGF12 | X |  |  |  | **S2**-1a |
| CSMD1 |  |  | X |  | **S2**-3d |  | FOXO1 | X | X | X | X | **S2**-1d, 2b, 2d, 4b, 4c; **S3**-3d, 4a, 4b, 4d |
| CTNNB1 |  | X | X | X | **S2**-1a, 2b, 2d, 3c, 3d, 4c, 4d, 5a |  | FPR3 | X |  |  |  | NS |
| CTSD |  | X |  |  | **S2**-3b |  | FRMD4A |  |  |  | X | **S2**-5a |
| CUL2 |  |  | X | X | **S2**-3d, 4c, 5a |  | GAK |  | X | X |  | **S2**-2a, 2d, 3a, 3b |
| CXCL12 |  |  |  | X | **S2**-5a; **S3**-1b, 2a, 3a, 3c, 4c, 5d |  | GAS2 |  |  |  | X | **S2**-5a; **S3**-3c |
| CXCR4 |  | X |  | X | **S2**-5a; **S3**-1b, 3a, 3c, 4b, 5d |  | GBA |  | X |  |  | **S2**-3b |
| CYCS | X |  |  | X | **S2**-1b, 1c, 2b, 2c |  | GCH1 | X | X |  |  | **S2**-3c; **S3**-3b |
| CYP17A1 |  |  | X |  | **S2**-3c, 3d, 5b |  | GDNF | X |  |  | X | **S2**-1b, 4a; **S3**-2a, 3c, 5c |
| DCUN1D1 |  | X | X |  | **S2**-3d, 4c |  | GJB2 |  | X |  | X | **S2**-3a, 3d, 4b, 4d |
| DDIT4 |  | X |  | X | **S2**-2c |  | GLDN |  |  |  | X | **S3**-3d |
| DDX3X |  |  |  | X | **S2**-4c |  | GNA12 | X | X | X | X | **S2**-1d, 3d, 4a, 4d, 5a |
| DGKQ | X |  | X |  | **S2**-1d, 2c, 3c |  | GPATCH2 |  |  | X |  | NS |
| DLG2 |  |  | X | X | **S2**-1d, 3a, 3d, 4a |  | GPR37 |  | X |  |  | **S2**-4a |
| DNAH11 |  | X |  |  | **S2**-4b |  | GSK3B | X | X | X | X | **S2**-1b, 1d, 2a, 2b, 2c, 2d, 3d, 4a, 4b, 4c, 4d, 5a, 5b, 5c |
| DNAJC13 |  | X |  |  | **S2**-2a |  | H1FX |  |  | X |  | **S2**-3c |
| DNAJC6 |  | X |  |  | **S2**-2a |  | HAVCR1 |  |  |  | X | **S3**-2c |
| DNM2 |  | X | X |  | **S2**-2a, 2b |  | Hb (HBA1, HBA2, HBB, HBD, HBE1, HBG1, HBG2, HBM, HBQ1, HBZ) | X |  |  |  | **S2**-1a |
| DRAM1 |  | X |  |  | **S2**-3b |  | HCAR1 | X |  |  |  | **S2**-2a |
| DRD2 | X | X |  | X | **S2**-2a; **S3**-5c |  | HIP1R |  | X |  |  | **S2**-3a |
| DRD3 | X |  |  |  | **S2**-3a |  | HK2 | X |  |  |  | **S2**-1c, 3b |
| DRG2 |  |  |  | X | **S3**-2c |  | HLA-B |  |  |  | X | **S3**-4b, 5b |
| DSCAM |  |  | X | X | **S2**-4d |  | HLA-C |  | X |  | X | **S3**-4b |
| E2F1 |  |  |  | X | **S2**-4c |  | HLA-DQA1 |  |  |  | X | **S3**-5b |
| EFCAB4B | X |  |  |  | **S3**-2c |  | HLA-DQA2 |  |  |  | X | **S3**-5a |
| EFCAB6 |  |  | X |  | **S2**-5b |  | HLA-DQB1 |  |  |  | X | **S3**-5b |
| EHMT2 | X |  |  | X | **S2**-4c; **S3**-3a |  | HLA-DQB2 |  |  |  | X | **S3**-5a |
| EIF2A |  |  | X |  | NS |  | HLA-DRA |  |  |  | X | **S3**-4b, 5b |
| EIF4E |  |  | X |  | **S2**-1b, 3c |  | HLA-DRB1 |  |  |  | X | **S3**-4b |
| EIF4EBP2 |  |  | X |  | **S2**-1b, 3c, 5c |  | HLCS |  |  | X |  | **S2**-1c |
| EIF4G1 |  |  | X |  | **S2**-1b, 2c, 2d, 3c, 3d |  | HLX |  |  |  | X | **S3**-1b, 2c |
| ENSA |  | X | X |  | **S2**-1b |  | HMOX1 | X |  |  | X | **S2**-1a, 1b, 4b, 4d; **S3**-3a, 2a |
| ETV6 |  |  |  | X | **S2**-4b, 4c; **S3**-4d |  | HMOX2 | X |  |  |  | NS |
| FAM134C |  |  |  | X | **S2**-5c |  | HP | X |  |  |  | **S2**-1a |
| FAM190A |  | X |  |  | **S2**-3a |  | HPGD |  | X |  |  | **S2**-2a |
| FBN1 |  | X |  | X | **S3**-3b |  | HS3ST1 |  |  |  | X | **S2**-4b |
| FBXO25 |  |  | X |  | **S2**-3d |  | HSPA5 |  |  | X |  | NS |
| FBXO36 |  |  | X |  | NS |  | HSPA8 |  | X |  |  | **S2**-2a, 2b, 2d, 3b, 3c, 3d, 4a |
| FBXO7 | X |  | X |  | **S2**-1c, 3d |  | HTRA2 | X |  | X | X | **S2**-1c, 2c, 2d |

| **Supplementary Table 5. (continued)** | | | | | | | | | | | | |
| --- | --- | --- | --- | --- | --- | --- | --- | --- | --- | --- | --- | --- |
| **Protein (csu/iso)** | **A** | **B** | **C** | **D** | **Location coordinates in Figures S2 and S3** |  | **Protein (csu/iso)** | **A** | **B** | **C** | **D** | **Location coordinates in Figures S2 and S3** |
| HUS1 | X |  |  | X | **S2**-4b; **S3**-5d |  | LRRC25 |  |  |  | X | **S3**-1b |
| ICAM1 |  |  |  | X | **S3**-1b, 1d, 2a, 2b, 2c, 3a, 3b, 3c, 4c, 5b |  | LRRK2 | X | X | X | X | **S2**-1c, 1d, 2a, 2b, 2c, 3a, 3b, 3c, 3d, 4a, 4b, 4d |
| IFNG |  |  |  | X | **S3**-3b, 3c, 4c |  | LSM7 |  |  | X |  | **S2**-3c |
| IGF1 |  |  | X |  | NS |  | LTBP1 |  |  |  | X | **S3**-3b, 4d |
| IL10 |  |  |  | X | **S3**-5b |  | LXR (LXRA, LXRB) |  | X |  | X | **S3**-3a, 4b |
| IL12 (IL12A, IL12B) |  |  |  | X | **S3**-1b, 1c, 2b, 2c, 3d, 4c, 5c |  | MAOA | X |  |  |  | **S2**-2b, 3c, 5b |
| IL1B |  |  |  | X | **S3**-3b, 3c, 3d, 4b, 5b |  | MAOB | X |  |  |  | **S2**-1b |
| IL2 |  |  |  | X | **S3**-2b, 2c, 3b, 3d |  | MAP1B | X |  | X | X | **S2**-1a, 1d, 2b, 3d, 5b; **S3**-1c |
| IL2RA |  |  |  | X | **S3**-1b, 2b, 3c |  | MAP2K6 |  | X | X | X | **S2**-2d, 3a, 4c |
| IL4 |  |  |  | X | NS |  | MAP3K7 |  | X | X |  | **S2**-2d, 3a |
| IL5 |  |  |  | X | **S3**-1b, 2b, 3c, 4c |  | MAPT |  | X | X |  | **S2**-1b, 1d, 2b, 2d, 3a, 3b, 3d |
| IL5RA |  |  |  | X | **S3**-1b, 4c |  | MARC1 | X |  |  |  | **S2**-1c |
| IL6 |  |  |  | X | **S3**-3a |  | MARK2 |  | X |  | X | **S2**-1d, 2a, 5a |
| IL8 |  |  |  | X | **S3**-5c |  | MAS1 | X |  |  |  | **S2**-1b, 3a |
| INS | X | X | X | X | **S2**-1c, 1d, 2c, 2d, 3a, 3c, 4a, 5b, 5c; **S3**-2a, 2b, 3b, 4a, 4b, 4c |  | MBNL2 |  | X |  |  | **S2**-4b |
| INSR | X | X | X | X | **S2**-1d, 2d, 3a, 4b, 5b; **S3**-4a, 4b, 5c |  | MBP |  |  |  | X | **S2**-3d, 4d; **S3**-2b, 2c, 5b |
| ITGA6 |  |  |  | X | **S2**-4a, 5b; **S3**-5c |  | MC1R |  | X |  | X | **S2**-1d, 4b |
| ITGA8 |  |  |  | X | **S2**-4a |  | MCC | X | X | X |  | **S2**-1c, 2a, 2d, 3c, 3d, 4c |
| ITGA2B |  |  |  | X | **S2**-4a; **S3**-2c, 3a |  | MCCC1 |  |  | X |  | **S2**-1c |
| JAK2 |  |  |  | X | **S2**-2a, 5a, 5b; **S3**-1b, 3b, 3c |  | MCFD2 |  |  | X |  | **S2**-3c |
| JARID2 |  |  |  | X | **S2**-4c |  | MCPH1 |  |  |  | X | **S2**-4c |
| KANSL1 |  |  |  | X | **S2**-5b |  | MED13 |  | X | X |  | **S2**-4c |
| KCNA5 |  | X |  |  | **S2**-2a |  | MICAL2 |  | X |  |  | **S2**-3a |
| KDM2B |  |  |  | X | **S2**-4b, 4c; **S3**-3c |  | MITF |  | X | X | X | **S2**-4c; **S3**-3d |
| KIF11 |  |  |  | X | **S2**-5a |  | MMRN1 |  |  |  | X | **S3**-4d |
| KIF14 |  |  | X |  | **S2**-3d |  | MPI |  |  |  | X | **S2**-5b |
| KPNA4 |  | X |  | X | **S2**-3b, 3c, 4b, 5b |  | mPTP (STAR) | X |  |  |  | **S2**-1b, 1c, 2c |
| LAMP1 |  | X |  | X | **S2**-1a, 2a, 3b |  | MREG |  | X |  |  | **S2**-2a |
| LAMP2 |  | X |  |  | **S2**-3b, 4b |  | MRPL18 | X |  |  |  | **S2**-1c |
| LAMP3 |  | X |  | X | **S3**-1d |  | MRPL19 | X |  |  |  | **S2**-1c |
| LDLR |  | X | X |  | **S2**-3a, 3c, 3d |  | MRPL3 | X |  |  |  | **S2**-1c |
| LFA-1 (ITGAL, ITGB2) |  |  |  | X | **S3**-1b, 1d, 3b, 3c, 4c |  | MSX1 |  |  | X | X | **S2**-4c |
| LIPC | X | X |  |  | **S2**-2a |  | MTERFD1 | X |  |  |  | **S2**-1c |
| LMX1A |  |  |  | X | **S2**-5c |  | mTORC1 (AKT1S1, DEPTOR, MLST8, MTOR, RPTOR) | X | X | X |  | **S2**-1b, 2c, 3c, 3d, 4b |
| LMX1B |  |  | X | X | **S2**-4c |  | mTORC2 (DEPTOR, MAPKAP1, MLST8, MTOR, PRR5, RICTOR) | X | X |  |  | **S2**-1d |
| LRP1 |  | X | X |  | **S2**-2a, 2d |  | MYO1E |  | X |  |  | **S2**-2a |
| LRP1B |  |  |  | X | **S3**-4d |  | NAPB |  | X |  |  | NS |
| LRPPRC | X |  |  |  | **S2**-1c, 3d |  | NCAM2 |  |  |  | X | **S2**-5a |
| LRRC16A |  |  | X |  | **S2**-3c |  | NDEL1 |  | X |  |  | **S2**-3a |

| **Supplementary Table 5. (continued)** | | | | | | | | | | | | |
| --- | --- | --- | --- | --- | --- | --- | --- | --- | --- | --- | --- | --- |
| **Protein (csu/iso)** | **A** | **B** | **C** | **D** | **Location coordinates in Figures S2 and S3** |  | **Protein (csu/iso)** | **A** | **B** | **C** | **D** | **Location coordinates in Figures S2 and S3** |
| NDUFAF2 | X |  |  |  | **S2**-1c |  | PRKCE | X | X |  | X | **S2**-1c, 1d, 2c, 3a, 4a, 5a, 5b; **S3**-1c, 2b, 3b |
| NEDD4 |  | X |  |  | **S2**-2a, 2b |  | PRKG1 |  |  | X |  | **S2**-3a, 3d, 4d |
| NEDD8 |  | X | X |  | **S2**-3d, 4c |  | PRKRIR |  | X |  | X | **S2**-4b |
| NEDD9 |  | X |  | X | **S2**-2a, 3b, 5c; **S3**-2b |  | PROK2 |  |  |  | X | **S2**-4b |
| NEGR1 |  |  |  | X | **S2**-5a |  | PSAP |  | X |  |  | **S2**-2a, 3b, 4a, 4b, 4d |
| NEO1 |  |  |  | X | **S3**-2c, 2d |  | PSEN1 |  |  | X |  | **S2**-2c, 2d |
| NEUROG2 |  |  |  | X | NS |  | PSEN2 |  |  | X |  | **S2**-2d |
| NF-KB (NFKB1, NFKB2, REL, RELA, RELB) |  | X |  | X | **S2**-3b, 3c, 4a, 4c, 5a, 5c; **S3**-1c, 2c, 3a, 3b, 4b, 4d, 5d |  | PTEN | X | X | X | X | **S2**-1c, 1d, 2b, 2c, 2d, 4c, 5a, 5c; **S3**-2c, 3c, 4d |
| NLGN1 |  |  |  | X | **S2**-4a, 5a |  | PTPN1 |  |  | X |  | **S2**-2c, 2d, 3c, 4a, 4b, 5a, 5b |
| NMT2 |  |  |  | X | **S2**-5a, 5c |  | PTPRT |  |  |  | X | **S2**-5b |
| NOS1 | X |  |  |  | **S2**-1d |  | RAB7L1 |  | X |  |  | **S2**-3c |
| NOS2 | X |  |  | X | **S2**-1d, 4d; **S3**-3a, 3d, 5d |  | RAC1 |  | X |  | X | **S2**-3b |
| NPTX2 |  |  |  | X | **S2**-4a; **S3**-5c |  | RAI1 |  |  |  | X | **S2**-3c, 4c; **S3**-1b, 2c |
| NR4A2 |  |  |  | X | **S2**-4b, 5c |  | RAP1A |  | X |  | X | **S2**-2c, 3b, 5a, 5c |
| NR5A1 | X |  | X | X | **S2**-3c |  | RAP2A |  | X |  | X | **S2**-2b |
| NSF |  | X |  |  | **S2**-2b, 3a |  | RBFOX1 |  |  | X |  | **S2**-3d |
| NTF3 | X | X |  | X | **S2**-4b; **S3**-4a |  | RCOR1 |  |  |  | X | **S2**-4c; **S3**-3a, 4b |
| OCA2 |  | X |  |  | **S2**-3b |  | RER1 |  | X | X |  | **S2**-2c |
| PALD1 |  | X |  |  | **S3**-5c |  | RFX3 |  | X |  |  | **S2**-4b |
| PARD3 |  | X | X | X | **S2**-3d, 5a; **S3**-2d |  | RFX4 |  | X |  |  | **S2**-4b |
| PARK2 | X | X | X | X | **S2**-1b, 1c, 2a, 2b, 2c, 2d, 3b, 3d, 4a, 4b, 5a |  | RGMA |  |  |  | X | **S3**-2c, 2d |
| PARK7 | X | X | X | X | **S2**-2b, 2c, 3b, 3c, 4c, 5a, 5b; **S3**-3a |  | RHOA |  |  |  | X | NS |
| PCDH8 |  |  |  | X | **S2**-5a |  | RIT2 |  | X |  |  | **S2**-2a, 3a |
| PCM1 |  | X |  |  | **S2**-3a |  | ROBO2 |  |  |  | X | **S2**-4a |
| PEO1 | X |  |  |  | **S2**-1c |  | RPH3AL |  | X |  |  | **S2**-1a |
| PIK3CD | X |  |  | X | **S2**-1d, 2c, 3c; **S3**-1b, 2d |  | RPL38 |  |  | X |  | **S2**-3d |
| PINK1 | X | X | X | X | **S2**-1c, 1d, 2b, 2c, 3d, 4c, 5a, 5b; **S3**-1c, 4b, 5c |  | RPL7A |  |  | X |  | **S2**-3d, 4a, 5b |
| PITX3 | X |  |  | X | **S2**-5c |  | RUFY3 | X |  |  |  | **S2**-1a |
| PLA2G6 | X | X | X |  | **S2**-2b; **S3**-4d |  | RUNX3 |  |  | X | X | **S2**-2d, 4d; **S3**-1b, 3d |
| PLAT |  |  |  | X | **S3**-4c, 4d, 5c |  | RXFP1 |  | X |  |  | **S2**-2a |
| PLAU |  |  |  | X | **S3**-3d, 4c, 4d |  | RXRA |  |  | X |  | NS |
| PLAUR |  |  |  | X | NS |  | SAMD4A |  | X |  |  | **S2**-2c |
| PLCB4 | X |  |  |  | **S2**-1d |  | SCAMP5 |  | X |  |  | **S2**-2a; **S3**-4b |
| PLEKHM1 |  | X |  | X | **S2**-5b |  | SCARB1 |  | X | X |  | **S2**-2a, 3c, 4d |
| PLG |  |  |  | X | **S3**-3c, 4c, 5c |  | SCARB2 |  | X |  |  | **S2**-2b, 3b |
| PLOD1 |  |  | X | X | **S2**-3d, 5c |  | SCIN |  | X |  | X | **S2**-3b |
| PLS1 |  | X |  |  | **S2**-3b |  | SCN2A | X |  |  |  | **S2**-1a |
| POU2F1 |  |  |  | X | **S2**-4c; **S3**-4b |  | SCNN1A | X |  |  |  | **S2**-3c |
| PPM1L |  |  | X |  | **S2**-2d |  | SDC1 |  |  |  | X | **S3**-5c |
| PP1 (PPP1CA, PPP1CB, PPP1CC, PPP1R12A, PPP1R12B, PPP1R14C) |  |  | X |  | **S2**-3a, 3c, 3d, 4c, 4d, 5c |  | SEMA3E |  |  |  | X | NS |
| PRF1 |  |  |  | X | **S3**-3d |  | SEMA6D |  |  |  | X | **S3**-2c |

| **Supplementary Table 5. (continued)** | | | | | | | | | | | | |
| --- | --- | --- | --- | --- | --- | --- | --- | --- | --- | --- | --- | --- |
| **Protein (csu/iso)** | **A** | **B** | **C** | **D** | **Location coordinates in Figures S2 and S3** |  | **Protein (csu/iso)** | **A** | **B** | **C** | **D** | **Location coordinates in Figures S2 and S3** |
| SENP7 |  |  |  | X | **S2**-4a |  | TCF12 | X |  |  | X | **S2**-4b; **S3**-1b, 2d |
| SEP15 |  |  | X |  | **S2**-3c |  | TF | X |  |  |  | **S2**-1a, 1c, 2b |
| SERPINB5 |  |  | X |  | NS |  | TFR2 | X |  |  |  | **S2**-1a |
| SERPINB9 |  |  |  | X | **S2**-5b |  | TGFBR2 |  |  | X | X | **S2**-3d, 5b |
| SERPINE1 |  |  |  | X | **S3**-3d, 4d, 5b, 5c |  | TH | X | X |  |  | **S2**-1b, 1c, 2a, 3d, 5a, 5c |
| SERPING1 |  | X |  | X | **S2**-2a |  | TIAL1 |  |  |  | X | **S3**-3d |
| SIK1 |  | X | X |  | **S2**-1b, 2c, 4c |  | TIMD4 |  |  |  | X | **S3**-2c, 3c |
| SIRT1 | X | X | X | X | **S2**-2b, 2c, 2d, 3c, 4a, 4c, 4d, 5a, 5c; **S3**-3c, 3d, 4d |  | TLE1 |  |  | X | X | **S2**-4c, 4d |
| SIRT7 |  |  | X |  | **S2**-3c, 3d |  | TMCC3 |  |  |  | X | **S2**-5a |
| SLC11A2 | X |  |  |  | **S2**-1a, 1b, 1c |  | TMEM2 |  |  | X |  | NS |
| SLC18A2 | X | X |  |  | **S2**-2b, 5c |  | TMPRSS3 |  | X | X |  | **S2**-3c |
| SLC24A3 | X |  |  |  | **S2**-1a |  | TMPRSS9 |  | X |  | X | **S3**-3d |
| SLC2A13 | X |  |  |  | **S2**-1d |  | TMX1 |  | X | X |  | **S2**-3d |
| SLC40A1 | X |  |  |  | **S2**-1a |  | TNF |  |  |  | X | **S3**-1d, 2c, 3b, 4c, 5c |
| SLC41A1 | X |  |  |  | **S2**-1a |  | TOM1L2 |  | X |  |  | **S2**-2a |
| SLC45A3 |  |  | X |  | **S2**-2d |  | TP53 |  |  |  | X | **S2**-1b, 3a, 3c, 4b, 4c, 5a; **S3**-3a, 3c, 4d, 5b |
| SLC6A3 | X | X |  |  | **S2**-2a, 4a, 5c; **S3**-5c |  | TP53BP1 |  |  |  | X | **S2**-3a, 3c, 4c |
| SLCO3A1 |  |  |  | X | NS |  | TPO | X |  |  |  | NS |
| SLIT2 |  |  |  | X | **S2**-4a, 5c |  | TRAF2 |  |  | X |  | **S2**-3c, 3d |
| SMAD4 |  |  | X |  | **S2**-4d |  | TRAF3 |  |  | X | X | **S2**-3a, 3c, 3d, 5a; **S3**-1b, 1c |
| SMAD5 |  | X | X |  | **S2**-2d, 4d; **S3**-4d |  | TRAPPC9 |  |  |  | X | **S2**-4a |
| SMPD1 |  | X |  |  | **S2**-3b |  | TRPC6 |  | X |  |  | **S2**-1d, 5a |
| SNCA | X | X | X | X | **S2**-1b, 1c, 1d, 2a, 2b, 2c, 2d, 3b, 3c, 3d, 4a, 4c, 4d, 5a; **S3**-1c, 3a, 3c, 4c, 5c |  | TSPO | X |  |  |  | **S2**-1b, 2c |
| SND1 |  | X | X |  | **S2**-2c, 3c, 3d, 4c |  | TYR |  | X |  |  | **S2**-3b |
| SNRK |  | X |  |  | **S2**-2c |  | UCHL1 | X | X | X | X | **S2**-2b, 3b, 3d, 4d, 5b |
| SNRPB2 |  |  | X |  | **S2**-4c |  | ULK2 |  | X | X |  | **S2**-2c, 3c |
| SOD2 | X |  |  |  | **S2**-1c, 4c |  | UNC13B |  | X |  |  | **S2**-1a |
| SOX2 |  |  |  | X | **S2**-4c |  | USP9X |  |  | X |  | **S2**-1a, 2c, 3c, 3d, 4d |
| SP110 |  |  |  | X | **S2**-4b, 4c |  | VDAC (VDAC1, VDAC2, VDAC3) | X |  |  |  | **S2**-1c |
| SPHK1 |  | X |  | X | **S2**-3a; **S3**-5d |  | VDR | X | X |  | X | **S2**-3c, 4c; **S3**-2c, 3c |
| SPNS2 |  | X |  | X | **S2**-3a; **S3**-5d |  | VLDLR |  | X |  | X | **S3**-4c, 4d |
| SPPL2B |  |  |  | X | **S3**-1d |  | VPS35 |  | X | X |  | **S2**-2c, 2d |
| SREBF1 |  | X | X |  | **S2**-2c, 2d, 3c, 4b, 4c; **S3**-4b |  | VPS41 |  | X |  |  | **S2**-2a |
| STAP1 |  |  |  | X | **S3**-1b, 4b |  | WDHD1 |  |  |  | X | **S2**-4b |
| STAT3 |  |  |  | X | **S2**-3a, 4b, 4c, 5a; **S3**-1b, 3b, 3c, 4d |  | WNK1 |  |  | X | X | **S2**-3d; **S3**-3d |
| STK11 |  | X |  |  | **S2**-1d, 2c, 3d, 5a; **S3**-3d |  | WNT3 |  |  | X |  | **S2**-4d |
| STK39 |  |  | X | X | **S2**-3d, 4c, 4d; **S3**-3d |  | WWOX |  |  | X |  | **S2**-2d, 4d |
| SYN3 |  | X |  |  | **S2**-2b |  | ZAK |  | X |  | X | **S2**-4a |
| SYNJ1 |  | X |  |  | **S2**-2a, 2b |  | ZBTB20 |  | X |  |  | **S2**-5c |
| SYT11 |  | X |  |  | **S2**-1b |  | ZNF385B |  |  |  | X | **S3**-1b |
| TACC2 |  |  | X |  | **S2**-4c |  | ZNF423 |  |  | X |  | **S2**-4d |
| TBX3 |  |  |  | X | **S2**-4c |  |  | | | | | |

**DETAILED DESCRIPTION OF THE MOLECULAR LANDSCAPE FOR PARKINSON’S DISEASE**

**TABLE OF CONTENTS**

[INTRODUCTION 46](#_Toc441927003)

[A. OXIDATIVE STRESS RESPONSE 48](#_Toc441927004)

[A1. Dopamine (DA) neuron specificity 48](#_Toc441927005)

[A1.1 DA synthesis, storage and degradation, and neuromelanin (NM) production 48](#_Toc441927006)

[A1.2 Hemoglobin, iron homeostasis and lipoprotein oxidation 50](#_Toc441927007)

[A1.3 Interaction of the DA- and angiotensin-system 52](#_Toc441927008)

[A2. Mitochondrial dysfunction 53](#_Toc441927009)

[A2.1 Regulation of mitochondrial membrane permeability and cytochrome c release 54](#_Toc441927010)

[A2.2 Ca^2+^-induced mitochondrial dysfunction 57](#_Toc441927011)

[A2.2.1 ER stress-induced mitochondrial dysfunction 58](#_Toc441927012)

[A2.2.2 Intracellular Ca^2+^ release and regulation of inositol phosphates 59](#_Toc441927013)

[A2.3 Cholesterol- and oxysterol-induced mitochondrial dysfunction 60](#_Toc441927014)

[A3. FOXO1 and SIRT1: Transcriptional regulators of oxidative stress 62](#_Toc441927015)

[A4. Concluding remarks 63](#_Toc441927016)

[B. ENDOSOMAL-LYSOSOMAL FUNCTIONING 64](#_Toc441927017)

[B1. Sphingolipids 64](#_Toc441927018)

[B1.1 Ceramide and sphingomyelin 65](#_Toc441927019)

[B1.2 Sphingosine 66](#_Toc441927020)

[B1.2.1 Sphingosine-1-phosphate 66](#_Toc441927021)

[B1.3 Cerebrosides and gangliosides 67](#_Toc441927022)

[B2. The endosome-lysosomal system 68](#_Toc441927023)

[B2.1 Clathrin-independent endocytosis 69](#_Toc441927024)

[B2.2 Clathrin-dependent endocytosis 70](#_Toc441927025)

[B2.2.1 Vesicle trafficking and recycling 71](#_Toc441927026)

[B2.2.2 Lipoprotein uptake and processing 72](#_Toc441927027)

[B3. The lysosome-autophagosomal system 73](#_Toc441927028)

[B3.1 AMPK and mTORC1 regulated autophagy 73](#_Toc441927029)

[B3.2 Lysosomal acidification 76](#_Toc441927030)

[B3.3 (Neuro)melanin regulation 77](#_Toc441927031)

[B3.3.1 Vitamin D3 78](#_Toc441927032)

[B3.4 Chaperone-mediated autophagy 79](#_Toc441927033)

[B4. Reverse cholesterol transport 80](#_Toc441927034)

[B4.1 Fat uptake and insulin (INS) 82](#_Toc441927035)

[B5. Concluding remarks 84](#_Toc441927036)

[C. ER STRESS RESPONSE 85](#_Toc441927037)

[C1. Activation of the UPR 85](#_Toc441927038)

[C1.1 UPR feedback inhibition 86](#_Toc441927039)

[C2. Protein aggregation 88](#_Toc441927040)

[C2.1 Protein translation 88](#_Toc441927041)

[C2.2 Ubiquitin 88](#_Toc441927042)

[C2.3 Proteasomal degradation 89](#_Toc441927043)

[C2.4 Presenilins and protein cleavage 90](#_Toc441927044)

[C2.5 Beta-catenin aggregation and -dependent transcription 91](#_Toc441927045)

[C3. ER stress- and cholesterol-regulated gene expression 92](#_Toc441927046)

[C4. Testosterone metabolism 94](#_Toc441927047)

[C4.1 Regulation of testosterone and cholesterol 94](#_Toc441927048)

[C4.2 Androgen receptor (AR) 96](#_Toc441927049)

[C5. Concluding remarks 97](#_Toc441927050)

[D. NEURON DEATH AND IMMUNE RESPONSE 97](#_Toc441927051)

[D1. DA neuron determination, survival and death 97](#_Toc441927052)

[D1.1 Regulation of the “DA signature” 98](#_Toc441927053)

[D1.2 Neurotrophic factors 99](#_Toc441927054)

[D1.3 Pro-apoptotic proteins 100](#_Toc441927055)

[D1.3.1 GSK3B 100](#_Toc441927056)

[D1.3.2 PTEN 101](#_Toc441927057)

[D1.3.3 TP53 102](#_Toc441927058)

[D1.3.4 CASP3 103](#_Toc441927059)

[D2. Immune regulation 103](#_Toc441927060)

[D2.1 Immune-related pathways 104](#_Toc441927061)

[D2.1.1 JAK2/STAT3 104](#_Toc441927062)

[D2.1.2 NF-KB 105](#_Toc441927063)

[D2.1.3 Cell adhesion, cell-cell interaction, axon guidance and immune regulation 107](#_Toc441927064)

[D2.2 Immune cell activation 109](#_Toc441927065)

[D2.2.1 Activation of the innate immune response 111](#_Toc441927066)

[D2.2.2 Activation of the adaptive immune response 113](#_Toc441927067)

[D2.2.3 Coagulation factors, lipoproteins and immune cell activation 118](#_Toc441927068)

[D3. Concluding remarks 120](#_Toc441927069)

[REFERENCES 122](#_Toc441927070)

## INTRODUCTION

In **Supplementary Figures** **2** and **3** all relevant protein interactions that constitute the molecular landscape of Parkinson’s disease (PD) are shown. In this description, the various interactions operational within the molecular landscape for PD are grouped into four main processes, (A) oxidative stress response, (B) endosomal-lysosomal functioning, (C) endoplasmic reticulum (ER) stress response, and (D) neuron death and immune response. Obviously, these processes overlap, and some of the proteins and protein signaling cascades are involved in multiple processes. Nevertheless, recurrent description of processes and interactions is avoided as much as possible. In **Supplementary Figures 2** and **3**, these processes are designated with the letters A-D. **Supplementary Table 5** gives an overview of all the proteins in the PD landscape, where they are located in **Supplementary Figures S2** and **S3** and in which process(es) they exert their main effect.

In all descriptions of the PD landscape, names of proteins derived from the genome-wide association studies (GWASs) are in **bold**, single-underlined proteins are genetically associated with PD, dotted underlined genes encode proteins that are differentially expressed in PD and double underlined genes are genetically associated with PD *and* encode a protein that is differentially expressed in PD. **Supplementary Table 2** gives an overview of all GWAS gene-encoded proteins per GWAS and the corroborating evidence for their associations with PD. **Supplementary Table 4** shows all other landscape proteins and the corroborating evidence for their associations with PD.

Not all GWAS gene-encoded proteins were placed in the landscape, either because there were no connections with other landscape proteins or due to lack of annotation, or both. However, this does not necessarily mean that they are not involved in PD pathophysiology. For example, SPPL2C is one of the genes that is not included in the landscape, but is still on the shortlist of highly interesting genes in relation to PD. Namely, SPPL2C is one of the most significantly PD-associated genes in four different PD GWASs^6, 10, 12, 262^, which includes association with two non-synonymous coding SNPs in SPPL2C. However, due to lack of annotation and knowledge about its protein function^263^ it could not be placed in the landscape. Future research should clarify if this highly associated gene indeed encodes a functional active protein^263, 264^ that is of importance in PD pathophysiology.

Overall, two generalization have been made. First, when a knock-out of protein A in a cell or animal model *increases* the expression of protein B, we assume that endogenous expression of protein A leads to the opposite effect and *decreases* the expression of protein B. Second, we assumed that all identified protein interactions (in any organism and/or cell type) can be extrapolated to the interactions in human DA neurons and immune cells.

In the descriptions below, the gene name abbreviations refer to both the gene and the protein. Furthermore, the terms ‘activates’ and ‘inhibits’ are used to indicate the activation of a protein (e.g. by (de)phosphorylation) by another protein. To describe a difference in abundance of a protein (directly or indirectly) induced by another protein, the terms ‘increases the expression of’ or ‘decreases the expression of’ are used. And lastly, interactions in the PD landscape that are not shown in the figures are indicated with ‘(not shown)’ in the text.

## A. OXIDATIVE STRESS RESPONSE

Oxidative stress in DA neurons is increased by cell-specific processes (A1), dysregulation of mitochondria and oxidative phosphorylation (A2) and by dysregulation of regulators of oxidative stress (A3). The functional interactions between the proteins within process A, ‘oxidative stress response’, are shown in **Supplementary Figure 2**.

### A1. Dopamine (DA) neuron specificity

The processes that have been associated with PD, as discussed in this and the next sections – such as autophagy, lysosomal degradation, ER stress, mitochondrial destabilization and apoptotic cascades – are often generic processes that can occur in virtually every cell in the body. However, midbrain dopamine (DA) neuron death is a hallmark of PD, implying a cell-specific vulnerability for dysregulation of these processes. Therefore, sections A1.1 - A1.3 describe processes that are (highly) specific to DA neurons and may play a crucial role in their vulnerability. Subsequently, a genetic predisposition for defects in more generic processes such as autophagy, lysosomal degradation, ER stress, mitochondrial destabilization or proteasomal degradation (as discussed in the next chapters) would increase the vulnerability of these neurons beyond that of neurons that do not synthesize DA or other monoaminergic neurotransmitters. In this way, a relatively small increase in protein aggregation, mitochondrial destabilization and/or lysosomal dysfunctioning may lead to specific DA neuron death.

#### A1.1 DA synthesis, storage and degradation, and neuromelanin (NM) production

In PD patients, monoaminergic neurotransmitter-synthesizing neurons are vulnerable and degenerate^265^. In DA neurons, DA can be spontaneously oxidized to the NM precursor aminochrome, an oxidation process that produces oxygen radicals and increases oxidative stress^266^. DA oxidation results in increased reactive oxygen species (ROS), that are involved in the formation of oxysterols and in lipid peroxidation. Indeed, PD patients show increased lipid peroxidase activity, indicated by the elevated presence of the lipid peroxidation end products malondialdehyde^179, 267-269^ and 4-hydroxynonenal^270, 271^.

Monoamine oxidase A (MAOA) increases the expression of SLC18A2^272^, that sequesters DA into secretory vesicles. SLC18A2 (that binds to PARK7^273^) thereby prevents DA oxidation^274^, as is suggested from the inverse association between SLC18A2 expression and both NM production in the SN and vulnerability to neuronal degradation in the human postmortem brain^274^, reduced vesicular storage of DA by SLC18A2 causing nigrostriatal neurodegeneration in mice^275^ and association studies showing that gain of function polymorphisms in the SLC18A2 gene promotor are protective against PD in females^229, 230^. Taken together increased sequestration of DA in secretory vesicles by SLC18A2 appears to have neuroprotective properties against PD.

Apart from DA loading into secretory vesicles, DA oxidation can also be prevented by monoamine oxidase (MAO)-dependent (occurring in two forms, MAOA and MAOB) or catechol ortho-methyl transferase (COMT)-dependent degradation of DA^266^ (all three genetically associated with an increased PD risk^145, 194, 196, 197^). More specifically, MAOA variations are associated with PD in males^194-196^, MAOB variations are associated with PD in females^198^ and a COMT polymorphism with lower age of onset in males^146^, showing that altered DA degradation affects the risk for PD. **PARK2** decreases the expression and inhibits the activity of both MAOA and MAOB^276^, whereas AR^277^ and SIRT1^278^ increase MAOA expression, calcium (Ca^2+^) increases MAOA activity^279^ and vitamin D3 decreases MAOB expression and activation^280^. Further, COMT expression is increased by **ANGPT2**^281^ (not shown) and TP53^282^. Thus, the expression of the DA degradating enzymes MAOA, MAOB and COMT is regulated by proteins in the PD landscape.

Taken together, decreased transport into secretory vesicles or decreased degradation of DA increases DA auto-oxidation and thus cellular aminochrome levels, which can have major effects on cellular processes by inhibiting the proteasome^283^, increasing cellular NM levels^266^ and binding to the familial PD proteins **SNCA**^284^, UCHL1^285^, **PARK2** (and aminochrome also inhibits **PARK2**^286^) and PARK7^287^. Aminochrome is speculated to affect chaperone mediated autophagy (CMA) function (for more on CMA see section B3.4) by binding to **SNCA** and UCHL1^266^, by inducing formation of **SNCA** protofibrils^284^ that inhibit CMA just like mutated **SNCA**^266^ and UCHL1^288^.

The mRNA and protein levels of tyrosine hydroxylase (TH), the rate-limiting enzyme in DA synthesis in the cytoplasm of DA neurons^289^, are decreased in PD SN neurons^246^. TH expression is normally increased by PARK7^290^, **CRHR1**^291^, **NTF3**^292^ (not shown), the DA transporter SLC6A3^293^ and the transcription factor PITX3^294^, and decreased by **SNCA**^295, 296^ (accumulates in lewy bodies^40, 41^), PTEN^297^ and **CREM**^298^, and inhibited by the DA receptor DRD2^299^. Cytoplasmic TH binds to **SNCA**^300^ and SLC18A2^301^ (not shown), suggesting a direct regulation of TH by **SNCA**. Further, SLC18A2 is found in a complex together with the DA transporter SLC6A3^302^ (not shown), i.e. in close contact with the DA transporter responsible for DA influx in the cell. SLC6A3, in turn, binds to the familial PD proteins **SNCA**^303^ and **PARK2**^304^.

In summary, monoaminergic neurotransmitter-synthesizing neurons are more vulnerable due to aminochrome production that increases the cellular burden by inhibiting the proteasome, increasing cellular NM levels and affecting familial PD proteins that regulate autophagy, CMA, mitochondrial and lysosomal stabilization and proteasomal degradation.

#### A1.2 Hemoglobin, iron homeostasis and lipoprotein oxidation

Hemoglobin (Hb) is an iron-containing metalloprotein that in the blood transports oxygen from the lungs to the rest of the body. Hb is a tetramer of globin subunits (HBA1, HBA2, HBB, HBD, HBE1, HBG1, HBG2, HBM, HBQ1 and HBZ) that changes its composition during embryonic and fetal development. During adult life the most common Hb is a tetramer of two alpha (HBA1 or HBA2) and two beta (HBB) subunits^305^. Higher levels of late-life Hb^161^ and genotypes of the Hb-binding protein haptoglobin (HP) are associated with increased PD risk^169^. PD patients show an increased number of HBA1 containing mitochondria, reduced mitochondrial/cytoplasmic HBB ratios in SN neurons^162^. HP, which also binds HDL^306, 307^, prevents oxidation of Hb and thereby ensures its transport into the cell by the scavenger receptor CD163^308^. SN DA neurons are (next to red blood cells) one of the few cell types in the body that express Hb^309^, where Hb may be involved in iron metabolism, oxygen supply and mitochondrial function^309^. Oxidation of hemeproteins (e.g. Hb) can result in the release of their heme group^310^, which can become cytotoxic in the presence of multiple inflammatory factors^310^. Heme is also a cofactor for the thyroid peroxidase **TPO** (not shown; necessary for thyroid hormone synthesis and reducing hydrogen peroxide to water)^311, 312^ and essential for NOS1 activation^313^ and therefore affects (anti)oxidative reations. The lipoproteins HDL and LDL are the initial heme scavengers in the circulation and get oxidized (to oxHDL and oxLDL, respectively) by binding to heme^310^, which also renders them toxic. Of note, oxLDL is increased in the plasma of (L-DOPA treated) PD patients^177^. Clearance of heme by its conversion into biliverdin by the heme oxygenases HMOX1 and HMOX2^312^ prevents the oxidation of HDL and LDL by oxidized Hb. The inducibe heme oxygenase HMOX1 is increased in PD serum^167^, the number of HMOX1-positive astroglia (see main process D) is increased in the PD SN^166^ and lewy bodies in the SN neurons show intense HMOX1 expression^166^. Also, the constitutively active heme oxygenase HMOX2 is genetically associated with PD^168^. EIF4E increases HMOX1 translation^314^ and HMOX1 expression is increased by oxLDL^315^, **ANGPT2**^316^, STAT3^317^ (not shown), AKT1^318, 319^ (not shown), FOXO1^320^, RAC1^321^ (not shown) and **SREBF1**^322^ and decreased by ferrous iron (Fe(II))^323^ and NF-KB^324^ (not shown). Further, HMOX1 activation is increased by TP53^325^, ER stress^326^ (not shown), hypoxia^327^ (not shown), superoxide^328^ (not shown) and DA^329^ and inhibited by SOD2^330^ (not shown). Downregulation of HMOX1 results in increased **SNCA** aggregation^323^. Moreover, HMOX1 increases the expression of BDNF^331^, GDNF^331^, TH^332^, ABCA1^333^, **ABCG1**^333^, PITX3^332^ (not shown), **ANGPT2**^334^ and DA^332^, decreases the expression of CXCL12^335^ and ICAM1^336, 337^ (both part of main process D) and inhibits the accumulation of cholesterol^333^.

Conversion of heme into biliverdin by HMOX1 results in the production of carbon monoxide and the highly oxidative ferrous iron (Fe(II))^312^. NM binds Fe(II) and is in DA neurons the main Fe(II) store^338, 339^. Fe(II) is also a cofactor for TH in the DA synthesis pathway^340^, but highly cytotoxic and its cytoplasmic levels should therefore be tightly controlled. In the SN of PD patients the total iron content is higher than in controls^228^, whereas increased *serum* iron levels are associated with a *decreased* risk of developing PD^341^. SLC40A1 transports Fe(II) out of the cell and is upregulated in the PD SN^231^. SLC11A2, another iron transporter, is genetically linked to PD risk^227^ and its different isoforms are differentially expressed in PD^228^. SLC11A2 isoforms transports Fe(II) from endosomes into the cytoplasm and also into the mitochondria^342^. **PARK2** increases proteasomal degradation of SLC11A2^343^, putatively decreasing cytoplasmic and mitochondrial Fe(II). Ferric iron (Fe(III)) can be transported into the cell by binding to transferrin (TF^344^; part of the HDL-complex^345^). TF binds to the transferrin receptor 2 (TFR2, the number of TF binding places, as a measure for transferring receptor expression, is lower on PD DA SN neurons^245^ and TFR2 expression is highly specific for SN DA neurons^244^), and is transported into an endosome where the low pH releases Fe(III) from TF^346^. The PD SN shows a dramatic increase of oxidized TF^244^. This oxidation of TF results in the reductive release of Fe(II) from TF^244^ and increases the oxidative cross-linking of TF to other proteins by formation of a disulfide bond, which likely impairs normal trafficking of TF^244^ and thus affects the localization of (cytotoxic) iron ions.

In summary, Hb and iron homeostasis increase the ‘oxidative burden’ of the DA neuron, and may also result in an increased oxidation of lipoproteins. Of note, low cholesterol intake, especially in combination with high dietary iron intake, increases PD risk^347^, perhaps due to insufficient (unoxidized) LDL (the major blood cholesterol carrier) in the PD brain, affecting the downstream targets and regulation of lipoproteins.

#### A1.3 Interaction of the DA- and angiotensin-system

The angiotensin II receptor **AGTR1** is involved in the renin-angiotensin system (RAS), that regulates water homeostasis and hypertension, of which the latter is associated with PD^348-350^. Moreover, overactivation of the RAS results in hypertension, increases superoxide formation by activating NADPH-dependent oxidases, increases microglial activation and increases oxidative stress as shown in PD animal models^351^. The DA and angiotensin II systems directly counterregulate each other in the ‘classical’ RAS in the renal cells^352^, but also in local RAS in the brain. Namely, in the striatum and SN angiotensin II exacerbates toxin-induced DA neuron death via **AGTR1** activation, whereas DA depletion increases the expression of the angiotensin receptors^353^. DA depletion may therefore result in increased RAS activation and exacerbate the angiotensin II-regulated oxidative stress and microglial inflammatory responses^354, 355^ and thereby contribute to the degeneration of DA neurons^351^.

Aging in general is associated with an increase in RAS activation, which can be reduced by angiotensin II antagonists and subsequently prevent SN DA neuron degeneration in PD models^351^. **AGTR1** regulates the JAK/STAT pathway^356-358^, caspase^359^ and NF-KB signaling^360, 361^, E3 ubiquitin-protein ligase complexes^362^, coagulation factors (for more on coagulation factors see section D2.2.3)^363, 364^, increases cholesterol esterification^365^, mobilization of intracellular Ca^2+^ and the production of inositol phosphates^366^, ER stress factors^367, 368^, autophagy^369, 370^ and endocytosis^371^. Thus, dysregulation of the RAS-dopamine system interaction can affect many cellular processes in the PD landscape. The angiotensin 1-7 receptor **MAS1** upregulates^372^, binds and is a functional antagonist of **AGTR1**^366^. Further, **AGTR1** expression is decreased by the secreted vascular remodeling protein angtiopoietin-2 (**ANGPT2**)^373^ and HDL-cholesterol^374^ (which levels are positively correlated with the duration of PD^375^) and increased by the oxysterols 24-hydroxycholesterol (24-OHC, which CSF-levels correlated with the duration of PD^376^) and 27-hydroxycholesterol (27-OHC)^377^ (for more on 24-OHC and 27-OHC see section B4).

In summary, the RAS and DA system counterregulate each other, indicating that the regulation of local brain RAS is important for a normal DA system. DA depletion, aging and oxysterols increase RAS activation, that in turn regulates multiple pathways in the PD landscape leading to increased oxidative stress, microglial activation and DA neuron death.

### A2. Mitochondrial dysfunction

Mitochondrial dysfunction, increased oxidative stress and release of the apoptosis-inducing cytochrome c may be caused by imperfect functioning mitochondrial proteins e.g., dysregulation of the mitochondrial transcription factor **MTERFD1**^378^ (its inactivation leads to respiratory complex deficiency^379^), the mitochondrial DNA regulator **PEO1**^312^, the mitochondrial complex I assembly chaperone **NDUFAF2**^380^ or the mitochondrial 39S ribosome complex proteins **MRPL3**, **MRPL18** and **MRPL19**^312^, which may impair mitochondrial protein translation.

The superoxide dismutase SOD2 (differentially expressed in the PD brain^237, 238, 381, 382^) neutralizes superoxide, the toxic byproduct of the respiratory chain^239^ and decreases release of cytochrome c from the mitochondria^383^. Further, SOD2 inhibits **ATF6**^384^ (not shown), regulates expression of **LIPC**^385^ (not shown), increases expression of the GTP cyclohydrolase **GCH1**^386^ (not shown) and binds **MCC**^387^. And, SOD2 expression is decreased by PARK7^388^, increased by FOXO1^389^ and **ANGPT2**^281^ (not shown) and enzymatic activity of SOD2 is attenuated (up to 50%) by DA neuron specific reactive dopamine-quinones^239^. Further, LRPPRC is an essential posttranscriptional regulator of (mitochondrial) mRNA^390^, localizes predominantly to mitochondria but also to the nuclear membrane^312^. LRPPRC interacts with and its expression is increased by PINK1^391, 392^, interacts with **PARK2**^393^ (not shown) and binds to **MCC**^387^, SIRT7^394^ (not shown), **FBXO25**^395^ and **CECR2**^396^. Dysregulation of LRPPRC results in defective regulation of cytochrome c oxidase subunits^392^. Thus, both SOD2 and LRPPRC may affect cytochrome c production in the mitochondria.

In addition to self-regulation, the mitochondria are also highly regulated by external factors from the cytoplasm. The next sections will discuss the regulation of mitochondrial (dys)function by familial PD proteins and the mitochondrial permeability transition pore (A2.1), by Ca^2+^ (A2.2) and by cholesterol and oxysterols (A2.3).

#### A2.1 Regulation of mitochondrial membrane permeability and cytochrome c release

Mitochondrion integrity and degradation is highly regulated by familial PD proteins, suggesting a key role in (familial) PD pathogenesis. Loss of the mitochondrial transmembrane potential (ΔΨm) results in activation of caspase-independent cell death pathway that is controlled by the serine protease HTRA2^397^ and mitochondrial accumulation of PINK1^398^. HTRA2 degradates denatured mitochondrial proteins and promotes apoptosis when released into the cytoplasm^399^ and accumulated mitochondrial PINK1 increases mitochondrial Ca^2+^ levels and opening of the mitochondrial permeability transition pore (mPTP)^400^ and subsequent cytochrome c release to the cytoplasm^401^. Further, accumulated PINK1 recruits **PARK2** to mitochondria to initiate mitophagy^398, 402, 403^. This recruition of **PARK2** to mitochondria is disturbed in FBXO7 deficient cells^404^. Further, HTRA2 binds and cleaves **PARK2** and thereby inhibits its E3 ubiquitin ligase activity^405^ and **PARK2** is bound and inhibited (via ubiquitination) by **LRRK2**^406^ (binds **SNCA**^407^). Thus, PINK1, FBXO7, HTRA2 and **LRRK2** regulate **PARK2** that, by ubiquinating BAX, functions as a ‘gateway’ for BAX transport to the mitochondrial membrane and subsequent cytochrome c release^408^.

Cytochrome c (endoded by CYCS) is an essential component in the mitochondrial electron transport chain, but is also involved in the initiation of apoptosis when release from the mitochondria into the cytoplasm. Cytochrome c release is increased by **ATP2B2**^409^ (not shown), PTEN^410^, **AMPK**^411^, RAC1^412^ (not shown), TP53^413^ (not shown), CASP3^414^, CASP9^415^ (not shown; activated in TH+ SN neurons of late-onset PD patients^138^), cholesterol^416^, ceramide^417^, sphingosine^418^, NM^419^ and L-DOPA^420^ and decreased by EIF4E421, SOD2^383^, **PRKCE**^422^ (not shown), **MAPT**^423^, ATP13A2^424^ (not shown), PINK1^425^, PARK7^426^ (inhibits **SNCA** aggregation^427^) and **PARK2**^428^. Cytoplasmic cytochrome c increases the aggregation of **SNCA**^429^ and activates the apoptotic caspase pathway by activating CASP9 and subsequently CASP3^430-433^, which are also regulated by the familial PD proteins, i.e. CASP3 is activated by UCHL1^434^ and HTRA2^435^ and inhibited by PARK7 and PINK1^436, 437^, whereas CASP9 is activated by HTRA2^438^ and inhibited by PARK7^436^.

The proapoptotic proteins BAX and BAK1 bind to the voltage-dependent anion channel (VDAC), which results in loss of ΔΨm, mPTP opening, release of cytochrome c in the cytoplasm and activation of the caspase pathway by activation of CASP9 and CASP3^439-444^. The VDAC (there are three VDAC isoforms present in humans; VDAC1-3) is the major mitochondrial outer membrane channel, that allows ATP/ADP exchange between cytoplasm and mitochondria and allows the transport of ions (e.g. Ca^2+^), lipids (e.g. cholesterol) and metabolites over the mitochondrial outer membrane^312, 445, 446^. The VDAC opens at low membrane potentials and closes at potentials above 30mV^447-449^ and regulates, but is not necessary for mPTP formation^450-453^. Of note, in yeast cells **SNCA**-toxicity is dependent on VDAC. More precisely, the VDAC is able to transport monomeric **SNCA** into mitochondria and is blocked by **SNCA**. In this way, **SNCA** disrupts the VDAC-mediated ATP/ADP exchange, decreases ΔΨm and impairs oxidative phosphorylation^454^ (not shown). In addition to BAX and BAK1, VDAC also binds to CAV1^455^ (not shown), cholesterol (may also affect VDAC functioning)^456^, **HLA-B**^387^ (not shown), **LRRK2**^457^, **MCC**^387^, **PARK2**^458, 459^, PLAT (on the plasma membrane)^460^ (not shown), **PRKCE**^422^ (not shown), SIRT7^394^ (not shown), **SNCA**^461^ and the mPTP components STAR^462^ and TSPO^463^. Further, VDAC accumulation^440^ and oligomerization induces apoptosis^464, 465^ and its expression is increased by **COL18A1**^440^ (not shown), IL2^466^ (not shown) and vitamin D^467^ (not shown) and decreased by TSPO^468^ (not shown). VDAC recruits **PARK2** to dysfunctioning mitochondria to induce mitophagy^458^. Ubiquitinating of VDAC by **PARK2** is PINK1-dependent^469^, and necessary for **PARK2**-mediated mitophagy^470^. Apoptotic pathways are activated via regulation of VDAC by BAX^471^, **COL18A1**^440^ and GSK3B^472^ and inhibited by **PRKCE**^422^. Of note, DA decreases the ΔΨm and *reduces* VDAC levels on the mitochondrial outer membrane^473, 474^, but this DA toxicity is not counteracted by (VDAC-dependent^458, 470^) mitophagy and thereby enhances oxidative stress, which may possibly explain DA neuron death in PD^474^.

Binding and stabilization of hexokinase 2 (HK2) to VDAC and the mitochondrial outer membrane suppresses (BAX-induced) cytochrome c release, CASP3 activation and apoptosis^475-477^, by preventing mitochondrial VDAC accumulation^440^. HK2 locates to the outer membrane of mitochondria and is involved in glucose metabolism by phosphorylating glucose to glucose-6-phosphate^312^. HK2 also protects against neurodegeneration in rotenone and MPTP mouse models of PD^478^ and it is proposed that the VDAC-HK2 complex is necessary to generate the ΔΨm^479^. **COL18A1** and GSK3B both increase phosphorylation of VDAC, which disrupts binding of the hexokinase HK2 to VDAC and decreases ΔΨm^440, 472^, whereas GSK3B inhibition increases the accumulation of HK2 in mitochondria, enhances glycolysis in the cell and increases neuronal survival^480^.

HK2 expression is regulated by binding of STAT3 and **SREBF1** to the HK2 gene promotor^481, 482^ and is increased by AKT1^483^ (not shown), mTORC1^484, 485^ (not shown), CAV1^486^ (not shown), STAT3^487^, IFNG^488^ (not shown), IL1B^489^ (not shown), insulin (INS)^490^ (not shown) and mutant TP53^491^ (not shown) and decreased by **COL18A1**^440^ (not shown). HK2 binds to **PRKCE**^422^ and **PARK2**^459^ and INSR activation results in HK2 translocation to mitochondria (via the AKT1 pathway)^492^ (not shown).

Thus, by regulating VDAC and HK2, cytochrome c release by the mPTP and subsequent activation of the caspase pathway can be suppressed. Of note, prevention of apoptosis by HK2 stabilization to VDAC and the mitochondrial outer membrane also favours the glycolysis pathway followed by lactic acid fermentation and reduces the oxidation of pyruvate in mitochondria^475, 493^. This change in energy production, from aerobic to anaerobic, is called the Warburg effect and reduces the production of ROS and is often seen in cancer cells^494^. Further, **PARK2** deficiency activates glycolysis and reduces aerobic respiration by the mitochondria^495^.

#### A2.2 Ca^2+^-induced mitochondrial dysfunction

The VDAC (as seen in A2.1) regulates transport of **SNCA**, metabolites and ions, such as Ca^2+^, over the mitochondrial membrane. However, the rate of their transport into the mitochondria also depends on their cytoplasmic levels. High cytoplasmic Ca^2+^ levels increase the transport of Ca^2+^ into the mitochondria. Therefore, regulation of cytoplasmic (**SNCA**) and Ca^2+^ levels is important to maintain homeostasis.

Of note, SN DA neurons are able to generate action potentials in the absence of synaptic input. They are autonomously active, which is regulated via Ca^2+^ entry through L-type calcium channels (composed of four subunits; CACNA1C, CACNA1D, CACNA1S and CACNA1F^312^)^496^. Ca^2+^ influx is therefore an important factor in regulating the basal activity of DA neurons. Further, L-type calcium channel-mediated Ca^2+^ influx enhances the production of DA from L-DOPA^497^, the use of L-type calcium channel blockers has been shown to reduce the risk of developing PD in a Danish population^498^. Furthermore, in the PD SN the number of cells that express CACNA1C and CACNA1D is lower^134^, but in these cells CACNA1D is higher expressed than in controls^134^. And, the CACNA1D subunit is also shown to be higher expressed in the PD SN^135^. Lastly, also the CACNA1D to CACNA1C ratio is increased in PD brains^134^. Thus, by regulating the intrinsic tonic firing typical for DA neurons, Ca^2+^ ensures that there is a continuous DA supply to target areas such as the striatum. This however exposes SN neurons to a higher Ca2+ influx than other neurons and it is hypothesized that together with the low Ca^2+^ buffering capacity of SN neurons, this influx is directly responsible for mitochondrial stress and increased ROS production, which makes them more vulnerable^499, 500^. Increased intracellular Ca^2+^ levels disrupt mitochondrial membrane integrity, which results in cytochrome c release and apoptosis^501^.

Secreted **SNCA**-toxicity is mediated by an increased Ca^2+^ influx and deregulation of the cellular Ca^2+^ homeostasis^503^. And, intracellular Ca^2+^ levels can also be increased by **CXCR4**, by **AGTR1** via activation of G proteins (e.g. **GNA12**)^312^, by influx through or mediated by the **Na^+^/K^+^/**Ca^2+^**-exchanger SLC24A3**^312^ **(**highly expressed in nigral DA neurons, and a potential role in DA neuron survival^504^**), by** the Ca2+-binding protein **EFCAB4B** (plays a key role in store-operated Ca2+ entry in T-cells^312^) **and the** voltage-dependent calcium channels (such as the L-type calcium channel subunits mentioned above, but also by other types of channels consisting of alpha-1 (**CACNA1A**), alpha-2 (**CACNA2D3**), beta and delta subunits^312^). These calcium channels are mediated by multiple proteins in the landscape i.e., **CACNA1A** binds to **USP9X**^505^, **MAP1B**^505^, **EHMT2**^506^ (not shown), LRP1^506^, SYNJ1^505^, CTNNB1^505^ and **AMIGO2**^506^ (activates the NF-KB complex^507^ (not shown)) and is inhibited by the dopamine receptor DRD3^508^ (not shown). Further, DA decreases the expression of **CACNA2D3**^509^ (not shown), which is high and highly specific expressed in the rat SN and is decreased after 6-OHDA treatment ^504^.

Other alpha-1 calcium channel subunits are also regulated by proteins in the landscape. CACNA1B (increases blood INS level^510^) binds **SCN2A**^505^ (**SCN2A** also binds to **FGF12**^511^), **CNNM2**^505^, **MAP1B**^505^ and the regulator of axonogenesis **RUFY3**^312, 505^ and is regulated by the dopamine receptor DRD2 (not shown)^512^. Further, CACNA1E also binds to **RUFY3**^505^ (not shown), CACNA1C binds SIRT1^513^ and alternative splicing of CACNA1D and CACNA1S (both L-type calcium channel subunits) is regulated by **RFBOX1**^514^ (not shown).

Administration of Mg^2+^ reduces Ca^2+^ mediated microglial DA neurotoxicity in PD^515^ and increases the kinase activity of **LRRK2**^516^. Mg^2+^ is transported out of the cell by the transporters **CNNM2**^517^ and **SLC41A1**^518^. Moreover, the magnesium dependent ATPase **ATP2B2** couples ATPase activity to Ca^2+^ efflux^312^ and its overexpression depletes intracellular Ca^2+^ stores and triggers apoptosis^409^.

Thus, voltage-gated calcium channels regulate Ca^2+^ levels in the neuron, which is important for the intrinsic tonic firing of DA neurons and DA release, and are themselves regulated by multiple landscape proteins (e.g. the DA receptors DRD2 and DRD3). Increased Ca^2+^ influx increases mitochondrial dysfunction and increases **SNCA**-toxicity.

##### A2.2.1 ER stress-induced mitochondrial dysfunction

Oxidative stress increases the influx of Ca^2+^ into the cytoplasm from the ECM, but also increases release of Ca^2+^ from the ER Ca^2+^-store^519^. Acute release of Ca^2+^ from the ER triggers Ca^2+^-mediated mitochondrial cell death^520^. Prolonged ER stress (disruption of normal ER function, resulting in activation of the unfolded protein response (UPR; see also section C1)) results in a perturbed mitochondrial function through a disturbed ER-mitochondrial Ca^2+^ homeostasis^521-523^, i.e. BAX and BAK1 oligomerize in the ER membrane and allow Ca^2+^ release to the cytoplasm^524^, which is taken up by the mitochondria resulting in loss of the ΔΨm^522, 524^. Further, during ER stress BAX translocates to the mitochondrial membrane (increased by mitochondrial lipid rafts^525^) where it interacts with the mPTP leading, together with the loss of ΔΨm, to cytochrome c release in the cytoplasm^439, 521, 524, 526^, which results in caspase activation and apoptosis^397^.

The relevance of these processes in PD is illustrated by the increased BAX immunoreactivity in NM-containing neurons^125^ and differences in aggregation of BAX-rich inclusions in PD^126^. Moreover, BAK1-deficient mice were resistant to paraquat neurotoxicity, a model for PD^527^ and the receptor for humanin, **FPR3** (not shown), that mediates the anti-apoptotic activity of humanin and suppresses BAX-dependent apoptosis^312^, was found in the GWASs^6^.

Hence, ER stress results in BAX-dependent apoptosis, through Ca^2+^ release from the ER that is taken up by mitochondria, leading to a loss of ΔΨm, mitochondrial dysfunction, cytochrome c release and activation of apoptotic pathways.

##### A2.2.2 Intracellular Ca^2+^ release and regulation of inositol phosphates

Ca^2+^ release from intracellular stores (including the ER) is also increased by the secondary messenger inositol triphosphate (IP3)^528^, which is produced together with diacylglycerol (DAG) by cleaving of phosphatidylinositol 4,5-bisphosphate (PIP2) by **PLCB4**^312^. Myo-inositol serves as an important component of inositol phosphates (e.g. PI, PIP2, PIP3 and IP3) and is transported into the cell by the proton myo-inositol cotransporter **SLC2A13**^529^ (genetically associated with PD^53^). **PIK3CD** generates the AKT1 activating PIP3 (phosphatidylinositol (3,4,5)-trisphosphate) by phosphorylating PIP2^312^ and increases intracellular Ca^2+530^. Further, **PIK3CD** also inhibits PTEN^531^, that generates PIP2 by dephosphorylating PIP3^312^ and thereby functions as an antagonist for the AKT1 signaling pathway^312^. The **SNCA-**binding^532^ serine/threonine-protein kinase **PRKCE** binds to diacylglycerol kinase theta (**DGKQ**, associated to PD^27-29^) and increases its translocation to the plasma membrane^533^. **DGKQ** phosphorylates DAG and thereby produces phosphatidic acid (PA)^312^. PA quantity is also increased by S1P^534^ (not shown). PA and DAG are both essential for **PRKCE** activation and translocation to the plasma membrane^535^. Further, PA also activates, and is necessary for mTORC1 signaling^536-538^, binds NR5A1^539^ (not shown) and increases NR5A1-dependent expression^539^, inhibits PPP1CA^540^, activates SPHK1^541^ (and increases its translocation to the plasma membrane^542^), activates **AGAP1**^543^ (GTPase activity stimulated by PIP3 and PIP2, whereas PA potentiates PIP2 activation^543^), activates the reverse activity of **ASAH1** (resulting in ceramide production)^544^ (part of main process B), increases **MAPT** phosphorylation (via MTOR activation)^545^ (not shown), binds HIP1R^546^ (not shown) and **MBP**^547^ (not shown) and induces fibrillization of **SNCA**^548^. Thus, inositol phosphate regulation affects Ca^2+^ release and production of PA affects among others, energy and redox sensing (mTORC1), sphingosine regulation (**ASAH1**, SPHK1) and **SNCA** aggregation as seen in PD.

#### A2.3 Cholesterol- and oxysterol-induced mitochondrial dysfunction

Several findings suggest that lipid metabolism is involved in PD pathogenesis. For example, high dietary intake of (poly)unsaturated fatty acids and plasma hypercholesterolemia are associated with lowering the risk of PD^350, 549, 550^, low plasma levels of LDL-cholesterol and total cholesterol are associated with an increased PD risk^551-554^ and PD disease duration is positively correlated with plasma HDL-cholesterol^375^. Dietary cholesterol increases nicotinamide adenine dinucleotide (NAD) synthesis from tryptophan by inhibiting the decarboxylase **ACMSD**^555^ (not shown), that is also downregulated by long chain fatty acids^556^. NAD+ is reduced to NADH in the citric acid cycle or during β-oxidation or glycolysis, which is subsequently used during ATP production through oxidative phosphorylation in the mitochondria^557^. Activation of the lactate receptor **HCAR1** inhibits lipolysis and thus the hydrolysis of triglycerides into glycerol and fatty acids^558, 559^ (its expression is inhibited by inflammation^560^). The mitochondrial oxidoreductase **MARC1** catalyzes the NADH-dependent nitrite reduction to nitric oxide (NO) under anaerobic conditions^561^, is associated with LDL cholesterol levels^562^ and binds **PARK2**^459^. Maintaining a balance between (oxLDL-generated) reactive oxygen species (ROS) and reactive nitrogen species (RNS) such as NO is important in preventing apoptosis^563^.

Plasma levels of oxidized cholesterol derivates are associated with PD. Patients have higher plasma oxLDL^177^ and 7-ketocholesterol (7-KC)^564^ (the main cholesterol oxidation product in oxLDL^565^). Statins, inhibitors of cholesterol synthesis, decrease oxysterol levels in the brain^566^ and reduce the aggregation of **SNCA** in vitro and in **SNCA** transgenic mice^567, 568^. Whereas high plasma LDL-cholesterol levels are protective, cellular LDL is oxidized in mitochondria^569^, increases ROS formation^570^ and impairs the activity of the mitochondrial oxidative complexes^571^. OxLDL consists of multiple oxysterols^572, 573^ and results in ΔΨm disruption and release of the pro-apoptotic proteins cytochrome c and HTRA2^574^. Further, high cytoplasmic free cholesterol levels are toxic^575^ and can cause mitochondrial dysfunction^416, 576^, i.e. increased mitochondrial cholesterol levels increase oxidative stress^577^, resulting in increased oxysterol formation^578^. Oxysterols can be generated by either auto-oxidation or by ER or mitochondrial cholesterol hydroxylases^579^ and regulate lipid metabolism (via **SREBF1**, see also main process C), receptor function, immune response and apoptosis^579^ and cause **SNCA** aggregation^568, 580, 581^ and lysosomal and mitochondrial destabilization^582-584^.

Mitochondrial function is affected by ER-stress-related components, e.g. BAX (see section A2.2.1) but also by cholesterol regulation in the ER. Namely, **SREBF1** inhibition or knockdown reduces cholesterol-dependent stabilization of PINK1 on the mitochondrial membrane, **PARK2** translocation to the mitochondria and subsequent mitophagy^585^. Further, the familial PD protein PLA2G6^222, 223^ catalyzes fatty acids release from phospholipids and associates with mitochondria during cholesterol-induced ER stress^586^, whereas sustained PLA2G6 activation leads to disruption of the mitochondrial outer membrane and cytochrome c release^587^.

Expression of the outer mitochondrial membrane translocator protein (TSPO) is increased in PD striatum and midbrain, and correlates with motor disease serverity^252^. It is uncertain if TSPO is part of the mPTP or only associated to this complex^578^. TSPO transports cholesterol into the mitochondria^578, 588, 589^. TSPO expression is increased by S1P^590^ and in a 6OHDA rat PD model^591^ and is activated during microglia activation^252^. In addition to TSPO, also the steroidogenic acute regulatory protein (STAR; part of the mPTP) binds cholesterol^592^ and increases cholesterol transport into the mitochondria^593, 594^. STAR expression is increased by angiotensin II^595, 596^ (not shown), NR5A1^597, 598^ (not shown), **CREM**^599^ (not shown), LDL^600^ (not shown), HDL^600^ (not shown), 27-OHC^601, 602^, S1P^590^ (not shown) and **SREBF1**^603, 604^ (not shown) and decreased by **AMPK**^605^, **SIK1**^606^, **BMP7**^607, 608^ (not shown), NF-KB^609^ (not shown), LXRA/RXRA^610^ (not shown) and **ASAH1**^611^ (not shown). Furthermore, STAR binds to the vitamin D receptor (VDR)^612^ (not shown) and **PRKG1**^613^ (not shown) and increases transport of VDR into mitochondria ^612^ (not shown). Thus, cellular lipoprotein (LDL, HDL), cholesterol-related (27-OHC, **SREBF1**) and sphingosine-related (S1P, **ASAH1**) proteins affect cholesterol uptake by the mitochondria. STAR-mediated mitochondrial cholesterol transport increases mitochondrial accumulation^614^ of the apoptotic cholesterol derivate 27-OHC^615^. 27-OHC is increased in the plasma of PD patients^564^, decreases TH expression and increases **SNCA** expression^616, 617^ and is involved in reverse cholesterol transport (see section B4).

Cholesterol transport into mitochondria is required for the formation of steroid hormones. TSPO and STAR transport cholesterol into the mitochondria for conversion to pregnenolone and eventually to testosterone^594, 618-622^ by **CYP17A1** in the ER^623, 624^. Thus, TSPO and STAR are indirectly important in production of testosterone. Testosterone decreases the expression of STAR^620^ (not shown), and thereby functions as a feedback loop on cholesterol transport into the mitochondria and subsequent testosterone synthesis, but may also increase the formation of 27-OHC and oxysterols. For more on testosterone function in PD pathogenesis see section C4.

### A3. FOXO1 and SIRT1: Transcriptional regulators of oxidative stress

The transcription factor FOXO1 mediates cellular homeostasis during oxidative stress^312^, is inhibited by INS signaling^312^ and promotes neuronal cell death^312^. FOXO1 has a key role as it is connected with many proteins in the landscape. FOXO1 binds STAT3^625^, is activated by CXCL12^626^ (not shown), its expression is increased by **TCF12**^627^ and **PIK3CD** increases FOXO1 degradation^628^. Further, FOXO1 increases expression of NF-KB^629^, **ATP6V0A1**^630^, **HUS1**^629^, SOD2^389^, INSR^631^, SCARB1^632^, the mTORC2 complex^633^ (not shown; mTORC2 consists of MTOR, RICTOR, MLST8, PRR5, MAPKAP1 and DEPTOR^312^, binds PINK1^634^, **EHMT2** and **BAT5**^635^ and increases the expression of PTEN^636^), LAMP2^632^, SIRT1^637^ and decreases the expression of **SREBF1**^632^, **ANGPT2**^638^, **PRF1**^630^ and SERPINE1^639^ and regulates CCL5 release^640^. Furthermore, deacetylated FOXO1 increases expression of ICAM1^641^. Thus, FOXO1 regulates DNA repair (**HUS1**), oxidative stress (SOD2), acidification of intracellular compartments (**ATP6V0A1**), INS metabolism (INSR), cholesterol metabolism (SCARB1, **SREBF1**), chaperone-mediated autophagy (LAMP2) and the immune response (**PRF1**, ICAM1, CCL5).

The PD-linked^226^ deacetylation factor SIRT1 is a sensor for cellular energy status and is activated by an increased NAD/NADH^+^ ratio, and is subsequently involved in regulation of cell cycle, apoptosis and autophagy, and shuttles between the cytoplasm and the nucleus^312^. SIRT1 is, like FOXO1, an important regulator of the main transcriptional pathways in the PD landscape (see below) and is involved in cholesterol homeostasis^642^. Mutations in the SIRT1 promotor that may decrease SIRT1 transcription were found in some PD patients, but not in controls^226^. Cytoplasmic SIRT1 binds mTORC1^643^, **MAPT**^644^, CTNNB1^645^, ATG5^646^, ATG7^646^, CACNA1C^513^ and **SREBF1**^647^ and binds to and activates **AMPK**^648^ (another energy sensor, activated in response to low cellular ATP levels, see section B3.1). In the nucleus, SIRT1 binds to NF-KB^649^, TP53^650^, STAT3^651^, **TLE1**^652^, AR^653^, VDR^654^, **CCAR2**^655^ (inhibits SIRT1^655^) and NEDD8^656^. SIRT1 activates PTEN^657, 658^ and **AMPK**^659^, and inhibits CASP3^660^, NF-KB^652^, SERPINE1^661^, TP53^650^ and **SREBF1**^662^. Although SIRT1 decreases **SREBF1** stability via deacetylation^662^, it also increases the expression of the cholesterol transporters ABCA1^663^ and **ABCG1**^663^ and thereby increases reverse cholesterol transport^663, 664^ (see also section B4).

Further, SIRT1 increases expression of MAOA^278^, regulates expression of SOD2^665, 666^ and decreases expression of the sodium channel **SCNN1A**^667^, STAT3^668^, TP53^651^, AR^653^ and the production of testosterone^669^.

Thus, in response to cellular energy levels, SIRT1 regulates autophagy (**AMPK**, mTORC1, ATG5, ATG7), transcription (FOXO1, STAT3, **SREBF1**, VDR, TP53), and is involved in the regulation of oxidative stress (SOD2) and apoptosis (CASP3, TP53). Finally, FOXO1 and SIRT1 tightly regulate each other. They bind^670^, FOXO1 activates^671^ and increases expression of SIRT1^637^ and SIRT1 inhibits FOXO1^672^.

### A4. Concluding remarks

The uptake, processing and signaling of lipoproteins and their components (e.g. cholesterol, sphingolipids, triglycerides) and their subsequent metabolites and derivatives (e.g. oxysterol, ceramide, sphingosine, S1P, fatty acids) appear to play a crucial role in the PD landscape. Dysregulation of cellular cholesterol levels increase oxidative- and mitochondrial stress, which - given the increased oxidative state of DA neurons (e.g. due to iron and DA metabolism) – may just tip the scales in DA neurons and result in (ER stress-induced) mitochondrial dysfunction, increased cellular stress and apoptosis. Of note, familial PD proteins are directly involved in mitochondrial membrane quality control and cellular apoptosis and are therefore located at the ‘end of the funnel’ of interactions ultimately leading to mitochondrial-mediated DA neuron death. Single sporadic variations have a lower impact on the same pathways, but when accumulated, will also lead to mitochondrial dysfunction and DA neuron death.

## B. ENDOSOMAL-LYSOSOMAL FUNCTIONING

Many of the PD-GWAS-associated proteins regulate endocytosis, autophagy and lysosomal function. This section discusses these processes and pathways in detail, starting with sphingolipids (B1), as they are important for membrane (and lipid raft) function and thus crucial for the regulation of endocytosis, autophagy and lysosomal function. Further, section B2 discusses the endocytosis of extracellular proteins and lipids into the intracellular endosome-lysosome system, and B3 elaborates on the function of the lysosome in autophagy and protein degradation. Lastly, section B4 covers the role of reverse cholesterol transport and (systemic) regulation of lipoproteins and INS in PD. The functional interactions between the proteins within process B, ‘endosomal-lysosomal functioning’, are shown in **Supplementary Figure 2**.

### B1. Sphingolipids

Sphingolipids are a class of bioactive lipids containing a hydrophobic backbone of a long-chain spingoid base that is linked to a fatty acid and an hydrophilic head group than may contain hydroxyl groups, phosphates or sugar residues. Simple spingolipids include sphingosine (sphingoid base) and ceramide (sphingosine linked to fatty acid without additional head groups). More complex sphingolipids include sphingomyelin (ceramide with a phosphocholine or phosphoethanolamine head group), cerebrosides (ceramide with a single glucose or galactose head group) and gangliosides (ceramide with at least three sugars, one of which must be sialic acid). Ceramide, sphingomyelin, sphingosine, cerebrosides and gangliosides are discussed in more detail in the next paragraphs.

#### B1.1 Ceramide and sphingomyelin

Ceramide is important for the functioning of cellular membranes and plays a role in apoptosis^673^. Further, plasma ceramide is higher in sporadic PD patients versus controls and in PD patients with versus without cognitive impairment^674^, whereas sphingomyelin is reduced in PD frontal cortex lipid rafts compared to controls^675^. In addition to the de novo synthesis from less complex molecules, ceramide can be generated through hydrolysis from sphingomyelin by the lysosomal sphingomyelinase SMPD1. SMPD1 is activated by cholesterol^676^ and induces translocation of **PRKCE** to the cytosol and NF-KB to the nucleus^677, 678^ (not shown), this is presumably resulting from a change in ceramide/sphingomyelin ratio by SMPD1, for ceramide increases cytosolic accumulation of **PRKCE**^677, 679^ (not shown) and translocation of NF-KB to the nucleus^678^. Moreover, ceramide also increases activation of NF-KB^680-683^, CASP3^684-686^, CASP9^687^ and BAX^418, 688^, inhibits AKT1^689-691^ and increases ER- Ca^2+^ and mitochondrial cytochrome C release and apoptosis^528, 692, 693^. The lysosomal glucosylceramidase **GBA** and ceramidase **ASAH1** respectively increase and decrease ceramide levels, i.e. **GBA** converts glucosyl-ceramide (a cerebroside, and also called glucocerebroside) to ceramide and **ASAH1** catalyzes the conversion of ceramide to sphingosine^694, 695^. **GBA** also binds to **PARK2**^696^ and **SNCA**^697^ and mutations in, or knockdown of **GBA**^698^ increases **SNCA** aggregation, whereas **SNCA** in turn inhibits **GBA** activity^698^.

The transporter **ABCG1** is located on the plasma membrane and increases secretion of sphingomyelin from the cell^699^. Further, sphingomyelin may be a substrate for and increases the expression of the late-endosomal/lysosomal cholesterol transporter **ABCA5**^700^ (mRNA expression is increased in the amygdala of PD patients^700^). Moreover, sphingomyelin also increases expression of **SNCA**^700^, and decreases expression of the cellular cholesterol homeostasis-controlling transcription factor **SREBF1**^701^. On the other hand, ceramide increases activation and nuclear translocation of **SREBF1**^702^, suggesting that sphingomyelin and ceramide have opposite effects on cholesterol regulation. In turn, cellular sphingolipid levels are affected by lipoproteins, e.g. HDL increases ceramide levels^703^ by binding to SCARB1^704^, and oxLDL and 7-KC can increase ceramide accumulation^705^ (not shown), but 7-KC in oxLDL can also inhibit lysosomal sphingomyelinase^706^ (not shown).

#### B1.2 Sphingosine

The sphingosine synthesis pathway is regulated by the precursor protein prosaposin (PSAP), whose uptake and transport into endosomal-lysosomal compartments^707^ and to the lysosome is regulated by its binding to LRP1^708, 709^ and sphingomyelin^710^ and to the PSAP receptor GPR37^711^. PSAP is increased by CTNNB1^712^, TP53^713^ and in the striatum by the DA transporters SLC6A3^714^ and SLC18A2^714^ (not shown). In the lysosome, PSAP binds CTSD^715^, that regulates the proteolytic processing of PSAP into saposin A, B, C and D^716^. These saposins are associated to PD-related mechanisms i.e. saposin A and B deficient mice show altered autophagy^717^, saposin A and B knockout mice show increased number of foot slips in the narrow bridge test (a behavioral test to assess motor balance and coordination) and develop a tremor^718^. Further, saposin C attenuates MPTP toxicity^719^ and binds^720^ and activates **GBA**^721-723^, whereas saposin D increases **ASAH1** activity^723, 724^. Thus, saposin C and D stimulate the conversion of glucocerebroside to ceramide (via **GBA**), to sphingosine (via **ASAH1**) respectively.

Of note, the PSAP receptor, GPR37 is associated with juvenile parkinsonism^157^, is accumulated in PD lewy bodies^156^ and its overexpression induces macroautophagy^725^. GPR37 increases ER stress and expression of HSPA5^725, 726^ (not shown) and **PARK2**^726^. GPR37 binds HSPA8^727^ and SLC6A3^301, 728^. Further, **PARK2** binds, increases the ubiquitination and increases the degradation of GPR37 and thereby prevents its aggregation and subsequent ER stress-mediated neuron death^157, 727, 729, 730^. GPR37 is involved in the expression of SLC6A3^301^ and the uptake and quantity of DA in the striatum^728, 731, 732^. So, overall, PSAP regulation is important for activation of the sphingosine synthesis pathway and in maintaining normal DA levels in the striatum.

##### B1.2.1 Sphingosine-1-phosphate

Sphingosine-1-phosphate (S1P), formed through phosphorylation of sphingosine by sphingosine kinase 1 (SPHK1)^733^, is an immune attractant (see section D2.2.2.1) and is transported out of the cell by the transporter **SPNS2**^734^. Further, S1P activates the UPR and ER stress^735, 736^ and is, similarly to cholesterol, transported by lipoproteins and involved in lipid raft functioning^590, 735-737^. Sphingosine and cholesterol metabolism are linked, illustrated by S1P activation of **SREBF1**^737^ (the cholesterol uptake proteins SCARB1 and LDLR are upregulated by S1P^590^) and the regulation of **GBA** activity by cholesterol^707^.

The S1P receptors bind and increase activation (after activation by S1P) of the G protein **GNA12** to regulate cell shape and motility^738, 739^. **GNA12** transduces extracellular signals over the membrane^312^ and also binds to CDH2^740^, **CXCR4**^741^, **LRRK2**^742^ and the cytoplasmic proteins **PRKCE**^743^ and **AXIN1**^744^. Thus, S1P activates **GNA12**^739^ and activates CTNNB1^740^, GSK3B^745^ and NF-KB^746^, increases the expression of the immune regulator **ITGA6**^747^, the diacylglycerol (DAG) activated calcium channel **TRPC6** (increasing Ca^2+^ influx)^748^ and NOS2^749^. NOS2 expression is also increased by **LRRK2**^750^, and **SREBF1**^751^ and decreased by **COL18A1**^752^ (not shown) and NOS2 itself increases the expression of perforin (**PRF1**)^753^ and IL6^312^, indicating a role in the immune response (part of main process D).

#### B1.3 Cerebrosides and gangliosides

Cerebrosides are primarily found in nervous tissue and are reduced in lipid rafts of the frontal cortex of PD patients^675^, but increased in the plasma of PD patients compared to controls^674^. Further, **GBA**, the glucocerebrosidase that converts glucocerebroside to ceramide, is downregulated in PD patients compared to controls^98, 754^. Glucocerebroside can be converted into globoside by addition of a galactose, further addition of the sugars sialic acid, N-acetylgalactosamine and galactose results in the synthesis of respectively the gangliosides GM3, GM2 and GM1. GM1 is the most common ganglioside in the brain and is involved in neuronal plasticity and repair as seen by its protective effects after a mechanical lesion of the dopaminergic nigro-striatal system^755^. Development of parkinsonistic features, i.e. motor impairment, striatal DA depletion, loss of TH positive neurons and **SNCA** aggregation were seen in mice devoid of the GM1^756, 757^. Whereas GM1 administration in mice treated with MPTP (a model for PD) resulted in partial restoration of DA neurons in the SN^758, 759^. Further, in a cell model for lysosomal disease, characterized by reduced lysosomal activity, lysosomal cytotoxicity, and inhibition of the autophagy-lysosomal pathway resulting in **SNCA** accumulation, GM1 administration reversed the phenotype^760^. Moreover, use of ganglioside GM1 by PD patients improves their motor symptoms and slows down symptom progression^761^, even over a five-year period^762^. And, anti-GM1 ganglioside antibodies are increased in the serum of PD patients compared to controls^763^. Lastly, GM1 content in the brain decreases with age, while GM3, a minor brain ganglioside, is increasing^764^. GM3 has a higher binding affinity to **SNCA** than GM1^765^ and can specifically regulate **SNCA**-induced pore formation^766, 767^ (not shown). **SNCA** membrane association, by binding to GM3 or GM1, induces folding of an alpha-helix domain that displays a high affinity for cholesterol enabling it to get inserted in a cholesterol rich part of the plasma membrane (lipid raft) and form an oligomeric ion channel^765^. In this way, **SNCA** can form pores in the neuronal plasma membrane^767^ that increase Ca^2+^ influx, increasing synaptic vesicle release and increase synaptotoxicity^768^. Of note, defects in the endocytic pathway and membrane trafficking to the lysosome results in accelerated release of exosome-associated GM1^769^ and these exosomes (extracellular vesicles secreted by the cell) containing GM1 or GM3 accelerate the aggregation of **SNCA**^770^ (not shown). Moreover, GM1 binds **MBP**^771^ (not shown) and GM3 binds the INSR^772^ (not shown) and decreases expression of ICAM1^773^ (not shown). Further, the proprotein PSAP binds GM1 and GM3 and may function as a ganglioside transport protein for transport into the cell^774^ (not shown), whereas its proteolytic cleavage product saposin B increases the degradation of GM1 in lysosomes^723, 775^ (not shown).

Thus, ganglioside content in plasma or exosome membranes, which is regulated by among others the endosomal-lysosomal pathway and PSAP, may affect membrane function, but also **SNCA** membrane association and thereby be important in PD pathophysiology.

### B2. The endosome-lysosomal system

The endosome-lysosomal system recycles and catabolizes material taken up by endocytosis, from the external milieu or from the cytosol by autophagy. The endosomal-lysosomal system regulates protein trafficking, sorting, recycling and degradation, and microtubular motor transport. Moreover, in immune cells, the endosomal-lysosomal system also functions in protein processing for antigen presentation. Its main subprocesses include clathrin- and caveolae-mediated endocytosis, the trafficking, targeting and recycling of vesicles, as well as lipoprotein uptake and processing. Particularly, the organization of membrane components in lipid rafts, such as cholesterol and sphingolipids (including sphingomyelin and ceramide), regulates the fluidity of the membrane^676, 776, 777^, and is essential for the clustering of receptor molecules, recruitment of intracellular signaling molecules^778-780^, endocytosis, membrane trafficking and activation of the immune response^781, 782^. Lipid rafts, i.e. cholesterol-rich microdomains, are located on plasma, endosomal, lysosomal, ER and mitochondrial membranes. Interestingly, oxysterols can regulate membrane fluidity^783^ and the formation of lipid rafts^777^. Further, also the familial PD protein **LRRK2** and cholesterol affect lipid raft function^784-787^. Moreover, membrane cholesterol affects the DA uptake/efflux function of the PD-associated^232-234, 788^ DA transporter SLC6A3^789-792^ that is located in both lipid rafts and non-raft membrane regions^789^ and undergoes clathrin-mediated endocytosis^793, 794^. In lipid rafts SLC6A3 binds to the PD associated^28^ and SN specific^504^ **RIT2** that regulates SLC6A3 internalization and functional downregulation^795^, and may be involved in the survival of DA neurons^504^.

The next paragraphs will discusse clathrin-independent (B2.1) and -dependent (B2.2) endocytosis and their role in cholesterol and lipoprotein uptake, vesicle trafficking and sorting.

#### B2.1 Clathrin-independent endocytosis

Clathrin-independent endocytosis occurs by caveolae, i.e. specialized lipid rafts in caveolin-1 (CAV1)-enriched plasma membrane invaginations^796, 797^. **SNCA** and **PRKCE** increase the expression of CAV1^798, 799^ and thus may promote the formation of caveolae. CAV1 binds to and increases lipid raft localization of the gap junction protein **GJB2**^800^. CAV1 also binds to PTEN^801^, INSR^802^, **SCARB2**^803^, RAC1^804^ (not shown), SCARB1^805^ (stabilization by **PARK2** prevents its degradation^806, 807^), LRP1^808^, CTNNB1^809^, JAK2^810^ and STAT3^811^, indicating that CAV1 and thus caveolae regulate the JAK2/STAT3 pathway as well as cholesterol (SCARB1, **SCARB2** and LRP1) and INS (INSR) signaling. Moreover, CAV1 increases the cholesterol content of lipid rafts^812^, decreases cholesterol esterification^813^ and increases cholesterol efflux^814^. In turn, cholesterol increases CAV1 expression^815, 816^ (not shown), indicating a complex interaction between CAV1, lipid rafts and cholesterol. Further, in lipid rafts sphingosine can be converted into sphingosine-1-phosphate (S1P) by sphingosine kinase 1 (SPHK1)^733^. S1P increases the localization of CAV1 and actin cytoskeleton-regulating proteins, such as the CAV1-binding **COL18A1** (endostatin)^817^, to lipid rafts^818^, enabling cytoskeleton regulation that is necessary for caveolae-mediated endocytosis. Defective sphingosine production (as regulated by the enzymes **GBA** and **ASAH1**) can therefore have major effects on lipid raft function and (caveolae-mediated) endocytosis by the cell. The CAV1-binding^819, 820^ microtubule-associated protein DNM2 mediates endocytosis and vesicle budding in caveolae^821^ and further binds to **CACNA1A**^505^, **MCC**^387^, **AMPH**^822^, **AMPK**^387^ and DRD2^823^, and binds to and colocalizes with the myosin motor protein **MYO1E**^824^ that is involved in freshly-budded vesicle trafficking. The caveolae-localized potassium voltage-gated channel subunit Kv1.5 (**KCNA5**) is regulated by angiotensin II^825^, cholesterol, sphingolipid and oxLDL^825-827^ and linked to apoptosis^828^. And lastly, the transcription factor **RFX4** heterodimerizes with RFX3^829^, a transcription factor that binds to the promotor of **DNAH11**^830^. **DNAH11** is a protein that is involved in microtubule motor activity and genetically associated with LDL levels^831^.

#### B2.2 Clathrin-dependent endocytosis

Clathrin-mediated endocytosis is regulated by the clathrin- and dynamin-binding protein amphiphysin (**AMPH**)^822, 832-837^ and by the adaptor protein 3-complex (**AP3**, subunit **AP3B1**) that binds and sorts proteins to endosomes and lysosomes^838^. Association of the endocytic clathrin-coat with the actin cytoskeleton is regulated by the genetically PD-linked HIP1R^77, 839, 840^. The myosin motor protein **MYO1E** (required for actin assembly during clathrin-mediated endocytosis)^841^, **AMPH**^842^, but also the cytoplasmic actin-bundling and calcium-sensitive protein fimbrin (**PLS1**)^843^ and RAC1 (necessary for actin polymerization during endocytic clathrin-coated pit formation)^844^ are involved in actin formation during endocytosis, whereas the cytoplasmic monooxygenase **MICAL2** promotes depolymerisation of F-actin^312^. RAC1 binds to and is activated by the familial PD proteins **LRRK2** (changes the cellular localization of membrane-bound RAC1)^845^ and **PARK2**^846^, the Rho GTPases **ARHGAP33** and **ARHGAP44**^312, 847, 848^, binds to the nuclear importin **KPNA4**^848^ (not shown), and is activated by **RAP1A**^849^, **RIT2**^850^ and **NEDD9**^851^ and inhibited by PTEN^852^.

In addition to endocytosis, clathrin is also used for protein sorting towards lysosomes^853, 854^. The clathrin-binding proteins **CLVS2** and **TOM1L2** are required for normal endosome/lysosome morphology^855^ and protein trafficking to the lysosome^856^ respectively. Of note, the ubiquitin ligase NEDD4 promotes degradation of **SNCA** by the endosomal-lysosomal pathway and is located in lewy bodies^857, 858^. NEDD4 binds to **SMAD5**^859^ (not shown), **RAP2A**^860^, **ZAK**^861^ (not shown), **GBA**^861^ (not shown), **TOM1L2**^861^, **MRPL19**^861^ (not shown) and binds and increases mono-ubiquitination of **DCUN1D1**^862^ (not shown).

To uncoat clathrin-coated vesicle – necessary for fission with the target membrane – DNAJC6 or its homologue **GAK** (also referred to as auxilin 1 and 2, respectively) recruit the (in the PD SN downregulated^171^) clathrin uncoating ATPase Hsc70 (HSPA8)^863-865^. Both **GAK** and HSPA8 bind to the familial PD lysosomal protein ATP13A2^866^ (found in the lewy bodies of remaining PD DA neurons^867^), which deficiency leads to lysosomal dysfunction and **SNCA** aggregation^868^. Further, depletion of either **GAK** or HSPA8 inhibits clathrin-mediated endocytosis^869^ and the **GAK**-**LRRK2** complex promotes golgi-derived vesicle clearance through the autophagy–lysosome system^870^.

##### B2.2.1 Vesicle trafficking and recycling

**PCM1** and **FAM190A** are both involved in early vesicle trafficking, by binding the dynein-mediated organellar transport regulator NDEL1, the dysfunctioning of which results in delayed endocytic-lysosomal compartment formation^871^. Further, the lysosomal trafficking protein **VPS41** binds the **AP3**-complex^872, 873^, reduces **SNCA** accumulation and caspase activation, and is protective against **SNCA** overexpression and the neurotoxins 6-OHDA and rotenone in PD models^874^. Lysosomal trafficking by **AP3B1** (**AP3**-complex)^875^ includes the membrane protein **SCARB2**^876^ that regulates lysosomal targetting of **GBA**^877^, enabling **GBA** to convert glucocerebroside into ceramide (see section B1). Further, Localization of **LAMP1** (lower expressed in DA neurons in the PD SN^34^) to the endosomal / lysosomal membrane is regulated by **AP3**^875, 878^. **LAMP1** decreases expression of LAMP2^879^ and both **LAMP1** and LAMP2 regulate cholesterol traffic and decrease cholesterol accumulation^880^.

Recycling of (endosomal) vesicles is regulated by multiple proteins in the landscape. The familial PD protein^260^ VPS35 is part of the retromer complex for endosome/trans-golgi network transmembrane receptor recycling and the sorting of cargo proteins^881, 882^, e.g. for sorting of the lysosomal acid protease cathepsin-D (CTSD)^883^. The retromer complex interacts with the PD-linked DNAJC13^148^, an endosomal recycling component regulating early-endosome clathrin-coat dynamics^148^. DNAJC13 binds to GSK3B^884^ and HSPA8^885^. Further, **RER1** regulates retrograde vesicle-mediated transport of proteins from the golgi to the ER^886^. **RER1** binds the γ-secretase complex (by bind to e.g. PSEN1) and thereby increases the retention and retrieval of this complex and its subunits in the ER, preventing γ-secretase activity^887-889^ (for more on the γ-secretase complex see section C2.4).

The membrane fusion protein **NSF** is required for endocytic vesicle/golgi fusion^890^, vesicle-mediated ER-golgi transport^891^, clathrin-coated vesicle/target membrane fusion^892, 893^, thus regulating endocytic recycling^894^ and exocytosis^895^. **LRRK2** binds to **NSF** (thus controling vesicle recycling)^896^ and interacts with **SNCA** (^406, 897, 898^ that inhibits vesicle recycling^899^. The cytoplasmic protein **SYN3** localizes to the membrane of cytoplasmic vesicles and decreases release of DA in the striatum^900^ and is downregulated by **SNCA**^901^. **FER** also binds to and phosphorylates **NSF**, thus inhibiting subsequently vesicle fusion^902^. **NSF**-attachement protein beta (**NAPB**) helps **NSF** binding to the SNARE complex^903^ and is thus involved in vesicle fusion and exocytosis. Other exocytosis-regulating proteins are **RPH3AL**^904^ (involved in INS secretion^905^) that binds to **UNC13B**^312, 906^ and the ER-stress-induced^907^, CCL5-regulating^908^ clathrin-coated vesicle protein^909^ **SCAMP5**^908^. The PD associated protein SYT11^76, 77, 104^ regulates Ca^2+^-dependent exocytosis of secretory vesicles^312^ (the Ca^2+^-dependent protein **SCIN**^312^ also regulates exocytosis by regulating the actin cytoskeleton during exocytosis^502^). SYT11 binds ATP13A2^866^ (not shown) and **PARK2**, that also increases SYT11 degradation^910^. The early-onset Parkinsonism-associated SYNJ1^240, 241^ is involved in synaptic vesicle recycling^240^ and components of this machinery, i.e. it binds to **AMPH**^911^, **MYO1E**^824^, **PARK2**^912^, clathrin^913^ and the calcium-channel subunit **CACNA1A**^505^. The recycling endosome membrane protein **RAP2A**^914^ binds to the mTORC1 complex^915^ and the **SNCA**-binding phosphoprotein **ENSA**^916-918^. Dysfunctioning of the **AP3** complex regulator **AGAP1** (that binds **AP3** and regulates **AP3**-dependent trafficking)^919^, affects striatal DA release, linking endocytic recycling to DA release^919, 920^.

##### B2.2.2 Lipoprotein uptake and processing

Lipoproteins are composed of lipids, triglycerides and proteins. Very low-density lipoprotein (VLDL) particles are enriched in triglycerides and the removal of triglycerides from VLDL results in the formation of intermediate density lipidprotein (IDL)^921^. IDL is enriched in cholesterol and can subsequently be converted by **LIPC** into LDL^312^, which is highly enriched in cholesterol. The clathrin-mediated endosomal-lysosomal system facilitates lipoprotein (e.g. VLDL, LDL) uptake via (among others) the VLDL receptor (VLDLR) and LDL receptor (LDLR)^312^. Early endocytic vesicle acidification by the V-ATPase (**ATP6V0A1**) releases LDL from the LDLR. LDL is then degraded in the lysosome, and cholesterol is salvaged for cellular use^922^ and increases the expression of the transporter **ABCG1**^923-925^ that subsequently exports cholesterol to HDL^926, 927^ and thereby also inhibits plasma membrane lipid raft formation^928^. LDLR expression is in turn increased by **PRKCE**^929^ and its activation inhibited by **MAP2K6**^930^ that is activated by **LRRK2**^931^. Uptake of IDL and HDL is regulated by the receptors SCARB1 (upregulated by FOXO1)^632^ and **SREBF1**^932^, and downregulated by **ASAH1**^590^ and LRP1 respectively, both of which are affected by the extracellular matrix (ECM) lipase **LIPC**^933-935^. **LIPC** deficiency increases serum HDL-cholesterol^936^. The expression of the APOE receptor LRP1 (binds to **SERPING1**^708^) and APOE itself is increased in early PD^117^, which may indicate a defect in lipoprotein regulation in early PD.

The ER serine protease **TMPRSS3**, the plasma membrane serine protease **TMPRSS9**, the anti-apoptotic protein^937, 938^ **RXFP1**, the pore forming complement system member **C9**, and the lipoprotein uptake receptors VLDLR, LRP1 and LDLR all contain a LDLR class A domain that can bind LDL^312^.

### B3. The lysosome-autophagosomal system

#### B3.1 AMPK and mTORC1 regulated autophagy

The AMP-activated protein kinase (**AMPK**)-complex and the mTORC1-complex are both essential for autophagy functioning^939, 940^. The mTORC1-complex (composed of MTOR, RPTOR, MLST8, AKT1S1 and DEPTOR) is a nutrient sensor that controls protein synthesis for cell growth and proliferation and is activated by INS, growth factors and oxidative stress^941, 942^. mTORC1 is involved in DA neuron survival, whereas the mTORC1 inhibitor rapamycin is neuroprotective in *in vitro* and *in vivo* PD models^943^ and blocks translation of the MTOR inhibitor DDIT4, a protein that is elevated in PD SN neurons and mediates cellular death in PD models^147^, indicating that inhibition of only certain mTORC1 functions is beneficial for neuronal survival. Of note, prolonged treatment of PD patients with the DA precursor L-DOPA results in L-DOPA-induced dyskinesia, which is associated with persistent activation of mTORC1^944^.

The **AMPK**-complex – consisting of an α-subunit (**PRKAA1** or PRKAA2), a β-subunit (PRKAB1 or PRKAB2) and a γ-subunit (PRKAG1, PRKAG2 or PRKAG3)) – is a cellular energy sensor that is activated when intracellular ATP levels are low^940^ and subsequently tries to restore cellular energy (ATP) levels by stimulation of fatty acid oxidation, glycolysis, glucose uptake and ketogenesis and inhibition of synthesis pathways for proteins, glycogens, fatty acids and cholesterol^945-948^. **AMPK** activation results in increased oxidative stress, release of mitochondrial cytochrome c and mitochondrial caspase pathway activation^949^. The mTORC1-complex is regulated by **AMPK**, i.e. **AMPK** binds mTORC1^950^ and inhibits mTORC1 activation^940, 950^, thereby preventing mTORC1-mediated autophagy inhibition^940^ and mTORC1-mediated **SREBF1** activation^951^. Following mTORC1 inhibition (by **AMPK**), autophagosomes form^952^ and fuse with the lysosome to degrade their cargo^953^. The PD-associated proteins ATG5 en ATG7^120, 121^ are involved in autophagosome assembly^954^. ATG5 binds to the familial PD protein UCHL1^387^ and SIRT1^646^, activates JAK2^955^, and is essential in T- and B- lymphocyte survival and proliferation^312^ (not shown; part of main process D). Both ATG5 and ATG7 are involved in mitochondrial quality control following oxidative damage^312^. ATG7 increases autophagy^956^, decreases **SNCA**^957^ and **LRRK2**^957^ aggregation, activates CASP3^958^, JAK2^955^ and STAT3^959^ and, like ATG5, binds to and is deacetylated by SIRT1^646, 960^. In a PD mouse model, conditional deletion of ATG7 results in age-related loss of DA neurons and loss of striatal dopamine^961^. Together, these results indicate that proper autophagy is important to prevent DA neuronal loss and PD.

In addition to inhibition by **AMPK**, mTORC1 activity is also inhibited by **ULK2**^962^, GSK3B^963^, inhibited and decreased by PTEN^964, 965^ and FOXO1^633, 966^ and activated by **ATF6**^967^ and INS^968, 969^. mTORC1 in turn inhibits GSK3B^970, 971^ and CASP3^972^, increases the expression of PTEN^636^, activates and increases the nuclear expression of **SREBF1**^973, 974^, regulates **BAMBI**^975^ (not shown), binds the deubiquitinase **USP9X**^976^, the NAD-dependent deacetylases SIRT1^643^ (not shown) and SIRT7^394^, the transcription factor EIF4E977 and binds and phosphorylates the repressor of translation initiation **EIF4EBP2**^977, 978^. Hypophosphorylated **EIF4EBP2** competes with the familial PD protein EIF4G1 to interact with the translation initiation factor EIF4E^312, 979^. Thus, mTORC1 favors the binding of EIF4E to EIF4G1^979^, which results in recognition of the mRNA cap and initiation of translation^312, 980^ (e.g. **LSM7** mRNA translation^314^) (part of main process C).

STK11 is a master upstream kinase that increases the activity of **AMPK** and **AMPK**-related kinases^981^. Deacetylation of STK11 by SIRT1 increases STK11 activation and subsequent **AMPK** activation ^982^. In addition, STK11 also binds **AMPK**^983-987^, the kinase **SIK1**^988, 989^, **SNRK**^990^ (a distant **AMPK** relative^981^), **PARD3**^991^, GSK3B^992^, PTEN^993^, inhibits MTOR^994^, increases the expression of PTEN^995^, **TIAL1**^995^ and **NCAM2**^995^, decreases the expression of **CNTN1**^995^ (**CNTN1** binds **SNCA**^461^) and binds and activates both TP53^996, 997^ and MARK2^988, 989^. MARK2 is an **AMPK**-related kinase^981^ that binds to **AMPK**^998^, **CEP85L** ^999^, **SNCA**^461^, **CACNA1A**^505^, binds and activates PINK1^1000^ and is inactivated by **PARD3**^1001^.

Further, **AMPK** also binds to and inhibits NF-KB^1002, 1003^, binds to **SIK1**^998^ (inhibits nuclear **SREBF1**^1004^), **SNRK**^998^ and **SND1**^954^, regulates **BAMBI**^975^ (not shown) and binds to, inhibits and increases expression of SIRT1^648, 1005, 1006^. Furthermore, familial PD proteins VPS35^387^ and EIF4G1^387^ directly bind to **AMPK**, while **LRRK2** (mutations in **LRRK2** have been associated with autophagy impairment^1007^) activates **AMPK**^1008^, indicating that altered **AMPK** function may be important in PD. The serine/threonine-protein kinase and **AMPK** inhibitor^1009^ **ULK2** is involved in autophagy and is activated following phosphorylation by **AMPK**^1010^. **ULK2** is also both a downstream effector^952^ and a negative regulator of mTORC1 signaling^962^ and **ULK2** expression is increased by **SREBF1**^1011^. Thus, **AMPK** and **SREBF1** regulate autophagy and mTORC1 activation via regulation of **ULK2**. Moreover, **ULK2** functions as a negative feedback loop for both **AMPK** (directly) and **SREBF1** (via inhibition of mTORC1).

Lastly, after Ser473-phosphorylation, AKT1 activates the mTORC1 activator **SAMD4A**^1012^, that is necessary for mTORC1 activation. In control brains, AKT1 and Ser473-phosphorylated AKT1 are expressed at high levels in DA neurons in the SN, whereas PD patients show diminished brain levels of both total and Ser473-phosphorylated AKT1^113^. In contrast to neuronal loss of AKT1 in PD, both phosphorylated and unphosphorylated AKT1 are increased in glia cells in the PD brain SN^113^. Further, AKT1 Ser473-phosphorylation is increased by the familial PD proteins PARK7 (by binding and inhibiting the negative AKT1-regulator PTEN)^1013, 1014^, PINK1 (by activating the mTORC2-**PRKCE**-AKT1 pathway)^634, 1015^ and **LRRK2** (that also binds to AKT1)^1016^. AKT1 is also activated by **PRKCE**^1017^ (binds **SNCA**^532^), **PIK3CD**^1018, 1019^, inhibited by **PTPN1**^1020, 1021^ (which is again inhibited by AKT1^1022^) and bound and inhibited by the **LRRK2** phosphatase PPP1CA (**PP1**)^1023, 1024^.

In summary, activation of the mTORC1 complex is regulated by multiple proteins in the PD landscape, either indirectly (via **AMPK** and AKT1) or directly (via DDIT4, **ULK2** and **SAMD4A**). mTORC1 subsequently regulates autophagy, cholesterol homeostasis (via **SREBF1**), apoptosis (via CASP3) and translation (via EIF4E, EIF4G1 and **EIF4EBP2**).

#### B3.2 Lysosomal acidification

Lysosomal function and autophagy require organellar acidification that involves **PLEKHM1** via its Rab7-binding domain RUN^1025, 1026^. The PD-associated Rab7-like protein **RAB7L1**^102, 106^ interacts with **LRRK2** to modify intraneuronal protein sorting^1027^ and binds to the pre-mRNA splicing factor **LSM7**^1028^. The familial PD lysosomal ATPase ATP13A2^1029-1031^ is decreased in PD SN^867^ and its dysfunctioning leads to lysosomal membrane instability, reduced processing of lysosomal proteins, diminished degradation of lysosomal substrates, reduced clearance of autophagosomes and impaired lysosomal acidification which subsequently may contribute to the formation of Lewy bodies, a hallmark of PD^867, 1007^. **ATP6V0A1** is a subunit of the V-ATPase, a proton pump essential for lysosomal acidification^1032^, and binds to **SNCA**^461^. Lysosomal V-ATPase activity is regulated by **DRAM1**^1033^, which is activated by TP53^1034^, and activates the lysosomal acid protease cathepsin-D (CTSD)^1035^ (lower expressed in PD SN neurons)^33^ and autophagy following mitochondrial dysfunction^1033^. CTSD is necessary for lysosomal protease activity, but e.g. also for proteolytic cleavage of PSAP into saposin C and D, peptides that activate **GBA** and **ASAH1** (see also section B1.1.2). Conversion of ceramide into sphingosine and *vice versa* by **ASAH1** in the lysosomes is pH-dependent^544, 1036^. Therefore, lysosomal pH, controlled by the proton V-ATPase (including subunit **ATP6V0A1**), affects celllular ceramide and sphingosine levels, that may affect cellular membrane regulation and apoptosis (see section B1.1). Of note, lysosomal acidification (and thus stability) is also dependent on lysosomal cholesterol membrane content^1037, 1038^, again illustrating the importance of proper cholesterol regulation for normal lysosomal functioning.

#### B3.3 (Neuro)melanin regulation

PD is characterized by selective death of SN DA neurons containing neuromelanin (NM), suggesting involvement of NM in PD pathogenesis. There is an age-related increase in NM in the SN^1039^, and NM production, through oxidation of DA, increases oxidative stress and lipid peroxidation (see section A1.1). NM is cytotoxic^419, 1040-1042^ and results in collapse of mitochondrial transmembrane potential, cytochrome c release and CASP3 activation^419^. Therefore, DA neurons need an optimal autophagy and lysosomal function to store NM and prevent cytoplasmic NM-toxicity^1040^. Moreover, cellular increase in NM might eventually interfere with the endosomal-lysosomal pathway and lysosomal function^1040^. Release of NM in the ECM, e.g. as a consequence of DA neuron death, increases immune cell activation^1043, 1044^ (see also section D2.2). Nevertheless, due to its ability to chelate ferrous iron^338, 339^ and free radicals, NM may in addition to its toxic properties also have neuroprotective functions^1043, 1044^.

Of note, NM has the ability to absorb lipids, e.g. cholesterol^1045^ and associates with **SNCA**^1046, 1047^ that itself contains two cholesterol-binding domains^1048^. Further, **SREBF1** increases the production of both cholesterol and isoprenoid dolichol^1049, 1050^, both lipid components of neuromelanin^1051^. This indicates that there may be a complex interaction between NM, **SNCA** and lipid accumulation in DA neurons. Dysregulation of either NM, **SNCA** or, for example, cholesterol may increase their aggregation and cytotoxicity.

Proteins regulating the melanin producing melanocytes in the periphery, like MC1R, MITF, **MREG** and TYR, might also be involved in the regulation of neuromelanin in DA neurons. Of note, mutations in the melanocyte-stimulating hormone receptor MC1R may increase the risk for PD^1052, 1053^ and MC1R binds to the DA neuron determinant^1054^ **MSX1**^1055^ (not shown, see also section section D1.1). Activation of MC1R triggers transcription of MITF^1056^, a transcription factor involved in melanocyte development and melanin production^1057^. MITF expression is increased by cholesterol^1058^ and decreased by STAT3^1059^. Further, MITF is activated by GSK3B^1060^, cleaved by CASP3 (this cleavage is essential in apoptosis of melanocytes)^1061^ (not shown), binds to CTNNB1^1062^ and STAT3^1063^ and increases the transcription of **ASAH1**^1064^, SCARB1^1064^, **PRF1**^1065^, **MBP**^1064^, **COL2A1**^1066^ (also increased by **MSX1**^1067^ and **KDM2B**^1068^), MC1R^1069^ (binds to PTEN^1070^ and its expression is decreased by retinoic acid^1071^ (not shown)) and the melanin producing^1072^ TYR^1064, 1073^. Subsequently, the lysosomal maturation protein melanoregulin (**MREG**) is involved in the transfer of melanin-containing melanosomes from melanocytes to keratinocytes, and as such drives skin and hair pigmentation^1074-1076^. **MREG** dysfunction results in an increased secretion of CTSD^1077^. The **AP3**-complex-regulated^1078, 1079^ membrane protein **OCA2** is involved in melanin synthesis^1080^ and regulates trafficking of TYR^1081^ that is mediated by the **AP3**-complex^1082^. Further, **OCA2** binds to the familial PD protein ATP13A2^866^ (ATP13A2 inhibits **SNCA** aggregation^868^) and loss of **OCA2** disrupts the unfolded protein response (UPR) and increases resistance to ER stress in melanocytes^1083^. TYR mRNA and protein are expressed in the SN^1084-1086^ and increased TYR levels are toxic for DA neurons and can exacerbate the toxic effect of mutant **SNCA**^1086^. Of note, decreasing hair color darkness is associated with an increased PD risk^1052^ and familial grey hair frequency is higher in PD patients^1087^, underscoring a possible association between peripheral melanin regulation (e.g. by MITF, MC1R, **MREG** and TYR) and PD.

##### B3.3.1 Vitamin D3

Active vitamin D3 (calcitriol) increases melanocyte maturation, inhibits their proliferation and increases TYR activity (by inducing MITF expression^1088^) and melanin production^1088, 1089^. Vitamin D3 is synthesized from a cholesterol precursor in the skin under influence of UV light^1090^. Vitamin D3 is lower expressed in PD patients^1090-1092^ and its supplementation may stabilize PD for a short period^1093^. Vitamin D3 regulates the expression of PPP1CA^1094^ (not shown) and **NEDD9**^1094^ (expression decreased by **ZBTB20**^1095^), increases the expression of **HPGD**^1096^, **FBN1**^1094^, **GCH1**^1097^, PLAT^1098^, GDNF^1099^ and SERPINB9^1100^ and decreases the expression of **ANGPT2**^1101^, **IL2RA**^1102^, **BMP7**^1103^ (not shown), **LTBP1**^1094^ and **SREBF1**^1104^. Vitamin D3 also activates JAK2^1105^, inhibits SERPINE1 expression by inhibiting NF-KB activation^1106^ and inhibits the immune response by reducing the production of CCL5^1107^ and IL8^1107^, by inhibiting NF-KB-mediated IL12 expression^1108^ and IFNG-activation of macrophages^1109^.

The vitamin D3 receptor (VDR) is a nuclear transcription factor that is associated with PD^257-259^ and binds to **ACTN4**^1110^, the transcription factors **POU2F1**^1111^ and **RUNX3**^1112^ and the transcription regulators SIRT1^654^ and **MED13** (also activates VDR)^1113^. The VDR is transported into the mitochondria by the mPTP^612^ and its expression is increased by **MAP2K6**^1114^. The active vitamin D3, binds to the VDR and thereby regulates gene expression in the nucleus of the cell. The VDR (bound by vitamin D3) inhibits **CREM**^1117^ and decreases the expression of the immune response-associated proteins CCL5^1115^ and ICAM1^1116^.

Thus, vitamin D3 and its receptor VDR regulate transcription (via NF-KB, **POU2F1**, SIRT1, **SREBF1** **RUNX3**, **MED13**), pigmentation (via MITF, cholesterol homeostasis (via **SREBF1**), coagulation (via **BMP7**, **LTBP1**, SERPINE1, PLAT), and the immune response (e.g. via JAK2/STAT3, NF-KB, CCL5, ICAM1). Moreover, as vitamin D3 and its receptor VDR can regulate production of melanin in the skin, they might also affect neuromelanin production and indirectly DA production in DA neurons in PD as indicated by decreased DA neuron death^1118^ and partially restored TH expression^1099^ by vitamin D3 in PD models.

#### B3.4 Chaperone-mediated autophagy

Chaperone-mediated autophagy (CMA) is the targeting of cytosolic proteins to the lysosomal membrane by chaperone HSPA8. The HSPA8-substrate complex binds to the lysosomal-associated membrane protein type 2A (LAMP2), so they can be translocated over the lysosomal membrane and subsequently degraded^1119^. LAMP2 is reduced in peripheral leukocytes of PD patients^192^ and both LAMP2 and HSPA8 are reduced in the PD SN^171^. LAMP2 is degraded in cholesterol-rich lipid rafts^1119, 1120^. Cholesterol depletion of lysosomal lipid rafts therefore enhances CMA activity, whereas lysosomal cholesterol loading reduces CMA activity^1119, 1120^. LAMP2 also decreases cholesterol accumulation^880^ and its reduced expression may thus affect cholesterol regulation in PD patients. Moreover, reduced levels of LAMP2 and HSPA8 affect MHC class II molecules^1121^ and therefore immune cell activity. Of note, the lysosomal membrane protein **LAMP3**, is involved in adjusting lysosomal function after the transfer of peptide-MHC class II molecules to the surface of dendritic cells^1122^ and increases autophagy^1123^.

HSPA8 also binds to (and increases degradation of) ATP13A2^866^, **LRRK2**^1124^, **SNCA**^918, 1125^, **PARK2**^727, 1126^, UCHL1^1127^, **MAPT**^1128^, TP53^1129^, AKT1^1130^, **AMPK**^1131^, the NF-KB-complex subunits REL, RELA, RELB and NFKB1^1132^, **LIPC**^1133^, NEDD8^1134^ (is associated with PD lewy bodies^200^, binds **RPL7A**^1135^ and UCHL1^846^ and binds **PARK2** and PINK1 and increases their ligase activity and stabilization respectively^200^), DNAJC6^1136^, **FBXO25**^395^ (not shown), **HLA-DRA**^1137^, AR^1138^, **AP3**-complex^1139^, **BAG6**^1140^, JAK2^1141^, **GAK**^863, 1142^, MAP3K7^1132^, GSK3B^1131^, **CCAR2**^1143^, BAX^1144^ (not shown) and LAMP2^1145^. LAMP2 also binds to UCHL1^288^, TP53^1146^ and **SNCA**^1147^, and its expression is increased by FOXO1^632^ and the **AP3**-complex^875^ (not shown) and is mediated by the NF-KB-complex^1148^ (not shown). Thus, HSPA8 and LAMP2 bind to at least five familial PD proteins and interacts with several key landscape proteins. Overall the CMA proteins HSPA8 and LAMP2 are required for a normal degradation of (aggregated) **SNCA**^1149, 1150^. This notion is underscored by mutant **LRRK2**^1124^ and mutant UCHL1^288^ that both inhibit CMA, which in turn results in increased **SNCA** aggregation^288, 1124^. Of note, in contrast to the familial PD proteins **LRRK2** and UCHL1, mutant TP53 is normaly degraded by CMA^1146^.

### B4. Reverse cholesterol transport

Reverse cholesterol transport is the routing of excess cholesterol and oxysterols back to the liver and the subsequently excretion from the body. Several key components of the landscape are involved in these processes, which contribute to cholesterol homeostasis. First, in the liver, the familial PD protein **PARK2** is a lipid-responsive regulator of fat uptake and thereby increases hepatic fat uptake^806^. Second, the neuronal ATP-binding cassette transporters **ABCA3** and **ABCA5** (located on lysosomal/late endosomal membranes^1151-1153^) and **ABCG1** (located on ER, golgi, late endosome and plasma membrane^1154-1156^) are involved in reverse transport, by transporting cholesterol into the lysosomes (**ABCA3**)^1152^ and increasing cholesterol efflux to HDL (**ABCA5** and **ABCG1**)^926, 927, 1153^. Of note, the prospective studies that found association of low plasma LDL-cholesterol and low total cholesterol with increased PD risk^552-554^ and the positive correlation between PD disease duration and plasma HDL-cholesterol^375^, indicate that lipoprotein-cholesterol levels are affected in PD, which may be due to dysregulation of reverse cholesterol transport regulated by these transporters and **PARK2**.

PD patients have higher plasma oxLDL^177^ and 7-KC^564^, the main cholesterol oxidation product in oxLDL^565^. Statins, inhibitors of cholesterol synthesis, also decrease oxysterol levels in the brain^566^ and reduce the aggregation of **SNCA** in vitro^567^ and in **SNCA** transgenic mice^568^. Statin use has been associated with lower PD risk^1157, 1158^ and even prospective studies found lower PD risk when using statins^1159, 1160^. However, statin use has also been reported not to affect PD risk^1161-1164^, and it is unclear if the PD risk lowering is due to statin use, or merely due to high baseline cholesterol levels of these statin users. The only prospective study, that did took baseline cholesterol levels before statin use and during PD treatment into account, showed association between statin use and *higher* PD risk^554^.

OxLDL increases expression of **ABCA5** and **ABCG1**^1153^. **ABCG1** protects against oxLDL-induced apoptosis by promoting efflux of 7-KC to HDL^565, 1165^, as 7-KC activates apoptotic cascades by increasing the amount of cytosolic BAX^1166, 1167^, activating CASP3^1167^ and increasing the expression of TP53^1167^. Further, 7-KC inhibits AKT1^1168^, induces ER stress by increasing the expression of the **ATF6**-dependent ER stress chaperone HSPA5^967, 1166^ (not shown), increases the expression of ICAM1 and **ITGAL** on microglia^1169^ and is involved in inhibition of inflammatory responses^1170^. Thus, maintaining low intracellular 7-KC levels by the regulation of 7-KC efflux by (among others) **ABCG1** is therefore important in prevention of ER stress, the activation of apoptotic and regulation of immune responses.

As opposed to cholesterol, its derivates hydroxycholesterol (24-OHC) and 27-hydroxycholesterol (27-OHC) can cross the blood brain barrier. 24-OHC originates primarily from the brain and is the main cholesterol elimination product of the brain^1171^. 27-OHC is the major oxysterol in the circulation that can cross the blood brain barrier and has under normal physiological conditions a steady influx into the brain^1172^. Both 24-OHC and 27-OHC levels are associated with PD, i.e. 24-OHC levels in the CSF of PD patients correlate with the duration of PD^376^, whereas 27-OHC is increased in the plasma of PD patients^564^. An increased 27-OHC flux into the brain, e.g. due to hypercholesterolemia, potentially has implications for PD pathogenesis, as 27-OHC decreases TH and increases **SNCA**^616, 617^ expression. Healthy men with low HDL-cholesterol have a high 27-OHC/total cholesterol ratio, indicating that the production of 27-OHC and its transport to the liver may represent an alternative pathway for reverse cholesterol transport by HDL^1173^.

Further, high 24-OHC and 27-OHC concentrations induce apoptosis of neuronal cells^616, 1174^ (low 27-OHC concentrations trigger survival and high concentrations apoptosis^615^) and should therefore be thighty controlled. One of these control mechanisms is the transport of 24-OHC out of the cell to HDL by the transporter ABCA1^1174^. 24-OHC increases the expression of ABCA1, but also that of **ABCG1**^1175^ and decreases the expression of LDLR and **SREBF1**^1175^. 27-OHC also increases the expression of ABCA1^1176, 1177^ and **ABCG1**^1177^ and is involved in the expression of **SREBF1**^1178, 1179^ (not shown). Thus, 24-OHC and 27-OHC increase the export and decrease the uptake of cholesterol by the cell.

In addition to regulation of cholesterol homeostasis, 24-OHC and 27-OHC both increase activation of STAT3 and increase the expression of the angtiotensin II receptor **AGTR1** via the liver X receptors (LXR)^377^ (not shown), indicating that there is a relation between oxysterol regulation and the brain renin-angiotensin system, that is again known to interact with the DA system (see section A1.3). 24-OHC and 27-OHC are both endogenous activators of the LXR^1178, 1180^. LXR, having 2 isoforms, LXRA and LXRB^312^ is activated by 27-OHC in response to cholesterol overload in the cell^1178^ and subsequently inhibits the production of IL1B and IL6 and the inflammatory response of microglia and astroglia^1181^ (part of process D). 27-OHC also induces production of TNF from macrophages^1182^ (not shown), a factor involved in T cell activation (part of process D), and the LXR also activates **SREBF1**^1183^ and redistributes **ABCG1** to the plasma membrane where it transports cholesterol out of the cell^1154^. Further, administration of an LXR agonist prevented DA neuron loss in a PD mice model, whereas knockout of LXR-beta increased damaged to the DA neurons in the SN. Interestingly, LXR-beta was not expressed in the DA neurons, but in the microglia and astroglia of the SN, indicating that LXR-beta activation inhibits activation of the microglia and astroglia in the SN^1184^. Thus, LXR is a mediator of cholesterol homeostasis in the cell, and involved in immune cell regulation that is critical for DA neuron survival.

In summary, oxysterols and cholesterol oxidation products are tightly regulated in the brain, any disturbance in the levels of 7-KC, 24-OHC and/or 27-OHC can therefore induce immunological or apoptotic responses that decrease the viability of DA neurons. More precisely, **ATF6**, **SREBF1**, STAT3, **AGTR1** and expression of TH and **SNCA** itself are regulated by cholesterol derivates, showing that they are regulating the main cascades in the PD landscape and fullfil a very important modulating role in PD.

#### B4.1 Fat uptake and insulin (INS)

Diabetics show an increased risk of developing PD^1185^, and the frequency of diabetics among PD patients is higher than normally expected^1087^, suggesting an influence of glucose metabolism on PD pathogenesis. Indeed, INS, is increased by the familial PD protein PLA2G6 (increases INS secretion; not shown)^1186, 1187^, whereas the ER protein **TMX1** increases cleavage of INS^1188^ and **ZBTB20** increases INS blood levels^1095^ (not shown). INS itself has effects on many components of the PD landscape. First, it activates JAK2^1189^, **ACTN4**^1190^ (not shown), mTORC1^968, 969, 978, 1191^, TH^1192^ and **SREBF1**^1193^ and inhibits **AMPK**^1194^. Furthermore, INS decreases expression of FOXO1^1195^, PTEN^1196^ and CCL5^1197^ and increases expression of **AGTR1**^1198^, **COL18A1**^1199^, **CYP17A1**^1200, 1201^, SCARB1^1202, 1203^ (not shown), the thyroid peroxidase **TPO**^1204^ (not shown; binds heme^312^), **NTF3**^1205^, **MBP**^1206^, **FER**^1207^ (not shown), **ITGA6**^1208^ and **SREBF1**^1201, 1209^. In addition to this, INS increases degradation of ABCA1^1210^ and increases binding of **FER** and STAT3^1207^ and binding of STAT3 to the ICAM1 promotor^1211^ (not shown). Moreover, INS increases binding of INSR and **SDC1**^1212^ and binding of EIF4E to **EIF4EBP2**^1213^ and to EIF4G1^1214^ (not shown).

The INS receptor (INSR) is a tyrosine kinase receptor that binds INS and regulates glucose homeostasis. The INSR is decreased in the SN of PD patients^190, 191^. The INSR is regulated by **MBNL2** (by regulating alternative splicing)^1215^, **PRKCE** (binds and inhibits)^1216^, FOXO1 (increases expression)^631^, **PALD1** (decreases expression)^1217^, **HLA-C** (binds and translocates INSR to the membrane)^1218, 1219^ and **PTPN1** (inhibits)^1020, 1220, 1221^). So, the expression of INSR, its alternative splicing and its localization to the membrane is part of the PD landscape. In turn, INSR increases the expression of **RPL7A**^1222^ and SCARB1^1223^ (not shown), regulates expression of SIRT1^1223, 1224^ (not shown), **ULK2**^1225^ (not shown), **CXCR4**^1225^ (not shown), **PRKRIR**^1226^ (not shown) and **ACTN4**^1227^ (not shown) and activates **CYP17A1**^1200^. Further, INSR binds **SDC1**^1212^, **PIK3CD**^1228^ and CAV1^802^, and binds and activates JAK2^1229, 1230^ and **PTPN1**^1231, 1232^.

Of note, INS metabolism has profound effects on fat uptake. INS inhibits HDL-mediated cholesterol reverse transport by inhibiting expression of **ABCG1**^1233^. Further, INS (resistance) disturbs cholesterol regulation in the periphery^1234, 1235^, a high-fat diet and INS resistance have been shown to impair nigrostriatal functioning (they attenuated release and clearance of DA in the striatum and increased iron deposition in the SN)^1236^ and PD patients show an increased autoimmune reaction towards serum INS^189^, indicating that INS function may affect PD pathogenesis.

In conclusion, INS and its receptor regulate in the PD landscape, among others, four important transcription regulators (PTEN, FOXO1, **SREBF1** and STAT3), cholesterol metabolism (via SCARB1, ABCA1, **SREBF1**), the rennin-angiotensin system (**AGTR1**), steroid production (**CYP17A1**), immune responses (**CXCR4**, **SCD1**, ICAM1) and mRNA translation (EIF4E, **EIF4EBP2**, EIF4G1).

### B5. Concluding remarks

Cholesterol and sphingolipids are important for membrane function and therefore crucial for normal functioning of the endosome-lysosomal and the lysosome-autophagosomal system that depend heavily on membrane lipid rafts, membrane fission, fusion and trafficking. In return, the endosomal-lysosomal and lysosome-autophagosomal system are important for normal cholesterol and sphingolipid transport and synthesis. The proprotein PSAP regulates the sphingosine synthesis pathway and the trafficking of gangliosides and is therefore an important regulator of (membrane) lipids in the cell. The endosomal-lysosomal system regulates clathrin-(in)dependent endocytosis and recycling and trafficking of vesicles in the cytoplasm. Thereby, the endosomal-lysosomal system regulates the uptake and the processing of lipoproteins, but also the reverse transport of excess lipids out of the cell. Moreover, it also regulates the uptake, degradation or recycling of membrane (receptor) proteins and is in immune cells required for the uptake and processing of proteins for antigen presentation.

During life NM content increases in the DA neurons, hindering the function of the lysosomal compartments and making DA neurons especially vulnerable for defects in the endosomal-lysosomal and lysosome-autophagosomal system. Autophagy-related pathways (e.g. the AMPK-mTORC1 pathway and chaperone-mediated autophagy) regulate protein degradation by the lysosome, reverse cholesterol transport and NM storage. Any defect in the endosomal-lysosomal or lysosome-autophagosomal system – e.g. due protein or NM aggregation, due to mutations or SNPs in genes coding for proteins important for these systems, or due to a dysbalance of membrane lipids – can result in missorting, (further) aggregation of proteins, differential endocytic uptake of lipoproteins and changed cholesterol levels in the cell, affecting cellular function and viability.

Thus, either aggregation of NM or proteins and/or a dysbalance of membrane lipids can create a vicous cycle of lysosomal dysfunction and increased protein aggregation and/or a further dysbalance in cellular lipid levels.

Lastly, systemic regulation of lipoproteins, cholesterol, INS and vitamin D3 are associated with PD and might prove valuable targets for future PD therapies. Moreover, these factors regulate multiple major cascades in the PD landscape and balancing of these factors in the periphery can possibly indirectly improve the viability of DA neurons in PD.

## C. ER STRESS RESPONSE

The ER has a broad range of functions, including protein folding, lipid biosynthesis and Ca^2+^ storage^1237^. The balance of synthesis, folding and degradation of proteins is perturbed as we age, resulting in the production and accumulation of misfolded proteins^1238^. Aging-linked declines in the expression and activity of ER molecular chaperones and folding enzymes compromise proper protein folding and the unfolded protein response (UPR)^1237^. And, as PD incidence is higher among older individuals, with most cases older 50 years of age^1239, 1240^ and PD prevalence rises with age^1241^, it is possible that aging-linked decline of ER function and increase of protein aggregation, resulting in ER stress, may play a role in onset of PD pathology.

Section C1 discusses the pathways and proteins that regulate the UPR and section C2 discusses the pathways and mechanisms that (when dysregulated) can increase protein aggregation and subsequently ER stress. Section C3 shows the interaction between ER stress regulation and cholesterol-regulated gene expression and section C4 discusses the role of the – in the ER synthesized – male hormone testosterone in PD. The functional interactions between the proteins within process C, ‘ER stress response’, are shown in **Supplementary Figure 2**.

### C1. Activation of the UPR

Disturbances in normal ER function, e.g. by unfolded or misfolded proteins in the ER, cause ER stress, which activates the UPR, a stress response that tries to restore normal ER function by degrading misfolded proteins, increasing production of ER chaperones that regulate protein folding and halting protein translation. The other way around, dysregulation of proteins involved in protein degradation, folding or synthesis may result in the accumulation of misfolded proteins that induce ER stress and activate the UPR^1242, 1243^. Misfolded proteins and protein aggregates, e.g. **SNCA** and CTNNB1 aggregates, thus induce ER stress^1244-1246^ and thereby activate site-1 and site-2 protease that in turn activate the transmembrane transcription factor **ATF6**^1247^. **ATF6** activation increases expression of genes controlled by ER stress elements, resulting in the UPR^1248, 1249^, i.e. ER stress increases the expression of **MCFD2**, the cargo receptor for ER-to-golgi transport^1250^ and the selenoprotein **SEP15**, which both may be involved in ER quality control of protein folding^312, 1250^. Further, also **TMX1** is involved in ER quality control^1251^ and binds to **MCC**^387^, that in turn binds to **CUL2**^387^, **DCUN1D1**^387^, **SNRPB2**^387^ and the familial PD proteins VPS35^387^, UCHL1^387^ (associated with the ER membrane^1252^) and EIF4G1^387^. Activation of **ATF6** also increases MTOR activity^967^ that in turn causes ER stress by inhibiting autophagy and increasing protein synthesis^1253, 1254^. Thus, MTOR activation increases the amount of misfolded proteins and creates a separate reinforcing feedback loop of UPR/**ATF6**- and MTOR- activation. Nevertheless, mild ER stress induces autophagy and inhibits neuronal death^939, 1243^, whereas prolonged ER stress decreases **ATF6** activation^1255^, results in increased **SNCA** aggregation^1245^ and causes neuronal death due to opening of the mPTP for cytochrome c release^1256^.

Of note, **LRRK2** is associated with the ER in DA neurons^1257^ and prevents DA neurodegeneration by supporting the upregulation of the **ATF6**-dependent molecular chaperone HSPA5 during ER stress^1258^. HSPA5 (also upregulated by CRH-activation of **CRHR1**, i.e. CRH-induced ER stress^1259^) activates the UPR and diminishes **SNCA** neurotoxicity in a PD rat model^967, 1260^.

#### C1.1 UPR feedback inhibition

The protein phosphatase-1 complex (**PP1**) dephosphorylates proteins and thereby regulates multiple processes in the cell, such as cell division, glycogen metabolism, muscle contractility and protein synthesis^312^. A hallmark of UPR activation is the phosphorylation of the translation initiator EIF2A^1261^ (EIF2 signaling is associated with PD^165^). This attenuates protein synthesis and enables the cell to remove misfolded proteins from the ER^1262^. During ER stress, there is feedback inhibition through **PP1**-dependent dephosphorylation of EIF2A^312, 1263-1265^ (not shown). **PP1** thereby reinitiates protein synthesis and facilitates the recovery of cell from stress^1263^. However, overactivation of the **PP1** complex would shut down the UPR too early and increase ER stress. Selective inhibition of the **PP1** complex can therefore protect cells from ER stress^1265^.

Two inhibitory subunits of the **PP1** (**PPP1R12B** and **PPP1R14C**^312^) were associated with PD in the GWASs^6, 13^. **PPP1R12B** is part of the myosin phosphatase complex, binds the catalytic **PP1** subunit PPP1CB^1266^ and thereby regulates myosin activity^312^. Further, another member of the myosin phosphatase complex PPP1R12A is downregulated in the CSF of PD patients^224^, is activated by **PRKG1**^1267^ and binds PPP1CB^387^ and **LRRK2**^742^. PPP1CA, PPP1CB and PPP1CC are the catalytic subunits of **PP1**, whereas PPP1CA is the physiological **LRRK2** phosphatase and is inhibited by the membrane phospholipid PA^540^ and activated by ceramide^540^. Pathogenic PD mutations in **LRRK2** mutations are associated with a decreased phosphorylation state of **LRRK2**^1268^, implying that phosphatase acitivity of PPP1CA is important in PD. PPP1CA also binds to **SNCA**^461^, EIF2A^1269^ (not shown), AKT1^1023^ (is inhibited by PPP1CA^1023, 1024^), **ATR**^1269^, **AXIN1**^1270^, CAV1^1023^ (not shown) (inhibits PPP1CA^1023^ (not shown)), **CNTN1**^1271^ (not shown), **CSMD1**^1272^, CASP9^1273^, **DLG2**^1272^ (binds NOS1^1274^ (not shown), **DSCAM**^1275^ and **ATP2B2**^1276^), **GPATCH2**^1272^ (not shown), GSK3B^1277^, HSPA8^1278^, **MAP1B**^1272^, **MAPT**^1269^, PTEN^1269^ , SIRT7^394^, TP53^859, 1269^, **WNK1**^1272^ and **WWOX**^1279^. PPP1CA expression is increased by **GNA12**^747^, regulated by vitamin D3^1094^ (not shown) and TP53^1280^ (not shown) and decreased by chronic DA depletion^509^.

In summary, a disturbance in **PP1** activity, e.g. due to dysregulation of PA, ceramide, vitamin D3 or DA, affects ER stress regulation and the regulation of multiple proteins in the landscape, including the familial PD proteins **LRRK2** and **SNCA**.

Another phosphatase, the **PP1**-like phosphatase **PPM1L**, inhibits binding of **MAP2K6** and MAP3K7^1281^ and thereby prevents activation of **MAP2K6** by MAP3K7^1282^. **MAP2K6** increases NF-KB complex expression^1283-1285^ and binds and regulates the expression of **LRRK2**^1286^, but **MAP2K6** is also phosphorylated by **LRRK2**^931^. Further, MAP3K7 activates the **AMPK** complex^1287^, is inhibited by **TRAF3**^1288^ and binds to **SMAD5**^1289^. **SMAD5** is activated by **BMP7**^1290^ and binds to SMAD4^859^ (binds to **ZNF423**^1291^), **RUNX3**^1292^ and the **26S** proteasome^859^ (binds **FBXO25**, that again binds to HSPA8^395^).

### C2. Protein aggregation

Timely detection of misfolded proteins by E3 ubiquitin-protein ligase complexes and subsequent degradation by the proteasome prevents protein aggregation and ER stress. Section C2.1 discusses shortly proteins that affect protein translation, section C2.2. discusses the targeting of proteins to the proteasome by the small protein ubiquitin and section C2.3 discusses the proteins in the PD landscape that directly regulate proteasome function. Section C2.4 shows the involvement of presenilins and protein cleavage and section C2.5 discusses the effects of beta-catenin (CTNNB1) aggregation on (DA) neuron function and how its levels are controlled in the cell.

#### C2.1 Protein translation

Incorrect protein translation can result in abnormal protein function, mislocalized proteins and protein aggregation. The ribosomal DNA transcription factor SIRT7 binds to proteins that are involved in the regulation of protein synthesis, e.g. the histone protein **H1FX**^394^, the RISC complex component **SND1**^394^, pre-mRNA splicing factor **BAT2**^394^ (binds the RNA-binding and alternative splicing regulator **RBFOX1**^1293^), the 60S ribosomal proteins **RPL7A**^394^ and **RPL38**^394^ and the familial PD protein EIF4G1^394^ (involved in mRNA cap recognition and recruitment to the ribosome^1294^). Further, SIRT7 also binds **USP9X**^394^ and **PLOD1** (involved in the formation and stabilization of collagens^1295^, such as **COL2A1** and **COL18A1**^394^) and regulates autophagic (via binding to MTOR, STK11) and cytoskeletal (via binding to **KIF14**, **MAP1B**, DNM2, **ACTN4**) processes^394^. Thus, the PD landscape contains multiple proteins that regulate protein transcription and translation.

#### C2.2 Ubiquitin

Ubiquitin is a small regulatory protein that, by binding to substate proteins, is able to regulate their cellular localization, protein-protein interaction or degradation^1296, 1297^. E3 ubiquitin-protein ligase complexes catalyze the ubiquitination of proteins and targets them for proteasomal degradation. **TRAF3** is an essential constituent of several E3 ubiquitin-protein ligase complexes^312^, regulates the NF-KB complex (see above) and binds to **AGTR1**^362^. Also, TRAF2 is part of the E3 ubiquitinase complex, binds **TRAF3**^1298^ and like **TRAF3** decreases the expression of the actin polymerization protein^1299^ **LRRC16A**^1300^. Further, TRAF2 ubiquitin ligase activity is strongly activated by S1P^1301^, TRAF2 mediates **SREBF1** activity^1302^ (not shown) and binds to **AMPK**^1303^ and the familial PD proteins HTRA2^954^ and FBXO7^1304^. Upon cellular stress, HTRA2 is released from mitochondria and induces apoptosis^405^ (part of process A) – e.g. by binding to the cell cycle and apoptosis regulator **CCAR2**^1305^ – and inhibits the E3 ubiquitin ligase activity of **PARK2**^405^. FBXO7 on the other hand, is together with the F-box proteins **FBXO25** (binds to a complex consisting of PPP1R12A and **PPP1R12B**^395^) and **FBXO36** part of an ubiquitin-protein ligase complex^312, 1306, 1307^.

Cullin-2 (**CUL2**) is a core component of E3 ubiquitin-protein ligase complexes^312^ and binds to **DCUN1D1**^1308^ and NEDD8^1309^. **DCUN1D1** activates the E3 ubiquitin-ligase complex by recruiting a NEDD8-charged E2 enzyme to the cullin component^312^. Further, NEDD8 also binds to SIRT1^656^, **RPL7A**^1135^, HSPA8^1134^, UCHL1^846^ and binds **PARK2** and increases its ubiquitinase activity^200, 1310^ (activates the **26S** proteasome^1310^) and binds and stabilizes PINK1^200^. Moreover, NEDD8 accumulation is observed in LB in DA neurons in the SN of PD patients^199, 200^.

The deubiquitinases UCHL1 and **USP9X** affect, by their ability to deubiquitinate, proteasomal degradation of misfolded proteins^43, 1252^. Of note, **USP9X** deubiquitinates **SNCA** and is lower expressed in the SN of PD patients, which may contribute to higher levels of monoubiquitinated **SNCA**^43^. **USP9X** thereby determines if **SNCA** is degraded by the proteasome (monoubiquitinated **SNCA**) or by autophagy (deubiquitinated **SNCA**) ^43, 1311^).

Thus, proteasomal targeting of proteins by ubiquitin is highly regulated in the PD landscape, either by ubiquitin-protein ligase complexes (composed of among others **CUL2**, **DCUN1D1**, FBXO7, **FBXO25**, **FBXO36**, **PARK2** and **TRAF3**) or deubiquitinases (e.g. UCHL1, **USP9X**).

#### C2.3 Proteasomal degradation

Proteasomal function is impaired in the PD SN and results in aggregation of (misfolded) proteins^45^. Degradation of misfolded proteins requires retrograde transport of these proteins across the ER membrane and subsequent degradation by the ubiquitin-proteasome system, which includes the **26S** proteasome^1312^. **PSMD11** is a regulatory subunit of the **26S** proteasome^1313^. The **26S** proteasome is inhibited by **AMPK**^1314^, whereas aggregated **SNCA** binds and decreases its activity^532, 1315, 1316^. The protein **BAG6**  is part of a ubiquitination-complex that prevents aggregation of mislocalized proteins, by targeting them for proteasomal degradation^1317^. Further, **BAG6** has a key role in assembly of the **26S**-proteasome complex^1318^ and cleavage of **BAG6** by CASP3 induces apoptosis^1319^. Further, the cGMP-dependent protein kinase **PRKG1** (also regulating actin cytoskeleton and myosin-mediated trafficking by binding to **PPP1R12B**^1320^) increases activity of the proteasome and increases proteasome-mediated degradation of misfolded proteins^1321^. Thus, proteasome function is regulated in the PD landscape (via **BAG6**, **PRKG1**, **PSMD11**) and defects in this system may result in protein aggregation and neuron death.

#### C2.4 Presenilins and protein cleavage

Activation of the UPR by ER stress increases the activation of γ-secretase to increase proteolytic cleavage of unfolded proteins in the ER^1322^. The γ-secretase is a protease complex (its catalytic core consisting of the presenilin PSEN1 or PSEN2) that cleaves single-pass transmembrane proteins within their transmembrane domain. γ-Secretase is well known for cleaving of APP^1323^ (not shown), resulting in amyloid-beta aggregation in Alzheimer’s disease^1324^. In addition to APP, γ-secretase also cleaves and thereby decreases CTNNB1 levels in the cell^1325-1327^, cleaves LDLR^1328^ (not shown), **SDC1**^1328^ (not shown), LRP1 (is in competition with APP for γ-secretase activity)^1329, 1330^ and increases the expression of **IL2RA** on the plasma membrane^1331^ (not shown). AKT1 is necessary for γ-secretase activation^1332^, cholesterol increases γ-secretase activation^1333^ and **RER1** increases the retention and retrieval of γ-secretase (subunits) in the ER, preventing its activity^887-889^. **PARK2** regulates the promoter activity of PSEN1 and PSEN2 and thereby increases PSEN1-associated γ-secretase activity and reduces PSEN2-associated CASP3 activation^1334^. The presenilins PSEN1 (activated by ER stress^1335^) and PSEN2 also have γ-secretase-independent functions^1336^, i.e. they decrease INSR expression and thereby inhibit INS-signaling^1337^ and are necessary for protein degradation by the lysosome-autophagosomal system (part of process B) by regulating (among others) mTOR and LAMP2^1338^. Of note, PSEN1 knockout mice show increased lysosomal **SNCA** aggregation^1339^. PSEN1 binds CTNNB1^1340^, **MAPT**^1341^, APOE^1342^, GSK3B^1343^, HSPA8^1344^ (not shown), **ENSA**^1342^ (not shown), EIF4G1^1345^, HTRA2^1346^ (not shown) and **ATP6V0A1**^1347^ (not shown) and activates HTRA2^1348^. Furthermore, PSEN2 binds CTNNB1^1349^, **ATP6V0A1**^1344^ (not shown) and CASP3^1350^, increases expression of TP53^1351^ (not shown) and CASP3 (not shown; and increases activation of CASP3)^1351, 1352^, decreases expression of PSEN1^1351^ and inhibits activation of PLA2G6^1353^ (not shown).

Thus, the presenilins PSEN1 and PSEN2 are important for γ-secretase-dependent *and* -independent cleavage and degradation of proteins. Dysregulation of these presenilins or γ-secretase (e.g. due to **RER1**- and **PARK2**-dependent regulation or via activation by AKT1 and cholesterol) may result in activation of apoptotic processes (via HTRA2, TP53, CASP3), dysregulation of the immune response (via **SDC1**, **IL2RA**) and of cellular cholesterol homeostasis (via LDLR and LRP1) and CTNNB1 and **SNCA** aggregation.

#### C2.5 Beta-catenin aggregation and -dependent transcription

Protein aggregation is not always bad and is sometimes even crucial for cell development, i.e. stabilization and subsequent aggregation of cytoplasmic beta-catenin (CTNNB1) in ventral midbrain precursor cells increases their differentiation into DA neurons^1354^. Aggregation of CTNNB1 in the cytoplasm causes it to translocate into the nucleus to function as a coactivator of transcription factors^1355^, e.g. CTNNB1 increases expression of UCHL1^1356^ and LMX1B^1357^. This implies that cytoplasmic CTNNB1 aggregation may regulate DA neuron development and maintenance (see also section D1.1) and could play a role in PD pathology. Nevertheless, cytoplasmic CTNNB1 aggregation also results in ER stress and activation of the UPR^1246^.

CTNNB1 expression and aggregation is decreased by CAV1^1358^ (not shown), **COL18A1**^1359-1361^, **PRKG1**^1362, 1363^, PTEN^1364^, **WWOX**^1365^ and γ-secretase (see section C2.4). Moreover, in a healthy cell, cytoplasmic CTNNB1 is degraded by the beta-catenin destruction complex, that consists of, among others, **AXIN1** and GSK3B^1366^. **AXIN1** binds CTNNB1^1366^ and degrades excessive cytoplasmic CTNNB1^1367^, whereas inhibition of GSK3B stabilizes cytoplasmic CTNNB1^1354^. Further, **AXIN1** also binds to **GAK**^846^ and GSK3B^1368^, and **AXIN1** inhibits GSK3B-dependent phosphorylation of **MAPT** (and thus may prevent against **MAPT** hyperphosphorylation and tau aggregation^1369^). **MAPT** also binds to **SNCA**^1370^, **LRRK2**^1371^ and **PARK2**^1372^. The wnt pathway inhibits degradation of CTNNB1 by the beta-catenin destruction complex^1373^ causing CTNNB1 accumulation – e.g. because **WNT3** is involved in a signaling cascade that stabilizes and increases the expression of CTNNB1^1365, 1374^ – and is involved in DA neuroprotection, development and repair^1375, 1376^. Further, under apoptotic-conditions (e.g. Ca^2+^ influx), the γ-secretase complex promotes disassembly of the E-cadherin/catenin-complex and thereby increases the pool of cytoplasmic CTNNB1^312^.

Other proteins that increase CTNNB1 aggregation or decrease its degradation are the deubiquitinase **USP9X** (binds CTNNB1, close to the **AXIN1**-binding site, and inhibits CTNNB1 degradation)^1377^, the familial PD protein UCHL1 (binds and stabilizes CTNNB1)^1356^ and **GNA12** (by decreasing the inhibitory function of cadherins on active CTNNB1^740^. **GNA12** also binds and is regulated by **AXIN1**^744^). Further, CAV1 binds CTNNB1^809^ and nuclear CTNNB1 is increased in a mouse CAV1-knockout^1358^, indicating a role for the endocytic/autophagic pathway in maintaining CTNNB1 levels.

Other proteins that bind to CTNNB1 are **CACNA1A**^505^, **ACTN4**^1378^, **ARMC8**^1379^ (not shown), SIRT1^645^, **CDH6**^1380^, **STK39**^1367^, **FER**^1381^, **PARD3**^1382^, UCHL1^1356^ and **SNCA**^461^. The latter indicating that CTNNB1 and **SNCA** aggregation may be able to affect each other.

Nuclear translocation of CTNNB1 increases the expression of **ACTN4**^1383^, **BAT2**^1383^, **GJB2**^1383^, **GNA12**^1383^, **BMP7**^1357^ (not shown), **TMEM2**^1384^ (not shown), **MSX1**^1385^, **BAMBI**^1386^ (not shown), **ARL4A**^1387^ (not shown) and UCHL1^1356^. Nuclear CTNNB1 binds to the transcription regulators **RUNX3**^1388^, FOXO1^1389^, MITF^1062^, SIRT1^645^, **TLE1**^1390^ and also to **SNCA**^461^ and may affect their function. Controlling CTNNB1 levels in the cell may therefore be important in DA neuron homeostasis (**MSX1**), regulation of familial PD proteins (UCHL1, **SNCA**) and multiple (PD associated) transcription regulators (**RUNX3**, FOXO1, MITF, SIRT1 and **TLE1**).

### C3. ER stress- and cholesterol-regulated gene expression

During ER stress **ATF6** is cleaved and translocated to the nucleus where it activates the transcription of genes involved in the UPR^1248, 1249^, e.g. HSPA5^1248^ (diminishes **SNCA** neurotoxicity in a rat PD model^1260^). Further, **ATF6** activates NF-KB^1391^ and is involved in astroglia activation and neuronal survival (in a PD mice model)^1392^.

ER stress not only activates the UPR, but also increases cholesterol uptake by increasing **SREBF1** activity^1393, 1394^, as the same site-1 and site-2 proteases that activate the transmembrane transcription factor **ATF6** also splice and activate **SREBF1**^1247, 1395^. Mature, cleaved **SREBF1**, enters the nucleus and increases the transcription of proteins involved in cholesterol metabolism. Of note, a SNP (rs11868035; which was also associated with PD via the GWASs^12^) located in the splice site of **SREBF1** is associated with gait impairment in PD^32^. **SREBF1** increases transcription of proteins involved in lipid and cholesterol regulation, e.g. SCARB1^932^, LDLR^1393, 1396, 1397^, ABCA1^925^ and STAR^603, 604^ (not shown) and **SREBF1** activation results in activation of the mevalonate pathway and synthesis of cholesterol^1398^. Cholesterol is de-novo synthesized in the ER (in small amounts) and transported to the cytoplasm by **ABCG1**^1154, 1399^. Other proteins that regulate the mevalonate pathway are the mitochondrial **MCCC1** and **ACSL6**. **MCCC1** is a 3-methylcrotonyl-CoA carboxylase (activated via biotinylation by **HLCS**^312^) involved in the leucine metabolism eventually resulting in the production of HMG-CoA, an intermediate of the mevalonate pathway^1400^. **ACSL6** is an acyl-Coa synthase that is located in membranes and activates long-chain fatty acids so they can subsequently be degraded by β-oxidation in the mitochondria and produce acetyl-CoA^312^. Acetyl-CoA can be oxidized in the citric-acid cycle or can enter the mevalonate pathway for the production of cholesterol^312^.

In addition to ER stress, **SREBF1** expression is also regulated by cholesterol^1401, 1402^, increased by LDLR^1403^ and the tyrosine-protein phosphatase and regulator of the UPR **PTPN1**^312, 1404^. Moreover, **SREBF1** is decreased by vitamin D3^1104^, FOXO1^632^, PTEN^1405, 1406^, the serine/theronine-protein kinase **SIK1**^1004^, TP53^1407^, STAT3^1408^, IL1B^1409^ (not shown) and ICAM1^1410^ and binds **ATR**^1411^, **MED13**^1412^ (binds also to **CDK19**^1413^) and SIRT1^647^. The transcriptional regulator **CREM** dysregulates cholesterol homeostasis by increasing ABCA1 expression^1414^, decreasing LDLR expression^1414^ and increasing the expression of the **SREBF1**-inhibiting kinase **SIK1**^1414^. **CREM** also decreases expression of **HLA-DRA**^1415^ and TH^298^ and thereby not only affects cholesterol homeostasis in the cell, but also immune function and dopamine production. Next to cholesterol-linked proteins, **SREBF1** also increases the transcription of SERPINE1^1416^, **ULK2**^1011^, TP53^1417^, NOS2^751^ and AR^1418^ and decreases the expression of ADH1C^1419^, a cytoplasmic enzyme involved in the production of retinoic acid^1420^. A mutation in ADH1C is associated with PD susceptibility^112^ and its expression is, in addition to **SREBF1**, also decreased by RXRA^1421^ (not shown) and INS^1419^ (not shown) and increased by angiotensin II^1422^ (not shown).

Of note, whereas ER stress increases **SREBF1** activity, dysregulation of cholesterol, oxLDL, sphingomyelin and **SREBF1**, and cellular lipid accumulation have been associated with ER stress and UPR activation by **ATF6**^523, 778, 1166, 1423-1426^.

In conclusion, both ER stress and dysregulation of lipid homeostasis can activate **ATF6** and **SREBF1**, and thereby show a complex interaction between ER stress, lipid homeostasis and activation of the UPR.

### C4. Testosterone metabolism

Overall, in Caucasian populations the incidence rate of PD is 1.5-2.0 times higher among men than women^1239, 1427, 1428^, making the male gender a risk factor for developing sporadic PD^23, 1239, 1429^ and suggesting that sex hormone levels may play a role in PD etiology. Of note, in Asian PD patients such a male predominance was not found^1239, 1428^. Healthy Asian males have comparable estradiol levels with healthy non-Asian males, but show lower testosterone levels^1430, 1431^. The higher testosterone levels in Caucasian males may make them more susceptible for the effects of a testosterone drop than females. Due to their lower testosterone levels, there is no such drop in Asian males, which may explain the absence of the male gender as a risk factor in the Asian population. Indeed, male PD patients show significantly reduced testosterone levels compared to healthy controls^1432-1434^, and their possible contribution to PD pathogenesis may be illustrated by the inverse correlation of testosterone level and apathy in PD patients^1432^. Further, other indicators of testosterone involvement in PD-related mechanisms are; the lower testosterone levels by inhibition of mitochondrial complex I in the rotenone-treated rat, a model for PD^1435^, and the increase in **SNCA** levels accompanying a decrease in TH-positive neurons and fibers in the SN and striatum respectively after castration of young male mice^1436^.

#### C4.1 Regulation of testosterone and cholesterol

**CYP17A1** dysregulation could attribute to the lower testosterone levels seen in male PD patients, namely, **CYP17A1** is one of the enzymes involved in the conversion of pregnenolone and progesterone (both synthesized from cholesterol) to testosterone^623, 1437^. **CYP17A1** is located on the mitochondrial and ER membrane^312^ and its expression is increased by INS^1200, 1201^, AR^1438^, angiotensin II^1439^, MTOR and the mTORC1^1440^, S1P (via activation of **SREBF1**)^737^ and decreased by RELA^609^, AR^1441^, **BMP7**^1442^ (not shown) and the acid ceramidase **ASAH1** (hydrolyzes ceramide into sphingosine)^1443^. The role of **ASAH1** can be explained by the inhibiting effect of sphingosine on **CYP17A1** expression by binding to NR5A1, a nuclear receptor that activates (synergistically with **DGKQ**^312^) **CYP17A1** transcription^1444^. NR5A1 also binds CTNNB1^1445^, NF-KB^1446^ (not shown), AR^1447^ (not shown), **SREBF1**^1448^ and **DGKQ** (^539^ and increases the expression of SCARB1^1449^ (not shown), **DGKQ**^1448^ and the mitochondrial cholesterol transporter protein STAR^597, 598^ (not shown). Dysregulation of the sphingosine-S1P balance in the cell can therefore have major consequences on (mitochondrial) cholesterol levels, **CYP17A1** expression and subsequently testosterone levels. See for details on the role of sphingosine and S1P in the PD landscape section B1.2.

Dysregulation of more upstream processes of the testosterone synthesis cascade, i.e. the cholesterol metabolism, could also affects testosterone levels. For example, the transporters STAR and TSPO regulate import of cholesterol into the mitochondria for conversion into pregnenolone and subsequently testosterone^619, 620, 1450^. Indeed, inhibition of TSPO results in a decreased testosterone production^1450^ and testosterone itself functions as a feedback inhibition by decreasing the expression of STAR^620^. Furthermore, the cholesterol transporter ABCA1 and also INS increase the quantity of testosterone^1201, 1451, 1452^.

In healthy adult men, free testosterone levels are positively correlated with LDL-, HDL- and total cholesterol levels^1453^. A decreased testosterone level could influence several processes in the PD landscape (not shown), as testosterone regulates efflux of LDL- and HDL-cholesterol to the blood^1454-1456^, increases the expression of INS^1457^, MHC class II proteins^1458^, CAV1^1459^, CASP3^1460^, SYT11^1461^, PSEN1^1461^, SIRT1^1462^, SOD2^1463^ and decreases expression of PLAU^1464^, TGFBR2^1465^ and **CRHR1**^1466^.

In summary, dysregulation of the cholesterol metabolism (e.g. due to ER stress) may result in lower testosterone levels as seen in PD patients. And, in return, changes in testosterone levels can affect cholesterol homeostasis and multiple pathways in the PD landscape, and may play a role in PD pathology.

#### C4.2 Androgen receptor (AR)

In the nucleus, testosterone binds and activates the AR, increasing AR-dependent transcription^1467^. This results in a positive feedback loop, as **CYP17A1** expression is increased by the AR^1438^. Regulation of the AR by other pathways and proteins in the PD landscape can thus indirectly also affect testosterone levels. In the PD landscape, AR expression is increased by **SREBF1**^1418^ and NF-KB^1468^ and decreased by SIRT1^653^, TP53^1469^ and regulated by MTOR^1470, 1471^, **SLC45A3**^1472^ and CTNNB1^1473, 1474^. Moreover, multiple PD landscape proteins bind to the AR, i.e. **SREBF1**^1475^, **EFCAB6**^1476^, CAV1^1477^ (not shown), FOXO1^1478^, PARK7^1479^, AKT1^1480^, GSK3B^1481^, STAT3^1482^, **POU2F1**^1483^ (not shown), NF-KB^1484^, SIRT1^653^, HSPA5^1138^ (not shown), PTEN^1485^, CASP3^1486^ (not shown), TP53^1487^, **GAK**^1488^ and **STK39**^1489^. Most of these physical interactions are often accompanied by an inhibition or activation of one or both of the binding partners, i.e. AR is activated by **BMP7**^1490^ and PARK7^1479, 1491^ (by binding and inhibition of **EFCAB6**^1476^), activates AKT1^1492^ (not shown) and activates and is activated by STAT3^1482^. Further, AR is inhibited by **SREBF1**^1475^, FOXO1^1493^, AKT1^1480^, GSK3B^1481^ and PTEN^1485^, inhibits and is inhibited by NF-KB^1484^ and is cleaved by CASP3^1494^ (not shown).

AR activation increases the expression of **TACC2**^1495^, CTNNB1^1496^ (increases nuclear CTNNB1^1497^ and CTNNB1-mediated transcription^1498^), MAOA^277^, PLAT^1499^ (not shown), **CACNA1A**^1499^, HSPA5^1500^ (not shown), IGF1^1501^ (not shown), PTEN^1502^ and STAT3^1482^. Further, AR decreases the expression of **ITGA6**^1503^, SERPINE1^1504^ (not shown) and **SERPINB5**^1505^ (not shown), is required for BAX translocation to mitochondria^1506^ (not shown) and decreases nuclear TP53 accumulation^1507^.

In summary, the AR is regulated by several major pathways in the PD landscape, is regulated by testosterone and by the familial protein PARK7. Moreover, AR itself regulates CTNNB1-mediated transcription, ER stress responses (via HSPA5), coagulation (via PLAT, SERPINE1), testosterone levels (via **CYP17A1**), intracellular Ca^2+^ levels (via **CACNA1A**), INS (via IGF1), immune (via STAT3, **ITGA6**) and apoptotic (via BAX, TP53) pathways. A slight change in testosterone level, as seen in PD patients can therefore have a huge effect on the regulation of these pathways.

### C5. Concluding remarks

Dysfunction of E3 ubiquitin ligase complexes, the proteasome or processes involved in protein translation, modification, cleavage and degradation (by presenilins, the proteasome and/or lysosome) result in protein misfolding and/or aggregation that leads to ER stress, activation of **ATF6** and the subsequent induction of the UPR. ER stress activates presenilins, that (either γ-secretase-dependent or –independent) decrease protein aggregation, but also regulate activation of apoptotic and immune pathways. The site-1 and -2 proteases necessary for **ATF6** activation during ER stress, also activate **SREBF1** and thereby affect the cellular cholesterol homeostasis. In turn, cholesterol, and (oxidated) lipids not only affect **SREBF1** activation, but also induce ER stress and activation of the UPR via **ATF6**. This shows that ER stress and cholesterol metabolism are highly interconnected, indicating that their dysregulation can lead to ER stress-induced mitochondrial dysfunction (as discussed in section A2.2.1) and neuron death.

Moreover, testosterone metabolism is affected by both cholesterol regulation and ER (dys)function, i.e. it is synthesized from cholesterol and produced in the ER. Lower testosterone levels as seen in male PD patients can result from a disbalance in either cholesterol metabolism or ER function and may play a role in PD pathology.

## D. NEURON DEATH AND IMMUNE RESPONSE

DA neuron death can occur due to dysregulation of intracellular pathways and processes, immune responses targeting DA neurons, or a combination of the two. This section discusses pathways that regulate DA neuron determination, neuron survival and death (section D1) and pathways that regulate the immune response and immune cell activation (section D2). The functional interactions between the proteins within process D, ‘neuron death and immune response’, are shown in **Supplementary Figures 2** and **3**.

### D1. DA neuron determination, survival and death

The following subsections discuss the regulation of a small set of transcription factors necessary for differentiation into and maintenance of DA neurons (D1.1), the neurotrophic factors brain-derived neurotrophic factor (BDNF) and glial cell line-derived factor (GDNF) that support the survival of neurons (D1.2) and the pro-apoptotic factors (GSK3B, PTEN, TP53 and CASP3) that regulate cellular death (D1.3).

#### D1.1 Regulation of the “DA signature”

The transcription factors ASCL1, **MSX1**, LMX1A, LMX1B, NEUROG2, NR4A2, PITX3 and SOX2 are implicated in DA neuron development and are sufficient for reprogramming of fibroblasts into DA neurons^1054, 1508-1510^. These transcription factors are therefore important in maintaining an expression pattern typically for DA neurons. ASCL1 increases the expression of TH^1511^ and **CACNA2D3**^1512^ and is itself regulated by active AKT1^1513^. SNPs in LMX1A and LMX1B may increase PD risk^1514^. LMX1A increases the expression of NR4A2^1509^, SLC6A3^1515^, NEUROG2^1516^ (not shown), SLIT2^1517^ and INS^1518^. LMX1B is required for normal functioning of the lysosomal-autophagosomal system and DA neuron survival and is decreased in PD DA neurons^193^. LMX1B expression is increased by CTNNB1^1357^ and decreased by PTEN^297^. **MSX1** increases expression of **COL2A1**^1067^. NEUROG2 expression is increased by PINK1^1519^ (not shown) and PITX3^1516^ (not shown), decreased by SIRT1^1520^ (not shown) and is inhibited by GSK3B^1521^ (not shown). NR4A2 increases the expression of DA transporters SLC6A3^1508, 1522^ and SLC18A2^1522^, TH^1522^, LMX1B^1522^, PITX3^1522^, **EIF4EBP2**^1523^ and PRKAA2^1523^, regulates oxLDL^1524^ (not shown) and binds to RELA^1525^ and **RCOR1**^1525^. NR4A2 expression is increased by HMOX1^332^ (not shown), MC1R^1526^ (not shown), STAT3^1527^, IL1B^1528^ (not shown) and TNF^1528^ (not shown) and decreased by oxLDL^1529^ (not shown), PTEN^297^ and **ASAH1**^611^ (not shown). Mutant **SNCA** increases the degradation of NR4A2, which is dependent on th **26s** proteasome complex^1530^ (not shown). SOX2 increases expression of **JARID2**^1531^, **TBX3**^1532^, ASCL1^1532, 1533^, GSK3B^1534^, binds^1534-1537^ and increases expression of CTNNB1^1538^, decreases expression of NFKB2^1532^, **WNT3**^1532, 1533^ and **AXIN1**^1533^ and phosphorylates TP53^1539^ (not shown). CAV1^1540^ (not shown), FOXO1^1541^ and CTNNB1^1542-1544^ increase the expression of SOX2. AKT1 binds, phosphorylates and stabilizes SOX2, which increases SOX2 expression^1545, 1546^ (not shown). Further, SOX2 binds **CCAR2**^1536^, **EHMT2**^1536^, **ETV6**^1536^, **TBX3**^1536^, **TIAL1**^1536^, **USP9X**^1536^, the nuclear transporter **KPNA4**^1536^ (not shown) and NR5A1^1547^. CTNNB1 also increases expression of **MSX1**^1385^.

In summary, normal transcriptional activity of ASCL1, **MSX1**, NEUROG2, NR4A2, PITX3 and SOX2 is necessary to maintain a dopaminergic phenotype. Multiple proteins in the landscape regulate and are regulated by these transcription factors, whose dysregulation would affect expression of proteins that are crucial for DA signaling and regulation e.g., TH, SLC6A3 and SLC18A2. Interestingly, oxLDL decreases NR4A2 expression, and AKT1 – which is highly regulated in the PD landscape – regulates both ASCL1 and SOX2.

#### D1.2 Neurotrophic factors

BDNF is an extracellular protein that supports the survival, growth and differentiation of neurons^1548, 1549^. Further, BDNF is associated with cognitive and motor impairment in PD^128, 131^, is decreased in PD SN neurons^130^ and dysregulated in PD serum^131, 132^ and CSF^133^. BDNF expression is increased by SIRT1^1550, 1551^ and NF-KB^1552^ and decreased by INS^1553^, IFNG^1554^ (not shown), PLAT^1555^ and **SNCA**^1556, 1557^. BDNF activates PLAT^1558^, NF-KB^1559^ (increases NF-KB translocation to the nucleus^1560^) (not shown), STAT3^1561^ (not shown), RAC1 (via **PARD3**)^1562^ (not shown), MTOR^1563, 1564^ (not shown), **SIK1** (and increases its translocation from the cytoplasm to the nucleus)^1565^ (not shown) and AKT1^1566^ (not shown) and inhibits CASP3^1567^ (not shown). Further, BDNF regulates activity of DRD2^1568^ (not shown), increases cholesterol synthesis^1569^ (not shown), decreases INS levels in the blood^1570^ (not shown) and binds clathrin in the cytoplasm^1571^ (not shown). BDNF increases the release of DA^1571, 1572^ (not shown) but can also decrease the expression of TH^1573^ (not shown). Furthermore, BDNF decreases expression of **CXCR4**^1574^ and increases expression of DA receptors^1571, 1575, 1576^ (not shown), AR^1577^ (not shown), PTEN^1578^ (not shown), SOD2^1579^ (not shown) and **MBP**^1580^. **MBP** is the major component of myelin sheats, allowing the axon to rapidly propagate action potentials^312^. Further, **MBP** binds to PA^547^, **HLA-DRA**^1581^ and binds and is phosphorylated by **STK39**^1582^ and **LRRK2**^1583, 1584^. Antibodies against **MBP** are increased in the serum of PD patients^110^, indicating a dysregulation of **MBP**, affecting signal transduction between cells. Moreover, **GLDN** is probably involved in the formation the nodes of Ranvier^312^ and **CNTNAP2** is localized at juxtaparanodes of myelinated axons and mediates interactions between neurons and glia^1585, 1586^, indicating that signaling between DA neurons and glia cells may be disturbed in PD.

Thus, BDNF is regulated by and regulates the immune response (**CXCR4**, NF-KB, IFNG, STAT3), regulates the activity and expression of DA receptors and DA (TH), affects signaltransduction (**MBP**) and regulates lipogenesis (MTOR, **SIK1**), oxidative stress (SOD2) and apoptosis (CASP3).

The neurotrophic factor GDNF promotes survival and differentiation of DA neurons and increases their DA uptake^1587^ and is decreased in neurons of the SN of PD patients^130^. GDNF expression is increased by **ITGA8**^1588^, NF-KB^1552^, regulated by CTNNB1^1589^ (not shown) and decreased by **SNCA**^1556^. GDNF activates STAT3^1590^ (not shown), RAC1^1591, 1592^ (not shown), AKT1^1591, 1593, 1594^ (not shown) and TH^1595^ (not shown). Further, GDNF increases secretion of DA^1596^ (not shown) and increases the expression of TH^1597, 1598^ (not shown), SLC6A3^1598-1600^, DRD2^1572^, SPHK1^418^ (increasing synthesis of S1P^418^), **ITGA6**^1601^ and decreases the expression of CASP3^1602^ (by decreasing cleavage of pro-CASP3^1603^) (not shown) and cytochrome c^1602^ (not shown). However, chronic upregulation of GDNF decreases TH expression^1604^ (not shown).

Thus, GDNF regulates DA homeostasis (TH, SLC6A3, DRD2) is regulated by and regulates the immune response (NF-KB, **ITGA6**, **ITGA8**, S1P) and apoptotic processes (CASP3, cytochrome c).

#### D1.3 Pro-apoptotic proteins

Cellular death is regulated by a variety of proteins, the main proteins that regulate apoptotic pathways in the PD landscape, GSK3B (D1.3.1), PTEN (D1.3.2), TP53 (D1.3.3) and CASP3 (D1.3.4) are discussed in this section.

##### D1.3.1 GSK3B

PD-associated polymorphisms alter the transcription and splicing of GSK3B^158^ and affect it functions in the cell. For example, GSK3B is part of the CTNNB1 destruction complex (see also section C2.5), regulates cellular energy levels, inhibits mitochondrial complex I, which affects mitochondrial function and increases ROS production^1605, 1606^ and increases accumulation of **SNCA**^1607^. In turn, **SNCA** can activate GSK3B^1606, 1607^, which may lead to a vicous cycle of GSK3B activation and **SNCA** aggregation. Further, GSK3B is also activated by **CRHR1**^1608^, IFNG^1609^, mTORC1^971^, PTEN^1610^ and inhibited by **PARD3**^1611^, **PTPN1**^1612^, **PIK3CD**^628, 1613^, AKT1^1614^, PTEN^1615^, INSR^1616^, **NTF3**^1617^, **ASAH1**^1618^ (not shown), **ATR**^1619^ (not shown), **PRKCE**^1620^, TP53^1280^ and HDL-S1P^1621^. Further, GSK3B binds to **SREBF1**^1622^, **MAPT**^1623^, **BAG6**^884^, HSPA8^1131^, **HLA-DQA1**^1624^ (not shown), DDIT4^1625^, AKT1^1626^, PPP1CA^1277^, **PTPN1**^1612^, **AXIN1**^1368^, NF-KB^1627^, mTORC1^884^, STAT3^1628^ and the PD proteins **LRRK2**^1629^ and **SNCA**^1630^.

GSK3B increases the phosphorylation of **MAP1B**^1631^ (binding to both **SNCA**^1632^ and PINK1^1633^) and **MAPT**^312^ (dephosphorylated by **AXIN1**^1369^), affecting microtubule binding and stabilization. GSK3B activation also increases accumulation of cholesterol^1634^, increases the accumulation and activation of TP53^1635, 1636^ and the activation of CASP3^1637, 1638^ and STAT3^1609^. Further, GSK3B inhibits **SREBF1**^1639^, inhibits lysosomal acidification and regulates autophagy by regulating mTORC1 activity^963, 1640^ and regulates NF-KB complex activation (in the nucleus)^1641-1645^ and TP53 expression^1646-1648^. Therefore, GSK3B can play a crucial role in **SNCA** aggregation, autophagy, cholesterol regulation and activation of the apoptotic factors TP53 and CASP3.

##### D1.3.2 PTEN

The phosphatase and tumor suppressor PTEN is located in the cytoplasm when it is nonubiquitinated and located in the nucleus when monoubiquitinated^312^. PTEN knock down in DA neurons is neuroprotective in PD models^297^. PTEN interacts with the familial PD proteins PARK7 and PINK1. (Oxidized) PARK7 binds PTEN^1649^, decreases its expression^428^ and inhibits its activity^1649^, whereas PINK1 is bound and regulated by PTEN^1650, 1651^. Further, PINK1 also binds to **KIF11**^1633^, the familial PD proteins HTRA2^1652^, **PARK2**^1653^, PARK7^1654^ and **SNCA**^1655^ and binds and is activated by MARK2^1000^ (MARK2 increases the expression of the vasopressin transporter^1656^ **SLCO3A1**^1657^ (not shown) and vasopressin levels are altered in PD^1658, 1659^). Thus, PTEN can affect multiple familial PD proteins via PINK1 regulation. PTEN expression is increased by **JARID2**^1660^, whereas **COL18A1** decreases tyrosine phosphorylation of PTEN^752^. In addition to PINK1 regulation, PTEN also regulates STAT3 expression^1661^, increases the expression of **FAM134C**^1405^, **HAVCR1**^1405^, **MBP**^1662^ and **RAP1A**^1663^ and decreases the expression of **PLOD1**^1405^, NF-KB^1664^, **SREBF1**^1405, 1406^, MTOR^965^, CTNNB1^1364^, **ANGPT2**^1665^, PITX3^297^, PLAU^1666^, CCL5^1667^ and TH^297^. Further, PTEN activates CASP3^1668^, inhibits RAC1^852^, and binds **MAP2K6**^1650^, **CENPC**^1650^, AR^1485^, PPP1CA^1269^, CAV1^801^ and MC1R^1070^.

PTEN also decreases FOXO1 phosphorylation^1669^, increases cytochrome c release^410^ and increases mislocalization of the adaptor protein **PARD3**^850^ (not shown; **PARD3** binds to **FRMD4A**^1670^). PTEN is therefore involved in regulation of apoptosis (CASP3, cytochrome c), autophagy (MTOR) protein modification (**PLOD1**), cholesterol homeostasis (**SREBF1**, CAV1), the immune response (STAT3, NF-KB, CCL5, **HAVCR1**; see also section D2), DA metabolism (TH, PITX3), the MAP kinase pathway (**MAP2K6**) and beta-catenin aggregation (CTNNB1).

##### D1.3.3 TP53

TP53 is activated in response to, among others, oxidative stress and DNA damage. Phosphorylated (i.e. activated) TP53 levels are increased in the SN of the PD brain^251^. TP53 expression and thus the susceptibility to TP53-dependent apoptosis is increased by **SREBF1**^1417^, **GAS2**^1671^, the familial PD protein UCHL1 (increases TP53 accumulation)^434^ and histon demethylase **KDM2B**^1672^ and decreased by the transcriptional repressor **TBX3**^1673^. TP53 is activated by PARK7^1674^ (not shown), GSK3B^1636^, STK11^997^, **CXCR4**^1675^ and **BAG6**^1676, 1677^ and binds **MAP1B**^1678^, SERPINB9^846^ (not shown) and UCHL1^1679^. TP53 increases the cleavage of familial PD protein EIF4G1^1680^ (not shown).

Activated TP53 translocates into the nucleus by binding to **KPNA4**^1681^, where TP53 subsequently binds to **EHMT2**^1682^, **KANSL1** ^1683^ (suggested to be involved in PD risk^1684^), **ATR**^1685^ (**ATR** binds to **HUS1**^1686^, **TP53BP1**^1687^ and transcription factor E2F1^1685^ and E2F1 binds to **MCPH1**^1688^), **TP53BP1**^1689^ (binds to E2F1^1690^), **PRKRIR**^1691^, GSK3B^1692^, SIRT1^650^ and PARK7^436^. Inhibition of the deacetylase activity of SIRT1 by **CCAR2**^655^ increases acetylated TP53 levels and TP53-mediated apoptosis^651^, whereas oxidized PARK7 binds to the TP53 DNA-binding region and thereby inhibits TP53-dependent gene transcription^1693^.

TP53 increases the expression of **AGTR1**^1694^, **COL2A1**^1695^, **COL18A1**^1696, 1697^, **ULK2**^1695^, **MPI**^713^, **PRKG1**^1698^, **FRMD4A**^1699^ (not shown), COMT^282^, **CD200**^1700^ and ICAM1^1701, 1702^ and decreases the expression of **HLA-DQA1**^1703^, **HLA-B**^1704^, **HS3ST1**^1703^, **WDHD1**^1705^, **IL2RA**^1706^, HSPA8^1703^ and **SREBF1**^1407^, showing involvement in regulation of, among others, DA degradation (via COMT), the angiotensin system (via **AGTR1**), autophagy (via **ULK2**), the immune response (via **CD200**, **HLA-DQA1**, **HLA-B**, **IL2RA**), protein degradation pathways (via HSPA8, **PRKG1**) and cholesterol regulation (via **SREBF1**).

##### D1.3.4 CASP3

CASP3 induces apoptosis and is increased in the SN of PD patients^125, 136^. CASP3 is activated by UCHL1^434^, ATG7^958^, **PTPN1**^1707^ (also activates CASP9^1707^), HTRA2^435, 1708^ and CASP9^433^ and inhibited by the TGF-beta propeptide **BMP7**^1709^, JAK2^1710^, SIRT1^660^, **FRMD4A**^1711^, **PRKCE**^1712^, PARK7^436, 1713^ and **SNCA**^1714^, whereas **AGTR1** activation increases CASP3 expression^359^. CASP3 activates the cell death substrate **GAS2**^1715^, which is associated to the cytoskeleton and during apoptosis cleaved by CASP3 causing rearrangements of the cytoskeleton^1715^.

The myristoyltransferase **NMT2** binds to both CASP3 and TP53^1716^. Myristoylation, the transfer of a myristoyl-group, a 14-carbon saturated fatty acid, to a protein, typically promotes membrane binding, which is essential for protein localization and function^1717^. During apoptosis CASP3 mediated cleavage of **NMT2**, results in a relocalization of **NMT2** from the cytoplasm to the plasma membrane^1718^ and CASP3-mediated cleavage of intracellular proteins enables **NMT2** to myristoylate many of these proteins^1717^, resulting in the translocation of these caspase-cleaved and myristoylated proteins to their new membrane locations to affect apoptosis^1719^.

### D2. Immune regulation

The PD landscape includes the regulation of immune cells, that may become activated by the death of DA neurons and/or cause the induction of DA neuron death. More specifically, the main immunological cascades in PD in a dendritic cell, astroglia, microglia, CD4+ T(helper) cell, CD8+ T(helper) cell, B cell and the DA neuron are presented. The next sections discuss the immune cell-specific processes. Yet, note that multiple pathways that are described below are not per se limited to immune cells and may also fulfill functions in DA neurons. Section D2.1 discusses the immune-related pathways and proteins in the PD landscape and section 2.2 discusses the activation of the immune cells of the innate and adaptive immune response.

#### D2.1 Immune-related pathways

STAT3 and the NF-KB-complex both regulate cellular responses to (extracellular) stimuli and both regulate activation of immune-related processes. More specifically, STAT3 and NF-KB are stress-responsive transcription factors that after activation in the cytoplasm bind to, and are transported into the nucleus by nuclear importin alpha 3 (**KPNA4**)^1720-1722^. In the nucleus they regulate transcription together with (among others) earlier mentioned transcription regulators SIRT1, PTEN, GSK3B and CTNNB1, the transcription factors **SREBF1**, **ATF6**, AR, VDR, FOXO1, and MITF and the – in DA neuron development implicated – transcription factors ASCL1, **MSX1**, NEUROG2, NR4A2, PITX3 and SOX2.

##### D2.1.1 JAK2/STAT3

The JAK2-STAT3 pathway transduces extracellular signals over the cell membrane into the cell by binding of JAK2 to multiple cell surface receptors. JAK2 binds to **CXCR4**^1723^, **AGTR1**^358^, the interleukin 5 receptor **IL5RA**^1724^ and binds and is inhibited by **PTPN1**^1725^ and is activated by **FER**^1726^, vitamin D3^1105^, the immune factors IL2^1727^, IL12^1728, 1729^ (not shown) and CCL5^1730^ and the receptors **CXCR4**^1731^, **AGTR1**^356, 357^ and the INS receptor (INSR)^1230^. Subsequently, JAK2 increases activation of NF-KB complexes^1732^ and binds^1733^ and activates the transcription factor STAT3^1734-1736^, which is involved in cell growth, immune response^312^, DA neuronal apoptosis^1737^ and extracellular **SNCA**-mediated neurotoxicity^1738^. STAT3 binds (in addition to JAK2) also to FOXO1^625^, RAC1^1739^ (not shown), **CXCR4**^1723^ (is increased by JAK2^1723^), **PTPRT**^1740^, **PTPN1**^1741^ and **PRKCE**^1742^ and is activated (via JAK2) by **FER**^1726^, IL2^1743^ (not shown), IL6^1744^ (not shown), IL10^1745^ (not shown), IFNG^1746^ (not shown), CXCL12^1747^ (not shown) and mTORC1^1748^ and inhibited (via JAK2 inhibition) by **PTPN1**^1725, 1749^ and **PTPRT**^1740^.

Moreover, STAT3 expression is increased by CCL5^1750^ and decreased by **COL18A1**^752^ and SIRT1^668^ and is indirectly affected by **CCAR2** that binds and inhibits SIRT1 activity^655^.

STAT3 increases the proliferation of T cells by preventing apoptosis^1751^ by limiting their production of IL2 (via upregulation of FOXO1^1752^). Activation of STAT3 results in its nuclear translocation where it acts as a transcription activator^1720^. This translocation of STAT3 is an active process that requires receptor-mediated endocytosis^1753^, e.g. dysregulation of the clathrin-mediated endocytosis regulator **AMPH** disrupts co-localization of STAT3 with endocytic vesicles and subsequent transport to the perinuclear region^1753^. In the nucleus STAT3 binds to **ETV6**^1754^, SIRT1^651^, FOXO1^625^, MTOR^1755^ (not shown), MITF^1063^ and NF-KB^1756-1758^. By binding, STAT3 and NF-KB can influence each other’s transcriptional activity^1758, 1759^ or can collaboratively induce gene expression^1760^. STAT3 also binds to the promoter of IFNG^1761^, IL5^1762^, IL6^1760^ and CCL5^1763^, increases the expression of **IL2RA**^1764^, AKT1^1765^ (not shown), PLAU^1766^, SOD2^1767^, **PROK2**^1768^, CCL5^1760^ (and also CCL5 release^1763^), ITGB2^1769, 1770^ and ICAM1^1211, 1769, 1771^, regulates the expression of **HLA-DRB1**, **HLA-DQA1** and **HLA-C**^1772^ and decreases the expression of transcription factor **SP110**^1766^, MITF^1059^ and **SREBF1**^1408^.

Thus, STAT3 regulates (or is regulated by) the immune response (via NF-KB, IFNG, IL2, IL5, IL6, CCL5, ITGB2, **IL2RA**, ICAM1, **HLA-DRB1**, **HLA-DQA1**, **HLA-C**), regulates gene transcription with multiple other transcription regulators (SIRT1, FOXO1, MITF, NF-KB, **SREBF1**), is affected by the ER UPR (via **PTPN1**) and regulates the AKT1/mTORC1/**SREBF1** pathway (see also ^973^).

##### D2.1.2 NF-KB

The cytoplasmic NF-KB complex (consisting of NFKB1, NFKB2, REL, RELA and RELB) functions as a transcription factor that rapidly acts upon cell stimulation by extracellular stimuli^312^ and regulates autophagy^1773^, immune responses, inflammation, cell growth and apoptosis^312^. PD patients show increased NF-KB levels in the nigrostriatal DA region^201^ and in the nuclei of DA neurons^225^.

The NF-KB-complex is activated by **CPNE4**^507^, **CUL2**^507^ (not shown), **GNA12**^746^, **PRKCE**^1774, 1775^, **PIK3CD**^1776^, **TRAPPC9**^1777^, **PARK2**^1778^, **ZAK**^1779^ and **AGTR1**^360, 361^, regulated by **TRAF3** (inhibits NFKB2^1300^, REL^1780^, RELA^1780^, RELB^1780^ and activates NFKB1^1781^) and inhibited by **PTPN1**^1782^, **SENP7**^1783^ and **NPTX2** (inhibits NF-KB through a TP53-PTEN-PI3K-AKT1 pathway^1784^, regulates excitatory synapse formation, is highly upregulated in PD and is a component of Lewy bodies^80^). Further, NF-KB binds to **CXCR4**^1785^, **CCAR2**^1132^, **RPL7A**^1132^, **CUL2**^1786^ (also binds to **TMCC3**^1134^), **USP9X**^1132^ and **SND1**^1002^, activates **PIK3CD**^1776^ and NF-KB transport into the nucleus is increased by PARK7^1787^. These NF-KB-regulating proteins are involved in a variety of processes, such as signal transduction (**GNA12**, **CXCR4**, **PRKCE**, **PIK3CD**), angiotensin system (**AGTR1**) protein synthesis (**RPL7A**, **SND1**), post-translational modification (**SENP7**), protein ubiquitination and degradation by the E3 ubiquitin ligase complex (**PARK2**, **TRAF3**, **CUL2**, **USP9X**), (apoptotic) transcription (**CCAR2**) and the ER UPR (**PTPN1**). Interestingly, many of these proteins are related to protein synthesis, modification and degradation. Dysregulation of these processes may result in misfolded proteins in the cytoplasm, resulting in increased ER stress and UPR activation.

Further, NF-KB expression is increased by FOXO1^629^ and **MAP2K6**^1284, 1285^ and decreased by PTEN^1664^ and the transcription factor **POU2F1**^1788^ (that also decreases **HLA-DRA**^1789^ and ICAM1^1790^ expression).

Nuclear NF-KB binds to **POU2F1**^1788, 1791^, the histon methyltransferase **EHMT2**^1792^ (that also decreases CXCL12 expression^1793^ and binds to histon modificator **RCOR1**^1794^), GSK3B^1627^, **TP53BP1**^1795^, **SND1**^1002^, **TLE1**^1796^ (not shown), **DDX3X**^1132^, STAT3^1756-1758^ and CCL5^1797^ (not shown). Further, NF-KB increases the expression of the cytokines CCL5^1798, 1799^, CXCL12^1800^, IFNG^1801^, IL1B^1802^, IL2^1803^, IL5^1804^, IL6^1805, 1806^, IL10^1807^, IL12^1108, 1806^ and TNF^1802, 1806^ and of **ABCG1**^1808^, **CREM**^1809^, **CXCR4**^1810, 1811^, **HLA-B**^1812^, **IL2RA**^1803, 1813-1816^ and ICAM1^1798, 1817^. Further, NF-KB decreases the expression of **CYP17A1**^609^ and regulates **PRF1** expression^1818^ (not shown). Thus, NF-KB regulates histon regulation (via **EHMT2**, **RCOR1**), many immune related processes (via CXCL12, IFNG, IL1B, IL2, IL5, IL6, IL10, IL12, TNF STAT3, CCL5, **HLA-B**, **CXCR4**, **IL2RA**, ICAM1, **PRF1**), testosterone production (via **CYP17A1**) and cholesterol regulation (via **ABCG1**).

Also multiple nuclear proteins regulate NF-KB activity, i.e. NF-KB is activated by **MAP2K6**^1283^ and GSK3B^1643^ and inhibited by CTNNB1^1819^ and by the kinase and DNA damage sensor **ATR**^1820^ (inhibits GSK3B-mediated NF-KB activation^1619^) and by a complex consisting of the transcriptional corepressor **TLE1** and SIRT1^652^. Moreover, **TLE1** also binds to GSK3B^884^, the **26S** proteasome^846^ and the transcription factor **RUNX3**^1390^ (decreases expression of AKT1^1821^ and increases expression of IFNG^1822^ (not shown) and **ITGAL**^1823^ (not shown)).

Thus, NF-KB is regulated by signal transduction, protein synthesizing and modifying pathways in the PD landscape, resulting in activation of multiple immune response pathways. Increase in NF-KB, as seen in the DA neurons of PD patients, mirrors the increased stress and the inflammatory state in DA neurons resulting in cell death.

##### D2.1.3 Cell adhesion, cell-cell interaction, axon guidance and immune regulation

Many proteins in the PD landscape are involved in cell adhesion, cell-cell interaction and axon guidance and are hereby also often involved in regulation of immune cells, for immune cells require these same mechanisms for e.g. chemotaxis, co-stimulation and activation. Members of the immunoglobulin superfamily are often associated with the immune system and include proteins involved in antigen presentation, cytokine binding, cell adhesion and co-stimulation. The immunoglobulin-like domains of the immunoglobulin superfamily proteins can be classified as variable (IgV), constant (IgC1 and IgC2) or intermediate (IgI). All PD-GWAS-associated HLA proteins have the IgC1 domain^312^ and can therefore interact with the T cell receptor. Further, **AMIGO2**, **CD200**, **CNTN1**, **DSCAM**, **NEO1**, **NCAM2**, **NEGR1**, **PTPRT**, **ROBO2** and **SEMA3E** all have one or more IgC2 domains^312^, the same domain that is found primarily in the mammalian T cell surface antigens CD2, CD4 and CD80 and the intercellular cell adhesion molecule ICAM1^312^. Furthermore, **HAVCR1** and **BTNL2** have the IgV domain that is also located on the T cell surface antigens CD2 and CD4^312^. Indicating that all these proteins may have a function in the immune system. And, apart from **AMIGO2**, **CNTN1** and **NEGR1** (that are involved in cell-adhesion and axon guidance^1824-1826^) all of these proteins indeed have a known function in the immune response. For instance, **CD200** is involved cell adhesion and in the activation of the immune response (see also section D2.2.1). The axon guidance receptor **DSCAM** is involved in neuronal self-avoidance^312^ and is part of the innate immune system in invertebrates^1827, 1828^. (However, it is unknow if **DSCAM** has a function in the human immune system.) RGMA is expressed by T- and B-cells and inhibits migration of these cells by contact- and chemo-repulsion via its receptor **NEO1**^1829, 1830^, activating RHOA^1831^ (not shown), which regulates the actin cytoskeleton in migration, axon guidance and cell adhesion^312^. **NCAM2** is a cell adhesions molecule that regulates neurite outgrowth^1832^, its expression is increased by IL2 and essential for survival and differentiation of natural killer cells^1833^ (not shown). **PTPRT** is involved in cellular adhesion^312^ and increases dephosphorylation of STAT3 (not shown)^1740^, which is essential in the differentiation of T cells^1834^. SLIT2, the ligand for **ROBO2**^1835, 1836^ has a role in axon guidance and regulates cell migration^312^, but also inhibits CCL5-induced^1837^ and CXCL12-**CXCR4**-induced^1838^ leukocyte chemotaxis (not shown). The secreted semaphorin **SEMA3E** (not shown) inhibits the CXCL12 regulated migration of thymocytes in the thymus^1839^. (**SEMA6D**, the other semaphorin in the GWASs, has no immunoglobulin-like domain, but is nevertheless required for late-phase activation of T cells, and its expression on the membrane of CD4+ T cells is increased after their activation^1840^.) And lastly, **HAVCR1** is expressed on CD4+ T cells and amplifies their activation^1841^, whereas **BTNL2** is a negative regulator of T cell proliferation^312^. As a result, expression of IL2 by T cells is increased by **HAVCR1**^1842^ and decreased by **BTNL2**^1843^.

Other PD-GWAS-associated proteins involved in cell adhesion, cell-cell interaction and/or axon guidance, but without immunoglobulin-like domain, are the integrins **ITGA2B**, **ITGA6** and **ITGA8**, the cadherins **CDH6**, **CDH23** and **PCDH8**, the cell-surface protein **NLGN1**, the contactin **CNTNAP2** and the metalloproteases **ADAM12**, **ADAMTS2** and **ADAMTS14**. The integrines **ITGA2B** (binds **COL2A1**^1844^, is increased by **PRKCE**^1845^ and **SCIN**^1846^), **ITGA6** and **ITGA8** mediate cell-cell interactions, neurite outgrowth and adhesion to the ECM^312^. The Ca^2+^-dependent cadherins CDH2, **CDH6**, **CDH23** and **PCDH8** form adherends junctions for cell-cell interactions. CDH2 binds to **GNA12**^740^ and **AMPK**^1847^, and is probably internalized by interaction with **PCDH8**^312^. **NLGN1** binds **PTPRT**^1848^ and **DLG2**^1849^ and is a postsynaptic cell-adhesion molecule that regulates the formation of excitatory synapses^1850^. **CNTNAP2** regulates the myelination of axons by glia cells^1851^. The metallopeptidase **ADAM12** is involved in amyloid-beta neurotoxicity and neuronal death^1852^. **PRKCE** binds to **ADAM12**^1853^ and increases, like **ANGPT2**^1854^, expression of **ADAM12**^1855^ and increases, like **COL2A1**^1853^, translocation of **ADAM12** to the cell membrane^1856^. At the cell membrane **ADAM12** binds to TGFBR2^1857^ a growth factor receptor that decreases the expression of gap junction protein **GJB2**^1858^, binds to **BAG6**^1859^ and is inhibited by **BAMBI**^1386^. The extracellular pro-collagen N-propeptidases **ADAMTS2** and **ADAMTS14** process procollagens into mature collagens that are able to form collagen fibrils and thereby regulate ECM organization^312, 1860^.

Of all these proteins, only **ITGA2B**, **ITGA6**, **ADAM12** and **ADAMTS2** have explicitly been linked to immune regulation. For **ITGA2B** increases proliferation of T cells^1861^ and secretion of IL6^1862^, **ITGA6** increases microglia activation^1863^, **ADAM12** regulates differentiation of T cells^1864^ and **ADAMTS2** mRNA is increased specifically in monocytes and macrophages after glucocoricoid stimulation^1865^.

Thus, multiple proteins in the landscape regulate cell adhesion and axon guidance and many of those proteins, especially when containing an immunoglobulin-like domain, also have explicit functions in immune cell regulation. The cell adhesion proteins, without an immunoglobulin-like domain (the integrins, cadherins and metalloproteases) are only sporadically linked to immune regulation. However, increasing knowledge about their protein function may show that they are also involved in adhesion, activation and/or migration of immune cells. Overall, dysregulation of these proteins may affect immune cell regulation and ofcourse also neuronal axonal and synaptic functioning.

#### D2.2 Immune cell activation

PD is associated with immune system alterations, such as ratio changes in lymphocyte populations^1866-1869^, immunoglobulin^1870^ and cytokine levels^185, 1871^. Moreover, PD patients show a lower incidence of infections and cancer, suggesting an (over)active immune system^1872^. PD pathogenesis is associated with multiple interleukines, inflammatory factors and cytokines. For example, SNPs in the gene or gene promotor of IL1B^183^, TNF^247-249^, IL6^187^ and IL8^188^ are associated with PD. And, IFNG^178, 179^, CCL5^140, 141^, CXCL12^79^, IL1B^141, 184, 185^, IL2^186^ ^185^, IL4^179, 185^ (not shown), IL6^184^, IL8^141^, IL10^181^ and TNF^141, 179, 250^ all show increased expression levels in PD patients. Further, the receptors (subunits) for IL2, IL5, CCL5 and CXCL12, respectively **IL2RA**, **IL5RA**, **CCR4** and **CXCR4** were found in the GWASs.

Upon cytokine activation the expression of intercellular adhesion molecule 1 (ICAM1) is increased (by IL1B^1873, 1874^, IFNG^1874, 1875^, TNF^1874, 1876^ that increase binding of NF-KB to the ICAM1 promotor and by IL2 that increases binding of STAT3 to the ICAM1 promotor^1877^). ICAM1 regulates cell-cell interactions, adhesion and proliferation and is expressed on many (immune) cell types, i.e. it is expressed on astroglia^176^, dendritic cells^1878^, microglia^1879^, T cells^1880-1882^, B cells^1883, 1884^ and vascular endothelial cells^1885^ and subsequently functions as a ligand for the integrin lymphocyte function-associated antigen-1 **LFA-1**^1886-1889^ and thereby attracts immune cells^1887, 1889, 1890^. **LFA-1** is a complex of integrin alpha-L (**ITGAL**) and integrin beta-2 (ITGB2)^1891, 1892^ that is expressed on circulating leukocytes and mediates rolling on ICAM1, which makes leukocytes bind to vascular endothelial cells, so they can enter the target tissue^1893^. The actin anchor **ACTN4** binds to ICAM1^1894^ and is required for leukocyte extravasation^1894^.

In addition to cytokines, ICAM1 expression is also increased by **ANGPT2**^1895^, TP53^1701^, FOXO1^641^, **COL2A1**^1896^, familial PD protein **PARK7**^1787^, JAK2 activation^1897, 1898^ and by INS that increases binding of STAT3 to the ICAM1 promotor^1211^. Further, ICAM1 expression is decreased by **HLX**^1899^, **BMP7**^1900^, **POU2F1**^1790^, **COL18A1**^752^ and IL10 (decreases NF-KB binding to the ICAM1 promotor^1875^). ICAM1 itself increases expression of **IL2RA** on CD8+ T cells^1901^ (not shown), decreases the expression of **SREBF1**^1410^ and binds **IL2RA**^1902^ and **ACTN4**^1894^. Furthermore, cholesterol^1903, 1904^, VLDL^1905^, 7-ketocholesterol^1169^ (not shown), LDL and oxLDL^1906^ increase expression of ICAM1, showing a role for lipids in activating the immune response.

(Sphingo)lipids (e.g. cholesterol, ceramide and sphingomyelin) are important in regulation of the immune response, i.e. shingomyelinase and ceramide inhibit IL2 production^1907^, ceramide increases expression and secretion of the proinflammatory interleukin IL8^1908, 1909^, expression of intercellular adhesion molecule 1 (ICAM1)^1910^ and is involved in the secretion of IL6^1911^. Ceramide and sphingomyelin are themselves also regulated by cytokines that are elevated in PD, i.e. IL4 decreases the quantity of ceramide^1912^ (not shown) and IFNG decreases the quantity of sphingomyelin^1913^ (not shown). Lipid rafts on the membrane also regulate clathrin-independent constitutive endocytosis of the lipid raft-associated interleukin-2 receptor (IL2R, subunit **IL2RA** in GWASs)^1914^, recycling of the immune related integrin complex **LFA-1** to the plasma membrane^1915^ and **LFA-1**-mediated adhesion of T cells and primary T cells^1916^.

Therefore, a proper balance of sphingolipids and cholesterol in the cell is mandatory to maintain a normal immune regulation, for their dysregulation directly affects the regulation of cytokines and migration of immune cells. Moreover, membrane organization is of crucial importance for antigen-presenting cells to capture and present antigens, i.e. membrane lipid rafts are involved in regulation of endocytosis, membrane trafficking and activation of the immune response^781, 782^. Discrimination between ‘self’ and ‘non-self’ antigens during antigen presentation is one of the key elements of the adaptive immune system (see section D2.2.2). The proteins required for antigen presentation, the MHC class I proteins **HLA-B** and **HLA-C** and the MHC class II proteins **HLA-DQA1**, **HLA-DQA2**, **HLA-DQB1**, **HLA-DQB2**, **HLA-DRA**, **HLA-DRB1**, were found in the PD GWASs, and the HLA gene region has been associated to PD^71-73^. Whereas MHC class I peptides for antigen presentation to CD8+ T cells are derived from **26s**-processed cytoplasmic proteins, MHC class II peptides for presentation to CD4+ cells are acquired via endocytosis of antigens^1917^. This shows the importance of the proteasome and the endosomal system in immune cell activation. For example, disruption of lipid rafts inhibits internalization of **SNCA** by microglia^1918^ and affects localization of MHC class II proteins on the plasma membrane of dendritic and B cells^782, 1919^, which again affects T cell activation^1919^.

In summary, activation of the immune response, production of cytokines, immune cell migration and regulation of antigen presentation (which requires the endosomal/lysosomal system (see also section B2) for antigen processing) are regulate by (sphingo)lipids and are highly associated with PD. The next sections discuss the activation of the innate (D2.2.1) and adaptive (D2.2.2) immune response and the involvement of coagulation factors in immune regulation (D2.2.3).

##### D2.2.1 Activation of the innate immune response

The innate immune system is the first line of defense against infection, and consists of epithelial barriers, astroglia^1920^, phagocytes (e.g. microglia, denritic cells) and the complement system. Activated astroglia secrete cytokines (e.g. IL6) that regulate both innate and adaptive immune responses^1920^. **SNCA** stimulates astroglia to produce IL6 and ICAM1^1921^, and together with the ability of **SNCA** to activate microglia, this can attract microglia to the site of inflammation^1922^. Indeed, the astroglia cells in the SN of PD patients, show increased ICAM1 expression, which is associated with increased **LFA-1** positive microglia in the SN^176^. Moreover, activated, ICAM1 and **LFA-1** positive, microglia are increased in the SN and striatum of PD patients^50, 1923^ and based on *in vitro* studies contribute to the death of DA neurons^1924-1926^. Multiple factors such as (extracellular) **SNCA**, NM, S1P and cholesterol activate microglia ^1927-1930^, whereas oxLDL/oxysterols ameliorates microglia activation via the LXR receptor^1170, 1930^. **SNCA**-mediated microglia activation increases the expression of MHC class II proteins, leading to CD4+ Th cell proliferation (part of the adaptive immune response) and DA neurodegeneration^1931^. Further, NM-mediated microglia activation is NF-KB-dependent^1927, 1932^ and results in the production of pro-inflammatory cytokines^1932^. The interaction of glycoprotein **CD200**, located on the plasma membrane of a neuron, with its receptor CD200R1 (reduced expression in PD patients^144^) on the microglia holds the microglia in a quiescent state^1933^. In a PD rat model impaired **CD200**-CD200R1 function resulted in an increase in microglia activation and increased degeneration of DA neurons^1933, 1934^. **CD200** expression is inversely correlated with expression of ICAM1^1933^ and **CD200** decreases the expression of **ITGAL** and thus **LFA-1**^1935^ and thereby controls the regulation and signaling of immune cells. Microglia activation is also increased by **ITGA6**^1863^, IL5 and **IL5RA**^1936^, **STAP1**^1937^ and IFNG^178^ and inhibited by **RCOR1**, that thereby prevents inflammation-induced death of DA neurons^1525^. In contrast, **RCOR1** also inhibits astroglia activation^1525^, whereas astroglia activation (by **ATF6**) results in neuronal survival in an MPTP mice model^1392^. This indicates that a delicate balance in microglia and astroglia activation and inhibition is necessary for DA neuron survival. Moreover, extracellular NM also stimulates the maturation of dendritic cells^1938^. Dendritic cell levels are reduced in blood of PD patients (maybe due to recruitment to inflammatory sites) and negatively associated with motor symptom severity^1939^.

Activated microglia and dendritic cells start secreting the pro-inflammatory factors IL1B, IL12 (IL12 is a heterodimeric protein, encoded by IL12A and IL12B^312^), TNF and CCL5^1929, 1940-1942^. IL1B increases NF-KB-dependent ICAM1 expression on glial cells^1873^, increases the expression of NR4A2^1528^ (not shown) and CCL5^1941^ and decreases the expression of **HLA-DRA**^1943^. IL12 secretion by microglia is inhibited by IL10 and astroglia^1940^ (not shown) and its expression is decreased by **TRAF3**^1780, 1944^ and blockage of Ca^2+^ influx^1108^ (e.g. via **CACNA1A** and **CACNA2D3**), but increased by **PRKCE**^1945^. In dendritic cells the intramembrane-cleaving aspartic protease **SPPL2B** promotes proteolysis of TNF to trigger IL12 production^1946^. Secreted IL12 increases the expression of **IL2RA**^1947^, **ITGA6**^1948^, **HLX**^1949^ and CCL5^1950^ and decreases the expression of the CCL5 receptor **CCR4** ^1951^.

Lastly, the complement protein **C9** is located in lewy bodies in PD SN^52^ and is a subunit of the membrane attack complex that forms pores in the plasma membrane of target cells^312^. **The plasma protease C1 inhibitor (SERPING1**) inhibits the complement system^1952^ (not shown).

Thus, the innate immune response is activated in PD pathology and NM- and **SNCA**-dependent activation of dendritic cells and microglia results in the presentation of SN antigens to the adaptive immune system^1938, 1953^. This indicates that autoimmune responses to DA neuron-specific antigen may play a role in the pathology of PD. Of note, whereas cholesterol and oxysterols both increase ICAM1 expression, they have opposing effects on microglia activation.

##### D2.2.2 Activation of the adaptive immune response

The adaptive immune system is unlike the innate immune system highly specific to the invading pathogens and is activated when the innate immune system is not sufficient. Cytokines secreted by cells of the innate immune system recruit and activate the cells of the adaptive immune response, (CD4+ and CD8+) T cells and B cells, to the site of inflammation, i.e. IL1B stimulates T and B cell maturation and proliferation^1954^, IL12 regulates differentiation of naive T cells into CD4+ T cells^1955^ and enhances the activity of naïve CD8+ T cells^1956^, whereas TNF activates CD4+ T cells^1957, 1958^. And lastly, CCL5 is a chemoattractant for T cells^312^ and increases the expression of IL1B^1959^ and STAT3^1750^.

The next sections will show in more detail the processes involved in migration and chemotaxis of T and B cells (D2.2.2.1) and the activation of T (D2.2.2.2) and B cells (D2.2.2.3).

###### D2.2.2.1 Migration and chemotaxis of T and B cells

In addition to ICAM1-mediated attraction of immune cells (see section D2.2), B and T cell migration is inhibited via contact repulsion by the repulsion guidance molecule RGMA together with its receptor **NEO1** ^1829^. They thereby regulate lymphocyte infiltration in the tissue and suppress the inflammatory response ^1829^. Moreover, chemotaxis is also regulated by **PARD3**, **CCR4**, CXCL12-**CXCR4** interaction and S1P, i.e. **PARD3** regulates polarization and chemotaxis of T cells^1960^ and **CCR4** is a chemoattractant homing receptor on circulating T cells^312^ and microglia^1961^, for the T cell attractant CCL5^1750^.

CXCL12 activates **RAP2A** (not shown), which is essential for B cell migration^1962^. Cholesterol increases the binding of CXCL12 to **CXCR4**^1963^, which activates **CXCR4** resulting in increased intracellular Ca^2+^ levels^312^, activation of the JAK2/STAT3 and NF-KB pathways (see sections D2.1.1 and D2.1.2) and an increased expression of **LFA-1**, which increases adhesion of B cells to ICAM1^1731, 1887, 1964, 1965^ (not shown). Interestingly, **CXCR4** is increased in the nigro-striatal system of PD patients^79^ and CXCL12 and **CXCR4** are both increased in a mouse model preceding DA neuron loss^79^. Moreover, **CXCR4** binds the familial PD protein ATP13A2^866^ and **CXCR4** distribution on the cell membrane is regulated by the CXCL12-activated^1966^ **RAP1A**^1967^.

And lastly, S1P is transported out of the cell by **SPNS2**^734^, to create a S1P gradient that attracts T and B cells^1968^. S1P is transported by the lipoproteins LDL and HDL^1969^ (HDL is the major S1P carrier^1970^ and has a 9-fold higher S1P content than LDL^1969^). HDL-S1P inhibits GSK3B^1621^, activates STAT3 and increases cell migration^1971^. STAT3 increases the expression of the S1P receptor (not shown), creating a positive feedback loop^1972^. S1P activates both STAT3 and NF-KB^1973^.

In summary, migration of T and B cells to their target tissue is regulated by ICAM1-**LFA1**, RGMA-**NEO1**, CCL5-**CCR4** and CXCL12-**CXCR4** ligand-receptor interactions and S1P gradients. Further, cholesterol, oxysterols and lipoproteins regulate the activation and expression of ICAM1 and **LFA1** and are therefore important in astroglia and microglia mediated activation of T and B cells. An increase of these factors in the PD SN indicates that T and B cell activation may be increased.

###### D2.2.2.2 T cell activation

In the blood of PD patients, CD4+ T cells subsets^1866-1868^ and CD4+:CD8+ T cell ratios^1867^ are decreased, and may reflect T cell alterations in brain. CD4+ T cell activation by peptides presented on MHC Class II proteins^1917^, results in expression and secretion of IL2^1974, 1975^, IL5^1974^, CCL5^1976^ and IFNG^1974^. IL2^1977, 1978^ activates T cells and is together with **IL2RA** required for T cell proliferation^1979^. IL5 stimulates differentiation of B cells into immunoglobulin secreting cells^312^ and is involved in microglia activation and proliferation^1936, 1980^. Further, CCL5 and also **NEDD9** increase secretion of IL2^1981, 1982^ and IFNG activates microglia-mediated DA neuron death^178^. Thus, IL2, IL5, CCL5 and IFNG are involved in several reinforcing feedback loops of T cell and microglia activation and proliferation. T cell activation and proliferation is also amplified by the interaction between **TIMD4** (expressed on antigen presenting cells) and its ligand **HAVCR1** (expressed on T cells early after their activation^1841, 1983^. And, **PIK3CD** activation contributes to T cell development, migration and differentiation^312^.

MHC Class I proteins (e.g. **HLA-B** and **HLA-C**) are located on almost all cells and present peptides to the immune system. CD8+ T cells become activated when these peptides are not recognized as endogenous and subsequently destroy the antigen presenting cell. The nuclear protein **RUNX3** regulates development of CD8+ T cells^1984^ and increases CD8+ T cell proliferation^1985^. (Activated) CD8+ T cells contain **TIAL1**^1986^ and **PRF1**^1987^ (binds cholesterol^1988^) in their cytotoxic granules which are secreted into the cleft between the T-cell and its target cell. **LAMP1** regulates **PRF1** quantity and localization to these granules^1989^. **PRF1** makes pores in the plasma membrane of the target cell^1990^ and **TIAL1** induces DNA fragmentation^1986^. Membrane permeabilization by **PRF1** makes it possible for granozymes and factors like **TIAL1** to enter the target cell and induce DNA fragmentation and apoptosis^1986, 1991^. **TIAL1** and **PRF1** are both regulated by IL2, i.e. it decreases **TIAL1**^466^ and increases **PRF1**^1818^ expression. Further, **PRF1** expression is increased by IL12^1992^, which also increases activation of MTOR in CD8+ T cells^1993^.

SERPINB9 (not shown) is a factor that **protects cells against cytotoxic T cell-mediated apoptosis^1994^** and is upregulated by STAT3^1766^, IL1B^1995^ and IFNG^1996^ and binds to TP53^846^, **TLE1**^846^ and **PLEKHM1**^846^ (**PLEKHM1** regulates vesicle acidification^1025^ and the endocytic and autophagic pathway^1026^).

In the PD landscape, several nuclear proteins regulate T cell proliferation and activation, i.e. **TCF12** increases differentiation of T cells^1997^, **RAI1** protects against auto-immune reactions by inhibiting lymphocyte activation^1998^ and differentiation of T cells into Th1 and Th17 subsets^1999^ and STAT3 activation is essential for the differentiation of helper T cells^1834, 2000^. Further, **HLX** is involved in the maturation of Th1 cells^2001^ and regulates expression of ICAM1 and **LFA-1**^1899^ (which are important for T cell-T cell interaction, adhesion and proliferation^1882^) and Th1-specific gene expression^2002^. **DRG2** overexpression on the other hand, suppresses T cell growth^2003^.

In summary, proliferation and activation of T cells, especially CD4+ and CD8+ T cells, is regulated by multiple proteins in the PD landscape. CD4+ T cells assist in B cell activation (see D2.2.2.3) and CD8+ cells destroy their target cell. Dysregulation in the activation or functioning of these cells may increase inflammation or autoimmune responses that increase DA neuron death.

###### D2.2.2.3 B cell activation

B cell activation occurs directly via antigen recognition by the B cell receptor (BCR) or with the assistance of CD4+ T cells. After antigen recognition the B cell presents a peptide of the antigen on MHC Class II proteins, which can be recognized by CD4+ T cells to provide costimulation and trigger B cell activation and proliferation. Now, the B cells migrate out of the lymphoid follicle (B cell zone) and a part of the activated B cells migrate into the germinal center for affinity maturation^2004^. Circulating B cells levels are reduced in the blood of PD patients^1866^, and activation, differentiation, affinity maturation and migration of B cells is regulated by multiple proteins in the PD landscape. Activation of naive B cells is inhibited by **BANK1**, a protein that is highly expressed in B cells^2005, 2006^. After activation of the BCR, **BANK1** is phosphorylated and increases the mobilization of Ca^2+^ in B cells^2005^ (not shown), inhibits AKT1 activation^2006^ (not shown) and binds to STAT3^2007^. Also, the transcriptional regulator **RAI1** inhibits B cell activation and proliferation, by inhibiting BCR signaling^1998^. Activation of the BCR increases the activation of **STAP1**^2008, 2009^. **PIK3CD** is required for BCR signaling and regulates B-cell development, proliferation, migration^312^. Further, the proteins **LRRC25**, **BST1** and **TCF12** activate and regulate development and growth of B cells^2010-2014^. Furthermore, **TCF12** increases B cell differentiation^2015^, increases the expression of FOXO1^627^ and decreases the expression of gap junction protein **GJB2**^2016^. FOXO1 deficiency impairs B-cell development^2017^ and is important in regulation of the PI3K-AKT1 axis in B-cell development^2017^, the same axis that is inhibited by **BANK1** (see above).

During co-stimulation the CD4+ T cell secretes the cytokines IL2, IL4 and IL5 that bind to their receptors (e.g. **IL2RA** and **IL5RA**) on the B cell and trigger B cell activation and proliferation^2018-2022^. CRH decreases the expression of **IL2RA**^2023^, but is also bound and inactivated by secreted **CRHBP**^312^. JAK2 binds to and is activated by the IL5 receptor subunit **IL5RA**^1724, 2022^. Further, IL5 and IL12 increase the expression of **HLX**^1949, 2024^, which is low expressed in inactive B (and T) cells, but high in activated lymphocytes^2025^, is involved in differentiation of B cells^2026^ and regulates IFNG expression^1949, 2027^.

B cell trafficking and recirculation through lymphoid tissues, which is required for efficient antigen presentation and subsequent activation of B cells, is regulated by CXCL12 and **CXCR4**^2028^. **CXCR4** is expressed on B cells and quickly downregulated after CXCL12 binding^2029^. CXCL12 is produced in the cell layers surrounding the germinal center and attracts naive and memory B cells, but not germinal center B cells^2029^. This shows that the responsiveness to the chemoattractant CXCL12 is regulated during activation of B cells, to increase their trafficking to germinal centers^2029^. Moreover, adherens of B cells to ICAM1 is impaired in **NEDD9** knockout B cells, resulting in a reduced migration to secondary lymphoid organs^2030^ for affinity maturation. B cells have to interact with antigen presenting cells in germinal centers to survive^2004^ and the **LFA-1**-ICAM1 interaction contributes to B cell selection and promotes their survival^2031^. **RUNX3** is expressed in the late stages of B cells development into plasma cells^2032, 2033^ and increases expression of **ITGAL** resulting in an increased **LFA-1** surface expression^1823^, which would therefore be beneficial in B cell selection and survival. **ZNF385B** (associated with PD in three GWASs) is expressed in germinal center B cells and binds to TP53^2034^ (not shown). Depending on the **ZNF385B** isoform, **ZNF385B** can have both pro- and anti-apoptotic actions in the B cell and can therefore affect B cell selection^2034^.

B cell isotope class switching occurs in germinal centers. MTOR deficiency in B cells leads to a reduced high-affinity antibody production by decreasing the activity of activation-induced (DNA-cytosine) deaminase (**AICDA, not shown**). This enzyme causes DNA mutations and thereby leads to antibody diversity^2035^ and thus is necessary for isotope class switching. **TRAF3** increases the expression of **AICDA^2036^** (not shown), but also inhibits (together with TRAF2) mature B cell survival and suppresses expression of NF-KB (^1300, 2037^. NF-KB binds to the AICDA promotor and is required for its expression^2038^ (not shown). This discrepancy with inhibition of NF-KB expression by **TRAF3** is explained by **TRAF3**-mediated inhibition of NF-KB inhibitors^2039, 2040^. Further, to induce isotope class switching, **AICDA is actively transported into the nucleus of B cells by the importin KPNA4^2041^ (not shown).**

S1P induces adhesion to ICAM1 and thereby allows B cells and plasma (B) cells to exit the germinal centers and re-enter the circulation^2042^. Circulating B cells not only bind to ICAM1 on other cells, but can also express ICAM1 themselves and thereby costimulate T cells^2043^.

In conclusion, the reduced levels of circulating B cells in PD and the regulation of their activation, differentiation, affinity maturation and migration by proteins in the PD landscape indicate that their regulation is part of the PD pathology. Changes in B cell activation by CD4+ T cells or by changes in their selection and maturation may affect the immune response in PD.

##### D2.2.3 Coagulation factors, lipoproteins and immune cell activation

Coagulation factors regulate the formation of a platelet and fibrin clot in a damaged vessel wall and dysregulation of this process can lead to increased clotting (thrombosis) or increased bleeding (hemorrhage). PD patients show a decrease in the serum coagulation factors α-2-antiplasmin and factor V^66^. Factor V deficiency leads to reduced clotting and hemorrhage^2044^, whereas a decrease in α-2-antiplasmin would increase plasmin activity^2045^. The PLG gene encodes the protein plasminogen, that is converted into plasmin by the plasminogen activators PLAU and PLAT^312^. Extracellular **SNCA** regulates the plasmin system by increasing SERPINE1 expression^2046^ (a serine protease inhibitor that mediates diabetic vascular complications and is increased in the plasma of patients with diabetes, obesity and hypertension^2047-2050^). SERPINE1 binds and inhibits both PLAU^2051-2053^ (PLAU increases proteolysis of **MMRN1**^2054^) and PLAT^2045, 2055^. SERPINE1, PLAT and PLAU bind and are internalized by the low-density lipoprotein receptor-related protein 1B (**LRP1B**)^2056^ and VLDLR binds to plasminogen and PLAU and mediates their endocytosis^2057^. Further, SERPINE1 increases cholesterol in the blood^2050^ and plasmin increases degradation of LDL^2057, 2058^, whereas plasminogen knockout mice show decreased HDL-cholesterol levels^2059^. Furthermore, SERPINE1 expression and secretion is increased by VLDL^2060, 2061^, VLDLR^2061, 2062^, LDL^2061^ and OxLDL^2061^ (enhances SERPINE1 expression compared to LDL induced expression^2061^) and PLAT expression is decreased by LDL^2063^. Thus coagulation factors and lipoproteins regulate each other. Knockout of PLAT in a mouse model decreased levels of dopamine, indicating a role for the PLAT/plasmin system in regulating dopamine release^2064^.

In addition to lipoproteins and **SNCA**, SERPINE1, PLAT and PLAU expression is regulated by multiple other proteins in the PD landscape. Namely, SERPINE1 expression is also increased by **SREBF1**^1416, 2065^, **ADAM12**^1857^ (not shown), **BAG6**^1859^, **BMP7**^608, 2066^, STK11^995^ (not shown), INS^639^ (not shown), **NEDD9**^2067^ (not shown) and **AGTR1**^363^, and is decreased by **COL18A1**^2068^, **PRKG1**^2069^ (not shown), FOXO1^639^, **SMAD5**^2070^, vitamin D3 (via inhibition of NF-KB)^1106^ and the cytoplasmic serine-threonine kinase **WNK1**^2071^ (that binds and is phosphorylated by **STK39**^2072^). Further, SERPINE1 expression is increased by mRNA stabilization by TP53^2073^ (not shown), SERPINE1 accumulation is decreased by **BMP7**^2070^ and SERPINE1 activation inhibited by SIRT1^661^. Thus, SERPINE1 expression is regulated by lipoproteins (VLDL, LDL, oxLDL, **LRP1B**), the angiotensin system (**AGTR1**) and by four (**SREBF1**, FOXO1, NF-KB and TP53) of the main transcription regulatory pathways in the PD landscape, which therefore can affect SERPINE1-mediated inhibition of PLAU and PLAT. Moreover, the secreted latent-transforming growth factor beta-binding protein 1 (**LTBP1**) binds the extracellular protein Fibrillin-1 (**FBN1**)^2074^, is activated by plasmin^2045^ and increases the expression of **COL2A1**^2075^ and SERPINE1^2076^ and is thus an important regulator in the ECM.

PLAU is inhibited by IL1B^2077^ and activated by a splice variant of **TMPRSS9** (highly expressed in CD8+ T cells)^2078^, whereas PLAT is inhibited by LDL^2079^ and activated by CRH via its receptor **CRHR1**^2080^. PLAU expression is decreased by **COL18A1**^2068^, IL12^2081^, PTEN^1666^, mediated by CXCL12^2082^ (not shown) and increased by **MAP2K6**^2083^ (not shown), **AGTR1**^364^, STAT3^1766^, NF-KB^2084^ (not shown), IFNG^2085^, IL1B^2085^ and TNF^2085^ (not shown). PLAT expression is decreased by IL1B^2086^ and increased by vitamin D3^1098^. So, in addition to SERPINE1, also PLAU and PLAT are directly regulated by PD landscape proteins, affecting plasminogen / plasmin levels.

Of note, coagulation factors also have a role in immune cell regulation. PLAT activates microglia^2087, 2088^ and PLAU increases the expression of ICAM1^2089^. Moreover, PLG is expressed by microglia^2090^, and plasmin cleaves and degrades both aggregated and monomeric forms of **SNCA**^2046^ and thereby inhibits the translocation of extracellular **SNCA** into neighboring cells and the activation of microglia and astroglia by extracellular **SNCA**^2046^. Plasmin also triggers chemotaxis of dendritic cells triggering a T cell response^2091^ and induces the release of IL8 from **SDC1** (a cell surface proteoglycan that stabilizes the chemoattractant form of IL8 at the cell surface)^2092^. SERPINE1 (by inhibiting plasmin production) stabilizes the chemoattractant function of IL8 by stabilizing its binding to **SDC1**^2092^. Further, PARK7 increases expression of IL8^1787^ and PD patients with a **GBA** mutation have increased IL8 plasma levels compared to PD patients without a **GBA** mutation^2093^. The latter indicates an involvement of sphingosine, which is indeed shown by a S1P-mediated suppression of IL8 secretion by T cells^2094^ (not shown). PLAT triggers activation of SPHK1 by binding to its receptor PLAUR^2095^ (not shown) and thereby increases the phosphorylation of sphingosine to S1P, a major regulator of T- and B-cell traficking^2096^.

In summary, coagulation factors, regulated by cytokines, lipoproteins and **SNCA**, regulate the chemotaxis of immune cells via IL8- and S1P-regulation and prevent **SNCA**-mediated activation of astroglia and microglia. Moreover, coagulation factors regulate lipoproteins, sphinglipids and cholesterol and can therefore modify their effects on immune cell activation.

### D3. Concluding remarks

The viability of DA neurons is maintained by DA neuron specific transcription factors, increased by neurotrophic factors and is decreased by activation and upregulation of pro-apoptotic proteins. The transcription factors ASCL1, **MSX1**, NEUROG2, NR4A2, PITX3 and SOX2 are necessary to maintain a dopaminergic phenotype. The neurotrophic factors BDNF and GDNF support the survival of DA neurons by regulating apoptotic pathways, whereas GSK3B, PTEN, TP53 and CASP3 increase apoptotic signaling and neuron death. Further, these neurotrophic and apoptotic proteins regulate, together with the transcription factors STAT3 and NF-KB, immune cell activation. In PD patients, cytokines and cytokine receptors are dysregulated and both the innate and adaptive immune response are affected. The innate immune response is activated by NM, **SNCA** and cholesterol, and results in the presentation of SN-specific antigens to the adaptive immune system that subsequently targets these cells for destruction. Migration, maturation, proliferation and activation of T and B cells (cells of the adaptive immune system) is regulated by proteins in the PD landscape. Moreover, almost all transcription factor pathways in the PD landscape regulate the immune regulator ICAM1, which stresses the importance of this protein in (dysfunctional) immune regulation in PD. Changed (oxidized) lipoprotein levels affect the immune response, directly, by regulating immune cell activation (and ICAM1 expression), or indirectly due to changes in membrane lipid composition, affecting (among others) internalization of antigens and localization of receptors responsible for chemotaxis or immune cell activation. Lastly, coagulation factors (regulated by cytokines, lipoproteins and **SNCA**) regulate the chemotaxis of immune cells, prevent **SNCA**-induced immune cell activation, but also regulate lipoproteins, sphingolipids and cholesterol and thereby mediate their effects on the immune response.

## REFERENCES

1. Maraganore DM, de Andrade M, Lesnick TG, Strain KJ, Farrer MJ, Rocca WA, et al. High-resolution whole-genome association study of Parkinson disease. Am J Hum Genet. 2005;77(5):685-93.

2. Fung HC, Scholz S, Matarin M, Simon-Sanchez J, Hernandez D, Britton A, et al. Genome-wide genotyping in Parkinson's disease and neurologically normal controls: first stage analysis and public release of data. Lancet Neurol. 2006;5(11):911-6.

3. Pankratz N, Wilk JB, Latourelle JC, DeStefano AL, Halter C, Pugh EW, et al. Genomewide association study for susceptibility genes contributing to familial Parkinson disease. Hum Genet. 2009;124(6):593-605.

4. Latourelle JC, Pankratz N, Dumitriu A, Wilk JB, Goldwurm S, Pezzoli G, et al. Genomewide association study for onset age in Parkinson disease. BMC Med Genet. 2009;10:98.

5. Satake W, Nakabayashi Y, Mizuta I, Hirota Y, Ito C, Kubo M, et al. Genome-wide association study identifies common variants at four loci as genetic risk factors for Parkinson's disease. Nat Genet. 2009;41(12):1303-7.

6. Simon-Sanchez J, Schulte C, Bras JM, Sharma M, Gibbs JR, Berg D, et al. Genome-wide association study reveals genetic risk underlying Parkinson's disease. Nat Genet. 2009;41(12):1308-12.

7. Edwards TL, Scott WK, Almonte C, Burt A, Powell EH, Beecham GW, et al. Genome-wide association study confirms SNPs in SNCA and the MAPT region as common risk factors for Parkinson disease. Ann Hum Genet. 2010;74(2):97-109.

8. Hamza TH, Zabetian CP, Tenesa A, Laederach A, Montimurro J, Yearout D, et al. Common genetic variation in the HLA region is associated with late-onset sporadic Parkinson's disease. Nat Genet. 2010;42(9):781-5.

9. Spencer CC, Plagnol V, Strange A, Gardner M, Paisan-Ruiz C, Band G, et al. Dissection of the genetics of Parkinson's disease identifies an additional association 5' of SNCA and multiple associated haplotypes at 17q21. Hum Mol Genet. 2011;20(2):345-53.

10. Saad M, Lesage S, Saint-Pierre A, Corvol JC, Zelenika D, Lambert JC, et al. Genome-wide association study confirms BST1 and suggests a locus on 12q24 as the risk loci for Parkinson's disease in the European population. Hum Mol Genet. 2011;20(3):615-27.

11. Simon-Sanchez J, van Hilten JJ, van de Warrenburg B, Post B, Berendse HW, Arepalli S, et al. Genome-wide association study confirms extant PD risk loci among the Dutch. Eur J Hum Genet. 2011;19(6):655-61.

12. Do CB, Tung JY, Dorfman E, Kiefer AK, Drabant EM, Francke U, et al. Web-based genome-wide association study identifies two novel loci and a substantial genetic component for Parkinson's disease. PLoS Genet. 2011;7(6):e1002141.

13. Liu X, Cheng R, Verbitsky M, Kisselev S, Browne A, Mejia-Sanatana H, et al. Genome-wide association study identifies candidate genes for Parkinson's disease in an Ashkenazi Jewish population. BMC Med Genet. 2011;12:104.

14. Hernandez DG, Nalls MA, Ylikotila P, Keller M, Hardy JA, Majamaa K, et al. Genome wide assessment of young onset Parkinson's disease from Finland. PLoS One. 2012;7(7):e41859.

15. Soreq L, Israel Z, Bergman H, Soreq H. Advanced microarray analysis highlights modified neuro-immune signaling in nucleated blood cells from Parkinson's disease patients. J Neuroimmunol. 2008;201-202:227-36.

16. Lehnert S, Jesse S, Rist W, Steinacker P, Soininen H, Herukka SK, et al. iTRAQ and multiple reaction monitoring as proteomic tools for biomarker search in cerebrospinal fluid of patients with Parkinson's disease dementia. Exp Neurol. 2012;234(2):499-505.

17. Bossers K, Meerhoff G, Balesar R, van Dongen JW, Kruse CG, Swaab DF, et al. Analysis of gene expression in Parkinson's disease: possible involvement of neurotrophic support and axon guidance in dopaminergic cell death. Brain Pathol. 2009;19(1):91-107.

18. Durrenberger PF, Grunblatt E, Fernando FS, Monoranu CM, Evans J, Riederer P, et al. Inflammatory Pathways in Parkinson's Disease; A BNE Microarray Study. Parkinsons Dis. 2012;2012:214714.

19. Botta-Orfila T, Tolosa E, Gelpi E, Sanchez-Pla A, Marti MJ, Valldeoriola F, et al. Microarray expression analysis in idiopathic and LRRK2-associated Parkinson's disease. Neurobiol Dis. 2012;45(1):462-8.

20. Riley BE, Gardai SJ, Emig-Agius D, Bessarabova M, Ivliev AE, Schule B, et al. Systems-based analyses of brain regions functionally impacted in Parkinson's disease reveals underlying causal mechanisms. PLoS One. 2014;9(8):e102909.

21. Cantuti-Castelvetri I, Keller-McGandy C, Bouzou B, Asteris G, Clark TW, Frosch MP, et al. Effects of gender on nigral gene expression and parkinson disease. Neurobiol Dis. 2007;26(3):606-14.

22. Simunovic F, Yi M, Wang Y, Macey L, Brown LT, Krichevsky AM, et al. Gene expression profiling of substantia nigra dopamine neurons: further insights into Parkinson's disease pathology. Brain. 2009;132(Pt 7):1795-809.

23. Simunovic F, Yi M, Wang Y, Stephens R, Sonntag KC. Evidence for gender-specific transcriptional profiles of nigral dopamine neurons in Parkinson disease. PLoS One. 2010;5(1):e8856.

24. Infante J, Prieto C, Sierra M, Sanchez-Juan P, Gonzalez-Aramburu I, Sanchez-Quintana C, et al. Identification of candidate genes for Parkinson's disease through blood transcriptome analysis in LRRK2-G2019S carriers, idiopathic cases, and controls. Neurobiol Aging. 2015;36(2):1105-9.

25. Licker V, Cote M, Lobrinus JA, Rodrigo N, Kovari E, Hochstrasser DF, et al. Proteomic profiling of the substantia nigra demonstrates CNDP2 overexpression in Parkinson's disease. J Proteomics. 2012;75(15):4656-67.

26. Jin J, Hulette C, Wang Y, Zhang T, Pan C, Wadhwa R, et al. Proteomic identification of a stress protein, mortalin/mthsp70/GRP75: relevance to Parkinson disease. Mol Cell Proteomics. 2006;5(7):1193-204.

27. Lill CM, Roehr JT, McQueen MB, Kavvoura FK, Bagade S, Schjeide BM, et al. Comprehensive research synopsis and systematic meta-analyses in Parkinson's disease genetics: The PDGene database. PLoS Genet. 2012;8(3):e1002548.

28. Pankratz N, Beecham GW, DeStefano AL, Dawson TM, Doheny KF, Factor SA, et al. Meta-analysis of Parkinson's disease: identification of a novel locus, RIT2. Ann Neurol. 2012;71(3):370-84.

29. Chen YP, Song W, Huang R, Chen K, Zhao B, Li J, et al. GAK rs1564282 and DGKQ rs11248060 increase the risk for Parkinson's disease in a Chinese population. J Clin Neurosci. 2013;20(6):880-3.

30. Rhodes SL, Sinsheimer JS, Bordelon Y, Bronstein JM, Ritz B. Replication of GWAS associations for GAK and MAPT in Parkinson's disease. Ann Hum Genet. 2011;75(2):195-200.

31. Lin CH, Chen ML, Tai YC, Yu CY, Wu RM. Reaffirmation of GAK, but not HLA-DRA, as a Parkinson's disease susceptibility gene in a Taiwanese population. Am J Med Genet B Neuropsychiatr Genet. 2013;162B(8):841-6.

32. Shulman JM, Yu L, Buchman AS, Evans DA, Schneider JA, Bennett DA, et al. Association of Parkinson disease risk loci with mild parkinsonian signs in older persons. JAMA Neurol. 2014;71(4):429-35.

33. Chu Y, Dodiya H, Aebischer P, Olanow CW, Kordower JH. Alterations in lysosomal and proteasomal markers in Parkinson's disease: relationship to alpha-synuclein inclusions. Neurobiol Dis. 2009;35(3):385-98.

34. Dehay B, Bove J, Rodriguez-Muela N, Perier C, Recasens A, Boya P, et al. Pathogenic lysosomal depletion in Parkinson's disease. J Neurosci. 2010;30(37):12535-44.

35. Polymeropoulos MH, Lavedan C, Leroy E, Ide SE, Dehejia A, Dutra A, et al. Mutation in the alpha-synuclein gene identified in families with Parkinson's disease. Science. 1997;276(5321):2045-7.

36. Kruger R, Kuhn W, Muller T, Woitalla D, Graeber M, Kosel S, et al. Ala30Pro mutation in the gene encoding alpha-synuclein in Parkinson's disease. Nat Genet. 1998;18(2):106-8.

37. Chartier-Harlin MC, Kachergus J, Roumier C, Mouroux V, Douay X, Lincoln S, et al. Alpha-synuclein locus duplication as a cause of familial Parkinson's disease. Lancet. 2004;364(9440):1167-9.

38. Ibanez P, Bonnet AM, Debarges B, Lohmann E, Tison F, Pollak P, et al. Causal relation between alpha-synuclein gene duplication and familial Parkinson's disease. Lancet. 2004;364(9440):1169-71.

39. Singleton AB, Farrer M, Johnson J, Singleton A, Hague S, Kachergus J, et al. alpha-Synuclein locus triplication causes Parkinson's disease. Science. 2003;302(5646):841.

40. Irizarry MC, Growdon W, Gomez-Isla T, Newell K, George JM, Clayton DF, et al. Nigral and cortical Lewy bodies and dystrophic nigral neurites in Parkinson's disease and cortical Lewy body disease contain alpha-synuclein immunoreactivity. J Neuropathol Exp Neurol. 1998;57(4):334-7.

41. Bayer TA, Jakala P, Hartmann T, Havas L, McLean C, Culvenor JG, et al. Alpha-synuclein accumulates in Lewy bodies in Parkinson's disease and dementia with Lewy bodies but not in Alzheimer's disease beta-amyloid plaque cores. Neurosci Lett. 1999;266(3):213-6.

42. Vogt IR, Lees AJ, Evert BO, Klockgether T, Bonin M, Wullner U. Transcriptional changes in multiple system atrophy and Parkinson's disease putamen. Exp Neurol. 2006;199(2):465-78.

43. Rott R, Szargel R, Haskin J, Bandopadhyay R, Lees AJ, Shani V, et al. alpha-Synuclein fate is determined by USP9X-regulated monoubiquitination. Proc Natl Acad Sci U S A. 2011;108(46):18666-71.

44. Lesnick TG, Papapetropoulos S, Mash DC, Ffrench-Mullen J, Shehadeh L, de Andrade M, et al. A genomic pathway approach to a complex disease: axon guidance and Parkinson disease. PLoS Genet. 2007;3(6):e98.

45. McNaught KS, Jenner P. Proteasomal function is impaired in substantia nigra in Parkinson's disease. Neurosci Lett. 2001;297(3):191-4.

46. Zhang Y, James M, Middleton FA, Davis RL. Transcriptional analysis of multiple brain regions in Parkinson's disease supports the involvement of specific protein processing, energy metabolism, and signaling pathways, and suggests novel disease mechanisms. Am J Med Genet B Neuropsychiatr Genet. 2005;137b(1):5-16.

47. Moran LB, Duke DC, Deprez M, Dexter DT, Pearce RK, Graeber MB. Whole genome expression profiling of the medial and lateral substantia nigra in Parkinson's disease. Neurogenetics. 2006;7(1):1-11.

48. Miller RM, Kiser GL, Kaysser-Kranich TM, Lockner RJ, Palaniappan C, Federoff HJ. Robust dysregulation of gene expression in substantia nigra and striatum in Parkinson's disease. Neurobiol Dis. 2006;21(2):305-13.

49. Zhang J, Sokal I, Peskind ER, Quinn JF, Jankovic J, Kenney C, et al. CSF multianalyte profile distinguishes Alzheimer and Parkinson diseases. American journal of clinical pathology. 2008;129(4):526-9.

50. Imamura K, Hishikawa N, Sawada M, Nagatsu T, Yoshida M, Hashizume Y. Distribution of major histocompatibility complex class II-positive microglia and cytokine profile of Parkinson's disease brains. Acta Neuropathol. 2003;106(6):518-26.

51. Dumitriu A, Latourelle JC, Hadzi TC, Pankratz N, Garza D, Miller JP, et al. Gene expression profiles in Parkinson disease prefrontal cortex implicate FOXO1 and genes under its transcriptional regulation. PLoS Genet. 2012;8(6):e1002794.

52. Yamada T, McGeer PL, McGeer EG. Lewy bodies in Parkinson's disease are recognized by antibodies to complement proteins. Acta Neuropathol. 1992;84(1):100-4.

53. Gao J, Nalls MA, Shi M, Joubert BR, Hernandez DG, Huang X, et al. An exploratory analysis on gene-environment interactions for Parkinson disease. Neurobiol Aging. 2012;33(10):2528 e1-6.

54. Kitada T, Asakawa S, Hattori N, Matsumine H, Yamamura Y, Minoshima S, et al. Mutations in the parkin gene cause autosomal recessive juvenile parkinsonism. Nature. 1998;392(6676):605-8.

55. Hattori N, Kitada T, Matsumine H, Asakawa S, Yamamura Y, Yoshino H, et al. Molecular genetic analysis of a novel Parkin gene in Japanese families with autosomal recessive juvenile parkinsonism: evidence for variable homozygous deletions in the Parkin gene in affected individuals. Ann Neurol. 1998;44(6):935-41.

56. Lucking CB, Abbas N, Durr A, Bonifati V, Bonnet AM, de Broucker T, et al. Homozygous deletions in parkin gene in European and North African families with autosomal recessive juvenile parkinsonism. The European Consortium on Genetic Susceptibility in Parkinson's Disease and the French Parkinson's Disease Genetics Study Group. Lancet. 1998;352(9137):1355-6.

57. Lucking CB, Bonifati V, Periquet M, Vanacore N, Brice A, Meco G. Pseudo-dominant inheritance and exon 2 triplication in a family with parkin gene mutations. Neurology. 2001;57(5):924-7.

58. Bonifati V, Lucking CB, Fabrizio E, Periquet M, Meco G, Brice A. Three parkin gene mutations in a sibship with autosomal recessive early onset parkinsonism. J Neurol Neurosurg Psychiatry. 2001;71(4):531-4.

59. Kann M, Jacobs H, Mohrmann K, Schumacher K, Hedrich K, Garrels J, et al. Role of parkin mutations in 111 community-based patients with early-onset parkinsonism. Ann Neurol. 2002;51(5):621-5.

60. Maruyama M, Ikeuchi T, Saito M, Ishikawa A, Yuasa T, Tanaka H, et al. Novel mutations, pseudo-dominant inheritance, and possible familial affects in patients with autosomal recessive juvenile parkinsonism. Ann Neurol. 2000;48(2):245-50.

61. Rawal N, Periquet M, Lohmann E, Lucking CB, Teive HA, Ambrosio G, et al. New parkin mutations and atypical phenotypes in families with autosomal recessive parkinsonism. Neurology. 2003;60(8):1378-81.

62. Abbas N, Lucking CB, Ricard S, Durr A, Bonifati V, De Michele G, et al. A wide variety of mutations in the parkin gene are responsible for autosomal recessive parkinsonism in Europe. French Parkinson's Disease Genetics Study Group and the European Consortium on Genetic Susceptibility in Parkinson's Disease. Hum Mol Genet. 1999;8(4):567-74.

63. Hedrich K, Marder K, Harris J, Kann M, Lynch T, Meija-Santana H, et al. Evaluation of 50 probands with early-onset Parkinson's disease for Parkin mutations. Neurology. 2002;58(8):1239-46.

64. Sutherland G, Mellick G, Sue C, Chan DK, Rowe D, Silburn P, et al. A functional polymorphism in the parkin gene promoter affects the age of onset of Parkinson's disease. Neurosci Lett. 2007;414(2):170-3.

65. Tan EK, Shen H, Tan JM, Lim KL, Fook-Chong S, Hu WP, et al. Differential expression of splice variant and wild-type parkin in sporadic Parkinson's disease. Neurogenetics. 2005;6(4):179-84.

66. Zhang X, Yin X, Yu H, Liu X, Yang F, Yao J, et al. Quantitative proteomic analysis of serum proteins in patients with Parkinson's disease using an isobaric tag for relative and absolute quantification labeling, two-dimensional liquid chromatography, and tandem mass spectrometry. Analyst. 2012;137(2):490-5.

67. Baloh RH, Salavaggione E, Milbrandt J, Pestronk A. Familial parkinsonism and ophthalmoplegia from a mutation in the mitochondrial DNA helicase twinkle. Arch Neurol. 2007;64(7):998-1000.

68. Kiferle L, Orsucci D, Mancuso M, Lo Gerfo A, Petrozzi L, Siciliano G, et al. Twinkle mutation in an Italian family with external progressive ophthalmoplegia and parkinsonism: a case report and an update on the state of art. Neurosci Lett. 2013;556:1-4.

69. Elstner M, Morris CM, Heim K, Bender A, Mehta D, Jaros E, et al. Expression analysis of dopaminergic neurons in Parkinson's disease and aging links transcriptional dysregulation of energy metabolism to cell death. Acta Neuropathol. 2011;122(1):75-86.

70. Kim JM, Lee KH, Jeon YJ, Oh JH, Jeong SY, Song IS, et al. Identification of genes related to Parkinson's disease using expressed sequence tags. DNA Res. 2006;13(6):275-86.

71. Hill-Burns EM, Factor SA, Zabetian CP, Thomson G, Payami H. Evidence for more than one Parkinson's disease-associated variant within the HLA region. PLoS One. 2011;6(11):e27109.

72. Wissemann WT, Hill-Burns EM, Zabetian CP, Factor SA, Patsopoulos N, Hoglund B, et al. Association of Parkinson disease with structural and regulatory variants in the HLA region. Am J Hum Genet. 2013;93(5):984-93.

73. Guo Y, Deng X, Zheng W, Xu H, Song Z, Liang H, et al. HLA rs3129882 variant in Chinese Han patients with late-onset sporadic Parkinson disease. Neurosci Lett. 2011;501(3):185-7.

74. Li Y, Grupe A, Rowland C, Holmans P, Segurado R, Abraham R, et al. Evidence that common variation in NEDD9 is associated with susceptibility to late-onset Alzheimer's and Parkinson's disease. Hum Mol Genet. 2008;17(5):759-67.

75. Shehadeh LA, Yu K, Wang L, Guevara A, Singer C, Vance J, et al. SRRM2, a potential blood biomarker revealing high alternative splicing in Parkinson's disease. PLoS One. 2010;5(2):e9104.

76. Nalls MA, Plagnol V, Hernandez DG, Sharma M, Sheerin UM, Saad M, et al. Imputation of sequence variants for identification of genetic risks for Parkinson's disease: a meta-analysis of genome-wide association studies. Lancet. 2011;377(9766):641-9.

77. Sharma M, Ioannidis JP, Aasly JO, Annesi G, Brice A, Van Broeckhoven C, et al. Large-scale replication and heterogeneity in Parkinson disease genetic loci. Neurology. 2012;79(7):659-67.

78. Chen ML, Lin CH, Lee MJ, Wu RM. BST1 rs11724635 interacts with environmental factors to increase the risk of Parkinson's disease in a Taiwanese population. Parkinsonism Relat Disord. 2014;20(3):280-3.

79. Shimoji M, Pagan F, Healton EB, Mocchetti I. CXCR4 and CXCL12 expression is increased in the nigro-striatal system of Parkinson's disease. Neurotox Res. 2009;16(3):318-28.

80. Moran LB, Hickey L, Michael GJ, Derkacs M, Christian LM, Kalaitzakis ME, et al. Neuronal pentraxin II is highly upregulated in Parkinson's disease and a novel component of Lewy bodies. Acta Neuropathol. 2008;115(4):471-8.

81. Paisan-Ruiz C, Jain S, Evans EW, Gilks WP, Simon J, van der Brug M, et al. Cloning of the gene containing mutations that cause PARK8-linked Parkinson's disease. Neuron. 2004;44(4):595-600.

82. Zimprich A, Biskup S, Leitner P, Lichtner P, Farrer M, Lincoln S, et al. Mutations in LRRK2 cause autosomal-dominant parkinsonism with pleomorphic pathology. Neuron. 2004;44(4):601-7.

83. Nichols WC, Pankratz N, Hernandez D, Paisan-Ruiz C, Jain S, Halter CA, et al. Genetic screening for a single common LRRK2 mutation in familial Parkinson's disease. Lancet. 2005;365(9457):410-2.

84. Di Fonzo A, Rohe CF, Ferreira J, Chien HF, Vacca L, Stocchi F, et al. A frequent LRRK2 gene mutation associated with autosomal dominant Parkinson's disease. Lancet. 2005;365(9457):412-5.

85. Gilks WP, Abou-Sleiman PM, Gandhi S, Jain S, Singleton A, Lees AJ, et al. A common LRRK2 mutation in idiopathic Parkinson's disease. Lancet. 2005;365(9457):415-6.

86. Lwin A, Orvisky E, Goker-Alpan O, LaMarca ME, Sidransky E. Glucocerebrosidase mutations in subjects with parkinsonism. Mol Genet Metab. 2004;81(1):70-3.

87. Sidransky E, Nalls MA, Aasly JO, Aharon-Peretz J, Annesi G, Barbosa ER, et al. Multicenter analysis of glucocerebrosidase mutations in Parkinson's disease. N Engl J Med. 2009;361(17):1651-61.

88. Aharon-Peretz J, Rosenbaum H, Gershoni-Baruch R. Mutations in the glucocerebrosidase gene and Parkinson's disease in Ashkenazi Jews. N Engl J Med. 2004;351(19):1972-7.

89. Spitz M, Rozenberg R, Pereira Lda V, Reis Barbosa E. Association between Parkinson's disease and glucocerebrosidase mutations in Brazil. Parkinsonism Relat Disord. 2008;14(1):58-62.

90. De Marco EV, Annesi G, Tarantino P, Rocca FE, Provenzano G, Civitelli D, et al. Glucocerebrosidase gene mutations are associated with Parkinson's disease in southern Italy. Mov Disord. 2008;23(3):460-3.

91. Nichols WC, Pankratz N, Marek DK, Pauciulo MW, Elsaesser VE, Halter CA, et al. Mutations in GBA are associated with familial Parkinson disease susceptibility and age at onset. Neurology. 2009;72(4):310-6.

92. Mao XY, Burgunder JM, Zhang ZJ, An XK, Zhang JH, Yang Y, et al. Association between GBA L444P mutation and sporadic Parkinson's disease from Mainland China. Neurosci Lett. 2010;469(2):256-9.

93. Dos Santos AV, Pestana CP, Diniz KR, Campos M, Abdalla-Carvalho CB, de Rosso AL, et al. Mutational analysis of GIGYF2, ATP13A2 and GBA genes in Brazilian patients with early-onset Parkinson's disease. Neurosci Lett. 2010;485(2):121-4.

94. Moraitou M, Hadjigeorgiou G, Monopolis I, Dardiotis E, Bozi M, Vassilatis D, et al. beta-Glucocerebrosidase gene mutations in two cohorts of Greek patients with sporadic Parkinson's disease. Mol Genet Metab. 2011;104(1-2):149-52.

95. Choi JM, Kim WC, Lyoo CH, Kang SY, Lee PH, Baik JS, et al. Association of mutations in the glucocerebrosidase gene with Parkinson disease in a Korean population. Neurosci Lett. 2012;514(1):12-5.

96. Kumar KR, Ramirez A, Gobel A, Kresojevic N, Svetel M, Lohmann K, et al. Glucocerebrosidase mutations in a Serbian Parkinson's disease population. Eur J Neurol. 2013;20(2):402-5.

97. Gonzalez-Del Rincon Mde L, Monroy Jaramillo N, Suarez Martinez AI, Yescas Gomez P, Boll Woehrlen MC, Lopez Lopez M, et al. The L444P GBA mutation is associated with early-onset Parkinson's disease in Mexican Mestizos. Clin Genet. 2013;84(4):386-7.

98. Gegg ME, Burke D, Heales SJ, Cooper JM, Hardy J, Wood NW, et al. Glucocerebrosidase deficiency in substantia nigra of parkinson disease brains. Ann Neurol. 2012;72(3):455-63.

99. Li NN, Tan EK, Chang XL, Mao XY, Zhao DM, Zhang JH, et al. MCCC1/LAMP3 reduces risk of sporadic Parkinson's disease in Han Chinese. Acta Neurol Scand. 2013;128(2):136-9.

100. Wang YQ, Tang BS, Yu RL, Li K, Liu ZH, Xu Q, et al. Association analysis of STK39, MCCC1/LAMP3 and sporadic PD in the Chinese Han population. Neurosci Lett. 2014;566:206-9.

101. Kolisek M, Sponder G, Mastrototaro L, Smorodchenko A, Launay P, Vormann J, et al. Substitution p.A350V in Na(+)/Mg(2+) Exchanger SLC41A1, Potentially Associated with Parkinson's Disease, Is a Gain-of-Function Mutation. PLoS One. 2013;8(8):e71096.

102. Tucci A, Nalls MA, Houlden H, Revesz T, Singleton AB, Wood NW, et al. Genetic variability at the PARK16 locus. Eur J Hum Genet. 2010;18(12):1356-9.

103. Yan Y, Tian J, Mo X, Zhao G, Yin X, Pu J, et al. Genetic variants in the RAB7L1 and SLC41A1 genes of the PARK16 locus in Chinese Parkinson's disease patients. Int J Neurosci. 2011;121(11):632-6.

104. Pihlstrom L, Axelsson G, Bjornara KA, Dizdar N, Fardell C, Forsgren L, et al. Supportive evidence for 11 loci from genome-wide association studies in Parkinson's disease. Neurobiol Aging. 2013;34(6):1708 e7-13.

105. Michelakakis H, Xiromerisiou G, Dardiotis E, Bozi M, Vassilatis D, Kountra PM, et al. Evidence of an association between the scavenger receptor class B member 2 gene and Parkinson's disease. Mov Disord. 2012;27(3):400-5.

106. Gan-Or Z, Bar-Shira A, Dahary D, Mirelman A, Kedmi M, Gurevich T, et al. Association of sequence alterations in the putative promoter of RAB7L1 with a reduced parkinson disease risk. Arch Neurol. 2012;69(1):105-10.

107. Pamphlett R, Morahan JM, Luquin N, Yu B. An approach to finding brain-situated mutations in sporadic Parkinson's disease. Parkinsonism Relat Disord. 2012;18(1):82-5.

108. Noureddine MA, Li YJ, van der Walt JM, Walters R, Jewett RM, Xu H, et al. Genomic convergence to identify candidate genes for Parkinson disease: SAGE analysis of the substantia nigra. Mov Disord. 2005;20(10):1299-309.

109. Knott C, Stern G, Kingsbury A, Welcher AA, Wilkin GP. Elevated glial brain-derived neurotrophic factor in Parkinson's diseased nigra. Parkinsonism Relat Disord. 2002;8(5):329-41.

110. Papuc E, Kurzepa J, Kurys-Denis E, Grabarska A, Krupski W, Rejdak K. Humoral response against glial derived antigens in Parkinson's disease. Neurosci Lett. 2014;566:77-81.

111. Abdi F, Quinn JF, Jankovic J, McIntosh M, Leverenz JB, Peskind E, et al. Detection of biomarkers with a multiplex quantitative proteomic platform in cerebrospinal fluid of patients with neurodegenerative disorders. J Alzheimers Dis. 2006;9(3):293-348.

112. Buervenich S, Carmine A, Galter D, Shahabi HN, Johnels B, Holmberg B, et al. A rare truncating mutation in ADH1C (G78Stop) shows significant association with Parkinson disease in a large international sample. Arch Neurol. 2005;62(1):74-8.

113. Timmons S, Coakley MF, Moloney AM, C ON. Akt signal transduction dysfunction in Parkinson's disease. Neurosci Lett. 2009;467(1):30-5.

114. Pulkes T, Papsing C, Mahasirimongkol S, Busabaratana M, Kulkantrakorn K, Tiamkao S. Association between apolipoprotein E genotypes and Parkinson's disease. J Clin Neurosci. 2011;18(10):1333-5.

115. Gao J, Huang X, Park Y, Liu R, Hollenbeck A, Schatzkin A, et al. Apolipoprotein E genotypes and the risk of Parkinson disease. Neurobiol Aging. 2011;32(11):2106 e1-6.

116. Gallegos-Arreola MP, Figuera LE, Ortiz GG, Jimenez-Gil FJ, Ramirez-Vega J, Ruiz-Sandoval JL, et al. Apolipoprotein E genotypes in Mexican patients with Parkinson's disease. Dis Markers. 2009;27(5):225-30.

117. Wilhelmus MM, Bol JG, Van Haastert ES, Rozemuller AJ, Bu G, Drukarch B, et al. Apolipoprotein E and LRP1 Increase Early in Parkinson's Disease Pathogenesis. Am J Pathol. 2011;179(5):2152-6.

118. Guo J, Sun Z, Xiao S, Liu D, Jin G, Wang E, et al. Proteomic analysis of the cerebrospinal fluid of Parkinson's disease patients. Cell Res. 2009;19(12):1401-3.

119. Deng H, Yang H, Le W, Deng X, Xu H, Xiong W, et al. Examination of the MASH1 gene in patients with Parkinson's disease. Biochem Biophys Res Commun. 2010;392(4):548-50.

120. Chen D, Zhu C, Wang X, Feng X, Pang S, Huang W, et al. A novel and functional variant within the ATG5 gene promoter in sporadic Parkinson's disease. Neurosci Lett. 2013;538:49-53.

121. Chen D, Pang S, Feng X, Huang W, Hawley RG, Yan B. Genetic analysis of the ATG7 gene promoter in sporadic Parkinson's disease. Neurosci Lett. 2013;534:193-8.

122. Duke DC, Moran LB, Kalaitzakis ME, Deprez M, Dexter DT, Pearce RK, et al. Transcriptome analysis reveals link between proteasomal and mitochondrial pathways in Parkinson's disease. Neurogenetics. 2006;7(3):139-48.

123. Di Fonzo A, Chien HF, Socal M, Giraudo S, Tassorelli C, Iliceto G, et al. ATP13A2 missense mutations in juvenile parkinsonism and young onset Parkinson disease. Neurology. 2007;68(19):1557-62.

124. Lin CH, Tan EK, Chen ML, Tan LC, Lim HQ, Chen GS, et al. Novel ATP13A2 variant associated with Parkinson disease in Taiwan and Singapore. Neurology. 2008;71(21):1727-32.

125. Tatton NA. Increased caspase 3 and Bax immunoreactivity accompany nuclear GAPDH translocation and neuronal apoptosis in Parkinson's disease. Exp Neurol. 2000;166(1):29-43.

126. Horowitz JM, Pastor DM, Goyal A, Kar S, Ramdeen N, Hallas BH, et al. BAX protein-immunoreactivity in midbrain neurons of Parkinson's disease patients. Brain Res Bull. 2003;62(1):55-61.

127. Parsian A, Sinha R, Racette B, Zhao JH, Perlmutter JS. Association of a variation in the promoter region of the brain-derived neurotrophic factor gene with familial Parkinson's disease. Parkinsonism Relat Disord. 2004;10(4):213-9.

128. Bialecka M, Kurzawski M, Roszmann A, Robowski P, Sitek EJ, Honczarenko K, et al. BDNF G196A (Val66Met) polymorphism associated with cognitive impairment in Parkinson's disease. Neurosci Lett. 2014;561:86-90.

129. Guerini FR, Beghi E, Riboldazzi G, Zangaglia R, Pianezzola C, Bono G, et al. BDNF Val66Met polymorphism is associated with cognitive impairment in Italian patients with Parkinson's disease. Eur J Neurol. 2009;16(11):1240-5.

130. Chauhan NB, Siegel GJ, Lee JM. Depletion of glial cell line-derived neurotrophic factor in substantia nigra neurons of Parkinson's disease brain. J Chem Neuroanat. 2001;21(4):277-88.

131. Scalzo P, Kummer A, Bretas TL, Cardoso F, Teixeira AL. Serum levels of brain-derived neurotrophic factor correlate with motor impairment in Parkinson's disease. J Neurol. 2010;257(4):540-5.

132. Ventriglia M, Zanardini R, Bonomini C, Zanetti O, Volpe D, Pasqualetti P, et al. Serum brain-derived neurotrophic factor levels in different neurological diseases. Biomed Res Int. 2013;2013:901082.

133. Salehi Z, Mashayekhi F. Brain-derived neurotrophic factor concentrations in the cerebrospinal fluid of patients with Parkinson's disease. J Clin Neurosci. 2009;16(1):90-3.

134. Hurley MJ, Brandon B, Gentleman SM, Dexter DT. Parkinson's disease is associated with altered expression of CaV1 channels and calcium-binding proteins. Brain. 2013;136(Pt 7):2077-97.

135. Basso M, Giraudo S, Corpillo D, Bergamasco B, Lopiano L, Fasano M. Proteome analysis of human substantia nigra in Parkinson's disease. Proteomics. 2004;4(12):3943-52.

136. Mogi M, Togari A, Kondo T, Mizuno Y, Komure O, Kuno S, et al. Caspase activities and tumor necrosis factor receptor R1 (p55) level are elevated in the substantia nigra from parkinsonian brain. J Neural Transm. 2000;107(3):335-41.

137. Kawamoto Y, Ito H, Ayaki T, Takahashi R. Immunohistochemical localization of apoptosome-related proteins in Lewy bodies in Parkinsons disease and dementia with Lewy bodies. Brain Res. 2014;1571:39-48.

138. Viswanath V, Wu Y, Boonplueang R, Chen S, Stevenson FF, Yantiri F, et al. Caspase-9 activation results in downstream caspase-8 activation and bid cleavage in 1-methyl-4-phenyl-1,2,3,6-tetrahydropyridine-induced Parkinson's disease. J Neurosci. 2001;21(24):9519-28.

139. Darvish H, Heidari A, Hosseinkhani S, Movafagh A, Khaligh A, Jamshidi J, et al. Biased homozygous haplotypes across the human caveolin 1 upstream purine complex in Parkinson's disease. J Mol Neurosci. 2013;51(2):389-93.

140. Rentzos M, Nikolaou C, Andreadou E, Paraskevas GP, Rombos A, Zoga M, et al. Circulating interleukin-15 and RANTES chemokine in Parkinson's disease. Acta Neurol Scand. 2007;116(6):374-9.

141. Reale M, Iarlori C, Thomas A, Gambi D, Perfetti B, Di Nicola M, et al. Peripheral cytokines profile in Parkinson's disease. Brain Behav Immun. 2009;23(1):55-63.

142. Gangemi S, Basile G, Merendino RA, Epifanio A, Di Pasquale G, Ferlazzo B, et al. Effect of levodopa on interleukin-15 and RANTES circulating levels in patients affected by Parkinson's disease. Mediators Inflamm. 2003;12(4):251-3.

143. Pey P, Pearce RK, Kalaitzakis ME, Griffin WS, Gentleman SM. Phenotypic profile of alternative activation marker CD163 is different in Alzheimer's and Parkinson's disease. Acta Neuropathol Commun. 2014;2:21.

144. Luo XG, Zhang JJ, Zhang CD, Liu R, Zheng L, Wang XJ, et al. Altered regulation of CD200 receptor in monocyte-derived macrophages from individuals with Parkinson's disease. Neurochem Res. 2010;35(4):540-7.

145. Kiyohara C, Miyake Y, Koyanagi M, Fujimoto T, Shirasawa S, Tanaka K, et al. Genetic polymorphisms involved in dopaminergic neurotransmission and risk for Parkinson's disease in a Japanese population. BMC Neurol. 2011;11:89.

146. Klebe S, Golmard JL, Nalls MA, Saad M, Singleton AB, Bras JM, et al. The Val158Met COMT polymorphism is a modifier of the age at onset in Parkinson's disease with a sexual dimorphism. J Neurol Neurosurg Psychiatry. 2013;84(6):666-73.

147. Malagelada C, Ryu EJ, Biswas SC, Jackson-Lewis V, Greene LA. RTP801 is elevated in Parkinson brain substantia nigral neurons and mediates death in cellular models of Parkinson's disease by a mechanism involving mammalian target of rapamycin inactivation. J Neurosci. 2006;26(39):9996-10005.

148. Vilarino-Guell C, Rajput A, Milnerwood AJ, Shah B, Szu-Tu C, Trinh J, et al. DNAJC13 mutations in Parkinson disease. Hum Mol Genet. 2013.

149. Edvardson S, Cinnamon Y, Ta-Shma A, Shaag A, Yim YI, Zenvirt S, et al. A deleterious mutation in DNAJC6 encoding the neuronal-specific clathrin-uncoating co-chaperone auxilin, is associated with juvenile parkinsonism. PLoS One. 2012;7(5):e36458.

150. Grevle L, Guzey C, Hadidi H, Brennersted R, Idle JR, Aasly J. Allelic association between the DRD2 TaqI A polymorphism and Parkinson's disease. Mov Disord. 2000;15(6):1070-4.

151. McGuire V, Van Den Eeden SK, Tanner CM, Kamel F, Umbach DM, Marder K, et al. Association of DRD2 and DRD3 polymorphisms with Parkinson's disease in a multiethnic consortium. J Neurol Sci. 2011;307(1-2):22-9.

152. Hoglinger GU, Breunig JJ, Depboylu C, Rouaux C, Michel PP, Alvarez-Fischer D, et al. The pRb/E2F cell-cycle pathway mediates cell death in Parkinson's disease. Proc Natl Acad Sci U S A. 2007;104(9):3585-90.

153. Chartier-Harlin MC, Dachsel JC, Vilarino-Guell C, Lincoln SJ, Lepretre F, Hulihan MM, et al. Translation initiator EIF4G1 mutations in familial Parkinson disease. Am J Hum Genet. 2011;89(3):398-406.

154. Shojaee S, Sina F, Banihosseini SS, Kazemi MH, Kalhor R, Shahidi GA, et al. Genome-wide linkage analysis of a Parkinsonian-pyramidal syndrome pedigree by 500 K SNP arrays. Am J Hum Genet. 2008;82(6):1375-84.

155. Di Fonzo A, Dekker MC, Montagna P, Baruzzi A, Yonova EH, Correia Guedes L, et al. FBXO7 mutations cause autosomal recessive, early-onset parkinsonian-pyramidal syndrome. Neurology. 2009;72(3):240-5.

156. Murakami T, Shoji M, Imai Y, Inoue H, Kawarabayashi T, Matsubara E, et al. Pael-R is accumulated in Lewy bodies of Parkinson's disease. Ann Neurol. 2004;55(3):439-42.

157. Imai Y, Soda M, Inoue H, Hattori N, Mizuno Y, Takahashi R. An unfolded putative transmembrane polypeptide, which can lead to endoplasmic reticulum stress, is a substrate of Parkin. Cell. 2001;105(7):891-902.

158. Kwok JB, Hallupp M, Loy CT, Chan DK, Woo J, Mellick GD, et al. GSK3B polymorphisms alter transcription and splicing in Parkinson's disease. Ann Neurol. 2005;58(6):829-39.

159. Nagao M, Hayashi H. Glycogen synthase kinase-3beta is associated with Parkinson's disease. Neurosci Lett. 2009;449(2):103-7.

160. Wills J, Jones J, Haggerty T, Duka V, Joyce JN, Sidhu A. Elevated tauopathy and alpha-synuclein pathology in postmortem Parkinson's disease brains with and without dementia. Exp Neurol. 2010;225(1):210-8.

161. Abbott RD, Ross GW, Tanner CM, Andersen JK, Masaki KH, Rodriguez BL, et al. Late-life hemoglobin and the incidence of Parkinson's disease. Neurobiol Aging. 2012;33(5):914-20.

162. Shephard F, Greville-Heygate O, Marsh O, Anderson S, Chakrabarti L. A mitochondrial location for haemoglobins--dynamic distribution in ageing and Parkinson's disease. Mitochondrion. 2014;14(1):64-72.

163. Sinha A, Patel S, Singh MP, Shukla R. Blood proteome profiling in case controls and Parkinson's disease patients in Indian population. Clin Chim Acta. 2007;380(1-2):232-4.

164. Sinha A, Srivastava N, Singh S, Singh AK, Bhushan S, Shukla R, et al. Identification of differentially displayed proteins in cerebrospinal fluid of Parkinson's disease patients: a proteomic approach. Clin Chim Acta. 2009;400(1-2):14-20.

165. Mutez E, Nkiliza A, Belarbi K, de Broucker A, Vanbesien-Mailliot C, Bleuse S, et al. Involvement of the immune system, endocytosis and EIF2 signaling in both genetically determined and sporadic forms of Parkinson's disease. Neurobiol Dis. 2014;63:165-70.

166. Schipper HM, Liberman A, Stopa EG. Neural heme oxygenase-1 expression in idiopathic Parkinson's disease. Exp Neurol. 1998;150(1):60-8.

167. Mateo I, Infante J, Sanchez-Juan P, Garcia-Gorostiaga I, Rodriguez-Rodriguez E, Vazquez-Higuera JL, et al. Serum heme oxygenase-1 levels are increased in Parkinson's disease but not in Alzheimer's disease. Acta Neurol Scand. 2010;121(2):136-8.

168. Ayuso P, Martinez C, Lorenzo-Betancor O, Pastor P, Luengo A, Jimenez-Jimenez FJ, et al. A polymorphism located at an ATG transcription start site of the heme oxygenase-2 gene is associated with classical Parkinson's disease. Pharmacogenet Genomics. 2011;21(9):565-71.

169. Costa-Mallen P, Checkoway H, Zabeti A, Edenfield MJ, Swanson PD, Longstreth WT, Jr., et al. The functional polymorphism of the hemoglobin-binding protein haptoglobin influences susceptibility to idiopathic Parkinson's disease. Am J Med Genet B Neuropsychiatr Genet. 2008;147B(2):216-22.

170. Zhao X, Xiao WZ, Pu XP, Zhong LJ. Proteome analysis of the sera from Chinese Parkinson's disease patients. Neurosci Lett. 2010;479(2):175-9.

171. Alvarez-Erviti L, Rodriguez-Oroz MC, Cooper JM, Caballero C, Ferrer I, Obeso JA, et al. Chaperone-mediated autophagy markers in Parkinson disease brains. Arch Neurol. 2010;67(12):1464-72.

172. Strauss KM, Martins LM, Plun-Favreau H, Marx FP, Kautzmann S, Berg D, et al. Loss of function mutations in the gene encoding Omi/HtrA2 in Parkinson's disease. Hum Mol Genet. 2005;14(15):2099-111.

173. Bogaerts V, Nuytemans K, Reumers J, Pals P, Engelborghs S, Pickut B, et al. Genetic variability in the mitochondrial serine protease HTRA2 contributes to risk for Parkinson disease. Hum Mutat. 2008;29(6):832-40.

174. Lin CH, Chen ML, Chen GS, Tai CH, Wu RM. Novel variant Pro143Ala in HTRA2 contributes to Parkinson's disease by inducing hyperphosphorylation of HTRA2 protein in mitochondria. Hum Genet. 2011;130(6):817-27.

175. Wang CY, Xu Q, Weng L, Zhang Q, Zhang HN, Guo JF, et al. Genetic variations of Omi/HTRA2 in Chinese patients with Parkinson's disease. Brain Res. 2011;1385:293-7.

176. Miklossy J, Doudet DD, Schwab C, Yu S, McGeer EG, McGeer PL. Role of ICAM-1 in persisting inflammation in Parkinson disease and MPTP monkeys. Exp Neurol. 2006;197(2):275-83.

177. Andican G, Konukoglu D, Bozluolcay M, Bayulkem K, Firtiina S, Burcak G. Plasma oxidative and inflammatory markers in patients with idiopathic Parkinson's disease. Acta Neurol Belg. 2012;112(2):155-9.

178. Mount MP, Lira A, Grimes D, Smith PD, Faucher S, Slack R, et al. Involvement of interferon-gamma in microglial-mediated loss of dopaminergic neurons. J Neurosci. 2007;27(12):3328-37.

179. Brodacki B, Staszewski J, Toczylowska B, Kozlowska E, Drela N, Chalimoniuk M, et al. Serum interleukin (IL-2, IL-10, IL-6, IL-4), TNFalpha, and INFgamma concentrations are elevated in patients with atypical and idiopathic parkinsonism. Neurosci Lett. 2008;441(2):158-62.

180. Mutez E, Larvor L, Lepretre F, Mouroux V, Hamalek D, Kerckaert JP, et al. Transcriptional profile of Parkinson blood mononuclear cells with LRRK2 mutation. Neurobiol Aging. 2011;32(10):1839-48.

181. Rentzos M, Nikolaou C, Andreadou E, Paraskevas GP, Rombos A, Zoga M, et al. Circulating interleukin-10 and interleukin-12 in Parkinson's disease. Acta Neurol Scand. 2009;119(5):332-7.

182. Garcia-Esparcia P, Llorens F, Carmona M, Ferrer I. Complex deregulation and expression of cytokines and mediators of the immune response in Parkinson's disease brain is region dependent. Brain Pathol. 2014.

183. Mattila KM, Rinne JO, Lehtimaki T, Roytta M, Ahonen JP, Hurme M. Association of an interleukin 1B gene polymorphism (-511) with Parkinson's disease in Finnish patients. J Med Genet. 2002;39(6):400-2.

184. Blum-Degen D, Muller T, Kuhn W, Gerlach M, Przuntek H, Riederer P. Interleukin-1 beta and interleukin-6 are elevated in the cerebrospinal fluid of Alzheimer's and de novo Parkinson's disease patients. Neurosci Lett. 1995;202(1-2):17-20.

185. Mogi M, Harada M, Narabayashi H, Inagaki H, Minami M, Nagatsu T. Interleukin (IL)-1 beta, IL-2, IL-4, IL-6 and transforming growth factor-alpha levels are elevated in ventricular cerebrospinal fluid in juvenile parkinsonism and Parkinson's disease. Neurosci Lett. 1996;211(1):13-6.

186. Stypula G, Kunert-Radek J, Stepien H, Zylinska K, Pawlikowski M. Evaluation of interleukins, ACTH, cortisol and prolactin concentrations in the blood of patients with parkinson's disease. Neuroimmunomodulation. 1996;3(2-3):131-4.

187. San Luciano M, Ozelius L, Lipton RB, Raymond D, Bressman SB, Saunders-Pullman R. Gender differences in the IL6 -174G>C and ESR2 1730G>A polymorphisms and the risk of Parkinson's disease. Neurosci Lett. 2012;506(2):312-6.

188. Ross OA, O'Neill C, Rea IM, Lynch T, Gosal D, Wallace A, et al. Functional promoter region polymorphism of the proinflammatory chemokine IL-8 gene associates with Parkinson's disease in the Irish. Hum Immunol. 2004;65(4):340-6.

189. Wilhelm KR, Yanamandra K, Gruden MA, Zamotin V, Malisauskas M, Casaite V, et al. Immune reactivity towards insulin, its amyloid and protein S100B in blood sera of Parkinson's disease patients. Eur J Neurol. 2007;14(3):327-34.

190. Moroo I, Yamada T, Makino H, Tooyama I, McGeer PL, McGeer EG, et al. Loss of insulin receptor immunoreactivity from the substantia nigra pars compacta neurons in Parkinson's disease. Acta Neuropathol. 1994;87(4):343-8.

191. Takahashi M, Yamada T, Tooyama I, Moroo I, Kimura H, Yamamoto T, et al. Insulin receptor mRNA in the substantia nigra in Parkinson's disease. Neurosci Lett. 1996;204(3):201-4.

192. Wu G, Wang X, Feng X, Zhang A, Li J, Gu K, et al. Altered expression of autophagic genes in the peripheral leukocytes of patients with sporadic Parkinson's disease. Brain Res. 2011;1394:105-11.

193. Laguna A, Schintu N, Nobre A, Alvarsson A, Volakakis N, Jacobsen JK, et al. Dopaminergic control of autophagic-lysosomal function implicates Lmx1b in Parkinson's disease. Nat Neurosci. 2015;18(6):826-35.

194. Jiang XH, Yang H, Yang JF, Dong XM, Xu QY, Chen B. [Relationship between the Fnu4HI site polymorphism of monoamine oxidase A gene and Parkinson's disease]. Zhonghua Yi Xue Yi Chuan Xue Za Zhi. 2003;20(3):211-4.

195. Nakatome M, Tun Z, Shimada S, Honda K. Detection and analysis of four polymorphic markers at the human monoamine oxidase (MAO) gene in Japanese controls and patients with Parkinson's disease. Biochem Biophys Res Commun. 1998;247(2):452-6.

196. Hotamisligil GS, Girmen AS, Fink JS, Tivol E, Shalish C, Trofatter J, et al. Hereditary variations in monoamine oxidase as a risk factor for Parkinson's disease. Mov Disord. 1994;9(3):305-10.

197. Mellick GD, Buchanan DD, McCann SJ, James KM, Johnson AG, Davis DR, et al. Variations in the monoamine oxidase B (MAOB) gene are associated with Parkinson's disease. Mov Disord. 1999;14(2):219-24.

198. Kang SJ, Scott WK, Li YJ, Hauser MA, van der Walt JM, Fujiwara K, et al. Family-based case-control study of MAOA and MAOB polymorphisms in Parkinson disease. Mov Disord. 2006;21(12):2175-80.

199. Dil Kuazi A, Kito K, Abe Y, Shin RW, Kamitani T, Ueda N. NEDD8 protein is involved in ubiquitinated inclusion bodies. J Pathol. 2003;199(2):259-66.

200. Choo YS, Vogler G, Wang D, Kalvakuri S, Iliuk A, Tao WA, et al. Regulation of parkin and PINK1 by neddylation. Hum Mol Genet. 2012;21(11):2514-23.

201. Mogi M, Kondo T, Mizuno Y, Nagatsu T. p53 protein, interferon-gamma, and NF-kappaB levels are elevated in the parkinsonian brain. Neurosci Lett. 2007;414(1):94-7.

202. Levecque C, Elbaz A, Clavel J, Richard F, Vidal JS, Amouyel P, et al. Association between Parkinson's disease and polymorphisms in the nNOS and iNOS genes in a community-based case-control study. Hum Mol Genet. 2003;12(1):79-86.

203. Hancock DB, Martin ER, Vance JM, Scott WK. Nitric oxide synthase genes and their interactions with environmental factors in Parkinson's disease. Neurogenetics. 2008;9(4):249-62.

204. Xu PY, Liang R, Jankovic J, Hunter C, Zeng YX, Ashizawa T, et al. Association of homozygous 7048G7049 variant in the intron six of Nurr1 gene with Parkinson's disease. Neurology. 2002;58(6):881-4.

205. Wu Y, Peng R, Chen W, Zhang J, Li T, Wang Y, et al. [Association of the polymorphisms in NURR1 gene with Parkinson's disease]. Zhonghua Yi Xue Yi Chuan Xue Za Zhi. 2008;25(6):693-6.

206. Le WD, Xu P, Jankovic J, Jiang H, Appel SH, Smith RG, et al. Mutations in NR4A2 associated with familial Parkinson disease. Nat Genet. 2003;33(1):85-9.

207. Sleiman PM, Healy DG, Muqit MM, Yang YX, Van Der Brug M, Holton JL, et al. Characterisation of a novel NR4A2 mutation in Parkinson's disease brain. Neurosci Lett. 2009;457(2):75-9.

208. Le W, Pan T, Huang M, Xu P, Xie W, Zhu W, et al. Decreased NURR1 gene expression in patients with Parkinson's disease. J Neurol Sci. 2008;273(1-2):29-33.

209. Liu H, Wei L, Tao Q, Deng H, Ming M, Xu P, et al. Decreased NURR1 and PITX3 gene expression in Chinese patients with Parkinson's disease. Eur J Neurol. 2012;19(6):870-5.

210. Chu Y, Le W, Kompoliti K, Jankovic J, Mufson EJ, Kordower JH. Nurr1 in Parkinson's disease and related disorders. J Comp Neurol. 2006;494(3):495-514.

211. Bonifati V, Rizzu P, van Baren MJ, Schaap O, Breedveld GJ, Krieger E, et al. Mutations in the DJ-1 gene associated with autosomal recessive early-onset parkinsonism. Science. 2003;299(5604):256-9.

212. Macedo MG, Verbaan D, Fang Y, van Rooden SM, Visser M, Anar B, et al. Genotypic and phenotypic characteristics of Dutch patients with early onset Parkinson's disease. Mov Disord. 2009;24(2):196-203.

213. Valente EM, Abou-Sleiman PM, Caputo V, Muqit MM, Harvey K, Gispert S, et al. Hereditary early-onset Parkinson's disease caused by mutations in PINK1. Science. 2004;304(5674):1158-60.

214. Hatano Y, Li Y, Sato K, Asakawa S, Yamamura Y, Tomiyama H, et al. Novel PINK1 mutations in early-onset parkinsonism. Ann Neurol. 2004;56(3):424-7.

215. Li Y, Tomiyama H, Sato K, Hatano Y, Yoshino H, Atsumi M, et al. Clinicogenetic study of PINK1 mutations in autosomal recessive early-onset parkinsonism. Neurology. 2005;64(11):1955-7.

216. Ishihara-Paul L, Hulihan MM, Kachergus J, Upmanyu R, Warren L, Amouri R, et al. PINK1 mutations and parkinsonism. Neurology. 2008;71(12):896-902.

217. Kumazawa R, Tomiyama H, Li Y, Imamichi Y, Funayama M, Yoshino H, et al. Mutation analysis of the PINK1 gene in 391 patients with Parkinson disease. Arch Neurol. 2008;65(6):802-8.

218. Fuchs J, Mueller JC, Lichtner P, Schulte C, Munz M, Berg D, et al. The transcription factor PITX3 is associated with sporadic Parkinson's disease. Neurobiol Aging. 2009;30(5):731-8.

219. Tang L, Zhao S, Wang M, Sheth A, Zhao Z, Chen L, et al. Meta-analysis of association between PITX3 gene polymorphism and Parkinson's disease. J Neurol Sci. 2012;317(1-2):80-6.

220. Qiu G, Fu C, Liang GH. Association between PITX3 promoter polymorphism and risk of Parkinson's disease: the impact of ethnicity and onset age. Neurosci Lett. 2014;561:128-33.

221. Paisan-Ruiz C, Bhatia KP, Li A, Hernandez D, Davis M, Wood NW, et al. Characterization of PLA2G6 as a locus for dystonia-parkinsonism. Ann Neurol. 2009;65(1):19-23.

222. Kauther KM, Hoft C, Rissling I, Oertel WH, Moller JC. The PLA2G6 gene in early-onset Parkinson's disease. Mov Disord. 2011;26(13):2415-7.

223. Gui YX, Xu ZP, Wen L, Liu HM, Zhao JJ, Hu XY. Four novel rare mutations of PLA2G6 in Chinese population with Parkinson's disease. Parkinsonism Relat Disord. 2013;19(1):21-6.

224. Wang ES, Sun Y, Guo JG, Gao X, Hu JW, Zhou L, et al. Tetranectin and apolipoprotein A-I in cerebrospinal fluid as potential biomarkers for Parkinson's disease. Acta Neurol Scand. 2010;122(5):350-9.

225. Hunot S, Brugg B, Ricard D, Michel PP, Muriel MP, Ruberg M, et al. Nuclear translocation of NF-kappaB is increased in dopaminergic neurons of patients with parkinson disease. Proc Natl Acad Sci U S A. 1997;94(14):7531-6.

226. Zhang A, Wang H, Qin X, Pang S, Yan B. Genetic analysis of SIRT1 gene promoter in sporadic Parkinson's disease. Biochem Biophys Res Commun. 2012;422(4):693-6.

227. Searles Nielsen S, Bammler TK, Gallagher LG, Farin FM, Longstreth W, Jr., Franklin GM, et al. Genotype and age at Parkinson disease diagnosis. Int J Mol Epidemiol Genet. 2013;4(1):61-9.

228. Salazar J, Mena N, Hunot S, Prigent A, Alvarez-Fischer D, Arredondo M, et al. Divalent metal transporter 1 (DMT1) contributes to neurodegeneration in animal models of Parkinson's disease. Proc Natl Acad Sci U S A. 2008;105(47):18578-83.

229. Glatt CE, Wahner AD, White DJ, Ruiz-Linares A, Ritz B. Gain-of-function haplotypes in the vesicular monoamine transporter promoter are protective for Parkinson disease in women. Hum Mol Genet. 2006;15(2):299-305.

230. Brighina L, Riva C, Bertola F, Saracchi E, Fermi S, Goldwurm S, et al. Analysis of vesicular monoamine transporter 2 polymorphisms in Parkinson's disease. Neurobiol Aging. 2013;34(6):1712 e9-13.

231. Visanji NP, Collingwood JF, Finnegan ME, Tandon A, House E, Hazrati LN. Iron deficiency in parkinsonism: region-specific iron dysregulation in Parkinson's disease and multiple system atrophy. J Parkinsons Dis. 2013;3(4):523-37.

232. Wang J, Liu Z, Chen B. [Association between genetic polymorphism of dopamine transporter gene and susceptibility to Parkinson's disease]. Zhonghua Yi Xue Za Zhi. 2000;80(5):346-8.

233. Kim JW, Kim DH, Kim SH, Cha JK. Association of the dopamine transporter gene with Parkinson's disease in Korean patients. J Korean Med Sci. 2000;15(4):449-51.

234. Zhai D, Li S, Zhao Y, Lin Z. SLC6A3 is a risk factor for Parkinson's disease: a meta-analysis of sixteen years' studies. Neurosci Lett. 2014;564:99-104.

235. Foo JN, Liany H, Bei JX, Yu XQ, Liu J, Au WL, et al. Rare lysosomal enzyme gene SMPD1 variant (p.R591C) associates with Parkinson's disease. Neurobiol Aging. 2013;34(12):2890 e13-5.

236. Gan-Or Z, Ozelius LJ, Bar-Shira A, Saunders-Pullman R, Mirelman A, Kornreich R, et al. The p.L302P mutation in the lysosomal enzyme gene SMPD1 is a risk factor for Parkinson disease. Neurology. 2013;80(17):1606-10.

237. Poirier J, Dea D, Baccichet A, Thiffault C. Superoxide dismutase expression in Parkinson's disease. Ann N Y Acad Sci. 1994;738:116-20.

238. Yoritaka A, Hattori N, Mori H, Kato K, Mizuno Y. An immunohistochemical study on manganese superoxide dismutase in Parkinson's disease. J Neurol Sci. 1997;148(2):181-6.

239. Belluzzi E, Bisaglia M, Lazzarini E, Tabares LC, Beltramini M, Bubacco L. Human SOD2 modification by dopamine quinones affects enzymatic activity by promoting its aggregation: possible implications for Parkinson's disease. PLoS One. 2012;7(6):e38026.

240. Quadri M, Fang M, Picillo M, Olgiati S, Breedveld GJ, Graafland J, et al. Mutation in the SYNJ1 gene associated with autosomal recessive, early-onset Parkinsonism. Hum Mutat. 2013;34(9):1208-15.

241. Krebs CE, Karkheiran S, Powell JC, Cao M, Makarov V, Darvish H, et al. The Sac1 domain of SYNJ1 identified mutated in a family with early-onset progressive Parkinsonism with generalized seizures. Hum Mutat. 2013;34(9):1200-7.

242. A two-stage meta-analysis identifies several new loci for Parkinson's disease. PLoS Genet. 2011;7(6):e1002142.

243. Rhodes SL, Buchanan DD, Ahmed I, Taylor KD, Loriot MA, Sinsheimer JS, et al. Pooled analysis of iron-related genes in Parkinson's disease: association with transferrin. Neurobiol Dis. 2014;62:172-8.

244. Mastroberardino PG, Hoffman EK, Horowitz MP, Betarbet R, Taylor G, Cheng D, et al. A novel transferrin/TfR2-mediated mitochondrial iron transport system is disrupted in Parkinson's disease. Neurobiol Dis. 2009;34(3):417-31.

245. Faucheux BA, Hauw JJ, Agid Y, Hirsch EC. The density of [125I]-transferrin binding sites on perikarya of melanized neurons of the substantia nigra is decreased in Parkinson's disease. Brain Res. 1997;749(1):170-4.

246. Kastner A, Hirsch EC, Agid Y, Javoy-Agid F. Tyrosine hydroxylase protein and messenger RNA in the dopaminergic nigral neurons of patients with Parkinson's disease. Brain Res. 1993;606(2):341-5.

247. Nishimura M, Mizuta I, Mizuta E, Yamasaki S, Ohta M, Kaji R, et al. Tumor necrosis factor gene polymorphisms in patients with sporadic Parkinson's disease. Neurosci Lett. 2001;311(1):1-4.

248. Wu YR, Feng IH, Lyu RK, Chang KH, Lin YY, Chan H, et al. Tumor necrosis factor-alpha promoter polymorphism is associated with the risk of Parkinson's disease. Am J Med Genet B Neuropsychiatr Genet. 2007;144B(3):300-4.

249. Bialecka M, Klodowska-Duda G, Kurzawski M, Slawek J, Gorzkowska A, Opala G, et al. Interleukin-10 (IL10) and tumor necrosis factor alpha (TNF) gene polymorphisms in Parkinson's disease patients. Parkinsonism Relat Disord. 2008;14(8):636-40.

250. Mogi M, Harada M, Riederer P, Narabayashi H, Fujita K, Nagatsu T. Tumor necrosis factor-alpha (TNF-alpha) increases both in the brain and in the cerebrospinal fluid from parkinsonian patients. Neurosci Lett. 1994;165(1-2):208-10.

251. Nair VD, McNaught KS, Gonzalez-Maeso J, Sealfon SC, Olanow CW. p53 mediates nontranscriptional cell death in dopaminergic cells in response to proteasome inhibition. J Biol Chem. 2006;281(51):39550-60.

252. Politis M, Su P, Piccini P. Imaging of microglia in patients with neurodegenerative disorders. Front Pharmacol. 2012;3:96.

253. Wintermeyer P, Kruger R, Kuhn W, Muller T, Woitalla D, Berg D, et al. Mutation analysis and association studies of the UCHL1 gene in German Parkinson's disease patients. Neuroreport. 2000;11(10):2079-82.

254. Toda T, Momose Y, Murata M, Tamiya G, Yamamoto M, Hattori N, et al. Toward identification of susceptibility genes for sporadic Parkinson's disease. J Neurol. 2003;250 Suppl 3:III40-3.

255. Hauser MA, Li YJ, Xu H, Noureddine MA, Shao YS, Gullans SR, et al. Expression profiling of substantia nigra in Parkinson disease, progressive supranuclear palsy, and frontotemporal dementia with parkinsonism. Arch Neurol. 2005;62(6):917-21.

256. Chu Y, Goldman JG, Kelly L, He Y, Waliczek T, Kordower JH. Abnormal alpha-synuclein reduces nigral voltage-dependent anion channel 1 in sporadic and experimental Parkinson's disease. Neurobiol Dis. 2014;69:1-14.

257. Han X, Xue L, Li Y, Chen B, Xie A. Vitamin D receptor gene polymorphism and its association with Parkinson's disease in Chinese Han population. Neurosci Lett. 2012;525(1):29-33.

258. Torok R, Torok N, Szalardy L, Plangar I, Szolnoki Z, Somogyvari F, et al. Association of vitamin D receptor gene polymorphisms and Parkinson's disease in Hungarians. Neurosci Lett. 2013;551:70-4.

259. Butler MW, Burt A, Edwards TL, Zuchner S, Scott WK, Martin ER, et al. Vitamin D receptor gene as a candidate gene for Parkinson disease. Ann Hum Genet. 2011;75(2):201-10.

260. Vilarino-Guell C, Wider C, Ross OA, Dachsel JC, Kachergus JM, Lincoln SJ, et al. VPS35 mutations in Parkinson disease. Am J Hum Genet. 2011;89(1):162-7.

261. Zimprich A, Benet-Pages A, Struhal W, Graf E, Eck SH, Offman MN, et al. A mutation in VPS35, encoding a subunit of the retromer complex, causes late-onset Parkinson disease. Am J Hum Genet. 2011;89(1):168-75.

262. Hamza TH, Chen H, Hill-Burns EM, Rhodes SL, Montimurro J, Kay DM, et al. Genome-wide gene-environment study identifies glutamate receptor gene GRIN2A as a Parkinson's disease modifier gene via interaction with coffee. PLoS Genet. 2011;7(8):e1002237.

263. Voss M, Schroder B, Fluhrer R. Mechanism, specificity, and physiology of signal peptide peptidase (SPP) and SPP-like proteases. Biochim Biophys Acta. 2013;1828(12):2828-39.

264. Golde TE, Wolfe MS, Greenbaum DC. Signal peptide peptidases: a family of intramembrane-cleaving proteases that cleave type 2 transmembrane proteins. Semin Cell Dev Biol. 2009;20(2):225-30.

265. Sulzer D, Surmeier DJ. Neuronal vulnerability, pathogenesis, and Parkinson's disease. Mov Disord. 2013;28(6):715-24.

266. Munoz P, Huenchuguala S, Paris I, Segura-Aguilar J. Dopamine oxidation and autophagy. Parkinsons Dis. 2012;2012:920953.

267. Vinish M, Anand A, Prabhakar S. Altered oxidative stress levels in Indian Parkinson's disease patients with PARK2 mutations. Acta Biochim Pol. 2011;58(2):165-9.

268. Sharma A, Kaur P, Kumar B, Prabhakar S, Gill KD. Plasma lipid peroxidation and antioxidant status of Parkinson's disease patients in the Indian population. Parkinsonism Relat Disord. 2008;14(1):52-7.

269. Sanyal J, Bandyopadhyay Sk Fau - Banerjee TK, Banerjee Tk Fau - Mukherjee SC, Mukherjee Sc Fau - Chakraborty DP, Chakraborty Dp Fau - Ray BC, Ray Bc Fau - Rao VR, et al. Plasma levels of lipid peroxides in patients with Parkinson's disease. Eur Rev Med Pharmacol Sci. 2009;13(2):129-32.

270. Yoritaka A, Hattori N, Uchida K, Tanaka M, Stadtman ER, Mizuno Y. Immunohistochemical detection of 4-hydroxynonenal protein adducts in Parkinson disease. Proc Natl Acad Sci U S A. 1996;93(7):2696-701.

271. Selley ML. (E)-4-hydroxy-2-nonenal may be involved in the pathogenesis of Parkinson's. Free Radic Biol Med. 1998;25(2):169-74.

272. Shih JC, Thompson RF. Monoamine oxidase in neuropsychiatry and behavior. Am J Hum Genet. 1999;65(3):593-8 FAU - Shih, J C.

273. Ishikawa S, Tanaka Y, Takahashi-Niki K, Niki T, Ariga H, Iguchi-Ariga SM. Stimulation of vesicular monoamine transporter 2 activity by DJ-1 in SH-SY5Y cells. Biochem Biophys Res Commun. 2012;421(4):813-8.

274. Liang CL, Nelson O, Yazdani U, Pasbakhsh P, German DC. Inverse relationship between the contents of neuromelanin pigment and the vesicular monoamine transporter-2: human midbrain dopamine neurons. J Comp Neurol. 2004;473(1):97-106.

275. Caudle WM, Richardson JR, Wang MZ, Taylor TN, Guillot TS, McCormack AL, et al. Reduced vesicular storage of dopamine causes progressive nigrostriatal neurodegeneration. J Neurosci. 2007;27(30):8138-48.

276. Jiang H, Jiang Q, Liu W, Feng J. Parkin suppresses the expression of monoamine oxidases. J Biol Chem. 2006;281(13):8591-9.

277. Ou XM, Chen K, Shih JC. Glucocorticoid and androgen activation of monoamine oxidase A is regulated differently by R1 and Sp1. J Biol Chem. 2006;281(30):21512-25.

278. Libert S, Pointer K, Bell EL, Das A, Cohen DE, Asara JM, et al. SIRT1 activates MAO-A in the brain to mediate anxiety and exploratory drive. Cell. 2011;147(7):1459-72.

279. Cao X, Wei Z, Gabriel GG, Li X, Mousseau DD. Calcium-sensitive regulation of monoamine oxidase-A contributes to the production of peroxyradicals in hippocampal cultures: implications for Alzheimer disease-related pathology. BMC Neurosci. 2007;8:73.

280. Sharan C, Halder SK, Thota C, Jaleel T, Nair S, Al-Hendy A. Vitamin D inhibits proliferation of human uterine leiomyoma cells via catechol-O-methyltransferase. Fertil Steril. 2011;95(1):247-53.

281. Shalamanova L, McArdle F, Amara AB, Jackson MJ, Rustom R. Albumin overload induces adaptive responses in human proximal tubular cells through oxidative stress but not via angiotensin II type 1 receptor. Am J Physiol Renal Physiol. 2007;292(6):F1846-57.

282. Wang L, Wu Q, Qiu P, Mirza A, McGuirk M, Kirschmeier P, et al. Analyses of p53 target genes in the human genome by bioinformatic and microarray approaches. J Biol Chem. 2001;276(47):43604-10.

283. Zafar KS, Siegel D, Ross D. A potential role for cyclized quinones derived from dopamine, DOPA, and 3,4-dihydroxyphenylacetic acid in proteasomal inhibition. Mol Pharmacol. 2006;70(3):1079-86.

284. Norris EH, Giasson BI, Hodara R, Xu S, Trojanowski JQ, Ischiropoulos H, et al. Reversible inhibition of alpha-synuclein fibrillization by dopaminochrome-mediated conformational alterations. J Biol Chem. 2005;280(22):21212-9.

285. Van Laar VS, Mishizen AJ, Cascio M, Hastings TG. Proteomic identification of dopamine-conjugated proteins from isolated rat brain mitochondria and SH-SY5Y cells. Neurobiol Dis. 2009;34(3):487-500.

286. LaVoie MJ, Ostaszewski BL, Weihofen A, Schlossmacher MG, Selkoe DJ. Dopamine covalently modifies and functionally inactivates parkin. Nat Med. 2005;11(11):1214-21.

287. McCoy MK, Cookson MR. DJ-1 regulation of mitochondrial function and autophagy through oxidative stress. Autophagy. 2011;7(5):531-2.

288. Kabuta T, Furuta A, Aoki S, Furuta K, Wada K. Aberrant interaction between Parkinson disease-associated mutant UCH-L1 and the lysosomal receptor for chaperone-mediated autophagy. J Biol Chem. 2008;283(35):23731-8.

289. Nagatsu T, Levitt M, Udenfriend S. TYROSINE HYDROXYLASE. THE INITIAL STEP IN NOREPINEPHRINE BIOSYNTHESIS. J Biol Chem. 1964;239:2910-7.

290. Lu L, Sun X, Liu Y, Zhao H, Zhao S, Yang H. DJ-1 upregulates tyrosine hydroxylase gene expression by activating its transcriptional factor Nurr1 via the ERK1/2 pathway. Int J Biochem Cell Biol. 2012;44(1):65-71.

291. Dermitzaki E, Tsatsanis C, Minas V, Chatzaki E, Charalampopoulos I, Venihaki M, et al. Corticotropin-releasing factor (CRF) and the urocortins differentially regulate catecholamine secretion in human and rat adrenals, in a CRF receptor type-specific manner. Endocrinology. 2007;148(4):1524-38.

292. Cellerino A, Pinzon-Duarte G, Carroll P, Kohler K. Brain-derived neurotrophic factor modulates the development of the dopaminergic network in the rodent retina. J Neurosci. 1998;18(9):3351-62.

293. Jones SR, Gainetdinov RR, Jaber M, Giros B, Wightman RM, Caron MG. Profound neuronal plasticity in response to inactivation of the dopamine transporter. Proc Natl Acad Sci U S A. 1998;95(7):4029-34.

294. Lebel M, Gauthier Y, Moreau A, Drouin J. Pitx3 activates mouse tyrosine hydroxylase promoter via a high-affinity binding site. J Neurochem. 2001;77(2):558-67.

295. Masliah E, Rockenstein E, Veinbergs I, Mallory M, Hashimoto M, Takeda A, et al. Dopaminergic loss and inclusion body formation in alpha-synuclein mice: implications for neurodegenerative disorders. Science. 2000;287(5456):1265-9.

296. Alerte TN, Akinfolarin AA, Friedrich EE, Mader SA, Hong CS, Perez RG. Alpha-synuclein aggregation alters tyrosine hydroxylase phosphorylation and immunoreactivity: lessons from viral transduction of knockout mice. Neurosci Lett. 2008;435(1):24-9.

297. Domanskyi A, Geissler C, Vinnikov IA, Alter H, Schober A, Vogt MA, et al. Pten ablation in adult dopaminergic neurons is neuroprotective in Parkinson's disease models. FASEB J. 2011;25(9):2898-910.

298. Zauli G, Secchiero P, Rodella L, Gibellini D, Mirandola P, Mazzoni M, et al. HIV-1 Tat-mediated inhibition of the tyrosine hydroxylase gene expression in dopaminergic neuronal cells. J Biol Chem. 2000;275(6):4159-65.

299. Lindgren N, Usiello A, Goiny M, Haycock J, Erbs E, Greengard P, et al. Distinct roles of dopamine D2L and D2S receptor isoforms in the regulation of protein phosphorylation at presynaptic and postsynaptic sites. Proc Natl Acad Sci U S A. 2003;100(7):4305-9.

300. Perez RG, Waymire JC, Lin E, Liu JJ, Guo F, Zigmond MJ. A role for alpha-synuclein in the regulation of dopamine biosynthesis. J Neurosci. 2002;22(8):3090-9.

301. Lin Z, Canales JJ, Bjorgvinsson T, Thomsen M, Qu H, Liu QR, et al. Monoamine transporters: vulnerable and vital doorkeepers. Prog Mol Biol Transl Sci. 2011;98:1-46.

302. Egana LA, Cuevas RA, Baust TB, Parra LA, Leak RK, Hochendoner S, et al. Physical and functional interaction between the dopamine transporter and the synaptic vesicle protein synaptogyrin-3. J Neurosci. 2009;29(14):4592-604.

303. Lee FJ, Liu F, Pristupa ZB, Niznik HB. Direct binding and functional coupling of alpha-synuclein to the dopamine transporters accelerate dopamine-induced apoptosis. FASEB J. 2001;15(6):916-26.

304. Jiang H, Jiang Q, Feng J. Parkin increases dopamine uptake by enhancing the cell surface expression of dopamine transporter. J Biol Chem. 2004;279(52):54380-6.

305. Proudfoot NJ, Shander MH, Manley JL, Gefter ML, Maniatis T. Structure and in vitro transcription of human globin genes. Science. 1980;209(4463):1329-36.

306. Watanabe J, Grijalva V, Hama S, Barbour K, Berger FG, Navab M, et al. Hemoglobin and its scavenger protein haptoglobin associate with apoA-1-containing particles and influence the inflammatory properties and function of high density lipoprotein. J Biol Chem. 2009;284(27):18292-301.

307. Spagnuolo MS, Di Stasi R, De Rosa L, Maresca B, Cigliano L, D'Andrea LD. Analysis of the haptoglobin binding region on the apolipoprotein A-I-derived P2a peptide. J Pept Sci. 2013;19(4):220-6.

308. Buehler PW, Abraham B, Vallelian F, Linnemayr C, Pereira CP, Cipollo JF, et al. Haptoglobin preserves the CD163 hemoglobin scavenger pathway by shielding hemoglobin from peroxidative modification. Blood. 2009;113(11):2578-86.

309. Biagioli M, Pinto M, Cesselli D, Zaninello M, Lazarevic D, Roncaglia P, et al. Unexpected expression of alpha- and beta-globin in mesencephalic dopaminergic neurons and glial cells. Proc Natl Acad Sci U S A. 2009;106(36):15454-9.

310. Larsen R, Gouveia Z, Soares MP, Gozzelino R. Heme cytotoxicity and the pathogenesis of immune-mediated inflammatory diseases. Front Pharmacol. 2012;3:77.

311. Ris-Stalpers C, Bikker H. Genetics and phenomics of hypothyroidism and goiter due to TPO mutations. Mol Cell Endocrinol. 2010;322(1-2):38-43.

312. UniProt: a hub for protein information. Nucleic Acids Res. 2015;43(Database issue):D204-12.

313. Morishima Y, Lau M, Peng HM, Miyata Y, Gestwicki JE, Pratt WB, et al. Heme-dependent activation of neuronal nitric oxide synthase by cytosol is due to an Hsp70-dependent, thioredoxin-mediated thiol-disulfide interchange in the heme/substrate binding cleft. Biochemistry. 2011;50(33):7146-56.

314. Larsson O, Li S, Issaenko OA, Avdulov S, Peterson M, Smith K, et al. Eukaryotic translation initiation factor 4E induced progression of primary human mammary epithelial cells along the cancer pathway is associated with targeted translational deregulation of oncogenic drivers and inhibitors. Cancer Res. 2007;67(14):6814-24.

315. Siow RC, Ishii T, Sato H, Taketani S, Leake DS, Sweiry JH, et al. Induction of the antioxidant stress proteins heme oxygenase-1 and MSP23 by stress agents and oxidised LDL in cultured vascular smooth muscle cells. FEBS Lett. 1995;368(2):239-42.

316. Konvalinka A, Zhou J, Dimitromanolakis A, Drabovich AP, Fang F, Gurley S, et al. Determination of an angiotensin II-regulated proteome in primary human kidney cells by stable isotope labeling of amino acids in cell culture (SILAC). J Biol Chem. 2013;288(34):24834-47.

317. Lee TS, Chau LY. Heme oxygenase-1 mediates the anti-inflammatory effect of interleukin-10 in mice. Nat Med. 2002;8(3):240-6.

318. Chowdhry S, Zhang Y, McMahon M, Sutherland C, Cuadrado A, Hayes JD. Nrf2 is controlled by two distinct beta-TrCP recognition motifs in its Neh6 domain, one of which can be modulated by GSK-3 activity. Oncogene. 2013;32(32):3765-81.

319. Salinas M, Diaz R, Abraham NG, Ruiz de Galarreta CM, Cuadrado A. Nerve growth factor protects against 6-hydroxydopamine-induced oxidative stress by increasing expression of heme oxygenase-1 in a phosphatidylinositol 3-kinase-dependent manner. J Biol Chem. 2003;278(16):13898-904.

320. Liu X, Cui Y, Li M, Xu H, Zuo J, Fang F, et al. Cobalt protoporphyrin induces HO-1 expression mediated partially by FOXO1 and reduces mitochondria-derived reactive oxygen species production. PLoS One. 2013;8(11):e80521.

321. Cuadrado A, Martin-Moldes Z, Ye J, Lastres-Becker I. Transcription factors NRF2 and NF-kappaB are coordinated effectors of the Rho family, GTP-binding protein RAC1 during inflammation. J Biol Chem. 2014;289(22):15244-58.

322. Kallin A, Johannessen LE, Cani PD, Marbehant CY, Essaghir A, Foufelle F, et al. SREBP-1 regulates the expression of heme oxygenase 1 and the phosphatidylinositol-3 kinase regulatory subunit p55 gamma. J Lipid Res. 2007;48(7):1628-36.

323. He Q, Song N, Jia F, Xu H, Yu X, Xie J, et al. Role of alpha-synuclein aggregation and the nuclear factor E2-related factor 2/heme oxygenase-1 pathway in iron-induced neurotoxicity. Int J Biochem Cell Biol. 2013;45(6):1019-30.

324. Rushworth SA, Bowles KM, Raninga P, MacEwan DJ. NF-kappaB-inhibited acute myeloid leukemia cells are rescued from apoptosis by heme oxygenase-1 induction. Cancer Res. 2010;70(7):2973-83.

325. Choe YJ, Lee SY, Ko KW, Shin SJ, Kim HS. Nutlin-3 induces HO-1 expression by activating JNK in a transcription-independent manner of p53. Int J Oncol. 2014;44(3):761-8.

326. Liu XM, Peyton KJ, Ensenat D, Wang H, Schafer AI, Alam J, et al. Endoplasmic reticulum stress stimulates heme oxygenase-1 gene expression in vascular smooth muscle. Role in cell survival. J Biol Chem. 2005;280(2):872-7.

327. Ryter SW, Si M, Lai CC, Su CY. Regulation of endothelial heme oxygenase activity during hypoxia is dependent on chelatable iron. Am J Physiol Heart Circ Physiol. 2000;279(6):H2889-97.

328. Freidja ML, Toutain B, Caillon A, Desquiret V, Lambert D, Loufrani L, et al. Heme oxygenase 1 is differentially involved in blood flow-dependent arterial remodeling: role of inflammation, oxidative stress, and nitric oxide. Hypertension. 2011;58(2):225-31.

329. Schmidt J, Mertz K, Morgan JI. Regulation of heme oxygenase-1 expression by dopamine in cultured C6 glioma and primary astrocytes. Brain Res Mol Brain Res. 1999;73(1-2):50-9.

330. Chandra-Kuntal K, Lee J, Singh SV. Critical role for reactive oxygen species in apoptosis induction and cell migration inhibition by diallyl trisulfide, a cancer chemopreventive component of garlic. Breast Cancer Res Treat. 2013;138(1):69-79.

331. Hung SY, Liou HC, Fu WM. The mechanism of heme oxygenase-1 action involved in the enhancement of neurotrophic factor expression. Neuropharmacology. 2010;58(2):321-9.

332. Song W, Zukor H, Lin SH, Hascalovici J, Liberman A, Tavitian A, et al. Schizophrenia-like features in transgenic mice overexpressing human HO-1 in the astrocytic compartment. J Neurosci. 2012;32(32):10841-53.

333. Liu Z, Wang J, Huang E, Gao S, Li H, Lu J, et al. Tanshinone IIA suppresses cholesterol accumulation in human macrophages: role of heme oxygenase-1. J Lipid Res. 2014;55(2):201-13.

334. Gueron G, De Siervi A, Ferrando M, Salierno M, De Luca P, Elguero B, et al. Critical role of endogenous heme oxygenase 1 as a tuner of the invasive potential of prostate cancer cells. Mol Cancer Res. 2009;7(11):1745-55.

335. Li FY, Lam KS, Tse HF, Chen C, Wang Y, Vanhoutte PM, et al. Endothelium-selective activation of AMP-activated protein kinase prevents diabetes mellitus-induced impairment in vascular function and reendothelialization via induction of heme oxygenase-1 in mice. Circulation. 2012;126(10):1267-77.

336. Wagener FA, da Silva JL, Farley T, de Witte T, Kappas A, Abraham NG. Differential effects of heme oxygenase isoforms on heme mediation of endothelial intracellular adhesion molecule 1 expression. J Pharmacol Exp Ther. 1999;291(1):416-23.

337. Seldon MP, Silva G, Pejanovic N, Larsen R, Gregoire IP, Filipe J, et al. Heme oxygenase-1 inhibits the expression of adhesion molecules associated with endothelial cell activation via inhibition of NF-kappaB RelA phosphorylation at serine 276. J Immunol. 2007;179(11):7840-51.

338. Double KL, Gerlach M, Schunemann V, Trautwein AX, Zecca L, Gallorini M, et al. Iron-binding characteristics of neuromelanin of the human substantia nigra. Biochem Pharmacol. 2003;66(3):489-94.

339. Faucheux BA, Martin ME, Beaumont C, Hauw JJ, Agid Y, Hirsch EC. Neuromelanin associated redox-active iron is increased in the substantia nigra of patients with Parkinson's disease. J Neurochem. 2003;86(5):1142-8.

340. Oka K, Kato T, Takita T, Takeuchi T, Umezawa H, Nagatsu T. Inhibition of tyrosine hydroxylase, a Fe(II)-stimulated monooxygenase, by bleomycin. J Antibiot (Tokyo). 1980;33(9):1043-7.

341. Pichler I, Del Greco MF, Gogele M, Lill CM, Bertram L, Do CB, et al. Serum iron levels and the risk of Parkinson disease: a Mendelian randomization study. PLoS Med. 2013;10(6):e1001462.

342. Wolff NA, Ghio AJ, Garrick LM, Garrick MD, Zhao L, Fenton RA, et al. Evidence for mitochondrial localization of divalent metal transporter 1 (DMT1). FASEB J. 2014;28(5):2134-45.

343. Roth JA, Singleton S, Feng J, Garrick M, Paradkar PN. Parkin regulates metal transport via proteasomal degradation of the 1B isoforms of divalent metal transporter 1. J Neurochem. 2010;113(2):454-64.

344. Kunitake ST, Jarvis MR, Hamilton RL, Kane JP. Binding of transition metals by apolipoprotein A-I-containing plasma lipoproteins: inhibition of oxidation of low density lipoproteins. Proc Natl Acad Sci U S A. 1992;89(15):6993-7.

345. McPherson PA, Young IS, McKibben B, McEneny J. High density lipoprotein subfractions: isolation, composition, and their duplicitous role in oxidation. J Lipid Res. 2007;48(1):86-95.

346. Abdizadeh H, Atilgan AR, Atilgan C. Detailed molecular dynamics simulations of human transferrin provide insights into iron release dynamics at serum and endosomal pH. Journal of biological inorganic chemistry : JBIC : a publication of the Society of Biological Inorganic Chemistry. 2015;20(4):705-18.

347. Powers KM, Smith-Weller T, Franklin GM, Longstreth WT, Jr., Swanson PD, Checkoway H. Dietary fats, cholesterol and iron as risk factors for Parkinson's disease. Parkinsonism Relat Disord. 2009;15(1):47-52.

348. Tsukamoto T, Kitano Y, Kuno S. Blood pressure fluctuation and hypertension in patients with Parkinson's disease. Brain Behav. 2013;3(6):710-4.

349. Oh YS, Kim JS, Yang DW, Koo JS, Kim YI, Jung HO, et al. Nighttime blood pressure and white matter hyperintensities in patients with Parkinson disease. Chronobiol Int. 2013;30(6):811-7.

350. Miyake Y, Tanaka K, Fukushima W, Sasaki S, Kiyohara C, Tsuboi Y, et al. Case-control study of risk of Parkinson's disease in relation to hypertension, hypercholesterolemia, and diabetes in Japan. J Neurol Sci. 2010;293(1-2):82-6.

351. Labandeira-Garcia JL, Rodriguez-Pallares J, Villar-Cheda B, Rodriguez-Perez AI, Garrido-Gil P, Guerra MJ. Aging, Angiotensin system and dopaminergic degeneration in the substantia nigra. Aging Dis. 2011;2(3):257-74.

352. Gildea JJ. Dopamine and angiotensin as renal counterregulatory systems controlling sodium balance. Curr Opin Nephrol Hypertens. 2009;18(1):28-32.

353. Villar-Cheda B, Rodriguez-Pallares J, Valenzuela R, Munoz A, Guerra MJ, Baltatu OC, et al. Nigral and striatal regulation of angiotensin receptor expression by dopamine and angiotensin in rodents: implications for progression of Parkinson's disease. Eur J Neurosci. 2010;32(10):1695-706.

354. Ruiz-Ortega M, Lorenzo O, Suzuki Y, Ruperez M, Egido J. Proinflammatory actions of angiotensins. Curr Opin Nephrol Hypertens. 2001;10(3):321-9.

355. Suzuki Y, Ruiz-Ortega M, Lorenzo O, Ruperez M, Esteban V, Egido J. Inflammation and angiotensin II. Int J Biochem Cell Biol. 2003;35(6):881-900.

356. Berk BC, Corson MA. Angiotensin II signal transduction in vascular smooth muscle: role of tyrosine kinases. Circ Res. 1997;80(5):607-16.

357. Ali MS, Sayeski PP, Dirksen LB, Hayzer DJ, Marrero MB, Bernstein KE. Dependence on the motif YIPP for the physical association of Jak2 kinase with the intracellular carboxyl tail of the angiotensin II AT1 receptor. J Biol Chem. 1997;272(37):23382-8.

358. Pan J, Fukuda K, Kodama H, Makino S, Takahashi T, Sano M, et al. Role of angiotensin II in activation of the JAK/STAT pathway induced by acute pressure overload in the rat heart. Circ Res. 1997;81(4):611-7.

359. Li X, Rayford H, Uhal BD. Essential roles for angiotensin receptor AT1a in bleomycin-induced apoptosis and lung fibrosis in mice. Am J Pathol. 2003;163(6):2523-30.

360. Suzuki Y, Lopez-Franco O, Gomez-Garre D, Tejera N, Gomez-Guerrero C, Sugaya T, et al. Renal tubulointerstitial damage caused by persistent proteinuria is attenuated in AT1-deficient mice: role of endothelin-1. Am J Pathol. 2001;159(5):1895-904.

361. Lemarie CA, Simeone SM, Nikonova A, Ebrahimian T, Deschenes ME, Coffman TM, et al. Aldosterone-induced activation of signaling pathways requires activity of angiotensin type 1a receptors. Circ Res. 2009;105(9):852-9.

362. Luttrell LM, Roudabush FL, Choy EW, Miller WE, Field ME, Pierce KL, et al. Activation and targeting of extracellular signal-regulated kinases by beta-arrestin scaffolds. Proc Natl Acad Sci U S A. 2001;98(5):2449-54.
[truncated: 360,505 more chars]
