# Supplementary figures and images for: Integrated molecular landscape of Parkinson’s disease
Source: NPJ Parkinsons Dis. 2017 Apr 10;3:14. doi: 10.1038/s41531-017-0015-3 (PMC5460267; doi:10.1038/s41531-017-0015-3)

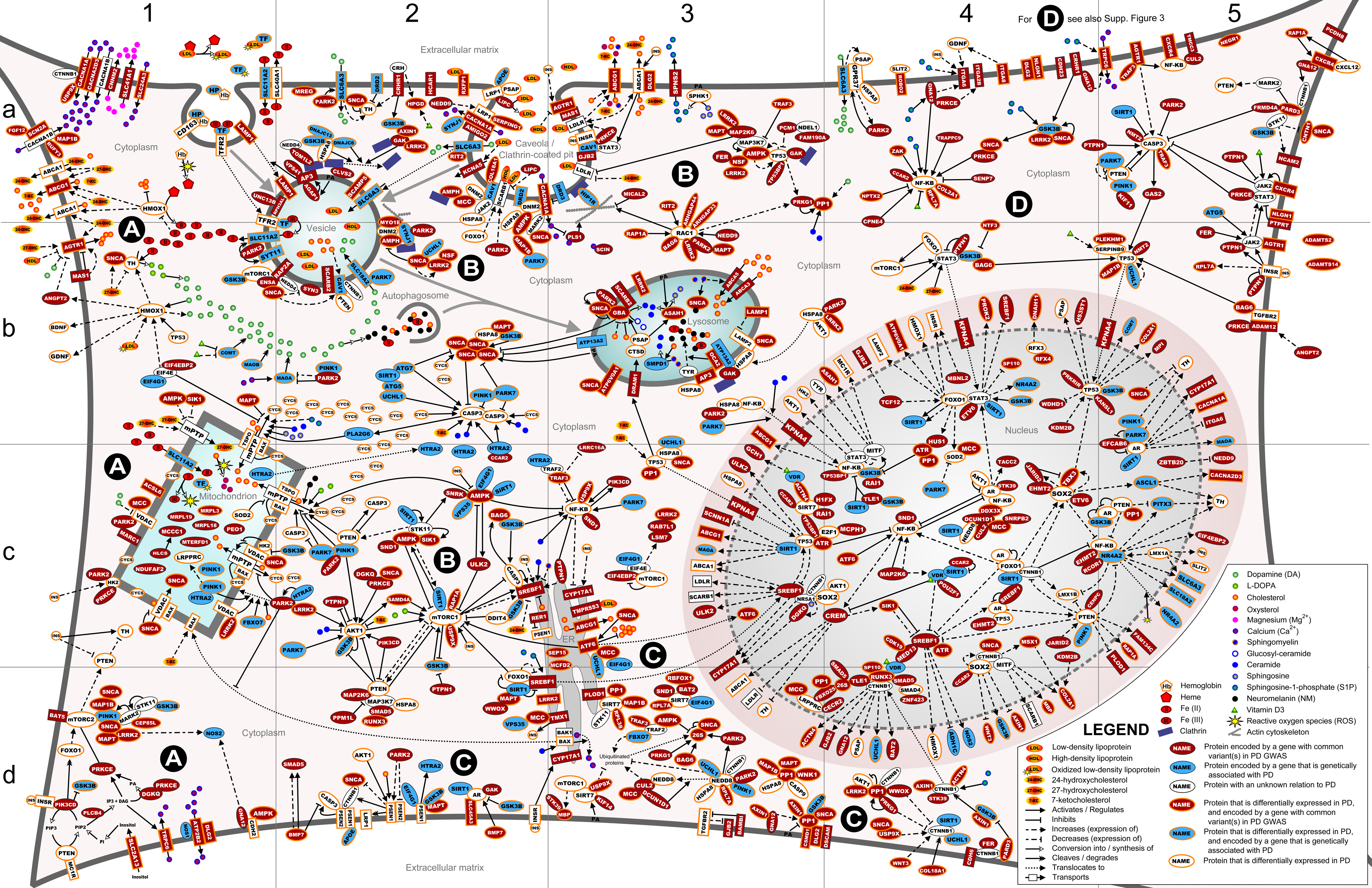

Supplement: Supplementary file 2 — Supplementary Figure 2 [file 41531_2017_15_MOESM2_ESM.tif]

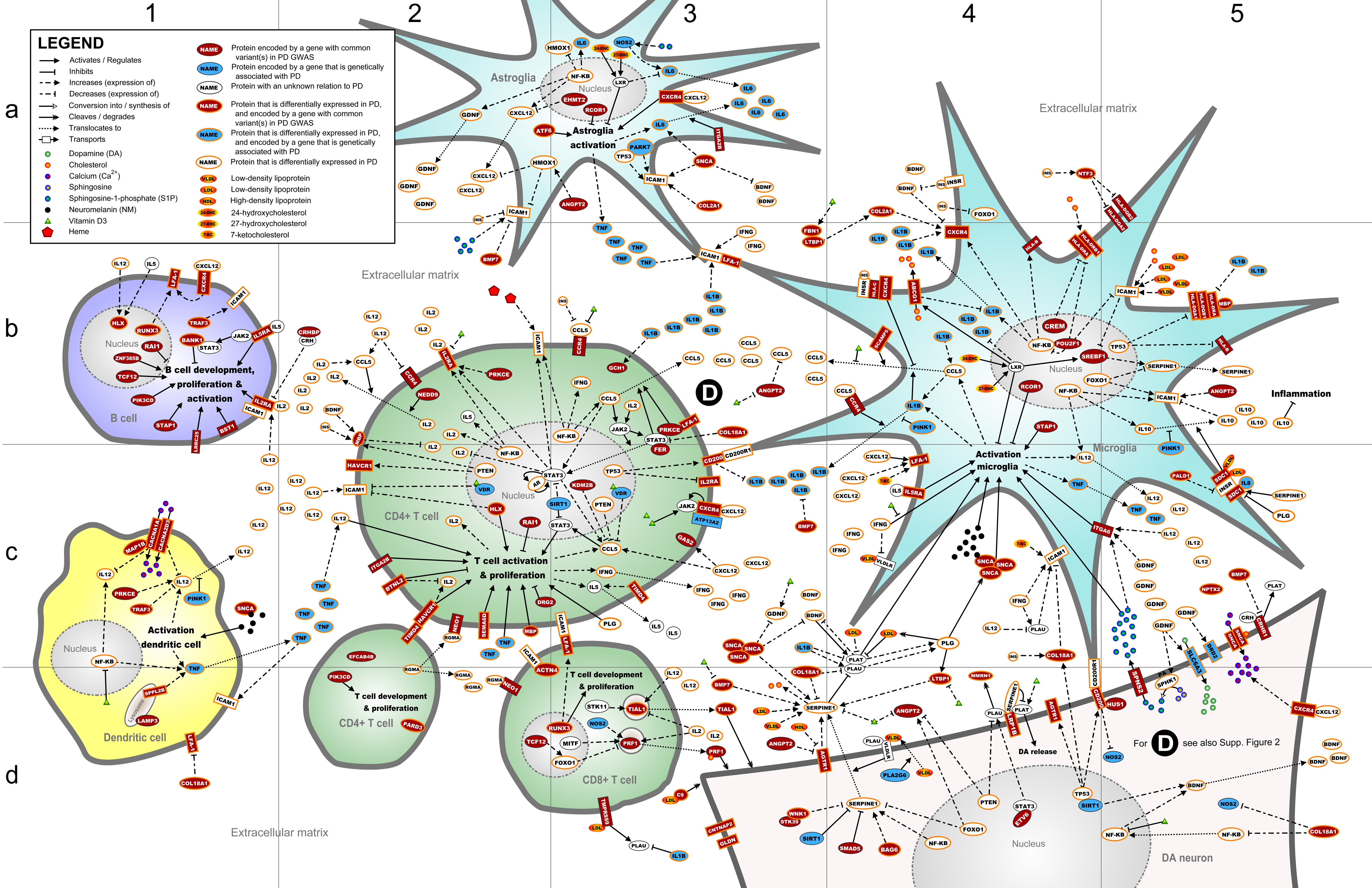

Supplement: Supplementary file 3 — Supplementary Figure 3 [file 41531_2017_15_MOESM3_ESM.tif]
